# Is periodontal disease a risk indicator for urogenital cancer? A systematic review and meta-analysis of cohort studies

**DOI:** 10.3389/fonc.2022.697399

**Published:** 2022-08-09

**Authors:** Weiqi Li, Simin Wang, Yuhan He, Yongshang Zhang, Shanfeng Lin, Dongdong Cen, Li Lin

**Affiliations:** ^1^ State Key Laboratory of Oral Diseases, National Clinical Research Center for Oral Diseases, Research Unit of Oral Carcinogenesis and Management, West China Hospital of Stomatology, Sichuan University, Chengdu, China; ^2^ School and Hospital of Stomatology, China Medical University, Shenyang, China

**Keywords:** urogenital cancer, periodontal disease, systematic review, meta-analysis, cohort studies

## Abstract

**Objectives:**

The objective of the present work was to conduct a systematic review and meta-analysis to assess the association between periodontal disease (PD) and urogenital cancer (UC) risk.

**Materials and methods:**

An electronic search in PubMed, EMBASE, the Cochrane Library, and Web of Science was conducted using MeSH terms to identify cohort studies published before May 17, 2022. Cohort studies examining the association between PD and UC risk were included. We used a random-effects model to summarize the effect sizes with 95% confidence intervals (CIs) of the included studies with PD as the indicator and UC as the outcome.

**Results:**

Eleven cohort studies met the inclusion criteria. Our results suggest that PD patients increases the risk of UC by 1.24-fold (hazard ratio (HR), 1.24; 95% CI, 1.17-1.31; I^2^, 22.4%). The strength of the sensitivity analysis and cumulative meta-analysis confirmed the reliability of the results.

**Conclusion:**

We found that PD is a potential risk factor for UC. Our results indicate that along with the decrease in the incidence of PD,PD treatment may help prevent UC. We hope that our study will raise awareness of periodontal health, thereby reducing the incidence of UC.

**Systematic Review Registration:**

https://www.crd.york.ac.uk/prospero/, identifier CRD42021244405.

## 1 Introduction

Urogenital cancer (UC) is a general term for cancers of the urogenital system. These include uterine cancer, ovarian cancer, prostate cancer UC and other male and female genital cancers, as well as kidney cancer, bladder cancer and other urologic cancers (1, 2). Cancer of the urogenital system is widely considered due to its high incidence and mortality (3–7). Due to the special function and location of the urogenital, the treatment of cancer often brings great trouble to patients’ lives (4, 8, 9). How to recognize UC and prevent its development and occurrence have become vital issues, and increasing research is focusing on how to prevent risk factors for UC (10–12). A large amount of evidence has confirmed that chronic inflammation may also be a risk factor for UC (13–18).

Periodontal disease (PD)is a common disease often manifests as chronic inflammation that compromises the integrity of periodontal tissues and other tooth-supporting tissues could cause loose teeth and tooth loss, including periodontitis, gingival disease and developmental and acquired deformities and conditions affecting the periodontium (19). PD not only causes a heavy economic burden but also greatly reduces the quality of life of patients (20, 21). From 2009 to 2012, 46% of American adults had periodontitis, and 8.9% had severe periodontitis (22). A variety of systemic diseases and conditions can affect the course of periodontitis or have a negative impact on the periodontal attachment apparatus, PD also affects overall health (23). Close associations of atherosclerosis and rheumatoid arthritis with PD have been widely reported (24–28). Whether PD is a risk factor for cancer has also become a hot topic. Studies have shown that PD increases the risk of pancreatic cancer and breast cancer (29, 30), and PD has been recognized as a risk indicator for gastrointestinal cancer (31). Persistent periodontal infection can lead to the spread of periodontal pathogens to many parts of the body, and the colonization of oral bacteria is closely related to the occurrence and development of cancer in many body parts, including the urogenital system (32–35). Periodontitis may lead to low-grade systemic inflammation and consequently could be associated with UC.

Existing evidence indicates that there may be a correlation between PD and the risk of UC, but the results of epidemiological studies on the correlation between the two are inconsistent (36–46). Considering that no systematic study has summarized the epidemiological results of the effects of PD on UC risk, the objective of the present work was to conduct a systematic review and meta-analysis to assess the association between periodontal disease (PD) and urogenital cancer (UC) risk.

## 2 Materials and methods

Our meta-analysis was conducted and reported according to the PRISMA 2020 statement (47). The protocol for this systematic review was registered on PROSPERO (CRD42021244405).

### 2.1 Eligibility criteria

#### 2.1.1 Inclusion criteria (PICOS)

Population: Adults; Indicator: Patients with PD; Comparison: Adults without a history of PD; Outcomes: The relationship between PD and UC risk with adjusted effect sizes (odds ratios (ORs)/relative risks (RRs)/hazard ratios (HRs)) and 95% confidence intervals (95% CIs); Study design: Cohort study.

PD diagnoses based on questionnaires, clinical periodontal examinations and radiographic examinations or other credible forms of medical records are acceptable. The diagnosis of UC is accepted in the form of medical records, pathological diagnosis records, and records from the cancer registry.

#### 2.1.2 Exclusion criteria

No full-text article available; Not published in an English-language peer-reviewed journal or studies not written in English; Investigation of tooth loss rather than PD.

### 2.2 Search strategy

#### 2.2.1 Electronic search

A systematic search of the PubMed, EMBASE, Web of Science, and Cochrane Library electronic databases was conducted up to May 17, 2022, to identify research on the correlation between PD and UC risk. We used Medical Subject Headings (MeSH) terms for document retrieval. PD MeSH included periodontal disease, periodontitis, gingivitis, peri-implantitis etc. UC MeSH included Urogenital Neoplasms, Genital Neoplasms, Uterine Neoplasms etc. Details of the complete search strategy can be found in **Appendix Table 1**.

#### 2.2.2 Manual search

Articles published by Journal of Clinical Periodontology and Journal of Periodontology from Jan 1,2000 to May 17, 2022, were reviewed. All references of the systematic reviews evaluating the relationship between PD and cancer risk were manually searched (30, 48–54).

### 2.3 Selection process

If data from multiple studies were derived from the same cohort, we selected the most representative study for data extraction based on follow-up time and data integrity. Two authors independently screened each record and each report retrieved used Endnote (version 20.0.0), if a disagreement occurred, a decision was made through consultation with the third author.

### 2.4 Data extraction

The information and data we extracted from the included studies were as follows: study identification (first author’s last name and published year), study design (cohort design and period), sample (sample characteristics, cohort population and country), length/age (the length of average follow-up durations and the included age of participants), gender, cohort (total number of participants), event (number of UC patients), PD diagnosis, UC diagnosis (diagnosis and the code of International Classification of Diseases), outcomes (incidence or mortality), UC types and periodontal status, effect sizes and their 95% CIs, and adjustment variables. The extracted data are shown in **Tables 1**, **2**.

**Table 1:** Characteristics and quality assessments of the included studies.

Author(year)	Study design(period)	Sample	Lenth/Age (yrs)	Gender	Cohort^∑^(Male/Female)	Event	PD diagnosis	UC diagnosis(Code)	Outcomes	UC type	Periodontal status	Risk estimates(95%-CI)
Hujoel 2003 (40)	Prospective (1971-1992)	NHANES ; USA	10/ 25-74	Male	4,466	67	Dental examination (Russell Index)	Medical/ pathology records (ICD-9)	Cancer incidence	Prostate	Periodontitis	1.81 (0.76-4.34)
											Gingivitis	1.48 (0.56-3.94)
											PD^&^	1.66 (0.86-3.17)
Arora 2010 (36)	Prospective (1963-2004)	TSTR ; Sweden	27/ 38-77	Male	15,333 (8,371/ 6,962)	604	Self-report (Teeth mobility)	Cancer registries (ICD)	Cancer mortality	Prostate	PD	1.47 (1.04-2.07)
				Both		174				Bladder		1.13 (0.59-2.20)
				Female		123				Uterine		2.20 (1.16-4.18)
				Both		901				Total UC*		1.51 (1.14-2.02)
Babic 2015 (37)	Prospective (1998-2012)	NHS ; USA	12/ 30-55	Female	60,560	395	Self-report (Periodontal bone loss)	Medical records (No info)	Cancer incidence	Ovarian	PD	0.86 (0.64–1.15)
Chung 2016 (39)	Retrospective (2002-2004)	LHID ; China	5/ ≥40	Both	80,280 (40,380/ 39,900)	850	Dental examination (Medical records , ICD-9-CM)	Medical records (ICD-9-CM)	Cancer mortality	Urogenital	Periodontitis	1.30 (1.21–1.39)
Mai 2016 (42)	Prospective (1997-2014)	BOPS ; USA	12/ 44-66	Female	1,337	12	Dental examination (ACH)	Medical records (ICD-O-2)	Cancer incidence	Uterine	Moderate PD	0.95 (0.23–3.93)
											Severe PD	1.10 (0.20–5.93)
											PD^#^	1.01 (0.34-3.00)
Michaud 2016 (43)	Prospective (1986-2012)	HPFS ; USA	24/ 40-75	Male	19,933	696	Self- Report (Periodontal disease/bone loss)/ Dental examination (Radio graphs)	Medical records (No info)	Cancer incidence	Prostate	PD	1.17 (0.94-1.47)
				Both		222				Bladder		1.38 (0.93-2.05)
				Both		137				Kidney		1.06 (0.61-1.85)
				Both		359				Urinary+		1.26 (0.92-1.74)
				Both		1,055				Total UC^+^		1.20 (1.00-1.44)
Nwizu 2017 (46)	Prospective (1994-2013)	WHI-OS ; USA	8/ 50-79	Female	65,869	819	self- report (Periodontal or gum disease)	Medical records (ICD-O-2)	Cancer incidence	Cervix	PD	0.79 (0.29-2.18)
										Endometrial		1.08 (0.87-1.34)
										Uterine^$^		1.07 (0.86-1.32)
										Ovarian		1.14 (0.88-1.47)
										Vaginal		1.05 (0.51-2.19)
										Vulvar		1.22 (0.60-2.45)
										Genital^$^		1.10 (0.95-1.29)
						367				Bladder		1.10 (0.81-1.49)
										Kidney		1.09 (0.76-1.56)
										Urinary^$^		1.16 (0.92-1.45)
						1,186				Total UC^$^		1.19 (0.99-1.27)
						379				Genital	PD (Nonsmoker)	1.13 (0.90-1.42)
						173				Urinary		1.19 (0.84-1.68)
						552				Total UC^$^		1.15 (0.95-1.39)
Michaud 2018 (45)	Prospective (1987-2012)	ARIC study ; USA	15/ 44-66	Male	3,378	375	Dental examination (CDC-AAP)	Cancer registries (No info)	Cancer incidence	Prostate	Moderate PD	1.25 (0.94-1.64)
											Severe PD	1.24 (0.91-1.69)
											Total PD^#^	1.25 (1.01-1.53)
Heikkilä 2018 (55)	Prospective (2001-2013)	PDSCH ; Finland	10/ ≥29	Male	28,675	26	Dental examination (Medical records, KELA)	Cancer registries (ICD-10)	Cancer mortality	Prostate	Periodontitis	0.95 (0.62-1.40)
Chung 2020 (38)	Retrospective (1987-2012)	DHTCG ; China	7/ >65	Male	43,052	182	Dental examination (Diagnosed by dentists)	Medical records (ICD-9/10)	Cancer mortality	Prostate	Periodontitis	1.34 (1.02-1.76)
Kim 2020 (41)	Prospective (2002-2015)	NHIS-ES ; Korea	14/ ≥60	Male	121,240	3,622	Dental examination (CDC-AAP)	Medical records (ICD-10)	Cancer incidence	Prostate	PD	1.24 (1.16-1.32)

Cohort^∑^(Male/Female), Total number of participants in the cohort study (If there are both male and female, indicated the respective numbers of male and female in brackets); All UC* data were calculated by combining Prostate cancer, Bladder cancer and Uterine cancer data; Urinary^+^ data were calculated by combining Bladder cancer and Kidney cancer data; All UC+ data were calculated by combining Prostate cancer, Bladder cancer and Kidney cancer data; Uterine^$^ data were calculated by combining Cervix cancer and Endometrial cancer data; Genital^$^ data were calculated by combining Uterine cancer,Ovarian cancer, Vaginal cancer and Vulvar cancer data; Urinary^$^ data were calculated by combining Bladder cancer and Kidney cancer data; All UC^$^ data were calculated by combining Genital^$^ cancer and Urinary^$^ cancer data, PD^&^ data were calculated by combining Periodontitis and Gingivitis data; PD^#^ data were calculated by combining Moderate PD and Severe PD data; All of above calculation used a fixed-effects model for the analysis.

NHANES, The National Health and Nutrition Examination Survey; TSTR, The Swedish Twin Registry; NHS, The Nurses’ Health Study; LHID, Longitudinal Health Insurance Database 2000; BOPS, Buffalo Osteo Perio Study; HPFS, Health Professionals Follow-up Study; WHI-OS, Women’s Health Initiative Observational Study; ARIC, Atherosclerosis Risk in Communities study; PDSCH, The Public Dental Service of the City of Helsinki; DHTCG, The dataset of health examinations supported by the Department of Health, Taipei City Government in Taiwan; Russell Index, The Russell Index was the summary measure for periodontitis used by the original investigators in the NHANES I study; ACH, A clinical measure of cumulative history of PD that reflects alveolar bone loss resulting from long-term iterative interactions between gingival bacterial infection and the host immune response, the distance from the cementoenamel junction to the most coronal portion of the alveolar crest in a plane parallel to the long axis of the tooth; CDC-AAP, The US Centers for Disease Control and Prevention–American Academy of Periodontology (CDC-AAP) definition developed for population-based surveillance of periodontitis, which uses both CAL and pocket depth (PD) measurements periodontitis, definition developed for population-based surveillance of periodontitis, which uses both CAL and pocket depth measurements; ICD, International Classification of Diseases; KCD-7: KELA: Dentists use the classification of the Finnish Social Insurance Institution (KELA) to record treatment measures provided, and these codes were used here to detect periodontal disease.

**Table 2:** The adjusted variables and Newcastle-Ottawa quality assessment scale for cohort studies.

Author(year)	Variables of adjustment	Selection				Comparability^e^		Exposure			Total scores
		Representativeness^a^	Selection^b^	Asertainment^c^	Demonstration^d^	Important factor^f^	Additional factor^g^	Assessment^h^	Follow-up		
									Length^i^	Adequacy^j^	
Hujoel 2003 (40)	Age and gender	Specific populations were oversampled	Same source^★^	Secure record^★^	Yes^★^	No	No	Record linkage^★^	Yes^★^	Lost <3.8%^★^	6★
Arora 2010 (36)	Age, gender, education, employment, the number of siblings, smoking status, smoking status of partner, alcohol status, diabetes, BMI	Selected group of twins	Same source^★^	Self-report	Yes^★^	Yes^★^	No	Record linkage^★^	Yes^★^	No description	5★
Babic 2015 (37)	Age, OC use, tubal ligation, family history of cancer, parity, duration of oestrogen HT, duration of oestrogen and progesterone HT	Selected group of nurses	Same source^★^	Secure record^★^	Yes^★^	Yes^★^	Yes^★^	Record linkage^★^	Yes^★^	No description	7★
Chung 2016 (39)	Age, gender, urbanization level, the index year, monthly income, geographic region	Selected aged ≥40	Same source^★^	Secure record^★^	Yes^★^	Yes^★^	Yes^★^	Record linkage^★^	No	No description	6★
Mai 2016 (42)	Age, smoking status, physical activity, alcohol status, age at menopause, age at menarche, parity, use of oral contraceptives	Selected group of postmenopausal women	Same source^★^	Secure record^★^	Yes^★^	Yes^★^	No	Record linkage^★^	Yes^★^	No description	6★
Michaud 2016 (43)	Age, race/ethnicity, alcohol status, physical activity, diabetes, BMI, geographical location, height, NSAIDs use.	Selected group of doctors	Same source^★^	Self-report	No	Yes^★^	Yes^★^	Record linkage^★^	Yes^★^	Lost <4%^★^	7★
Nwizu 2017 (46)	Age and BMI	Selected group of postmenopausal women	Same source^★^	Self-report	Yes^★^	No	No	Record linkage^★^	No	Lost <2.5%^★^	4★
Michaud 2018 (45)	Age, race/ethnicity, field centre, education, smoking status, smoking duration, alcohol status, BMI, diabetes	representative^★^	Same source^★^	Secure record^★^	Yes^★^	Yes^★^	Yes^★^	Record linkage^★^	Yes^★^	Lost =21.4%	8★
Heikkilä 2018 (55)	Age, diabetes, field centre, calendar time, economic status, oral health, dental treatments	representative^★^	Same source^★^	Secure record^★^	Yes^★^	Yes^★^	Yes^★^	Record linkage^★^	Yes^★^	No description	8★
Chung 2020 (38)	Age	Selected group of older	Same source^★^	Secure record^★^	Yes^★^	Yes^★^	No	Record linkage^★^	No	No description	5★
Kim 2020 (41)	Sociodemographic factors, comorbidities, smoking status, alcohol status, regular exercise	Selected group of older	Same source^★^	Secure record^★^	Yes^★^	Yes^★^	Yes^★^	Record linkage^★^	Yes^★^	No description	7★

BMI, Body mass index; NSAIDS, nonsteroidal anti-inflammatory drugs; HRT, hormone replacement therapy; ^a^, Representativeness of the exposed cohort; ^b^, Selection of the non-exposed cohort; ^c^, Ascertainment of exposure; ^d^, Demonstration that outcome of interest was not present at start of study; ^e^, Comparability of cohorts on the basis of the design or analysis; ^f^, Study controls for select the most important factor; ^g^, Study controls for any additional factor; ^h^, Assessment of outcome; ^i^, Was follow up long enough for outcomes to occur; ^j^, Adequacy of follow up of cohorts; ^★^, Earned a star.

### 2.5 Quality evaluation and risk of bias

The Newcastle-Ottawa scale (NOS) (28) is widely used to assess the quality of literature in cohort studies. It is based on the semiquantitative principle of a star system, with a total score of nine stars. Studies with 0-3 stars are considered low-quality research with low reliability, while studies with 4-6 stars and 7-9 stars are considered medium- and high-quality research with relatively high reliability.

Considering the small number of studies included, Egger’s test and the trim-and-fill method were carried out to detect publication bias. The trim-and-fill method is a non-parametric statistical method used to estimate the number of missing studies and to assess and correct for publication bias (56).

### 2.6 Statistical analysis

Since the probability of UC occurrence is very low and all the included studies were observational cohort studies, ORs and RRs were regarded as HRs in our analysis (57, 58). In this meta-analysis, we chose HR as the common correlation measure across studies. The effect sizes and CIs provided by all studies were adjusted for some confounding factors, details could be found in **Table 2**. Due to different PD diagnoses, UC types and other factors, there was significant clinical heterogeneity. We selected the random-effects model to incorporate the effect sizes and their 95% CIs into the study. Some studies classified PD according to severity. We divided the data according to severity into two groups: Moderate PD (gingivitis) and Severe PD (periodontitis), other studies without classification were pulled into Total PD group. Studies includes multiple sites for UC or studies only overall UC data without subdivision of organs are provided were pulled into total UC group. To improve the reliability of the results, for studies that provided only the respective effect sizes and CIs for different UC types or PD severities but did not provide the total effect size and CI, we combined the data provided using a fixed-effect model to obtain the effect size and CI for total UC and total PD (30). The I² statistic was used to measure the degree of heterogeneity in the meta-analysis (59). We analysed the origin of heterogeneity through subgroup analyses and meta-regressions according to UC types, periodontal status, follow-up duration, study design, outcomes, sex, smoking status, risk of bias and location. The regression coefficient estimates the difference between the intervention effect in each subgroup and a nominated reference subgroup, and the *P* value of the regression coefficient indicates the presence of statistical significance (48). We excluded one study at a time and then applied the random-effects model to perform a sensitivity analysis of the data to evaluate the robustness of the results. We conducted a cumulative meta-analysis in chronological order to observe the changes in the results over time. Two authors completed the data extraction and analyses; and used the ‘meta’ package in the statistical software R (version 4.0.0) to independently perform all statistical analyses. If a disagreement occurred, a decision was made through consultation with the third author.

### 2.7 Narrative synthesis

Referring to the guidelines (60), we grouped different types of UC into groups for narrative synthesis. With reference to the WHO classification of cancers of the urogenital system. The three types of cancer are further subdivided according to the sources. Female genital cancer includes uterine cancer, ovarian cancer, vaginal cancer and vulvar cancer; male genital cancer is mainly prostate cancer; urologic cancers is divided into kidney cancer and bladder cancer.

## 3 Results

### 3.1 Study selection

**Figure 1:**
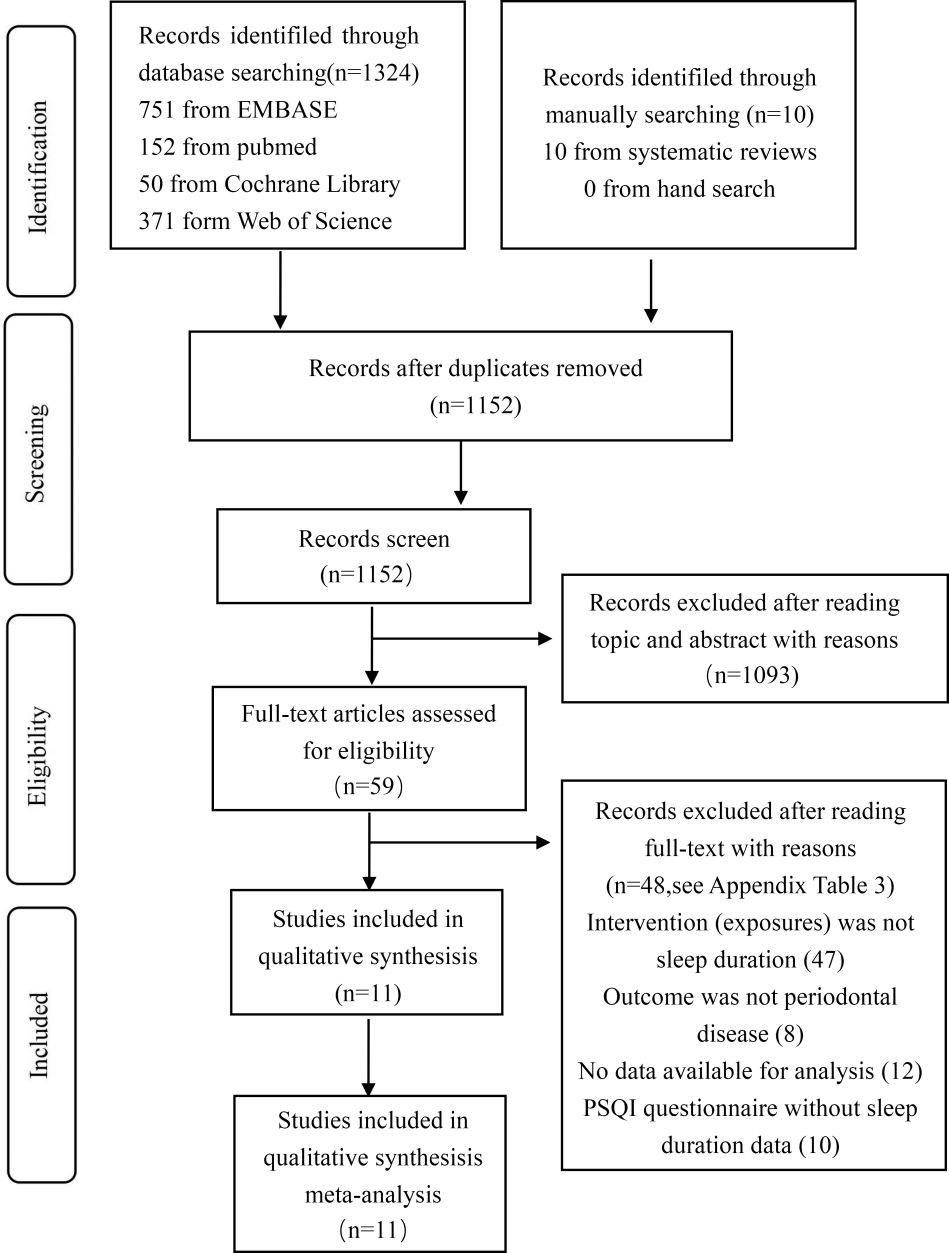
The result of search flowchart.

Our initial search strategy identified 1,334 records, including 1,324 items retrieved from the databases and 10 items retrieved manually. After removing 182 duplicate studies, the authors confidently scanned the titles and abstracts of 1,152 studies, and 991 studies not reporting an association between PD and UC risk were excluded. 59 articles met the criteria for full-text assessment, and after reading the full texts, 41 records were excluded according to the eligibility criteria. Finally, 11 eligible studies were included in our analysis. **Figure 1** shows the search flowchart. The reasons for record exclusion after reading topics and abstracts or the full texts Details can be found in **Appendix Tables 2**, **3**.

### 3.2 Study characteristics

The included cohort studies were published between 2003 and 2020 by researchers in the United States, Sweden, Finland, South Korea and China. Eleven cohort studies involving 444,123 participants and 7,485 UC patients met the inclusion criteria. The follow-up durations in the included studies ranged from 5 to 27 years. The maximum number of participants in the cohort studies was 121,240, and the minimum number was 1,337. All included studies had a quality score between 4 and 8 stars. Six medium-quality and five high-quality studies were included. **Tables 1**, **2** list the details of each study. **Table 2** shows the complete NOS quality assessment results.

#### 3.2.1 Periodontal disease and urogenital cancer diagnosis

Of the 11 studies, only 2 studies established a diagnosis of PD based on participants’ self-reports (36, 46), and the other 9 provided clear diagnostic criteria (37–43, 45, 55). The diagnosis of UC in four studies was derived from death data from cancer registries (36, 38, 39, 55), and the remaining seven studies were derived from clinical diagnosis in medical records (37, 40–43, 45, 46). Eight studies provided ICD codes for cancer diagnosis (36, 38–42, 46, 55), while the remaining three did not (37, 43, 45).

#### 3.2.2 Data adjustment for confounding factors

Except for one study that adjusted only for age (38), the remaining studies adjusted for multiple confounding factors. Four studies adjusted for smoking status, five for alcohol status (36, 41–43, 45), four for diabetes status (36, 43, 45, 55), three for sex (36, 39, 40) and four for body mass index (36, 43, 45, 46). Other adjusted factors included employment (36), the number of siblings (36), physical activity (42), comorbidities (41), regular exercise and sociodemographic factors (41). Details of the variables for which the analyses were adjusted can be found in **Table 2**.

### 3.3 Narrative synthesis

Of the 11 studies, 4 studies provided the overall effect value of PD and UC risk and its 95% CI, and four other studies investigated the relationship between PD and female genital cancer. We included seven items about male genital cancer and 3 studies that provided data on urologic cancers. We combined data from different studies through random effects models and analysed these data through subgroup analysis and meta regression. See **Table 3** for specific data, and the specific studies and data of each group are shown in **Appendix Table 5**.

**Table 3:** Results of subgroup analyses and meta-regression.

Subgroups	No. of studies	Heterogeneity		Effect model	Meta-analysis			*P* for Meta regression		
		I^2^ (%)	*P*		HR	95%CI	*P*	*R^2^*	*P*	
**Urogenital cancer diagnoses**										
**Total UC**	4	7.6	0.355	Random	1.27	1.19-1.35	<0.001***	100%		
**Female genital cancer**										
Uterine	3	0	0.939	Random	1.08	0.80-1.32	0.480		0.118	
Ovarian	2	50.3	0.156	Random	1.00	0.76-1.32	0.996		0.023*	
Vaginal	1	/	/	/	1.05	0.51-2.19	0.890		0.604	
Vulvar	1	/	/	/	1.22	0.60-2.45	0.580		0.904	
Genital^#^	1	/	/	/	1.10	0.95-1.29	0.220		0.077	
Overall^#^	4	0	0.511	Random	1.05	0.92-1.20	0.475		0.008**	
**Male genital cancer**										
Prostatic	7	0	0.662	Random	1.24	1.18-1.31	<0.001***		0.532	
Genital	/	/	/	/	/	/	/	/	/	
Overall^$^	7	0	0.662	Random	1.24	1.18-1.31	<0.001***		0.532	
**Urologic cancer**										
Bladder	3	0	0.664	Random	1.19	0.95-1.49	0.135		0.560	
Kidney	2	0	0.934	Random	1.08	0.80-1.46	0.612		0.293	
Urologic^&^	2	0	0.680	Random	1.19	0.99-1.43	0.061		0.503	
Overall^&^	3	0	0.908	Random	1.19	0.99-1.42	0.058		0.463	
**Periodontal status^#^**										
Total PD	5	52.2	0.079	Random	1.20	1.10-1.30	<0.001***	0.0%		
Periodontitis/Severe PD	6	0	0.671	Random	1.29	1.21-1.37	<0.001***		0.189	
Gingivitis/Moderate PD	3	0	0.879	Random	1.25	0.96-1.62	0.085*		0.848	
**Follow-up duration**										
≥10 years	8	32.2	0.171	Random	1.23	1.16-1.30	<0.001***	0.0%		
<10 years	3	0	0.448	Random	1.27	1.19-1.36	<0.001***		0.346	
**Study design**										
Prospective	9	23.9	0.231	Random	1.22	1.16-1.28	<0.001***	0.0%	
Retrospective	2	0	0.833	Random	1.30	1.22-1.39	<0.001***		0.127	
**Outcomes**										
Cancer incidence	7	13.2	0.329	Random	1.22	1.15-1.28	<0.001***	0.0%		
Cancer mortality	4	11.2	0.337	Random	1.30	1.22-1.39	<0.001***		0.107	
**Gender^$^**										
Male and female	3	0	0.409	Random	1.30	1.22-1.38	<0.001***	0.0%		
Female	3	50.5	0.133	Random	1.04	0.79-1.38	0.501		0.039	
Male	5	0	0.604	Random	1.24	1.17-1.32	<0.001***		0.321	
**Risk of bias**										
High	5	44.6	0.125	Random	1.17	1.05-1.30	0.011*	0.0%		
Medium	6	0	0.599	Random	1.29	1.21-1.36	<0.001***		0.178	
**Smoking status adjustment^&^**										
No	6	59.5	0.030	Random	1.20	1.04-1.35	0.001***	0.0%		
Yes	4	0	0.608	Random	1.25	1.18-1.33	<0.001***		0.568	
Non-smokers cohort	2	0	0.751	Random	1.18	1.03-1.34	0.016*		0.685	
**Location**										
America	6	15.9	0.312	Random	1.17	1.08-1.28	<0.001***	0.0%		
Asia	3	0	0.574	Random	1.27	1.21-1.33	<0.001***		0.121	
Europe	2	70	0.061	Random	1.23	0.78-1.93	0.389		0.441	
**PD diagnose**										
Dental examination	6	0	0.569	Random	1.27	1.21-1.33	<0.001***	0.0%		
Self-Report	4	60.5	.055	Random	1.19	0.98-1.45	0.086		0.380	
Examination & Self-Report	1	/	/	/	1.19	1.05-1.35	0.006**		0.364	

Genital^#^: This group included studies that provided data on overall female genital cancer; Over all^#^: This group pooled all studies that mentioned part or all of the cancers of the female genital cancers; Overall^$^: Studies on male genital cancer only have data on prostate cancer; Urologic^#^: This group included studies that provided data on overall female urologic cancer; Over all^#^: This group pooled all studies that mentioned part or all of the cancers of the urologic cancers.

Some studies provide more than one item of data, and the number of studies is only calculated once when the data need to be summarized.

Periodontal status^#^: Some studies classified PD according to severity. We divided the data according to severity into two groups: Moderate PD (gingivitis) and Severe PD (periodontitis), other studies without classification were pulled into Total PD group; Gender^$^: Some studies observed both men and women, but some cancers such as prostate cancer only affected men. We included these data in the male group together with other studies that only observed men, while some cancers such as uterine cancer only affected women. We included these data in the female group; Smoking status adjustment^&^: This subgroup was grouped according to whether the study adjusted for smoking. Nwizu 2017 did not adjust for smoking status, but provided additional data on non-smokers, Michaud 2016 observed on non-smokers. We divided the two sets of data into Non-smoker cohort group.

Significant code: ‘*’ <0.05; ‘**’ <0.01; ‘***’ <0.001.

#### 3.3.1 Urogenital cancer

Among the four studies, three studies provided cancer data in multiple kinds of urogenital (36, 43, 46). We combined them using a fixed-effect model to obtain UC data. Another study directly provided the overall UC data (39). Two studies involving both males and females found that PD and UC risk were closely related, with significant differences (Arora 2010, HR, 1.51; Chung 2016, HR, 1.30) (36, 39). Another study involved both sexes (Michaud 2016, HR, 1.20) (43), and the study only investigated females (Nuwizu 2017, HR, 1.19) (46) indicated that PD increased the risk of UC, but their results were not significant.

#### 3.3.2 Female genital cancer

Four studies were included in this section: three mentioned uterine cancer (Arora 2010, HR, 1.20; Mai 2016, HR, 1.01; Nuwizu 2017, HR, 1.07) (36, 42, 46), two involved ovarian cancer (Babic 2015, HR, 0.86; Nuwizu 2017, HR, 1.14) (37, 46) and one study mentioned vaginal cancer (Nuwizu 2017, HR, 1.05) (46) and vulvar cancer (Nuwizu 2017, HR, 1.22). In addition, another study provided overall data on female genital cancer (Nuwizu 2017, HR, 1.10). All the results were not significant.

#### 3.3.3 Male genital cancer

Seven studies mentioned that data on male genital cancer are all focused on prostate cancer. Four of the studies found that patients with PD were more likely to develop prostate cancer (Arora 2010, HR, 1.47; Michaud 2018, HR, 1.25; Chung 2016, HR, 1.34; Kim 2020, HR, 1.24) (36, 39, 41, 45). The other three studies did not find a significant association (Hujoel 2003, HR, 1.66; Michaud 2016, HR, 1.17; Heikkilä 2018, HR, 0.95) (40, 43, 55).

#### 3.3.4 Urologic cancers

Research on urologic cancers focuses on bladder cancer and Kidney cancer. Two studies provided data on both bladder cancer (Michaud 2016, HR, 1.38; Nuwizu 2017, HR, 1.10) (43, 46) and kidney cancer (Michaud 2016, HR, 1.06; Nuwizu 2017, HR, 1.19). We used a fixed-effects model to combine the data of the two cancers as the result of urologic cancers (Michaud 2016, HR, 1.26; Nuwizu 2017, HR, 1.16). In addition, another study only provided data on bladder cancer (Arora 2010, HR, 1.13) (36). None of the studies found a clear correlation between PD and urologic cancers.

### 3.4 Results of the meta-analysis

#### 3.4.1 Overall estimation and sensitivity analysis

**Figure 2:**
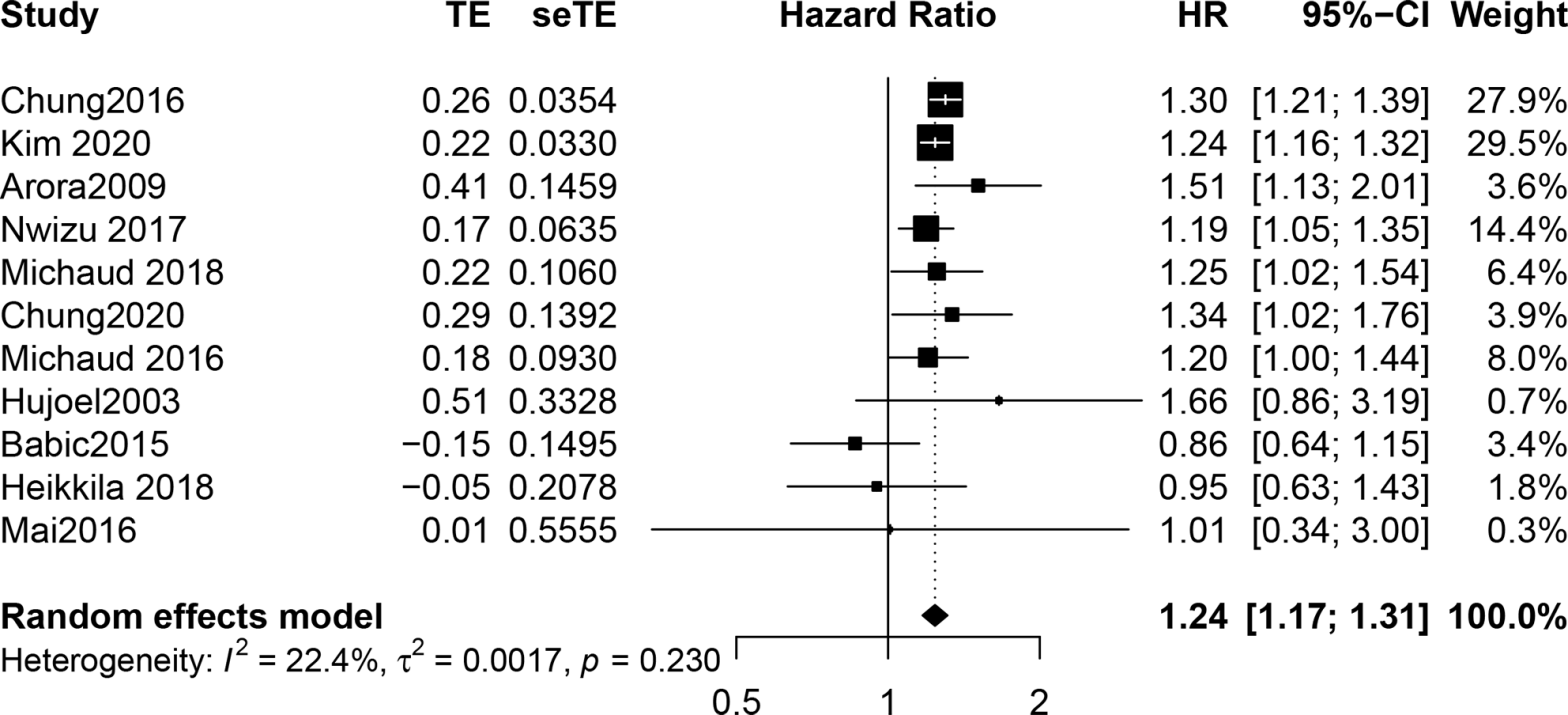
Forest plot incorporating the results of all the studies.

**Figure 3:**
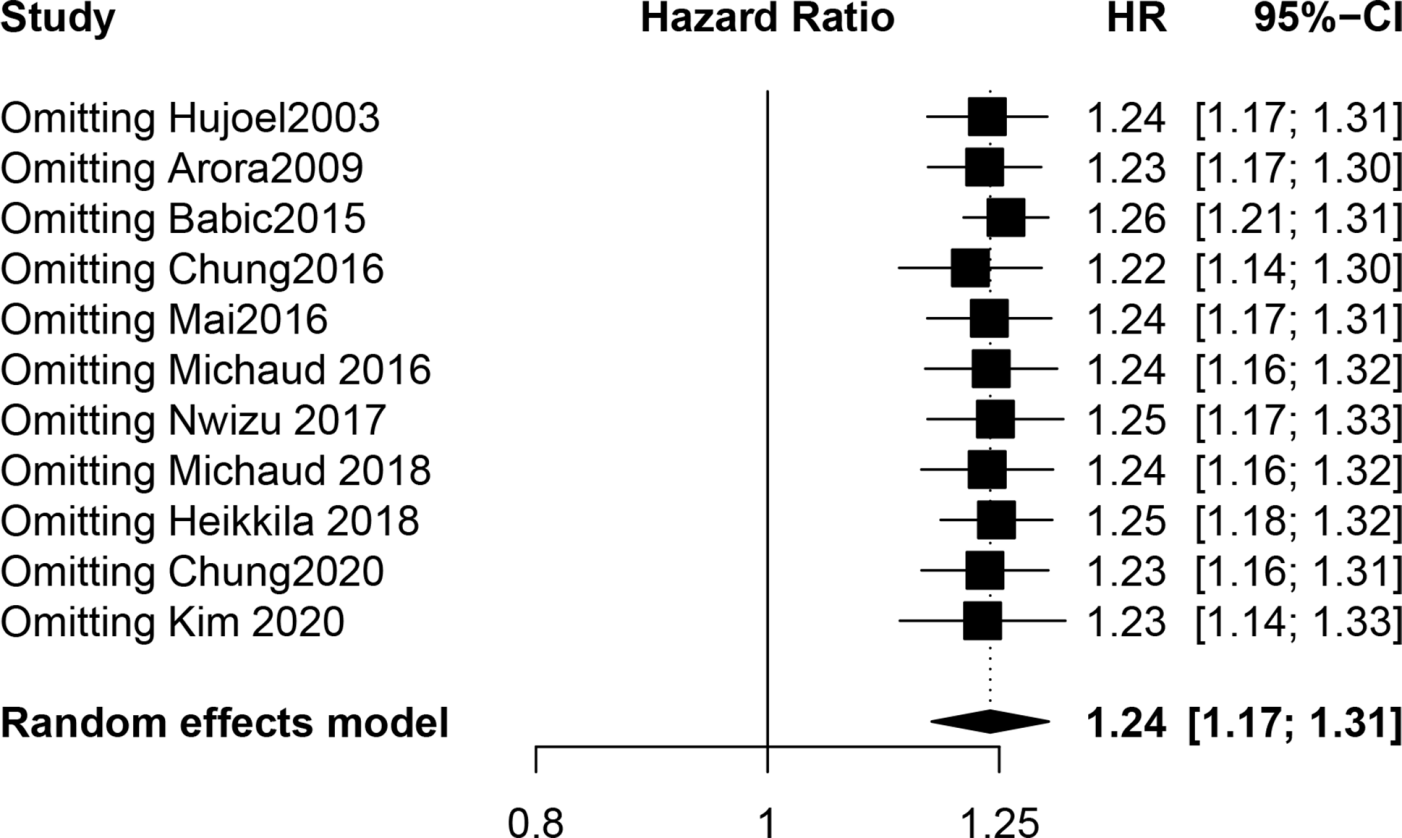
The result of sensitivity analysis.

Our meta-analysis showed that the risk of UC in PD patients was 1.24 times higher than that in patients without PD (HR, 1.24; 95% CI, 1.17-1.31; *P* < 0.001). The I² statistic showed no heterogeneity (I^2^ = 22.4%; the heterogeneity *P* was 0.230). A forest plot incorporating the results of all the studies is shown in **Figure 2**. After removing each study, the combined effect values were statistically significant. Other details are shown in **Figure 3**.

#### 3.4.2 Subgroup analysis and meta-regression

Subgroup analysis and meta-regression were carried out according to predefined groupings. The results showed that the HRs of each subgroup were ≥ 1.00 but not all results were significant. Random effects model used in data analysis in all subgroups.

##### 3.4.2.1 Results of subgroup analysis based on urogenital cancer diagnoses

According to UC diagnoses, the risk of cancer in PD patients was significantly increased in the study reporting urogenital cancer (HR, 1.27, *P* < 0.001) and male genital cancer (HR, 1.24, *P* < 0.001), and there was no significant relationship between PD and female genital cancer (HR, 1.05, *P* = 0.475) or urologic cancers (HR, 1.19, *P* = 0.058). The meta-regression results showed that there was a significant difference between UC and Female genital cancer (*P* = 0.008), as well as UC and ovarian cancer (*P* = 0.023). More details about the results of subgroup analysis and meta-regression based on urogenital cancer diagnoses are shown in **Table 3**.

##### 3.4.2.2 Other results of subgroup analyses and meta-regression

The subgroup analyses according to the follow-up duration, study design, outcomes, risk of bias and smoking status adjustment showed that the results were all similar (HRs, from 1.18 to 1.30) and statistically significant (*P* < 0.01). According to periodontal status, the HRs of the three groups were similar (total PD, HR, 1.20, P < 0.001; severe PD, HR, 1.29, P < 0.001; moderate PD, HR, 1.25, P = 0.085), and only moderate PD (gingivitis) had no significant correlation with UC. Grouped according to gender, the HR of the study that only observed females was significantly lower than that of the other two groups (male and female, HR, 1.30, *P* < 0.001; male, HR, 1.28, *P* = 0.007; female, HR, 1.05, *P* = 0.501), and there was no significant difference in the results of only the female group. Studies in America and Asia have found a correlation between PD and UC (America, HR, 1.18, *P* < 0.001; Asia, HR, 1.27, *P* < 0.001), but Europe has not yet (Europe, HR, 1.22, *P* = 0.389). The meta-regression results showed that the *P* value in each group was greater than 0.05. More details about the other results of subgroup analyses and meta-regression are shown in **Table 3**.

#### 3.4.3 Publication bias and cumulative meta-analysis

**Figure 4:**
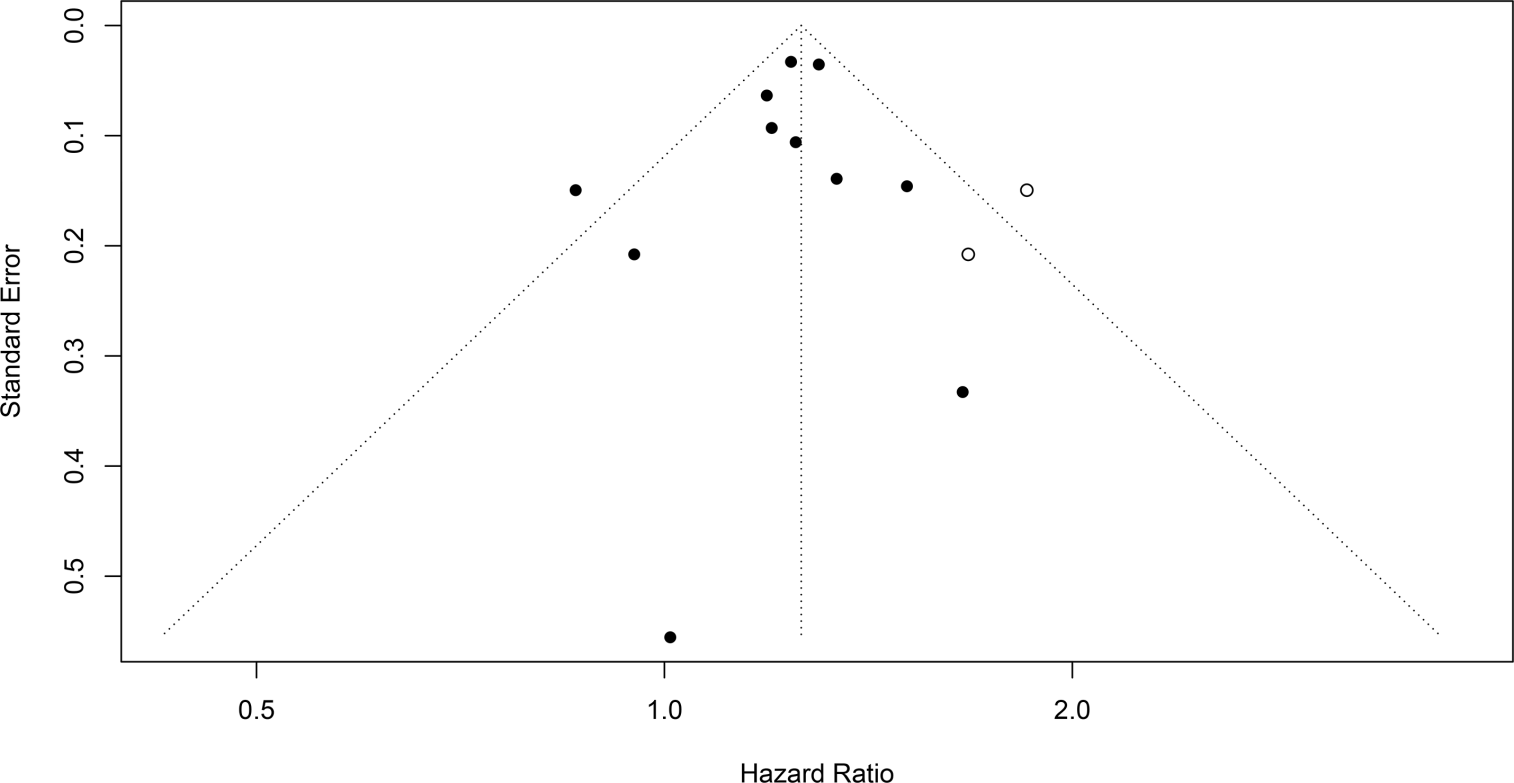
The result of the trim-and-fill method.

**Figure 5:**
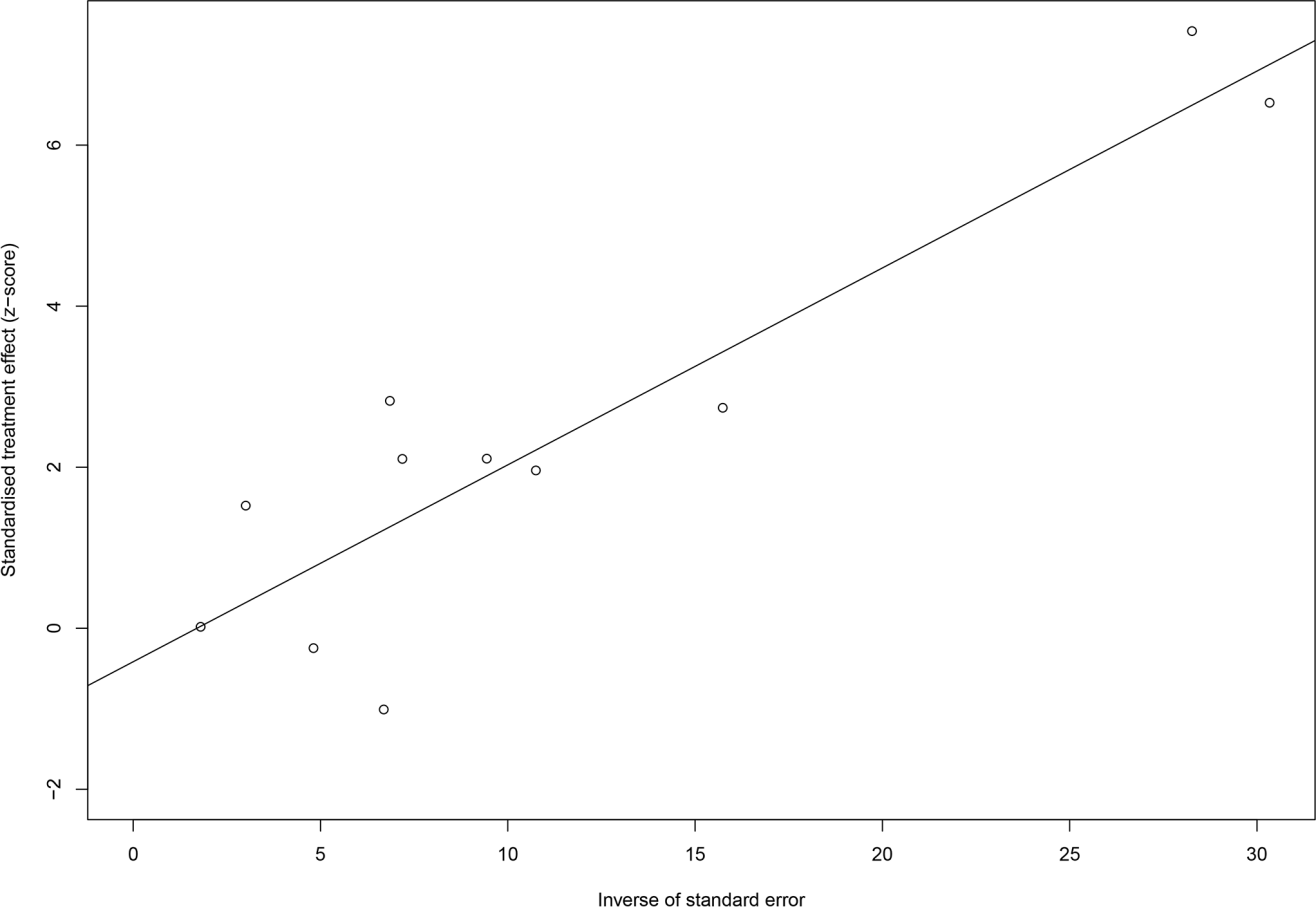
The result of Egger’s test.

**Figure 6:**
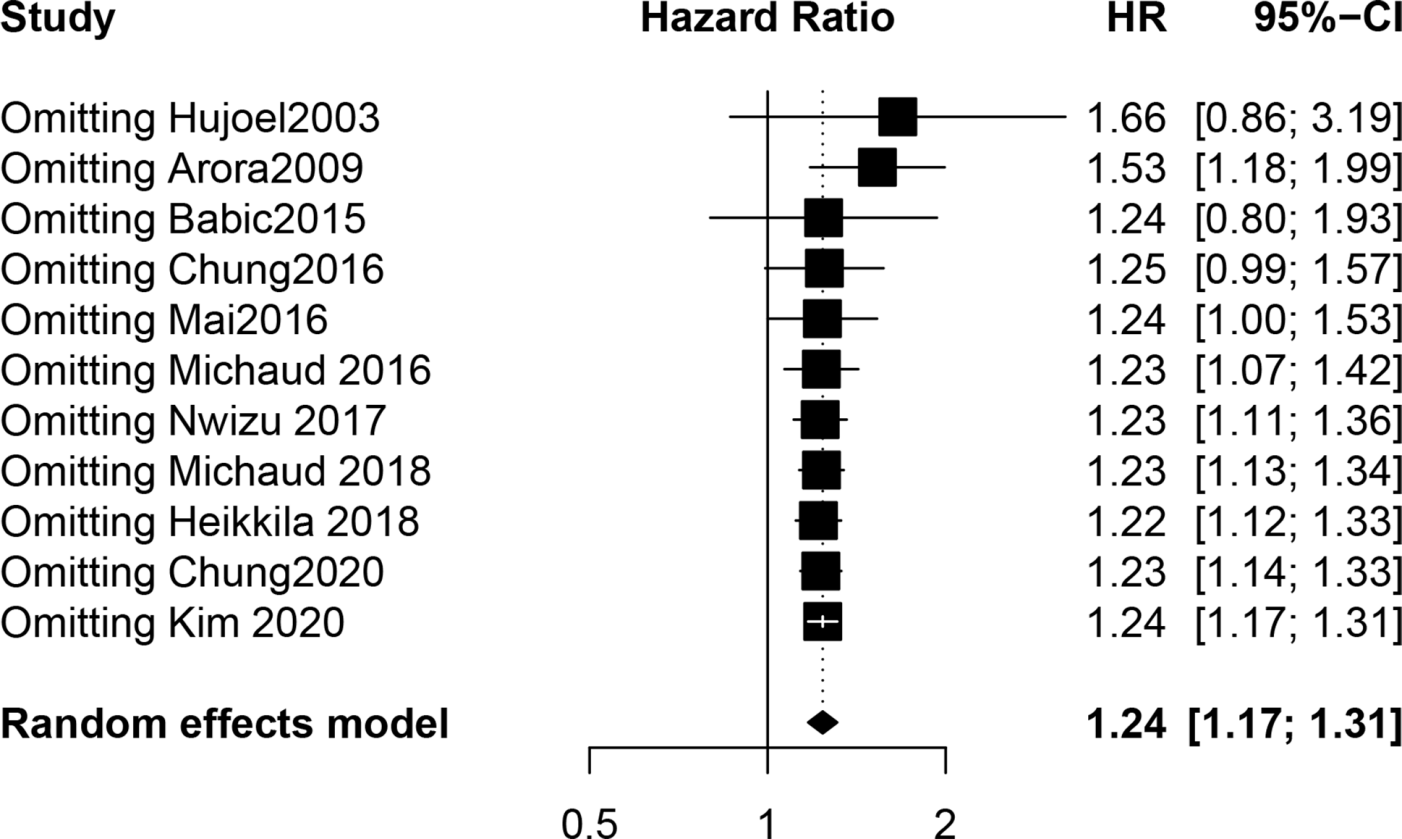
The result of cumulative meta-analysis.

Egger’s test (*P* = 0.473) indicated no obvious publication bias, and the trim-and-fill method with 2 added studies yielded robust results (*P* < 0.001, random-effects model). More details about Egger’s test and the trim-and-fill method are shown in **Figures 4**, **5**. The cumulative meta-analysis also showed that our results were stable over time. After cumulative analysis in chronological order, HR point estimates and interval estimates tended to be stable. *P* < 0.05 was considered statistically significant, and the influence of PD on UC first achieved significance in 2016. Subsequent studies narrowed the range of CIs and improved the reliability of the results. The results of the cumulative meta-analysis are shown in **Figure 6**.

## 4 Discussion

To date, no systematic review or meta-analysis has examined the relationship between PD and UC risk. All eleven studies included in our study were medium- and high-quality cohort studies, and nine were prospective studies. Cohort studies first aim to determine exposure factors and then to explore the results by longitudinal prospective observation. Since exposure factors are evaluated first and then results are observed, this study design is more able to test the causal relationship between a certain factor and a disease. Our meta-analysis of cohort studies can prove the potential causal relationship between PD and UC risk. Our results showed that PD increased the risk of UC by 1.24 times (CI, 1.17-1.31, *P* < 0.001). The I² statistic was 22.4% and *P* = 0.230 for heterogeneity; thus, no significant heterogeneity was observed. Subgroup analysis revealed that there were differences between females and other groups according to gender, but the meta-regression analysis found that the difference was not significant. Combining the results of subgroup analysis and meta-regression, we found that there was a significant difference between female genital cancer and UC, which is considered the source of heterogeneity. We believe that the particularity of Female genital cancer is caused by the physiological structure, and the connectivity between the male genital system and the urologic system has caused the results of the two to be closer. The sensitivity analysis, trim-and-fill method and subgroup analysis showed that our research results were reliable. No publication bias was found in Egger’s test. The results of the cumulative meta-analysis indicated that the results tended to be moderate.

Periodontal infection and inflammatory reactions caused by PD correlate with many general diseases (24–28). Many cancers are caused by chronic microbial infections and chronic inflammation damage (61–63). PD and oral bacteria have a strong association with the occurrence and development of cancers in the endocrine system, including the mammary glands and pancreas (29–31, 64). In addition to the chronic inflammatory state caused by cancer, periodontal pathogens may promote cancer by affecting cell proliferation and NF-κB activation and inhibiting apoptosis (63). In addition, periodontal pathogens may release carcinogens (65). The plaque of patients with PD is often not under reasonable control, causing periodontal pathogens to disseminate and accumulate in some parts of the body through the digestive tract, respiratory tract or endocrine system, promoting the development and occurrence of cancer (29–35, 64).

Anaerobic bacteria, such as *Fusobacterium* and *Porphyromonas*, are not only pathogens of PD but also pathogenic bacteria of the urinary system (66).A study found that some periodontal pathogens, including *Porphyromonas*, *Fusobacterium* and Treponema, existed in the urogenital fluid of patients who had PD with chronic prostatitis and benign urogenital hyperplasia (67). PD and the relationship between its pathogens and chronic kidney disease, including nephritis, have also been widely studied (68–70). The inflammatory status induced by chronic prostatitis and urinary tract infection has been reported to be closely correlated with UC (14, 71–73).In addition, we observed a high correlation between PD and prostate cancer risk (HR, 1.24, *P* < 0.001), which is a Urogenital System organ specific to male, which we believe explains to some extent why PD is more significantly associated with UC risk in male. Moreover, *Fusobacterium* was found in the tumours of UC patients (32, 65). We believe that invasion of oral pathogens after epithelial or mucosal traumatic injury may cause urogenital disease development and then have an impact on the subsequent progression of cancer. Although we lack direct evidence of the association between PD and UC, all signs indicate that PD and oral pathogens are inextricably linked to UC.

This meta-analysis has some notable advantages. First, this study was the first meta-analysis in this field. Our study is innovative in exploring the correlation between PD and UC. Second, our study included all studies that met the inclusion criteria, and no publication bias was observed; moreover, the effect sizes and CIs provided by all studies were adjusted for many confounding factors. Third, the studies we included were all cohorts, and the studies had a high level of evidence, suggesting a potential causal relationship between PD and UC risk while minimizing selection and recall bias. Fourth, the average follow-up time in the included studies was more than 10 years, which was long enough to avoid the effects of insufficient follow-up time on the results. Fifth, this study included populations in Europe, Asia and North America, thereby reducing the possible influence of nationality on the results. Finally, our meta-analysis included medium- to high-quality research, increasing the reliability of our results.

While there were strengths, there are naturally many limitations. First, as the clinical definition of PD continues to evolve with the development of the discipline, the diagnostic criteria in different studies were disparate. Even diagnosis in some early studies was based on patient self-reporting, which impacted the heterogeneity of our research. Additionally, it also affects the reliability of the results. Second, although Egger’s test did not indicate bias and the results of the trim-and-fill method were robust, there may still be potential publication bias considering the small number of included studies and articles published in only English. Third, the size of our sample and the number of included studies were small, at least ten observations should be available for each characteristic modelled and when the covariates are unevenly distributed need more. affecting the robustness of the subgroup analyses and meta-regression results. Fourth, confounding factors may have influenced the results. Although each study controlled for some confounding factors, there are still many confounding factors that were not adjusted for in the studies we included, and each study adjusted their confounding factors differently, which not only increased the heterogeneity but also affected the validity of the results. Finally, it was difficult to explore whether there was a difference in the associations between PD and UC with varying severity on account of the limited data provided by the included studies.

Due to the slow progression of UC, most patients do not exhibit significant symptoms, rendering UC difficult to recognize. Therefore, it is important to avoid risk factors that cause UC. PD can be prevented and treated. We can educate people on the correct way to brush their teeth, the harm of smoking and the significance of attending regular dental examinations to decrease PD-associated morbidity. We look forward to helping people realize the importance of maintaining their oral health. With the prevention of PD, we can decrease the risk of UC.

## 5 Conclusion

In conclusion, our study shows that the risk of UC in PD patients is increased by 24% compared with people without PD. This study found a link between PD and UC risk, reminding people to pay close attention to PD and the importance of oral health. Of course, our results show only a possible epidemiological correlation between PD and UC, and we do not understand the exact mechanism of the connection between the two. We look forward to finding direct evidence to prove the connection between the two in the future. We also hope that in the future, there will be additional large-scale, multicentre, prospective cohort studies with uniform standards to enrich and expand our research results worldwide.

## Data availability statement

The raw data supporting the conclusions of this article will be made available by the authors, without undue reservation.

## Author contributions

WL and SW designed this study. WL and SW performed search and collected data. YZ and YH re-checked data. WL, SL, and DC performed analysis. WL and SL wrote the manuscript. LL reviewed the manuscript. All authors contributed to the article and approved the submitted version.

## Funding

This work was supported by a grant from the National Nature Science Foundation of China (GM 81970942).

## Acknowledgments

The authors would like to thank CDD team for excellent technical support and Professor Dongmei Zhang for critically reviewing the manuscript.

## Conflict of interest

The authors declare that the research was conducted in the absence of any commercial or financial relationships that could be construed as a potential conflict of interest.

## Publisher’s note

All claims expressed in this article are solely those of the authors and do not necessarily represent those of their affiliated organizations, or those of the publisher, the editors and the reviewers. Any product that may be evaluated in this article, or claim that may be made by its manufacturer, is not guaranteed or endorsed by the publisher.
