# Supplementary material for: Is periodontal disease a risk indicator for urogenital cancer? A systematic review and meta-analysis of cohort studies
Source: Front Oncol. 2022 Aug 9;12:697399. doi: 10.3389/fonc.2022.697399 (PMC9395701; doi:10.3389/fonc.2022.697399)
Supplement: Supplementary file 1 [file Table_1.docx]

| **Appendix Table 1. Electronic search criteria for systematic review of literature** | | |
| --- | --- | --- |
| Database | Search criteria | Results |
| EMBASE | #1 'periodontal disease'/exp | 118,645 |
|  | #2 'periodontal diseases' OR 'disease periodontal' OR 'diseases periodontal' OR parodontosis OR parodontoses OR 'pyorrhea alveolaris' | 8,967 |
|  | #3 'gingivitis'/exp OR 'periimplantitis'/exp OR 'alveolar bone loss'/exp OR 'periodontitis'/exp | 55,753 |
|  | #4 'furcation defects' OR 'defect furcation' OR 'defects furcation' OR 'furcation defect' OR gingivitides OR 'peri implantitis' OR 'peri implantitides' OR periimplantitides OR periodontitides OR pericementitis OR 'alveolar bone losses' OR 'alveolar process atrophy' OR 'alveolar process atrophies' OR 'alveolar resorption' OR 'resorption alveolar' OR 'bone loss periodontal' OR 'periodontal bone losses' OR 'periodontal bone loss' OR 'periodontal resorption' OR 'resorption periodontal' OR 'alveolar bone atrophy' OR 'bone loss alveolar' OR 'attachment loss periodontal' OR 'loss periodontal attachment' OR 'periodontal attachment loss' | 4,641 |
|  | #5 #1 OR #2 OR #3 OR #4 | 123,794 |
|  | #6 'urogenital tract tumor'/exp | 963,223 |
|  | #7 'urogenital neoplasms' OR 'urogenital neoplasm' OR 'neoplasm, urogenital' OR 'neoplasms, urogenital' OR 'genito-urinary neoplasm' OR 'genito-urinary neoplasms' OR 'neoplasm, genito-urinary' OR 'neoplasms, genito-urinary' OR 'genitourinary neoplasms' OR 'genitourinary neoplasm' OR 'neoplasm, genitourinary' OR 'neoplasms, genitourinary' OR 'genitourinary cancer' OR 'cancer, genitourinary' OR 'cancers, genitourinary' OR 'genitourinary cancers' OR 'genito-urinary cancer' OR 'cancer, genito-urinary' OR 'cancers, genito-urinary' OR 'genito urinary cancer' OR 'genito-urinary cancers' OR 'urogenital cancer' OR 'cancer, urogenital' OR 'cancers, urogenital' OR 'urogenital cancers' | 11,243 |
|  | #8 'female genital tract tumor'/exp | 418,419 |
|  | #9 'genital neoplasms, female' OR 'neoplasms, female genital' OR 'gynecologic neoplasms' OR 'neoplasms, gynecologic' OR 'gynecologic neoplasm' OR 'neoplasm, gynecologic' OR 'female genital neoplasms' OR 'female genital neoplasm' OR 'genital neoplasm, female' OR 'neoplasm, female genital' | 1,033 |
|  | #10 'uterine tube tumor'/exp OR 'uterus cancer'/exp OR 'vagina tumor'/exp OR 'vulva tumor'/exp | 198,485 |
|  | #11 'fallopian tube neoplasms' OR 'fallopian tube neoplasm' OR 'neoplasm, fallopian tube' OR 'neoplasms, fallopian tube' OR 'fallopian tube cancer' OR 'cancer, fallopian tube' OR 'cancers, fallopian tube' OR 'fallopian tube cancers' OR 'cancer of the fallopian tube' | 1,162 |
|  | #12 'uterine neoplasms' OR 'neoplasms, uterus' OR 'neoplasm, uterus' OR 'uterus neoplasm' OR 'uterus neoplasms' OR 'neoplasms, uterine' OR 'neoplasm, uterine' OR 'uterine neoplasm' OR 'cancer of uterus' OR 'uterus cancers' OR 'cancer of the uterus' OR 'cancer, uterus' OR 'cancers, uterus' OR 'uterine cancer' OR 'cancer, uterine' OR 'cancers, uterine' OR 'uterine cancers' | 11,696 |
|  | #13 'vaginal neoplasms' OR 'neoplasm, vaginal' OR 'vaginal neoplasm' OR 'vagina neoplasms' OR 'neoplasm, vagina' OR 'neoplasms, vagina' OR 'vagina neoplasm' OR 'neoplasms, vaginal' OR 'vaginal cancer' OR 'cancer, vaginal' OR 'cancers, vaginal' OR 'vaginal cancers' OR 'cancer of the vagina' OR 'cancer of vagina' OR 'vagina cancers' OR 'vagina cancer' OR 'cancer, vagina' OR 'cancers, vagina' | 4,334 |
|  | #14 'vulvar neoplasms' OR 'neoplasm, vulvar' OR 'vulvar neoplasm' OR 'neoplasms, vulvar' OR 'vulva neoplasms' OR 'neoplasm, vulva' OR 'neoplasms, vulva' OR 'vulva neoplasm' OR 'cancer of vulva' OR 'vulva cancers' OR 'cancer of the vulva' OR 'vulva cancer' OR 'cancer, vulva' OR 'cancers, vulva' OR 'vulvar cancer' OR 'cancer, vulvar' OR 'cancers, vulvar' OR 'vulvar cancers' | 6,969 |
|  | #15 'male genital tract tumor'/exp OR 'penis tumor'/exp OR 'prostate tumor'/exp OR 'testis tumor'/exp | 319,389 |
|  | #16 'genital neoplasms, male' OR 'neoplasms, male genital' OR 'male genital neoplasms' OR 'genital neoplasm, male' OR 'male genital neoplasm' OR 'neoplasm, male genital' | 20 |
|  | #17 'penile neoplasms' OR 'neoplasms, penis' OR 'penis neoplasms' OR 'neoplasm, penis' OR 'penis neoplasm' OR 'neoplasms, penile' OR 'neoplasm, penile' OR 'penile neoplasm' OR 'cancer of penis' OR 'penis cancers' OR 'cancer of the penis' OR 'penis cancer' OR 'cancer, penis' OR 'cancers, penis' OR 'penile cancer' OR 'cancer, penile' OR 'cancers, penile' OR 'penile cancers' | 5,458 |
|  | #18 'prostatic neoplasms' OR 'prostate neoplasms' OR 'neoplasms, prostate' OR 'neoplasm, prostate' OR 'prostate neoplasm' OR 'neoplasms, prostatic' OR 'neoplasm, prostatic' OR 'prostatic neoplasm' OR 'prostate cancer' OR 'cancer, prostate' OR 'cancers, prostate' OR 'prostate cancers' OR 'cancer of the prostate' OR 'prostatic cancer' OR 'cancer, prostatic' OR 'cancers, prostatic' OR 'prostatic cancers' OR 'cancer of prostate' | 269,193 |
|  | #19 'testicular neoplasms' OR 'testicular neoplasm' OR 'neoplasm, testicular' OR 'testicular tumors' OR 'neoplasms, testis' OR 'neoplasm, testis' OR 'testis neoplasm' OR 'testis neoplasms' OR 'testicular tumor' OR 'tumor, testicular' OR 'tumors, testicular' OR 'neoplasms, testicular' OR 'tumor of rete testis' OR 'rete testis tumor' OR 'rete testis tumors' OR 'testis tumor, rete' OR 'testis tumors, rete' OR 'cancer of testis' OR 'testis cancer' OR 'cancer, testis' OR 'cancers, testis' OR 'testis cancers' OR 'cancer of the testes' OR 'cancer of the testis' OR 'testicular cancer' OR 'cancer, testicular' OR 'cancers, testicular' OR 'testicular cancers' | 26,505 |
|  | #20 'urinary tract tumor'/exp OR 'kidney tumor'/exp OR 'bladder tumor'/exp | 279,898 |
|  | #21 'urologic neoplasms' OR 'urological neoplasms' OR 'neoplasm, urological' OR 'neoplasms, urological' OR 'urological neoplasm' OR 'urinary tract neoplasms' OR 'neoplasm, urinary tract' OR 'neoplasms, urinary tract' OR 'tract neoplasm, urinary' OR 'tract neoplasms, urinary' OR 'urinary tract neoplasm' OR 'neoplasms, urologic' OR 'neoplasm, urologic' OR 'urologic neoplasm' OR 'cancer of urinary tract' OR 'urinary tract cancers' OR 'urological cancer' OR 'cancer, urological' OR 'cancers, urological' OR 'urological cancers' OR 'cancer of the urinary tract' OR 'urinary tract cancer' OR 'cancer, urinary tract' OR 'cancers, urinary tract' OR 'urologic cancer' OR 'cancer, urologic' OR 'cancers, urologic' OR 'urologic cancers' | 10,840 |
|  | #22 'kidney neoplasms' OR 'kidney neoplasm' OR 'neoplasm, kidney' OR 'renal neoplasms' OR 'neoplasm, renal' OR 'neoplasms, renal' OR 'renal neoplasm' OR 'neoplasms, kidney' OR 'cancer of kidney' OR 'kidney cancers' OR 'renal cancer' OR 'cancer, renal' OR 'cancers, renal' OR 'renal cancers' OR 'cancer of the kidney' OR 'kidney cancer' OR 'cancer, kidney' | 40,461 |
|  | #23 'ureteral neoplasms' OR 'neoplasm, ureteral' OR 'ureteral neoplasm' OR 'neoplasms, ureteral' OR 'ureter neoplasms' OR 'ureter neoplasm' OR 'neoplasms of ureter' OR 'cancer of ureter' OR 'ureter cancers' OR 'ureter, cancer of' OR 'ureter cancer' OR 'ureteral cancer' OR 'cancer, ureteral' OR 'cancers, ureteral' OR 'ureteral cancers' OR 'cancer of the ureter' | 1,625 |
|  | #24 'urethral neoplasms' OR 'neoplasms, urethral' OR 'neoplasm, urethral' OR 'urethral neoplasm' OR 'urethra neoplasms' OR 'neoplasm, urethra' OR 'neoplasms, urethra' OR 'urethra neoplasm' OR 'cancer of urethra' OR 'urethra cancers' OR 'urethra cancer' OR 'cancer, urethra' OR 'cancers, urethra' OR 'urethral cancer' OR 'cancer, urethral' OR 'cancers, urethral' OR 'urethral cancers' OR 'cancer of the urethra' | 1,106 |
|  | #25 'urinary bladder neoplasms' OR 'neoplasms, urethral' OR 'urinary bladder neoplasm' OR 'neoplasms, bladder' OR 'bladder neoplasms' OR 'bladder neoplasm' OR 'neoplasm, bladder' OR 'bladder tumors' OR 'tumor, bladder' OR 'tumors, bladder' OR 'urinary bladder cancer' OR 'cancer, urinary bladder' OR 'malignant tumor of urinary bladder' OR 'cancer of the bladder' OR 'bladder cancer' OR 'bladder cancers' OR 'cancer, bladder' OR 'cancer of bladder' | 84,865 |
|  | #26 #6 OR #7 OR #8 OR #9 OR #10 OR #11 OR #12 OR #13 OR #14 OR #15 OR #16 OR #17 OR #18 OR #19 OR #20 OR #21 OR #22 OR #23 OR #24 OR #25 | 999,838 |
|  | #27 #5 AND #26 AND [1966-2022]/py | 751 |
| PUBMED/MEDLINE | #1 "Periodontal Diseases"[MeSH Terms] | 92,675 |
|  | #2 "disease periodontal"[All Fields] OR "diseases periodontal"[All Fields] OR "periodontal disease"[All Fields] OR "Parodontosis"[All Fields] OR "Parodontoses"[All Fields] OR "pyorrhea alveolaris"[All Fields] | 21,082 |
|  | #3 "Furcation Defects"[MeSH Terms] OR "Gingivitis"[MeSH Terms] OR "peri-implantitis"[MeSH Terms] OR "Periodontitis"[MeSH Terms] OR "Alveolar Bone Loss"[MeSH Terms] OR "Periodontal Attachment Loss"[MeSH Terms] | 51,371 |
|  | #4 "defect furcation"[All Fields] OR "defects furcation"[All Fields] OR "Furcation Defect"[All Fields] OR "Gingivitides"[All Fields] OR "Peri Implantitis"[All Fields] OR "Peri-Implantitides"[All Fields] OR "Periimplantitis"[All Fields] OR "Periimplantitides"[All Fields] OR "Periodontitides"[All Fields] OR "Pericementitis"[All Fields] OR "Alveolar Bone Losses"[All Fields] OR "Alveolar Process Atrophy"[All Fields] OR "Alveolar Process Atrophies"[All Fields] OR "Alveolar Resorption"[All Fields] OR "resorption alveolar"[All Fields] OR "bone loss periodontal"[All Fields] OR "Periodontal Bone Losses"[All Fields] OR "Periodontal Bone Loss"[All Fields] OR "Periodontal Resorption"[All Fields] OR "resorption periodontal"[All Fields] OR "Alveolar Bone Atrophy"[All Fields] OR "bone loss alveolar"[All Fields] OR "attachment loss periodontal"[All Fields] OR "loss periodontal attachment"[All Fields] | 4,433 |
|  | #5 #1 OR #2 OR #3 OR #4 | 100,287 |
|  | #6 "Urogenital Neoplasms"[MeSH Terms] | 557,868 |
|  | #7 "Urogenital Neoplasm"[All Fields] OR "Neoplasm, Urogenital"[All Fields] OR "Neoplasms, Urogenital"[All Fields] OR "Genito-urinary Neoplasm"[All Fields] OR "Genito-urinary Neoplasms"[All Fields] OR "Neoplasm, Genito-urinary"[All Fields] OR "Neoplasms, Genito-urinary"[All Fields] OR "Genitourinary Neoplasms"[All Fields] OR "Genitourinary Neoplasm"[All Fields] OR "Neoplasm, Genitourinary"[All Fields] OR "Neoplasms, Genitourinary"[All Fields] OR "Genitourinary Cancer"[All Fields] OR "Cancer, Genitourinary"[All Fields] OR "Cancers, Genitourinary"[All Fields] OR "Genitourinary Cancers"[All Fields] OR "Genito-urinary Cancer"[All Fields] OR "Cancer, Genito-urinary"[All Fields] OR "Cancers, Genito-urinary"[All Fields] OR "Genito urinary Cancer"[All Fields] OR "Genito-urinary Cancers"[All Fields] OR "Urogenital Cancer"[All Fields] OR "Cancer, Urogenital"[All Fields] OR "Cancers, Urogenital"[All Fields] OR "Urogenital Cancers"[All Fields] | 1,789 |
|  | #8 "Genital Neoplasms, Female"[MeSH Terms] | 246,865 |
|  | #9 "Neoplasms, Female Genital"[All Fields] OR "Gynecologic Neoplasms"[All Fields] OR "Neoplasms, Gynecologic"[All Fields] OR "Gynecologic Neoplasm"[All Fields] OR "Neoplasm, Gynecologic"[All Fields] OR "Female Genital Neoplasms"[All Fields] OR "Female Genital Neoplasm"[All Fields] OR "Genital Neoplasm, Female"[All Fields] OR "Neoplasm, Female Genital"[All Fields] | 14,930 |
|  | #10 "Fallopian Tube Neoplasms"[MeSH Terms] | 3,066 |
|  | #11 "Fallopian Tube Neoplasm"[All Fields] OR "Neoplasm, Fallopian Tube"[All Fields] OR "Neoplasms, Fallopian Tube"[All Fields] OR "Fallopian Tube Cancer"[All Fields] OR "Cancer, Fallopian Tube"[All Fields] OR "Cancers, Fallopian Tube"[All Fields] OR "Fallopian Tube Cancers"[All Fields] OR "Cancer of the Fallopian Tube"[All Fields] | 555 |
|  | #12 "Uterine Neoplasms"[MeSH Terms] | 141,993 |
|  | #13 "Neoplasms, Uterus"[All Fields] OR "Neoplasm, Uterus"[All Fields] OR "Uterus Neoplasm"[All Fields] OR "Uterus Neoplasms"[All Fields] OR "Neoplasms, Uterine"[All Fields] OR "Neoplasm, Uterine"[All Fields] OR "Uterine Neoplasm"[All Fields] OR "Cancer of Uterus"[All Fields] OR "Uterus Cancers"[All Fields] OR "Cancer of the Uterus"[All Fields] OR "Uterus Cancer"[All Fields] OR "Cancer, Uterus"[All Fields] OR "Cancers, Uterus"[All Fields] OR "Uterine Cancer"[All Fields] OR "Cancer, Uterine"[All Fields] OR "Cancers, Uterine"[All Fields] OR "Uterine Cancers"[All Fields] | 7,668 |
|  | #14 "Vaginal Neoplasms"[MeSH Terms] | 5,432 |
|  | #15 "Neoplasm, Vaginal"[All Fields] OR "Vaginal Neoplasm"[All Fields] OR "Vagina Neoplasms"[All Fields] OR "Neoplasm, Vagina"[All Fields] OR "Neoplasms, Vagina"[All Fields] OR "Vagina Neoplasm"[All Fields] OR "Neoplasms, Vaginal"[All Fields] OR "Vaginal Cancer"[All Fields] OR "Cancer, Vaginal"[All Fields] OR "Cancers, Vaginal"[All Fields] OR "Vaginal Cancers"[All Fields] OR "Cancer of the Vagina"[All Fields] OR "Cancer of Vagina"[All Fields] OR "Vagina Cancers"[All Fields] OR "Vagina Cancer"[All Fields] OR "Cancer, Vagina"[All Fields] OR "Cancers, Vagina"[All Fields] | 1,385 |
|  | #16 "Vulvar Neoplasms"[MeSH Terms] | 8,646 |
|  | #17 "Neoplasm, Vulvar"[All Fields] OR "Vulvar Neoplasm"[All Fields] OR "Neoplasms, Vulvar"[All Fields] OR "Vulva Neoplasms"[All Fields] OR "Neoplasm, Vulva"[All Fields] OR "Neoplasms, Vulva"[All Fields] OR "Vulva Neoplasm"[All Fields] OR "Cancer of Vulva"[All Fields] OR "Vulva Cancers"[All Fields] OR "Cancer of the Vulva"[All Fields] OR "Vulva Cancer"[All Fields] OR "Cancer, Vulva"[All Fields] OR "Cancers, Vulva"[All Fields] OR "Vulvar Cancer"[All Fields] OR "Cancer, Vulvar"[All Fields] OR "Cancers, Vulvar"[All Fields] OR "Vulvar Cancers"[All Fields] | 2,744 |
|  | #18 "Genital Neoplasms, Male"[MeSH Terms] | 176,421 |
|  | #19 "Neoplasms, Male Genital"[All Fields] OR "Neoplasms, Male Genital"[All Fields] OR "Male Genital Neoplasms"[All Fields] OR "Genital Neoplasm, Male"[All Fields] OR "Male Genital Neoplasm"[All Fields] OR "Neoplasm, Male Genital"[All Fields] | 2,958 |
|  | #20 "Penile Neoplasms"[MeSH Terms] | 5,775 |
|  | #21 "Neoplasms, Penis"[All Fields] OR "Penis Neoplasms"[All Fields] OR "Neoplasm, Penis"[All Fields] OR "Penis Neoplasm"[All Fields] OR "Neoplasms, Penile"[All Fields] OR "Neoplasm, Penile"[All Fields] OR "Penile Neoplasm"[All Fields] OR "Cancer of Penis"[All Fields] OR "Penis Cancers"[All Fields] OR "Cancer of the Penis"[All Fields] OR "Penis Cancer"[All Fields] OR "Cancer, Penis"[All Fields] OR "Cancers, Penis"[All Fields] OR "Penile Cancer"[All Fields] OR "Penile Cancer"[All Fields] OR "Cancer, Penile"[All Fields] OR "Cancers, Penile"[All Fields] OR "Penile Cancers"[All Fields] | 2,545 |
|  | #22 "Prostatic Neoplasms"[MeSH Terms] | 142,245 |
|  | #23 "Prostate Neoplasms"[All Fields] OR "Neoplasms, Prostate"[All Fields] OR "Neoplasm, Prostate"[All Fields] OR "Neoplasm, Prostate"[All Fields] OR "Prostate Neoplasm"[All Fields] OR "Neoplasms, Prostatic"[All Fields] OR "Neoplasm, Prostatic"[All Fields] OR "Prostatic Neoplasm"[All Fields] OR "Prostate Cancer"[All Fields] OR "Cancer, Prostate"[All Fields] OR "Cancers, Prostate"[All Fields] OR "Prostate Cancers"[All Fields] OR "Cancer of the Prostate"[All Fields] OR "Prostatic Cancer"[All Fields] OR "Cancer, Prostatic"[All Fields] OR "Cancers, Prostatic"[All Fields] OR "Prostatic Cancers"[All Fields] OR "Cancer of Prostate"[All Fields] | 143,665 |
|  | #24 "Testicular Neoplasms"[MeSH Terms] | 27,070 |
|  | #25 "Testicular Neoplasm"[All Fields] OR "Neoplasm, Testicular"[All Fields] OR "Testicular Tumors"[All Fields] OR "Neoplasms, Testis"[All Fields] OR "Neoplasm, Testis"[All Fields] OR "Testis Neoplasm"[All Fields] OR "Testis Neoplasms"[All Fields] OR "Testicular Tumor"[All Fields] OR "Tumor, Testicular"[All Fields] OR "Tumors, Testicular"[All Fields] OR "Neoplasms, Testicular"[All Fields] OR "Tumor of Rete Testis"[All Fields] OR "Rete Testis Tumor"[All Fields] OR "Rete Testis Tumors"[All Fields] OR "Testis Tumor, Rete"[All Fields] OR "Testis Tumors, Rete"[All Fields] OR "Cancer of Testis"[All Fields] OR "Testis Cancer"[All Fields] OR "Cancer, Testis"[All Fields] OR "Cancers, Testis"[All Fields] OR "Testis Cancers"[All Fields] OR "Cancer of the Testes"[All Fields] OR "Cancer of the Testis"[All Fields] OR "Testicular Cancer"[All Fields] OR "Cancer, Testicular"[All Fields] OR "Cancers, Testicular"[All Fields] OR "Testicular Cancers"[All Fields] | 13,568 |
|  | #26 "Urologic Neoplasms"[MeSH Terms] | 146,543 |
|  | #27 "Urological Neoplasms"[All Fields] OR "Neoplasm, Urological"[All Fields] OR "Neoplasms, Urological"[All Fields] OR "Urological Neoplasm"[All Fields] OR "Urinary Tract Neoplasms"[All Fields] OR "Neoplasm, Urinary Tract"[All Fields] OR "Neoplasms, Urinary Tract"[All Fields] OR "Tract Neoplasm, Urinary"[All Fields] OR "Tract Neoplasms, Urinary"[All Fields] OR "Urinary Tract Neoplasm"[All Fields] OR "Neoplasms, Urologic"[All Fields] OR "Neoplasm, Urologic"[All Fields] OR "Urologic Neoplasm"[All Fields] OR "Cancer of Urinary Tract"[All Fields] OR "Urinary Tract Cancers"[All Fields] OR "Urological Cancer"[All Fields] OR "Cancer, Urological"[All Fields] OR "Cancers, Urological"[All Fields] OR "Urological Cancers"[All Fields] OR "Cancer of the Urinary Tract"[All Fields] OR "Urinary Tract Cancer"[All Fields] OR "Cancer, Urinary Tract"[All Fields] OR "Cancers, Urinary Tract"[All Fields] OR "Urologic Cancer"[All Fields] OR "Cancer, Urologic"[All Fields] OR "Cancers, Urologic"[All Fields] OR "Urologic Cancers"[All Fields] | 3,897 |
|  | #28 "Kidney Neoplasms"[MeSH Terms] | 80,930 |
|  | #29 "Kidney Neoplasm"[All Fields] OR "Neoplasm, Kidney"[All Fields] OR "Renal Neoplasms"[All Fields] OR "Neoplasm, Renal"[All Fields] OR "Neoplasms, Renal"[All Fields] OR "Renal Neoplasm"[All Fields] OR "Neoplasms, Kidney"[All Fields] OR "Cancer of Kidney"[All Fields] OR "Kidney Cancers"[All Fields] OR "Renal Cancer"[All Fields] OR "Cancer, Renal"[All Fields] OR "Cancers, Renal"[All Fields] OR "Renal Cancers"[All Fields] OR "Cancer of the Kidney"[All Fields] OR "Kidney Cancer"[All Fields] OR "Cancer, Kidney"[All Fields] | 16,399 |
|  | #30 "Ureteral Neoplasms"[MeSH Terms] | 4,971 |
|  | #31 "Neoplasm, Ureteral"[All Fields] OR "Ureteral Neoplasm"[All Fields] OR "Neoplasms, Ureteral"[All Fields] OR "Ureter Neoplasms"[All Fields] OR "Ureter Neoplasm"[All Fields] OR "Neoplasms of Ureter"[All Fields] OR "Cancer of Ureter"[All Fields] OR "Ureter Cancers"[All Fields] OR "Ureter, Cancer Of"[All Fields] OR "Ureter Cancer"[All Fields] OR "Ureteral Cancer"[All Fields] OR "Cancer, Ureteral"[All Fields] OR "Cancers, Ureteral"[All Fields] OR "Ureteral Cancers"[All Fields] OR "Cancer of the Ureter"[All Fields] | 508 |
|  | #32 "Urethral Neoplasms"[MeSH Terms] | 2,620 |
|  | #33 "Neoplasms, Urethral"[All Fields] OR "Neoplasm, Urethral"[All Fields] OR "Urethral Neoplasm"[All Fields] OR "Urethra Neoplasms"[All Fields] OR "Neoplasm, Urethra"[All Fields] OR "Neoplasms, Urethra"[All Fields] OR "Urethra Neoplasm"[All Fields] OR "Cancer of Urethra"[All Fields] OR "Urethra Cancers"[All Fields] OR "Urethra Cancer"[All Fields] OR "Cancer, Urethra"[All Fields] OR "Cancers, Urethra"[All Fields] OR "Urethral Cancer"[All Fields] OR "Cancer, Urethral"[All Fields] OR "Cancers, Urethral"[All Fields] OR "Urethral Cancers"[All Fields] OR "Cancer of the Urethra"[All Fields] | 484 |
|  | #34 "Urinary Bladder Neoplasms"[MeSH Terms] | 59,610 |
|  | #35 "Neoplasms, Urethral"[All Fields] OR "Urinary Bladder Neoplasm"[All Fields] OR "Neoplasms, Bladder"[All Fields] OR "Bladder Neoplasms"[All Fields] OR "Bladder Neoplasm"[All Fields] OR "Neoplasm, Bladder"[All Fields] OR "Bladder Tumors"[All Fields] OR "Bladder Tumor"[All Fields] OR "Tumor, Bladder"[All Fields] OR "Tumors, Bladder"[All Fields] OR "Urinary Bladder Cancer"[All Fields] OR "Cancer, Urinary Bladder"[All Fields] OR "Malignant Tumor of Urinary Bladder"[All Fields] OR "Cancer of the Bladder"[All Fields] OR "Bladder Cancer"[All Fields] OR "Bladder Cancers"[All Fields] OR "Cancer, Bladder"[All Fields] OR "Cancer of Bladder"[All Fields] | 72,267 |
|  | #36 #6 OR #7 OR #8 OR #9 OR #10 OR #11 OR #12 OR #13 OR #14 OR #15 OR #16 OR #17 OR #18 OR #19 OR #20 OR #21 OR #22 OR #23 OR #24 OR #25 OR #26 OR #27 OR #28 OR #29 OR #30 OR #31 OR #32 OR #33 OR #34 OR #35 | 616,332 |
|  | #37 #5 AND #36 | 157 |
|  | #38 "1966/01/01"[Date - Publication] : "2021/03/31"[Date - Publication] | 31,609,223 |
|  | #39 #37 AND #38 | 152 |
| Cochrane Library | #1 MeSH descriptor: [Periodontal Diseases] explode all trees | 7,091 |
|  | #2 MeSH descriptor: [Furcation Defects] explode all trees | 181 |
|  | #3 MeSH descriptor: [Gingivitis] explode all trees | 1,495 |
|  | #4 MeSH descriptor: [Peri-Implantitis] explode all trees | 226 |
|  | #5 MeSH descriptor: [Periodontitis] explode all trees | 3,169 |
|  | #6 MeSH descriptor: [Periodontal Attachment Loss] explode all trees | 883 |
|  | #7 (Disease, Periodontal) OR (Diseases, Periodontal) OR (Periodontal Disease) OR (Parodontosis) OR (Parodontoses) OR (Pyorrhea Alveolaris) OR (defect furcation) OR (defects furcation) OR (Furcation Defect) OR (Gingivitides) OR (Peri Implantitis) OR (Peri-Implantitides) OR (Periimplantitis) OR (Periimplantitides) OR (Periodontitides) OR (Pericementitis) OR (Alveolar Bone Losses) OR (Alveolar Process Atrophy) OR (Alveolar Process Atrophies) OR (Alveolar Resorption) OR (resorption alveolar) OR (bone loss periodontal) OR (Periodontal Bone Losses) OR (Periodontal Bone Loss) OR (Periodontal Resorption) OR (resorption periodontal) OR (Alveolar Bone Atrophy) OR (bone loss alveolar) OR (attachment loss periodontal) OR (loss periodontal attachment) | 7,679 |
|  | #8 #1 OR #2 OR #3 OR #4 OR #5 OR #6 OR #7 | 10,925 |
|  | #9 MeSH descriptor: [Urogenital Neoplasms] explode all trees | 13,624 |
|  | #10 (Urogenital Neoplasm) OR (Neoplasm, Urogenital) OR (Neoplasms, Urogenital) OR (Genito-urinary Neoplasm) OR (Genito-urinary Neoplasms) OR (Neoplasm, Genito-urinary) OR (Neoplasms, Genito-urinary) OR (Genitourinary Neoplasms) OR (Genitourinary Neoplasm) OR (Neoplasm, Genitourinary) OR (Neoplasms, Genitourinary) OR (Genitourinary Cancer) OR (Cancer, Genitourinary) OR (Cancers, Genitourinary) OR (Genitourinary Cancers) OR (Genito-urinary Cancer) OR (Cancer, Genito-urinary) OR (Cancers, Genito-urinary) OR (Genito urinary Cancer) OR (Genito-urinary Cancers) OR (Urogenital Cancer) OR (Cancer, Urogenital) OR (Cancers, Urogenital) OR (Urogenital Cancers) | 2,203 |
|  | #11 MeSH descriptor: [Genital Neoplasms, Female] explode all trees | 4,415 |
|  | #12 (Neoplasms, Female Genital) OR (Gynecologic Neoplasms) OR (Neoplasms, Gynecologic) OR (Gynecologic Neoplasm) OR (Neoplasm, Gynecologic) OR (Female Genital Neoplasms) OR (Female Genital Neoplasm) OR (Genital Neoplasm, Female) OR (Neoplasm, Female Genital) | 2,081 |
|  | #13 MeSH descriptor: [Fallopian Tube Neoplasms] explode all trees | 268 |
|  | #14 (Fallopian Tube Neoplasm) OR (Neoplasm, Fallopian Tube) OR (Neoplasms, Fallopian Tube) OR (Fallopian Tube Cancer) OR (Cancer, Fallopian Tube) OR (Cancers, Fallopian Tube) OR (Fallopian Tube Cancers) OR (Cancer of the Fallopian Tube) | 1,047 |
|  | #15 MeSH descriptor: [Uterine Neoplasms] explode all trees | 3,617 |
|  | #16 (Neoplasms, Uterus) OR (Neoplasm, Uterus) OR (Uterus Neoplasm) OR (Uterus Neoplasms) OR (Neoplasms, Uterine) OR (Neoplasm, Uterine) OR (Uterine Neoplasm) OR (Cancer of Uterus) OR (Uterus Cancers) OR (Cancer of the Uterus) OR (Uterus Cancer) OR (Cancer, Uterus) OR (Cancers, Uterus) OR (Uterine Cancer) OR (Cancer, Uterine) OR (Cancers, Uterine) OR (Uterine Cancers) | 6,667 |
|  | #17 MeSH descriptor: [Vaginal Neoplasms] explode all trees | 57 |
|  | #18 (Neoplasm, Vaginal) OR (Vaginal Neoplasm) OR (Vagina Neoplasms) OR (Neoplasm, Vagina) OR (Neoplasms, Vagina) OR (Vagina Neoplasm) OR (Neoplasms, Vaginal) OR (Vaginal Cancer) OR (Cancer, Vaginal) OR (Cancers, Vaginal) OR (Vaginal Cancers) OR (Cancer of the Vagina) OR (Cancer of Vagina) OR (Vagina Cancers) OR (Vagina Cancer) OR (Cancer, Vagina) OR (Cancers, Vagina) | 2,466 |
|  | #19 MeSH descriptor: [Vulvar Neoplasms] explode all trees | 107 |
|  | #20 (Neoplasm, Vulvar) OR (Vulvar Neoplasm) OR (Neoplasms, Vulvar) OR (Vulva Neoplasms) OR (Neoplasm, Vulva) OR (Neoplasms, Vulva) OR (Vulva Neoplasm) OR (Cancer of Vulva) OR (Vulva Cancers) OR (Cancer of the Vulva) OR (Vulva Cancer) OR (Cancer, Vulva) OR (Cancers, Vulva) OR (Vulvar Cancer) OR (Cancer, Vulvar) OR (Cancers, Vulvar) OR (Vulvar Cancers) | 410 |
|  | #21 MeSH descriptor: [Genital Neoplasms, Male] explode all trees | 6,413 |
|  | #22 (Neoplasms, Male Genital) OR (Neoplasms, Male Genital) OR (Male Genital Neoplasms) OR (Genital Neoplasm, Male) OR (Male Genital Neoplasm) OR (Neoplasm, Male Genital) | 213 |
|  | #23 MeSH descriptor: [Penile Neoplasms] explode all trees | 27 |
|  | #24 (Neoplasms, Penis) OR (Penis Neoplasms) OR (Neoplasm, Penis) OR (Penis Neoplasm) OR (Neoplasms, Penile) OR (Neoplasm, Penile) OR (Penile Neoplasm) OR (Cancer of Penis) OR (Penis Cancers) OR (Cancer of the Penis) OR (Penis Cancer) OR (Cancer, Penis) OR (Cancers, Penis) OR (Penile Cancer) OR (Penile Cancer) OR (Cancer, Penile) OR (Cancers, Penile) OR (Penile Cancers) | 472 |
|  | #25 MeSH descriptor: [Prostatic Neoplasms] explode all trees | 6,115 |
|  | #26 (Prostate Neoplasms) OR (Neoplasms, Prostate) OR (Neoplasm, Prostate) OR (Neoplasm, Prostate) OR (Prostate Neoplasm) OR (Neoplasms, Prostatic) OR (Neoplasm, Prostatic) OR (Prostatic Neoplasm) OR (Prostate Cancer) OR (Cancer, Prostate) OR (Cancers, Prostate) OR (Prostate Cancers) OR (Cancer of the Prostate) OR (Prostatic Cancer) OR (Cancer, Prostatic) OR (Cancers, Prostatic) OR (Prostatic Cancers) OR (Cancer of Prostate) | 17,561 |
|  | #27 MeSH descriptor: [Testicular Neoplasms] explode all trees | 250 |
|  | #28 (Testicular Neoplasm) OR (Neoplasm, Testicular) OR (Testicular Tumors) OR (Neoplasms, Testis) OR (Neoplasm, Testis) OR (Testis Neoplasm) OR (Testis Neoplasms) OR (Testicular Tumor) OR (Tumor, Testicular) OR (Tumors, Testicular) OR (Neoplasms, Testicular) OR (Tumor of Rete Testis) OR (Rete Testis Tumor) OR (Rete Testis Tumors) OR (Testis Tumor, Rete) OR (Testis Tumors, Rete) OR (Cancer of Testis) OR (Testis Cancer) OR (Cancer, Testis) OR (Cancers, Testis) OR (Testis Cancers) OR (Cancer of the Testes) OR (Cancer of the Testis) OR (Testicular Cancer) OR (Cancer, Testicular) OR (Cancers, Testicular) OR (Testicular Cancers) | 2,783 |
|  | #29 MeSH descriptor: [Urologic Neoplasms] explode all trees | 2,954 |
|  | #30 (Urological Neoplasms) OR (Neoplasm, Urological) OR (Neoplasms, Urological) OR (Urological Neoplasm) OR (Urinary Tract Neoplasms) OR (Neoplasm, Urinary Tract) OR (Neoplasms, Urinary Tract) OR (Tract Neoplasm, Urinary) OR (Tract Neoplasms, Urinary) OR (Urinary Tract Neoplasm) OR (Neoplasms, Urologic) OR (Neoplasm, Urologic) OR (Urologic Neoplasm) OR (Cancer of Urinary Tract) OR (Urinary Tract Cancers) OR (Urological Cancer) OR (Cancer, Urological) OR (Cancers, Urological) OR (Urological Cancers) OR (Cancer of the Urinary Tract) OR (Urinary Tract Cancer) OR (Cancer, Urinary Tract) OR (Cancers, Urinary Tract) OR (Urologic Cancer) OR (Cancer, Urologic) OR (Cancers, Urologic) OR (Urologic Cancers) | 4,286 |
|  | #31 MeSH descriptor: [Kidney Neoplasms] explode all trees | 1298 |
|  | #32 (Kidney Neoplasm) OR (Neoplasm, Kidney) OR (Renal Neoplasms) OR (Neoplasm, Renal) OR (Neoplasms, Renal) OR (Renal Neoplasm) OR (Neoplasms, Kidney) OR (Cancer of Kidney) OR (Kidney Cancers) OR (Renal Cancer) OR (Cancer, Renal) OR (Cancers, Renal) OR (Renal Cancers) OR (Cancer of the Kidney) OR (Kidney Cancer) OR (Cancer, Kidney) | 12408 |
|  | #33 MeSH descriptor: [Ureteral Neoplasms] explode all trees | 27 |
|  | #34 (Neoplasm, Ureteral) OR (Ureteral Neoplasm) OR (Neoplasms, Ureteral) OR (Ureter Neoplasms) OR (Ureter Neoplasm) OR (Neoplasms of Ureter) OR (Cancer of Ureter) OR (Ureter Cancers) OR (Ureter, Cancer Of) OR (Ureter Cancer) OR (Ureteral Cancer) OR (Cancer, Ureteral) OR (Cancers, Ureteral) OR (Ureteral Cancers) OR (Cancer of the Ureter) | 383 |
|  | #35 MeSH descriptor: [Urethral Neoplasms] explode all trees | 16 |
|  | #36 (Neoplasms, Urethral) OR (Neoplasm, Urethral) OR (Urethral Neoplasm) OR (Urethra Neoplasms) OR (Neoplasm, Urethra) OR (Neoplasms, Urethra) OR (Urethra Neoplasm) OR (Cancer of Urethra) OR (Urethra Cancers) OR (Urethra Cancer) OR (Cancer, Urethra) OR (Cancers, Urethra) OR (Urethral Cancer) OR (Cancer, Urethral) OR (Cancers, Urethral) OR (Urethral Cancers) OR (Cancer of the Urethra) | 765 |
|  | #37 MeSH descriptor: [Urinary Bladder Neoplasms] explode all trees | 1,609 |
|  | #38 (Neoplasms, Urethral) OR (Urinary Bladder Neoplasm) OR (Neoplasms, Bladder) OR (Bladder Neoplasms) OR (Bladder Neoplasm) OR (Neoplasm, Bladder) OR (Bladder Tumors) OR (Bladder Tumor) OR (Tumor, Bladder) OR (Tumors, Bladder) OR (Urinary Bladder Cancer) OR (Cancer, Urinary Bladder) OR (Malignant Tumor of Urinary Bladder) OR (Cancer of the Bladder) OR (Bladder Cancer) OR (Bladder Cancers) OR (Cancer, Bladder) OR (Cancer of Bladder) | 6,158 |
|  | #39 #9 OR #10 OR #11 OR #12 OR #13 OR #14 OR #15 OR #16 OR #17 OR #18 OR #19 OR #20 OR #21 OR #22 OR #23 OR #24 OR #25 OR #26 OR #27 OR #28 OR #29 OR #30 OR #31 OR #32 OR #33 OR #34 OR #35 OR #36 OR #37 OR #38 | 44,420 |
|  | #40 #8 AND #39 with Cochrane Library publication date Between Jan 1966 and Mar 2021 | 50 |
| Web of Science | #1 TS=('Periodontal Diseases' OR 'disease periodontal' OR 'diseases periodontal' OR 'periodontal disease' OR 'Parodontosis' OR 'Parodontoses' OR 'pyorrhea alveolaris') | 33,127 |
|  | #2 TS=('Furcation Defects' OR 'Gingivitis' OR 'peri-implantitis' OR 'Periodontitis' OR 'Alveolar Bone Loss' OR 'Periodontal Attachment Loss' OR 'defect furcation' OR 'defects furcation' OR 'Furcation Defect' OR 'Gingivitides' OR 'Peri Implantitis' OR 'Peri-Implantitides' OR 'Periimplantitis' OR 'Periimplantitides' OR 'Periodontitides' OR 'Pericementitis' OR 'Alveolar Bone Losses' OR 'Alveolar Process Atrophy' OR 'Alveolar Process Atrophies' OR 'Alveolar Resorption' OR 'resorption alveolar' OR 'bone loss periodontal' OR 'Periodontal Bone Losses' OR 'Periodontal Bone Loss' OR 'Periodontal Resorption' OR 'resorption periodontal' OR 'Alveolar Bone Atrophy' OR 'bone loss alveolar' OR 'attachment loss periodontal' OR 'loss periodontal attachment') | 50,695 |
|  | #3 #1 OR #2 | 65,085 |
|  | #4 TS=('Urogenital Neoplasms' OR 'Urogenital Neoplasm' OR 'Neoplasm, Urogenital' OR 'Neoplasms, Urogenital' OR 'Genito-urinary Neoplasm' OR 'Genito-urinary Neoplasms' OR 'Neoplasm, Genito-urinary' OR 'Neoplasms, Genito-urinary' OR 'Genitourinary Neoplasms' OR 'Genitourinary Neoplasm' OR 'Neoplasm, Genitourinary' OR 'Neoplasms, Genitourinary' OR 'Genitourinary Cancer' OR 'Cancer, Genitourinary' OR 'Cancers, Genitourinary' OR 'Genitourinary Cancers' OR 'Genito-urinary Cancer' OR 'Cancer, Genito-urinary' OR 'Cancers, Genito-urinary' OR 'Genito urinary Cancer' OR 'Genito-urinary Cancers' OR 'Urogenital Cancer' OR 'Cancer, Urogenital' OR 'Cancers, Urogenital' OR 'Urogenital Cancers') | 7,868 |
|  | #5 TS=('Genital Neoplasms, Female' OR 'Neoplasms, Female Genital' OR 'Gynecologic Neoplasms' OR 'Neoplasms, Gynecologic' OR 'Gynecologic Neoplasm' OR 'Neoplasm, Gynecologic' OR 'Female Genital Neoplasms' OR 'Female Genital Neoplasm' OR 'Genital Neoplasm, Female' OR 'Neoplasm, Female Genital') | 2,571 |
|  | #6 TS=('Fallopian Tube Neoplasms' OR 'Fallopian Tube Neoplasm' OR 'Neoplasm, Fallopian Tube' OR 'Neoplasms, Fallopian Tube' OR 'Fallopian Tube Cancer' OR 'Cancer, Fallopian Tube' OR 'Cancers, Fallopian Tube' OR 'Fallopian Tube Cancers' OR 'Cancer of the Fallopian Tube') | 4,428 |
|  | #7 TS=('Uterine Neoplasms' OR 'Neoplasms, Uterus' OR 'Neoplasm, Uterus' OR 'Uterus Neoplasm' OR 'Uterus Neoplasms' OR 'Neoplasms, Uterine' OR 'Neoplasm, Uterine' OR 'Uterine Neoplasm' OR 'Cancer of Uterus' OR 'Uterus Cancers' OR 'Cancer of the Uterus' OR 'Uterus Cancer' OR 'Cancer, Uterus' OR 'Cancers, Uterus' OR 'Uterine Cancer' OR 'Cancer, Uterine' OR 'Cancers, Uterine' OR 'Uterine Cancers') | 34,101 |
|  | #8 TS=('Vaginal Neoplasms' OR 'Neoplasm, Vaginal' OR 'Vaginal Neoplasm' OR 'Vagina Neoplasms' OR 'Neoplasm, Vagina' OR 'Neoplasms, Vagina' OR 'Vagina Neoplasm' OR 'Neoplasms, Vaginal' OR 'Vaginal Cancer' OR 'Cancer, Vaginal' OR 'Cancers, Vaginal' OR 'Vaginal Cancers' OR 'Cancer of the Vagina' OR 'Cancer of Vagina' OR 'Vagina Cancers' OR 'Vagina Cancer' OR 'Cancer, Vagina' OR 'Cancers, Vagina') | 12,469 |
|  | #9 TS=('Vulvar Neoplasms' OR 'Neoplasm, Vulvar' OR 'Vulvar Neoplasm' OR 'Neoplasms, Vulvar' OR 'Vulva Neoplasms' OR 'Neoplasm, Vulva' OR 'Neoplasms, Vulva' OR 'Vulva Neoplasm' OR 'Cancer of Vulva' OR 'Vulva Cancers' OR 'Cancer of the Vulva' OR 'Vulva Cancer' OR 'Cancer, Vulva' OR 'Cancers, Vulva' OR 'Vulvar Cancer' OR 'Cancer, Vulvar' OR 'Cancers, Vulvar' OR 'Vulvar Cancers') | 5,983 |
|  | #10 TS=('Genital Neoplasms, Male' OR 'Neoplasms, Male Genital' OR 'Neoplasms, Male Genital' OR 'Male Genital Neoplasms' OR 'Genital Neoplasm, Male' OR 'Male Genital Neoplasm' OR 'Neoplasm, Male Genital') | 221 |
|  | #11 TS=('Penile Neoplasms' OR 'Neoplasms, Penis' OR 'Penis Neoplasms' OR 'Neoplasm, Penis' OR 'Penis Neoplasm' OR 'Neoplasms, Penile' OR 'Neoplasm, Penile' OR 'Penile Neoplasm' OR 'Cancer of Penis' OR 'Penis Cancers' OR 'Cancer of the Penis' OR 'Penis Cancer' OR 'Cancer, Penis' OR 'Cancers, Penis' OR 'Penile Cancer' OR 'Penile Cancer' OR 'Cancer, Penile' OR 'Cancers, Penile' OR 'Penile Cancers') | 5,436 |
|  | #12 TS=('Prostatic Neoplasms' OR 'Prostate Neoplasms' OR 'Neoplasms, Prostate' OR 'Neoplasm, Prostate' OR 'Neoplasm, Prostate' OR 'Prostate Neoplasm' OR 'Neoplasms, Prostatic' OR 'Neoplasm, Prostatic' OR 'Prostatic Neoplasm' OR 'Prostate Cancer' OR 'Cancer, Prostate' OR 'Cancers, Prostate' OR 'Prostate Cancers' OR 'Cancer of the Prostate' OR 'Prostatic Cancer' OR 'Cancer, Prostatic' OR 'Cancers, Prostatic' OR 'Prostatic Cancers' OR 'Cancer of Prostate') | 255,101 |
|  | #13 TS=('Testicular Neoplasms' OR 'Testicular Neoplasm' OR 'Neoplasm, Testicular' OR 'Testicular Tumors' OR 'Neoplasms, Testis' OR 'Neoplasm, Testis' OR 'Testis Neoplasm' OR 'Testis Neoplasms' OR 'Testicular Tumor' OR 'Tumor, Testicular' OR 'Tumors, Testicular' OR 'Neoplasms, Testicular' OR 'Tumor of Rete Testis' OR 'Rete Testis Tumor' OR 'Rete Testis Tumors' OR 'Testis Tumor, Rete' OR 'Testis Tumors, Rete' OR 'Cancer of Testis' OR 'Testis Cancer' OR 'Cancer, Testis' OR 'Cancers, Testis' OR 'Testis Cancers' OR 'Cancer of the Testes' OR 'Cancer of the Testis' OR 'Testicular Cancer' OR 'Cancer, Testicular' OR 'Cancers, Testicular' OR 'Testicular Cancers') | 30,054 |
|  | #14 TS=('Urologic Neoplasms' OR 'Urological Neoplasms' OR 'Neoplasm, Urological' OR 'Neoplasms, Urological' OR 'Urological Neoplasm' OR 'Urinary Tract Neoplasms' OR 'Neoplasm, Urinary Tract' OR 'Neoplasms, Urinary Tract' OR 'Tract Neoplasm, Urinary' OR 'Tract Neoplasms, Urinary' OR 'Urinary Tract Neoplasm' OR 'Neoplasms, Urologic' OR 'Neoplasm, Urologic' OR 'Urologic Neoplasm' OR 'Cancer of Urinary Tract' OR 'Urinary Tract Cancers' OR 'Urological Cancer' OR 'Cancer, Urological' OR 'Cancers, Urological' OR 'Urological Cancers' OR 'Cancer of the Urinary Tract' OR 'Urinary Tract Cancer' OR 'Cancer, Urinary Tract' OR 'Cancers, Urinary Tract' OR 'Urologic Cancer' OR 'Cancer, Urologic' OR 'Cancers, Urologic' OR 'Urologic Cancers') | 18,745 |
|  | #15 TS=('Kidney Neoplasms' OR 'Kidney Neoplasm' OR 'Neoplasm, Kidney' OR 'Renal Neoplasms' OR 'Neoplasm, Renal' OR 'Neoplasms, Renal' OR 'Renal Neoplasm' OR 'Neoplasms, Kidney' OR 'Cancer of Kidney' OR 'Kidney Cancers' OR 'Renal Cancer' OR 'Cancer, Renal' OR 'Cancers, Renal' OR 'Renal Cancers' OR 'Cancer of the Kidney' OR 'Kidney Cancer' OR 'Cancer, Kidney') | 97,175 |
|  | #16 TS=('Ureteral Neoplasms' OR 'Neoplasm, Ureteral' OR 'Ureteral Neoplasm' OR 'Neoplasms, Ureteral' OR 'Ureter Neoplasms' OR 'Ureter Neoplasm' OR 'Neoplasms of Ureter' OR 'Cancer of Ureter' OR 'Ureter Cancers' OR 'Ureter, Cancer Of' OR 'Ureter Cancer' OR 'Ureteral Cancer' OR 'Cancer, Ureteral' OR 'Cancers, Ureteral' OR 'Ureteral Cancers' OR 'Cancer of the Ureter') | 4,042 |
|  | #17 TS=('Urethral Neoplasms' OR 'Neoplasms, Urethral' OR 'Neoplasm, Urethral' OR 'Urethral Neoplasm' OR 'Urethra Neoplasms' OR 'Neoplasm, Urethra' OR 'Neoplasms, Urethra' OR 'Urethra Neoplasm' OR 'Cancer of Urethra' OR 'Urethra Cancers' OR 'Urethra Cancer' OR 'Cancer, Urethra' OR 'Cancers, Urethra' OR 'Urethral Cancer' OR 'Cancer, Urethral' OR 'Cancers, Urethral' OR 'Urethral Cancers' OR 'Cancer of the Urethra') | 4,584 |
|  | #18 TS=('Urinary Bladder Neoplasms' OR 'Neoplasms, Urethral' OR 'Urinary Bladder Neoplasm' OR 'Neoplasms, Bladder' OR 'Bladder Neoplasms' OR 'Bladder Neoplasm' OR 'Neoplasm, Bladder' OR 'Bladder Tumors' OR 'Bladder Tumor' OR 'Tumor, Bladder' OR 'Tumors, Bladder' OR 'Urinary Bladder Cancer' OR 'Cancer, Urinary Bladder' OR 'Malignant Tumor of Urinary Bladder' OR 'Cancer of the Bladder' OR 'Bladder Cancer' OR 'Bladder Cancers' OR 'Cancer, Bladder' OR 'Cancer of Bladder') | 85,866 |
|  | #19 #4 OR #5 OR #6 OR #7 OR #8 OR #9 OR #10 OR #11 OR #12 OR #13 OR #14 OR #15 OR #16 OR #17 OR #18 | 491,559 |
|  | #20 #3 AND #19 (Indexes=SCI-EXPANDED Timespan=1966-2021) | 215 |
