# Supplementary material for: Is periodontal disease a risk indicator for urogenital cancer? A systematic review and meta-analysis of cohort studies
Source: Front Oncol. 2022 Aug 9;12:697399. doi: 10.3389/fonc.2022.697399 (PMC9395701; doi:10.3389/fonc.2022.697399)
Supplement: Supplementary file 2 [file Table_2.docx]

| **Appendix Table 2. Records excluded after reading topic and abstract with reasons** | |
| --- | --- |
| **Reasons** | No. of  studies |
| Animal studies | 32 |
| Bacteria studies | 51 |
| cancer therapy studies | 107 |
| Case report | 86 |
| Other disease therapy | 142 |
| Drug efficacy and route of administration | 77 |
| Cancer metastasis | 107 |
| No direct relationship between PD and UC | 32 |
| No mention of PD | 81 |
| No mention of UC | 89 |
| Osteonecrosis | 56 |
| Other disease | 62 |
| The subject of studies is tobacco /betel | 11 |
| The subject of studies is transplantation | 47 |
| Other studies no mention of PD and UC | 113 |
| **Total** | 1,093 |
