# Supplementary material for: Is periodontal disease a risk indicator for urogenital cancer? A systematic review and meta-analysis of cohort studies
Source: Front Oncol. 2022 Aug 9;12:697399. doi: 10.3389/fonc.2022.697399 (PMC9395701; doi:10.3389/fonc.2022.697399)
Supplement: Supplementary file 3 [file Table_3.docx]

| **Appendix Table 3. Records excluded after reading full-text with reasons** | | |
| --- | --- | --- |
| **48 records excluded after title and abstract review** | | |
| No. | Study ID | Characteristics |
| **Reason for exclusion: Intervention (exposures) was not PD patients (4 records excluded)** | | |
| 1 | (Hwang, Sun, Lin, Lee, & Kao, 2014) | Intervention: Routine treatment of periodontal disease |
| 2 | (Famili, Cauley, & Greenspan, 2007) | Intervention: Effect of androgen deprivation therapy |
| 3 | (da Silva, Alluri, Bissada, & Gupta, 2019) | Intervention: Oral pathogens |
| 4 | (Alluri et al., 2021) | Intervention: Pathogenic bacteria |
| **Reason for exclusion: Control was not adults without a history of PD (3 record excluded)** | | |
| 1 | (Dizdar et al., 2017) | Control: No control. |
| 2 | (Deniz Can Guven et al., 2018) | Control: No control. |
| 3 | (D. C. Guven et al., 2019) | Control: No control. |
| **Reason for exclusion: Outcome was not UC incidence (6 records excluded)** | | |
| 1 | (Yoshihara, Deguchi, Hanada, & Miyazaki, 2007) | Outcome: Renal function. |
| 2 | (Thorman, Neovius, & Hylander, 2009a) | Outcome: Chronic kidney disease. |
| 3 | (Grant, 2012) | Outcome: Chronic kidney disease. |
| 4 | (Liu et al., 2013) | Outcome: Chronic kidney disease. |
| 5 | (Joshy, Arora, Korda, Chalmers, & Banks, 2016) | Outcome: Cardiovascular disease |
| 6 | (Ruokonen et al., 2017) | Outcome: Cardiovascular disease |
| **Reason for exclusion: Study design was not cohort study (22 records excluded)** | | |
| 1 | (Fitzgerald, McInnes, & Manry, 1982) | Type of article: Case report. |
| 2 | (Shepherd & Lyon, 1990) | Type of article: Case report. |
| 3 | (Kshirsagar et al., 2002) | Type of article: Meeting Abstract. |
| 4 | (Migliorati, 2008) | Type of article: Comment. |
| 5 | (Fitzpatrick & Katz, 2010) | Type of article: Review |
| 6 | (Linden, Herzberg, & Working group 4 of joint, 2013) | Type of article: Review |
| 7 | (Linden, Lyons, & Scannapieco, 2013) | Type of article: Review |
| 8 | (Ruospo et al., 2014) | Type of article: System Review |
| 9 | (Corbella et al., 2018) | Type of article: System Review |
| 10 | (Shi et al., 2018) | Type of article: Meta-analysis |
| 11 | (Xie et al., 2018) | Type of article: Meta-analysis |
| 12 | (Zhao et al., 2018) | Type of article: System Review and Meta-analysis |
| 13 | (Jagodzinski et al., 2020) | Type of article: Review |
| 14 | (Ma, Zheng, & Li, 2020) | Type of article: Meta-analysis |
| 15 | (Schmalz & Ziebolz, 2020) | Type of article: Review |
| 16 | (Wei, Zhong, Wang, & Huang, 2020) | Type of article: System Review and Meta-analysis |
| 17 | (Guo et al., 2021b) | Type of article: System Review and Meta-analysis |
| 18 | (Park et al., 2021) | Type of article: Only Abstract. |
| 19 | (Guo et al., 2021a) | Type of article: Meta-analysis |
| 20 | (Miyata et al., 2021) | Type of article: Review |
| 21 | (Pilati & Pilati, 2021) | Type of article: Meta-analysis |
| 22 | (Wei, Zhong, Wang, & Huang, 2021) | Type of article: Meta-analysis |
| **Reason for exclusion: Intervention (exposures) was not PD patients and Outcome was not UC incidence (8 records excluded)** | | |
| 1 | (Famili et al., 2007) | Intervention: Androgen deprivation therapy  Outcome: Periodontal disease in men with prostate cancer. |
| 2 | (Tramini, Montal, & Valcarcel, 2007) | Intervention: Tooth loss.  Outcome: Cancer, Nephrological disease. |
| 3 | (Thorman, Neovius, & Hylander, 2009b) | Intervention: Oral fungal infection.  Outcome: Chronic kidney disease. |
| 4 | (Hutton, Bradwell, English, & Chapple, 2010) | Intervention: Treatment for a solid tumour or lymphoma.  Outcome: Oral health. |
| 5 | (Sereti, Stamatiou, Thanos, Semergidis, & Dimitriadis, 2010) | Intervention: Dental treatment.  Outcome: Advanced prostate cancer patients before initiation of zoledronic acid treatment. |
| 6 | (Droz et al., 2012) | Intervention: Cultural mediation; low income; illegal immigrants and so on.  Outcome: Cancer. |
| 7 | (Sabharwal, Gomes-Filho, Stellrecht, & Scannapieco, 2018) | Intervention: periodontal therapy  Outcome: Systemic diseases |
| 8 | (Nishikawa & Yamamoto, 2020) | Intervention: Dental Care  Outcome: Metabolic Health Status |
| **Reason for exclusion: Overlap study (5 record excluded)** | | |
| 1 | (Michaud, Liu, Meyer, Giovannucci, & Joshipura, 2008) | Michaud 2008 and Michaud 2016 are all result from the Health Professionals Follow-up Study (HPFS). |
| 2 | (Mai et al., 2014) | Mai 2014 and Mai 2016 are all result from the Buffalo OsteoPerio Study (BOPS). |
| 3 | (Babic et al., 2015) | The same Aritcle (Periodontal bone loss and risk of epithelial ovarian cancer) was published into different Journals: Clinical Cancer Research & Cancer Research |
| 4 | (Lee, Kweon, Choi, Kim, & Choi, 2017) | Mai 2014 and Mai 2016 are all result from the National Health Insurance Service (NHIS). |
| 5 | (Oh, Lee, Giovannucci, & Keum, 2020) | Oh 2020 and Michaud 2016 are all result from the Health Professionals Follow-up Study (HPFS). |

**Reforance：**

Babic, A., Poole, E. M., Terry, K. L., Cramer, D. W., Teles, R. P., & Tworoger, S. S. (2015). Periodontal bone loss and risk of epithelial ovarian cancer in the Nurses' Health Study. Cancer Research, 75(15). doi:10.1158/1538-7445.AM2015-858

Corbella, S., Veronesi, P., Galimberti, V., Weinstein, R., Del Fabbro, M., & Francetti, L. (2018). Is periodontitis a risk indicator for cancer? A meta-analysis. PLoS One, 13(4), e0195683. doi:10.1371/journal.pone.0195683

da Silva, A. P. B., Alluri, L. S. C., Bissada, N. F., & Gupta, S. (2019). Association between oral pathogens and prostate cancer: building the relationship. Am J Clin Exp Urol, 7(1), 1-10.

Dizdar, O., Hayran, M., Guven, D. C., Yilmaz, T. B., Taheri, S., Akman, A. C., . . . Berker, E. (2017). Increased cancer risk in patients with periodontitis. Curr Med Res Opin, 33(12), 2195-2200. doi:10.1080/03007995.2017.1354829

Droz, J.-P., Cenciu, B., Lopoh, A., Guillier, A., Bianco, L., Fayette, J., . . . Couppié, P. (2012). Cancer in the elderly in an equatorial area: French Guiana. Aging Health, 8(3), 293-300. doi:10.2217/ahe.12.22

Famili, P., Cauley, J. A., & Greenspan, S. L. (2007). The effect of androgen deprivation therapy on periodontal disease in men with prostate cancer. J Urol, 177(3), 921-924. doi:10.1016/j.juro.2006.10.067

Fitzgerald, R. H., Jr., McInnes, B. K., & Manry, H. C. (1982). Renal cell carcinoma involving oral soft tissues. J Oral Maxillofac Surg, 40(9), 604-606. doi:10.1016/0278-2391(82)90294-4

Fitzpatrick, S. G., & Katz, J. (2010). The association between periodontal disease and cancer: a review of the literature. J Dent, 38(2), 83-95. doi:10.1016/j.jdent.2009.10.007

Grant, W. B. (2012). Disparities in periodontitis prevalence among chronic kidney disease patients. J Dent Res, 91(3), 321; author reply 322. doi:10.1177/0022034511431263

Guo, Z., Gu, C., Li, S., Gan, S., Li, Y., Xiang, S., . . . Wang, S. (2021). Periodontal disease and the risk of prostate cancer: a meta-analysis of cohort studies. Int Braz J Urol, 47. doi:10.1590/S1677-5538.IBJU.2020.0333

Guven, D. C., Dizdar, O., Akman, A. C., Berker, E., Yekeduz, E., Ceylan, F., . . . Hayran, M. (2018). Evaluation of cancer risk in patients with periodontal diseases. Journal of Clinical Oncology, 36(15_suppl), e13571-e13571. doi:10.1200/JCO.2018.36.15_suppl.e13571

Guven, D. C., Dizdar, O., Akman, A. C., Berker, E., Yekeduz, E., Ceylan, F., . . . Hayran, M. (2019). Evaluation of cancer risk in patients with periodontal diseases. Turk J Med Sci, 49(3), 826-831. doi:10.3906/sag-1812-8

Hutton, A., Bradwell, M., English, M., & Chapple, I. (2010). The oral health needs of children after treatment for a solid tumour or lymphoma. Int J Paediatr Dent, 20(1), 15-23. doi:10.1111/j.1365-263X.2009.00999.x

Hwang, I. M., Sun, L. M., Lin, C. L., Lee, C. F., & Kao, C. H. (2014). Periodontal disease with treatment reduces subsequent cancer risks. QJM, 107(10), 805-812. doi:10.1093/qjmed/hcu078

Jagodzinski, A., Johansen, C., Koch-Gromus, U., Aarabi, G., Adam, G., Anders, S., . . . Blankenberg, S. (2020). Rationale and Design of the Hamburg City Health Study. Eur J Epidemiol, 35(2), 169-181. doi:10.1007/s10654-019-00577-4

Joshy, G., Arora, M., Korda, R. J., Chalmers, J., & Banks, E. (2016). Is poor oral health a risk marker for incident cardiovascular disease hospitalisation and all-cause mortality? Findings from 172 630 participants from the prospective 45 and Up Study. Bmj Open, 6(8), e012386. doi:10.1136/bmjopen-2016-012386

Kshirsagar, A., Elter, J., Lohman, A., Singh, S., Offenbacher, S., & Beck, J. (2002). Periodontis is highly prevalent in a sample of chronic hemodialysis patients. Journal of the American Society of Nephrology, 13, 743A-743A.

Lee, J. H., Kweon, H. H., Choi, J. K., Kim, Y. T., & Choi, S. H. (2017). Association between Periodontal disease and Prostate cancer: Results of a 12-year Longitudinal Cohort Study in South Korea. J Cancer, 8(15), 2959-2965. doi:10.7150/jca.20532

Linden, G. J., Herzberg, M. C., & Working group 4 of joint, E. F. P. A. A. P. w. (2013). Periodontitis and systemic diseases: a record of discussions of working group 4 of the Joint EFP/AAP Workshop on Periodontitis and Systemic Diseases. J Clin Periodontol, 40 Suppl 14, S20-23. doi:10.1111/jcpe.12091

Linden, G. J., Lyons, A., & Scannapieco, F. A. (2013). Periodontal systemic associations: review of the evidence. J Periodontol, 84(4 Suppl), S8-S19. doi:10.1902/jop.2013.1340010

Liu, K., Liu, Q., Chen, W., Liang, M., Luo, W., Wu, X., . . . Yu, X. (2013). Prevalence and risk factors of CKD in Chinese patients with periodontal disease. PLoS One, 8(8), e70767. doi:10.1371/journal.pone.0070767

Ma, H., Zheng, J., & Li, X. (2020). Potential risk of certain cancers among patients with Periodontitis: a supplementary meta-analysis of a large-scale population. Int J Med Sci, 17(16), 2531-2543. doi:10.7150/ijms.46812

Mai, X., Freudenheim, J. L., La Monte, M. J., Hovey, K. M., Andrews, C. A., Genco, R. J., & Wactawski-Wende, J. (2014). Periodontal disease severity and incident cancer in postmenopausal women: The Buffalo OsteoPerio Study. Cancer Research, 74(19). doi:10.1158/1538-7445.AM2014-256

Michaud, D. S., Liu, Y., Meyer, M., Giovannucci, E., & Joshipura, K. (2008). Periodontal disease, tooth loss, and cancer risk in male health professionals: a prospective cohort study. Lancet Oncol, 9(6), 550-558. doi:10.1016/S1470-2045(08)70106-2

Migliorati, C. A. (2008). Periodontal diseases and cancer. Lancet Oncol, 9(6), 510-512. doi:10.1016/S1470-2045(08)70138-4

Nishikawa, K., & Yamamoto, M. (2020). Combined Associations of Body Mass Index and Metabolic Health Status on Medical and Dental Care Days and Costs in Japanese Male Employees: A 4-Year Follow-Up Study. J Epidemiol, 30(5), 201-207. doi:10.2188/jea.JE20180268

Oh, H., Lee, D. H., Giovannucci, E. L., & Keum, N. (2020). Gastric and duodenal ulcers, periodontal disease, and risk of bladder cancer in the Health Professionals Follow-up Study. Cancer Causes Control, 31(4), 383-391. doi:10.1007/s10552-020-01274-4

Ruokonen, H., Nylund, K., Furuholm, J., Meurman, J. H., Sorsa, T., Kotaniemi, K., . . . Heikkinen, A. M. (2017). Oral Health and Mortality in Patients With Chronic Kidney Disease. J Periodontol, 88(1), 26-33. doi:10.1902/jop.2016.160215

Ruospo, M., Palmer, S. C., Craig, J. C., Gentile, G., Johnson, D. W., Ford, P. J., . . . Strippoli, G. F. (2014). Prevalence and severity of oral disease in adults with chronic kidney disease: a systematic review of observational studies. Nephrol Dial Transplant, 29(2), 364-375. doi:10.1093/ndt/gft401

Sabharwal, A., Gomes-Filho, I. S., Stellrecht, E., & Scannapieco, F. A. (2018). Role of periodontal therapy in management of common complex systemic diseases and conditions: An update. Periodontol 2000, 78(1), 212-226. doi:10.1111/prd.12226

Schmalz, G., & Ziebolz, D. (2020). Special Issue "Oral Health and Systemic Diseases". J Clin Med, 9(10), 1-3. doi:10.3390/jcm9103156

Sereti, M., Stamatiou, G., Thanos, T., Semergidis, T., & Dimitriadis, I. (2010). Delay of zoledronic acid treatment in patients with prostate cancer who require ongoing dental work. European Urology, Supplements, 9(6), 561.

Shepherd, S. M., & Lyon, W. K. (1990). Gingival bleeding: initial presentation of prostatic cancer. J Fam Pract, 30(1), 98-100.

Shi, J., Leng, W., Zhao, L., Deng, C., Xu, C., Wang, J., . . . Peng, X. (2018). Tooth loss and cancer risk: a dose-response meta analysis of prospective cohort studies. Oncotarget, 9(19), 15090-15100. doi:10.18632/oncotarget.23850

Thorman, R., Neovius, M., & Hylander, B. (2009a). Clinical findings in oral health during progression of chronic kidney disease to end-stage renal disease in a Swedish population. Scand J Urol Nephrol, 43(2), 154-159. doi:10.1080/00365590802464817

Thorman, R., Neovius, M., & Hylander, B. (2009b). Prevalence and early detection of oral fungal infection: a cross-sectional controlled study in a group of Swedish end-stage renal disease patients. Scand J Urol Nephrol, 43(4), 325-330. doi:10.1080/00365590902836492

Tramini, P., Montal, S., & Valcarcel, J. (2007). Tooth loss and associated factors in long-term institutionalised elderly patients. Gerodontology, 24(4), 196-203. doi:10.1111/j.1741-2358.2007.00183.x

Wei, Y., Zhong, Y., Wang, Y., & Huang, R. (2020). Association between periodontal disease and prostate cancer: a systematic review and meta-analysis. Med Oral Patol Oral Cir Bucal. doi:10.4317/medoral.24308

Xie, W. Z., Jin, Y. H., Leng, W. D., Wang, X. H., Zeng, X. T., & investigators, B. (2018). Periodontal Disease and Risk of Bladder Cancer: A Meta-Analysis of 298476 Participants. Front Physiol, 9, 979. doi:10.3389/fphys.2018.00979

Yoshihara, A., Deguchi, T., Hanada, N., & Miyazaki, H. (2007). Renal function and periodontal disease in elderly Japanese. J Periodontol, 78(7), 1241-1248. doi:10.1902/jop.2007.070025

Zhao, D., Khawaja, A. T., Jin, L., Li, K. Y., Tonetti, M., & Pelekos, G. (2018). The directional and non-directional associations of periodontitis with chronic kidney disease: A systematic review and meta-analysis of observational studies. J Periodontal Res, 53(5), 682-704. doi:10.1111/jre.12565

Babic, A., Poole, E. M., Terry, K. L., Cramer, D. W., Teles, R. P., & Tworoger, S. S. (2015). Periodontal bone loss and risk of epithelial ovarian cancer in the Nurses' Health Study. Cancer Research, 75(15). doi:10.1158/1538-7445.AM2015-858

Corbella, S., Veronesi, P., Galimberti, V., Weinstein, R., Del Fabbro, M., & Francetti, L. (2018). Is periodontitis a risk indicator for cancer? A meta-analysis. PLoS One, 13(4), e0195683. doi:10.1371/journal.pone.0195683

da Silva, A. P. B., Alluri, L. S. C., Bissada, N. F., & Gupta, S. (2019). Association between oral pathogens and prostate cancer: building the relationship. Am J Clin Exp Urol, 7(1), 1-10.

Dizdar, O., Hayran, M., Guven, D. C., Yilmaz, T. B., Taheri, S., Akman, A. C., . . . Berker, E. (2017). Increased cancer risk in patients with periodontitis. Curr Med Res Opin, 33(12), 2195-2200. doi:10.1080/03007995.2017.1354829

Droz, J.-P., Cenciu, B., Lopoh, A., Guillier, A., Bianco, L., Fayette, J., . . . Couppié, P. (2012). Cancer in the elderly in an equatorial area: French Guiana. Aging Health, 8(3), 293-300. doi:10.2217/ahe.12.22

Famili, P., Cauley, J. A., & Greenspan, S. L. (2007). The effect of androgen deprivation therapy on periodontal disease in men with prostate cancer. J Urol, 177(3), 921-924. doi:10.1016/j.juro.2006.10.067

Fitzgerald, R. H., Jr., McInnes, B. K., & Manry, H. C. (1982). Renal cell carcinoma involving oral soft tissues. J Oral Maxillofac Surg, 40(9), 604-606. doi:10.1016/0278-2391(82)90294-4

Fitzpatrick, S. G., & Katz, J. (2010). The association between periodontal disease and cancer: a review of the literature. J Dent, 38(2), 83-95. doi:10.1016/j.jdent.2009.10.007

Grant, W. B. (2012). Disparities in periodontitis prevalence among chronic kidney disease patients. J Dent Res, 91(3), 321; author reply 322. doi:10.1177/0022034511431263

Guo, Z., Gu, C., Li, S., Gan, S., Li, Y., Xiang, S., . . . Wang, S. (2021). Periodontal disease and the risk of prostate cancer: a meta-analysis of cohort studies. Int Braz J Urol, 47. doi:10.1590/S1677-5538.IBJU.2020.0333

Guven, D. C., Dizdar, O., Akman, A. C., Berker, E., Yekeduz, E., Ceylan, F., . . . Hayran, M. (2018). Evaluation of cancer risk in patients with periodontal diseases. Journal of Clinical Oncology, 36(15_suppl), e13571-e13571. doi:10.1200/JCO.2018.36.15_suppl.e13571

Guven, D. C., Dizdar, O., Akman, A. C., Berker, E., Yekeduz, E., Ceylan, F., . . . Hayran, M. (2019). Evaluation of cancer risk in patients with periodontal diseases. Turk J Med Sci, 49(3), 826-831. doi:10.3906/sag-1812-8

Hutton, A., Bradwell, M., English, M., & Chapple, I. (2010). The oral health needs of children after treatment for a solid tumour or lymphoma. Int J Paediatr Dent, 20(1), 15-23. doi:10.1111/j.1365-263X.2009.00999.x

Hwang, I. M., Sun, L. M., Lin, C. L., Lee, C. F., & Kao, C. H. (2014). Periodontal disease with treatment reduces subsequent cancer risks. QJM, 107(10), 805-812. doi:10.1093/qjmed/hcu078

Jagodzinski, A., Johansen, C., Koch-Gromus, U., Aarabi, G., Adam, G., Anders, S., . . . Blankenberg, S. (2020). Rationale and Design of the Hamburg City Health Study. Eur J Epidemiol, 35(2), 169-181. doi:10.1007/s10654-019-00577-4

Joshy, G., Arora, M., Korda, R. J., Chalmers, J., & Banks, E. (2016). Is poor oral health a risk marker for incident cardiovascular disease hospitalisation and all-cause mortality? Findings from 172 630 participants from the prospective 45 and Up Study. Bmj Open, 6(8), e012386. doi:10.1136/bmjopen-2016-012386

Kshirsagar, A., Elter, J., Lohman, A., Singh, S., Offenbacher, S., & Beck, J. (2002). Periodontis is highly prevalent in a sample of chronic hemodialysis patients. Journal of the American Society of Nephrology, 13, 743A-743A.

Lee, J. H., Kweon, H. H., Choi, J. K., Kim, Y. T., & Choi, S. H. (2017). Association between Periodontal disease and Prostate cancer: Results of a 12-year Longitudinal Cohort Study in South Korea. J Cancer, 8(15), 2959-2965. doi:10.7150/jca.20532

Linden, G. J., Herzberg, M. C., & Working group 4 of joint, E. F. P. A. A. P. w. (2013). Periodontitis and systemic diseases: a record of discussions of working group 4 of the Joint EFP/AAP Workshop on Periodontitis and Systemic Diseases. J Clin Periodontol, 40 Suppl 14, S20-23. doi:10.1111/jcpe.12091

Linden, G. J., Lyons, A., & Scannapieco, F. A. (2013). Periodontal systemic associations: review of the evidence. J Periodontol, 84(4 Suppl), S8-S19. doi:10.1902/jop.2013.1340010

Liu, K., Liu, Q., Chen, W., Liang, M., Luo, W., Wu, X., . . . Yu, X. (2013). Prevalence and risk factors of CKD in Chinese patients with periodontal disease. PLoS One, 8(8), e70767. doi:10.1371/journal.pone.0070767

Ma, H., Zheng, J., & Li, X. (2020). Potential risk of certain cancers among patients with Periodontitis: a supplementary meta-analysis of a large-scale population. Int J Med Sci, 17(16), 2531-2543. doi:10.7150/ijms.46812

Mai, X., Freudenheim, J. L., La Monte, M. J., Hovey, K. M., Andrews, C. A., Genco, R. J., & Wactawski-Wende, J. (2014). Periodontal disease severity and incident cancer in postmenopausal women: The Buffalo OsteoPerio Study. Cancer Research, 74(19). doi:10.1158/1538-7445.AM2014-256

Michaud, D. S., Liu, Y., Meyer, M., Giovannucci, E., & Joshipura, K. (2008). Periodontal disease, tooth loss, and cancer risk in male health professionals: a prospective cohort study. Lancet Oncol, 9(6), 550-558. doi:10.1016/S1470-2045(08)70106-2

Migliorati, C. A. (2008). Periodontal diseases and cancer. Lancet Oncol, 9(6), 510-512. doi:10.1016/S1470-2045(08)70138-4

Nishikawa, K., & Yamamoto, M. (2020). Combined Associations of Body Mass Index and Metabolic Health Status on Medical and Dental Care Days and Costs in Japanese Male Employees: A 4-Year Follow-Up Study. J Epidemiol, 30(5), 201-207. doi:10.2188/jea.JE20180268

Oh, H., Lee, D. H., Giovannucci, E. L., & Keum, N. (2020). Gastric and duodenal ulcers, periodontal disease, and risk of bladder cancer in the Health Professionals Follow-up Study. Cancer Causes Control, 31(4), 383-391. doi:10.1007/s10552-020-01274-4

Ruokonen, H., Nylund, K., Furuholm, J., Meurman, J. H., Sorsa, T., Kotaniemi, K., . . . Heikkinen, A. M. (2017). Oral Health and Mortality in Patients With Chronic Kidney Disease. J Periodontol, 88(1), 26-33. doi:10.1902/jop.2016.160215

Ruospo, M., Palmer, S. C., Craig, J. C., Gentile, G., Johnson, D. W., Ford, P. J., . . . Strippoli, G. F. (2014). Prevalence and severity of oral disease in adults with chronic kidney disease: a systematic review of observational studies. Nephrol Dial Transplant, 29(2), 364-375. doi:10.1093/ndt/gft401

Sabharwal, A., Gomes-Filho, I. S., Stellrecht, E., & Scannapieco, F. A. (2018). Role of periodontal therapy in management of common complex systemic diseases and conditions: An update. Periodontol 2000, 78(1), 212-226. doi:10.1111/prd.12226

Schmalz, G., & Ziebolz, D. (2020). Special Issue "Oral Health and Systemic Diseases". J Clin Med, 9(10), 1-3. doi:10.3390/jcm9103156

Sereti, M., Stamatiou, G., Thanos, T., Semergidis, T., & Dimitriadis, I. (2010). Delay of zoledronic acid treatment in patients with prostate cancer who require ongoing dental work. European Urology, Supplements, 9(6), 561.

Shepherd, S. M., & Lyon, W. K. (1990). Gingival bleeding: initial presentation of prostatic cancer. J Fam Pract, 30(1), 98-100.

Shi, J., Leng, W., Zhao, L., Deng, C., Xu, C., Wang, J., . . . Peng, X. (2018). Tooth loss and cancer risk: a dose-response meta analysis of prospective cohort studies. Oncotarget, 9(19), 15090-15100. doi:10.18632/oncotarget.23850

Thorman, R., Neovius, M., & Hylander, B. (2009a). Clinical findings in oral health during progression of chronic kidney disease to end-stage renal disease in a Swedish population. Scand J Urol Nephrol, 43(2), 154-159. doi:10.1080/00365590802464817

Thorman, R., Neovius, M., & Hylander, B. (2009b). Prevalence and early detection of oral fungal infection: a cross-sectional controlled study in a group of Swedish end-stage renal disease patients. Scand J Urol Nephrol, 43(4), 325-330. doi:10.1080/00365590902836492

Tramini, P., Montal, S., & Valcarcel, J. (2007). Tooth loss and associated factors in long-term institutionalised elderly patients. Gerodontology, 24(4), 196-203. doi:10.1111/j.1741-2358.2007.00183.x

Wei, Y., Zhong, Y., Wang, Y., & Huang, R. (2020). Association between periodontal disease and prostate cancer: a systematic review and meta-analysis. Med Oral Patol Oral Cir Bucal. doi:10.4317/medoral.24308

Xie, W. Z., Jin, Y. H., Leng, W. D., Wang, X. H., Zeng, X. T., & investigators, B. (2018). Periodontal Disease and Risk of Bladder Cancer: A Meta-Analysis of 298476 Participants. Front Physiol, 9, 979. doi:10.3389/fphys.2018.00979

Yoshihara, A., Deguchi, T., Hanada, N., & Miyazaki, H. (2007). Renal function and periodontal disease in elderly Japanese. J Periodontol, 78(7), 1241-1248. doi:10.1902/jop.2007.070025

Zhao, D., Khawaja, A. T., Jin, L., Li, K. Y., Tonetti, M., & Pelekos, G. (2018). The directional and non-directional associations of periodontitis with chronic kidney disease: A systematic review and meta-analysis of observational studies. J Periodontal Res, 53(5), 682-704. doi:10.1111/jre.12565

Alluri, L. S. C., Paes Batista da Silva, A., Verma, S., Fu, P., Shen, D. L., MacLennan, G., . . . Bissada, N. F. (2021). Presence of Specific Periodontal Pathogens in Prostate Gland Diagnosed With Chronic Inflammation and Adenocarcinoma. CUREUS, 13(9), e17742. doi:10.7759/cureus.17742

Babic, A., Poole, E. M., Terry, K. L., Cramer, D. W., Teles, R. P., & Tworoger, S. S. (2015). Periodontal bone loss and risk of epithelial ovarian cancer in the Nurses' Health Study. Cancer Research, 75(15). doi:10.1158/1538-7445.AM2015-858

Corbella, S., Veronesi, P., Galimberti, V., Weinstein, R., Del Fabbro, M., & Francetti, L. (2018). Is periodontitis a risk indicator for cancer? A meta-analysis. PLoS One, 13(4), e0195683. doi:10.1371/journal.pone.0195683

da Silva, A. P. B., Alluri, L. S. C., Bissada, N. F., & Gupta, S. (2019). Association between oral pathogens and prostate cancer: building the relationship. Am J Clin Exp Urol, 7(1), 1-10.

Dizdar, O., Hayran, M., Guven, D. C., Yilmaz, T. B., Taheri, S., Akman, A. C., . . . Berker, E. (2017). Increased cancer risk in patients with periodontitis. Curr Med Res Opin, 33(12), 2195-2200. doi:10.1080/03007995.2017.1354829

Droz, J.-P., Cenciu, B., Lopoh, A., Guillier, A., Bianco, L., Fayette, J., . . . Couppié, P. (2012). Cancer in the elderly in an equatorial area: French Guiana. Aging Health, 8(3), 293-300. doi:10.2217/ahe.12.22

Famili, P., Cauley, J. A., & Greenspan, S. L. (2007). The effect of androgen deprivation therapy on periodontal disease in men with prostate cancer. J Urol, 177(3), 921-924. doi:10.1016/j.juro.2006.10.067

Fitzgerald, R. H., Jr., McInnes, B. K., & Manry, H. C. (1982). Renal cell carcinoma involving oral soft tissues. J Oral Maxillofac Surg, 40(9), 604-606. doi:10.1016/0278-2391(82)90294-4

Fitzpatrick, S. G., & Katz, J. (2010). The association between periodontal disease and cancer: a review of the literature. J Dent, 38(2), 83-95. doi:10.1016/j.jdent.2009.10.007

Grant, W. B. (2012). Disparities in periodontitis prevalence among chronic kidney disease patients. J Dent Res, 91(3), 321; author reply 322. doi:10.1177/0022034511431263

Guo, Z., Gu, C., Li, S., Gan, S., Li, Y., Xiang, S., . . . Wang, S. (2021a). Periodontal disease and the risk of prostate cancer: a meta-analysis of cohort studies. International braz j urol : official journal of the Brazilian Society of Urology, 47(6), 1120-1130. doi:10.1590/S1677-5538.IBJU.2020.0333

Guo, Z., Gu, C., Li, S., Gan, S., Li, Y., Xiang, S., . . . Wang, S. (2021b). Periodontal disease and the risk of prostate cancer: a meta-analysis of cohort studies. Int Braz J Urol, 47. doi:10.1590/S1677-5538.IBJU.2020.0333

Guven, D. C., Dizdar, O., Akman, A. C., Berker, E., Yekeduz, E., Ceylan, F., . . . Hayran, M. (2018). Evaluation of cancer risk in patients with periodontal diseases. Journal of Clinical Oncology, 36(15_suppl), e13571-e13571. doi:10.1200/JCO.2018.36.15_suppl.e13571

Guven, D. C., Dizdar, O., Akman, A. C., Berker, E., Yekeduz, E., Ceylan, F., . . . Hayran, M. (2019). Evaluation of cancer risk in patients with periodontal diseases. Turk J Med Sci, 49(3), 826-831. doi:10.3906/sag-1812-8

Hutton, A., Bradwell, M., English, M., & Chapple, I. (2010). The oral health needs of children after treatment for a solid tumour or lymphoma. Int J Paediatr Dent, 20(1), 15-23. doi:10.1111/j.1365-263X.2009.00999.x

Hwang, I. M., Sun, L. M., Lin, C. L., Lee, C. F., & Kao, C. H. (2014). Periodontal disease with treatment reduces subsequent cancer risks. QJM, 107(10), 805-812. doi:10.1093/qjmed/hcu078

Jagodzinski, A., Johansen, C., Koch-Gromus, U., Aarabi, G., Adam, G., Anders, S., . . . Blankenberg, S. (2020). Rationale and Design of the Hamburg City Health Study. Eur J Epidemiol, 35(2), 169-181. doi:10.1007/s10654-019-00577-4

Joshy, G., Arora, M., Korda, R. J., Chalmers, J., & Banks, E. (2016). Is poor oral health a risk marker for incident cardiovascular disease hospitalisation and all-cause mortality? Findings from 172 630 participants from the prospective 45 and Up Study. Bmj Open, 6(8), e012386. doi:10.1136/bmjopen-2016-012386

Kshirsagar, A., Elter, J., Lohman, A., Singh, S., Offenbacher, S., & Beck, J. (2002). Periodontis is highly prevalent in a sample of chronic hemodialysis patients. Journal of the American Society of Nephrology, 13, 743A-743A.

Lee, J. H., Kweon, H. H., Choi, J. K., Kim, Y. T., & Choi, S. H. (2017). Association between Periodontal disease and Prostate cancer: Results of a 12-year Longitudinal Cohort Study in South Korea. J Cancer, 8(15), 2959-2965. doi:10.7150/jca.20532

Linden, G. J., Herzberg, M. C., & Working group 4 of joint, E. F. P. A. A. P. w. (2013). Periodontitis and systemic diseases: a record of discussions of working group 4 of the Joint EFP/AAP Workshop on Periodontitis and Systemic Diseases. J Clin Periodontol, 40 Suppl 14, S20-23. doi:10.1111/jcpe.12091

Linden, G. J., Lyons, A., & Scannapieco, F. A. (2013). Periodontal systemic associations: review of the evidence. J Periodontol, 84(4 Suppl), S8-S19. doi:10.1902/jop.2013.1340010

Liu, K., Liu, Q., Chen, W., Liang, M., Luo, W., Wu, X., . . . Yu, X. (2013). Prevalence and risk factors of CKD in Chinese patients with periodontal disease. PLoS One, 8(8), e70767. doi:10.1371/journal.pone.0070767

Ma, H., Zheng, J., & Li, X. (2020). Potential risk of certain cancers among patients with Periodontitis: a supplementary meta-analysis of a large-scale population. Int J Med Sci, 17(16), 2531-2543. doi:10.7150/ijms.46812

Mai, X., Freudenheim, J. L., La Monte, M. J., Hovey, K. M., Andrews, C. A., Genco, R. J., & Wactawski-Wende, J. (2014). Periodontal disease severity and incident cancer in postmenopausal women: The Buffalo OsteoPerio Study. Cancer Research, 74(19). doi:10.1158/1538-7445.AM2014-256

Michaud, D. S., Liu, Y., Meyer, M., Giovannucci, E., & Joshipura, K. (2008). Periodontal disease, tooth loss, and cancer risk in male health professionals: a prospective cohort study. Lancet Oncol, 9(6), 550-558. doi:10.1016/S1470-2045(08)70106-2

Migliorati, C. A. (2008). Periodontal diseases and cancer. Lancet Oncol, 9(6), 510-512. doi:10.1016/S1470-2045(08)70138-4

Miyata, Y., Matsuo, T., Matsuda, T., Harada, J., Mukae, Y., Otsubo, A., . . . Sakai, H. (2021). PATHOLOGICAL SIGNIFICANCE OF PERIODONTAL DISEASE IN PATIENTS WITH UROTHELIAL CANCERS: CORRELATION WITH URINARY TRACT RECURRENCE AND OXIDATIVE STRESS. JOURNAL OF UROLOGY, 206, E699-E699.

Nishikawa, K., & Yamamoto, M. (2020). Combined Associations of Body Mass Index and Metabolic Health Status on Medical and Dental Care Days and Costs in Japanese Male Employees: A 4-Year Follow-Up Study. J Epidemiol, 30(5), 201-207. doi:10.2188/jea.JE20180268

Oh, H., Lee, D. H., Giovannucci, E. L., & Keum, N. (2020). Gastric and duodenal ulcers, periodontal disease, and risk of bladder cancer in the Health Professionals Follow-up Study. Cancer Causes Control, 31(4), 383-391. doi:10.1007/s10552-020-01274-4

Park, C. H., Kim, E. H., Myeongjee, L., Joong Bae, A., Sang Jun, S., Beom, S. H., . . . Kim, H. S. (2021). Periodontal disease and cancer risk: A nationwide population-based cohort study. Annals of Oncology, 32, S1261. doi:10.1016/j.annonc.2021.08.641

Pilati, S. F. M., & Pilati, P. V. F. (2021). Does periodontal disease have an association with prostate cancer? Evidence-based dentistry, 22(4), 140-142. doi:10.1038/s41432-021-0213-z

Ruokonen, H., Nylund, K., Furuholm, J., Meurman, J. H., Sorsa, T., Kotaniemi, K., . . . Heikkinen, A. M. (2017). Oral Health and Mortality in Patients With Chronic Kidney Disease. J Periodontol, 88(1), 26-33. doi:10.1902/jop.2016.160215

Ruospo, M., Palmer, S. C., Craig, J. C., Gentile, G., Johnson, D. W., Ford, P. J., . . . Strippoli, G. F. (2014). Prevalence and severity of oral disease in adults with chronic kidney disease: a systematic review of observational studies. Nephrol Dial Transplant, 29(2), 364-375. doi:10.1093/ndt/gft401

Sabharwal, A., Gomes-Filho, I. S., Stellrecht, E., & Scannapieco, F. A. (2018). Role of periodontal therapy in management of common complex systemic diseases and conditions: An update. Periodontol 2000, 78(1), 212-226. doi:10.1111/prd.12226

Schmalz, G., & Ziebolz, D. (2020). Special Issue "Oral Health and Systemic Diseases". J Clin Med, 9(10), 1-3. doi:10.3390/jcm9103156

Sereti, M., Stamatiou, G., Thanos, T., Semergidis, T., & Dimitriadis, I. (2010). Delay of zoledronic acid treatment in patients with prostate cancer who require ongoing dental work. European Urology, Supplements, 9(6), 561.

Shepherd, S. M., & Lyon, W. K. (1990). Gingival bleeding: initial presentation of prostatic cancer. J Fam Pract, 30(1), 98-100.

Shi, J., Leng, W., Zhao, L., Deng, C., Xu, C., Wang, J., . . . Peng, X. (2018). Tooth loss and cancer risk: a dose-response meta analysis of prospective cohort studies. Oncotarget, 9(19), 15090-15100. doi:10.18632/oncotarget.23850

Thorman, R., Neovius, M., & Hylander, B. (2009a). Clinical findings in oral health during progression of chronic kidney disease to end-stage renal disease in a Swedish population. Scand J Urol Nephrol, 43(2), 154-159. doi:10.1080/00365590802464817

Thorman, R., Neovius, M., & Hylander, B. (2009b). Prevalence and early detection of oral fungal infection: a cross-sectional controlled study in a group of Swedish end-stage renal disease patients. Scand J Urol Nephrol, 43(4), 325-330. doi:10.1080/00365590902836492

Tramini, P., Montal, S., & Valcarcel, J. (2007). Tooth loss and associated factors in long-term institutionalised elderly patients. Gerodontology, 24(4), 196-203. doi:10.1111/j.1741-2358.2007.00183.x

Wei, Y., Zhong, Y., Wang, Y., & Huang, R. (2020). Association between periodontal disease and prostate cancer: a systematic review and meta-analysis. Med Oral Patol Oral Cir Bucal. doi:10.4317/medoral.24308

Wei, Y., Zhong, Y., Wang, Y., & Huang, R. (2021). Association between periodontal disease and prostate cancer: A systematic review and meta-analysis. MEDICINA ORAL PATOLOGIA ORAL Y CIRUGIA BUCAL, 26(4), e459-e465. doi:10.4317/medoral.24308

Xie, W. Z., Jin, Y. H., Leng, W. D., Wang, X. H., Zeng, X. T., & investigators, B. (2018). Periodontal Disease and Risk of Bladder Cancer: A Meta-Analysis of 298476 Participants. Front Physiol, 9, 979. doi:10.3389/fphys.2018.00979

Yoshihara, A., Deguchi, T., Hanada, N., & Miyazaki, H. (2007). Renal function and periodontal disease in elderly Japanese. J Periodontol, 78(7), 1241-1248. doi:10.1902/jop.2007.070025

Zhao, D., Khawaja, A. T., Jin, L., Li, K. Y., Tonetti, M., & Pelekos, G. (2018). The directional and non-directional associations of periodontitis with chronic kidney disease: A systematic review and meta-analysis of observational studies. J Periodontal Res, 53(5), 682-704. doi:10.1111/jre.12565
