# Supplementary material for: Is periodontal disease a risk indicator for urogenital cancer? A systematic review and meta-analysis of cohort studies
Source: Front Oncol. 2022 Aug 9;12:697399. doi: 10.3389/fonc.2022.697399 (PMC9395701; doi:10.3389/fonc.2022.697399)
Supplement: Supplementary file 4 [file Table_4.docx]

| **Appendix Table 4. Results of subgroup based on urogenital cancer diagnoses** | | | | |
| --- | --- | --- | --- | --- |
| Subgroups | HR | 95%CI | | |
| **total UC^#^** |  |  |  |  |
| Arora2009(Urogenital)^$^ | 1.51 | 1.14 | - | 2.02 |
| Chung2016 | 1.30 | 1.21 | - | 1.39 |
| Michaud 2016(Urogenital) | 1.20 | 1.00 | - | 1.44 |
| Nwizu 2017 (Urogenital) | 1.19 | 0.99 | - | 1.27 |
| **Genital Neoplasms, Female** |  |  |  |  |
| **Uterine** |  |  |  |  |
| Arora2009(Uterine) | 1.20 | 1.16 | - | 4.18 |
| Mai2016 | 1.01 | 0.34 | - | 3.00 |
| Nwizu 2017(Uterine) | 1.07 | 0.86 | - | 1.32 |
| **Ovarian** |  |  |  |  |
| Babic2015 | 0.86 | 0.64 | - | 1.15 |
| Nwizu 2017 (Ovarian) | 1.14 | 0.88 | - | 1.47 |
| **Vaginal** |  |  |  |  |
| Nwizu 2017 (Vaginal) | 1.05 | 0.51 | - | 2.19 |
| **Vulvar** |  |  |  |  |
| Nwizu 2017 (Vulvar) | 1.22 | 0.60 | - | 2.45 |
| Genital# |  |  |  |  |
| Nwizu 2017 (Genital) | 1.10 | 0.95 | - | 1.29 |
| Over all# |  |  |  |  |
| Arora2009(Uterine) | 1.20 | 1.16 | - | 4.18 |
| Mai2016 | 1.01 | 0.34 | - | 3.00 |
| Nwizu 2017 (Genital) | 1.10 | 0.95 | - | 1.29 |
| Babic2015 | 0.86 | 0.64 |  | 1.15 |
| **Genital Neoplasms, Male** |  |  |  |  |
| **Prostatic** |  |  |  |  |
| Hujoel2003 | 1.66 | 0.86 | - | 3.17 |
| Arora2009(Prostate) | 1.47 | 1.04 | - | 2.07 |
| Michaud 2016(Prostate) | 1.17 | 0.94 | - | 1.47 |
| Michaud 2018 | 1.25 | 1.01 | - | 1.53 |
| Heikkila 2018 | 0.95 | 0.62 | - | 1.40 |
| Chung2020 | 1.34 | 1.02 | - | 1.76 |
| Kim 2020 | 1.24 | 1.16 | - | 1.32 |
| **Urologic Neoplasms** |  |  |  |  |
| **Bladder** |  |  |  |  |
| Arora2009(Bladder) | 1.13 | 0.59 | - | 2.20 |
| Michaud 2016(Bladder) | 1.38 | 0.93 | - | 2.05 |
| Nwizu 2017 (Bladder) | 1.10 | 0.81 | - | 1.49 |
| **Kidney** |  |  |  |  |
| Michaud 2016(Kidney) | 1.06 | 0.61 | - | 1.85 |
| Nwizu 2017 (Kidney) | 1.09 | 0.76 | - | 1.56 |
| Urologic& |  |  |  | |
| Michaud 2016(Urinary) | 1.26 | 0.92 | 1.74 | |
| Nwizu 2017 (Urinary) | 1.16 | 0.92 | 1.45 | |
| Over all& |  |  |  | |
| Arora2009(Bladder) | 1.13 | 0.59 | 2.20 | |
| Michaud 2016(Urinary) | 1.26 | 0.92 | 1.74 | |
| Nwizu 2017 (Urinary) | 1.16 | 0.92 | 1.45 | |

# This subgroup of studies includes multiple sites for UC or studies only overall UC data without subdivision of organs are provided, such as Arora 2009, includes bladder, uterine and prostate cancers; $ For studies with multiple sites, we will note the cancer sites in parentheses. The absence of a note indicates that the study has only one cancer sites. It is worth noting that. Chung 2016 has only UC data and not site-specific data, so we also do not note the location in parentheses.

HR: hazard ratio; 95%CI: 95% confidence interval.
