# Supplementary material for: Is periodontal disease a risk indicator for urogenital cancer? A systematic review and meta-analysis of cohort studies
Source: Front Oncol. 2022 Aug 9;12:697399. doi: 10.3389/fonc.2022.697399 (PMC9395701; doi:10.3389/fonc.2022.697399)
Supplement: Supplementary file 5 [file Table_5.docx]

| **Appendix Table for review. Records excluded after reading topic and abstract with reasons** | | |
| --- | --- | --- |
| **From 1966-01-01 to 2021-03-31** | | |
| **Reasons** | | No. of  studies |
| Animal studies | | 29 |
| Bacteria studies | | 44 |
| cancer therapy studies | | 93 |
| Case report | | 78 |
| Other disease therapy | | 134 |
| Drug efficacy and route of administration | | 77 |
| Cancer metastasis | | 103 |
| No direct relationship between PD and UC | | 29 |
| No mention of PD | | 74 |
| No mention of UC | | 78 |
| Osteonecrosis | | 49 |
| Other disease | | 54 |
| The subject of studies are tobacco /betel | | 10 |
| The subject of studies are transplantation | | 46 |
| Other studies no mention of PD and UC | | 93 |
| **Total** | | 991 |
| **Details for the records excluded after reading topic and abstract with reasons** | | |
| Reason | Studies | No. of  studies |
| Animal studies | (Azuma et al., 2017; Basi et al., 2011; Beyaz, Atasever, Apaydin, & Deniz, 2009; Brazzell & Borjesson, 2006; Busk et al., 2010; Chase et al., 2011; J. Chen et al., 2001; Cohen, Reif, Brodey, & Keiser, 1974; Deluchi, Ledur, Pavarini, & Poppl, 2018; M. Fedele, Gualillo, & Vecchione, 2012; Ichimaru et al., 2018; Johnson, 2020; C. J. Kim et al., 2017; E. Kim et al., 2018; Z. Liang et al., 2015; Lowenstine, McManamon, & Terio, 2016; Murai et al., 2012; Nakanishi et al., 2002; Niemiec, 2008; O'Neill et al., 2019; Re, Barbero, & Cuniberti, 2009; Sabattini, Bassi, & Bettini, 2015; Sessa et al., 2010; Silva et al., 2019; Turturro, Duffy, Hass, Kodell, & Hart, 2002; Q. Wang, Kessler, Kensler, & Dechow, 2016; Williams, Annetti, & Nagy, 2018; Yu & Tsai, 2001; Y. Zhang, Knutsen, Brown, & Ruest, 2013) | 29 |
| Bacteria studies | (Arweiler et al., 2011; Balle et al., 2020; Cao et al., 2018; Chandler, 2014; Chaudhary, Conway, & Schlundt, 2018; J. Chen, Domingue, & Sears, 2017; Conway de Macario & Macario, 2009; Cummins & Tangney, 2013; Di Cerbo, Palmieri, Aponte, Morales-Medina, & Iannitti, 2016; Doolittle & Webster-Cyriaque, 2014; Efenberger, Agier, Pawlowska, & Brzezinska-Blaszczyk, 2015; Fan et al., 2018; Fang et al., 2020; Gargano & Hughes, 2014; Guerrero-Preston et al., 2017; Hasegawa-Nakamura et al., 2011; Huston & Tachedjian, 2020; Inaba et al., 2016; Kuroe et al., 2004; Lenoir-Wijnkoop et al., 2007; G. Liang et al., 2020; Liao, Luo, Peng, & Zeng, 2021; Marwaha, Morris, & Rigby, 2020; Mousa, 2016; Nagasawa et al., 2019; Ngwarai, Ah Tow, Nicol, & Kaba, 2016; Nichols, Peters, & Patterson, 2019; Ohadian Moghadam & Momeni, 2021; Olczak-Kowalczyk et al., 2012; Olsen, 2015; Parida & Sharma, 2019; Porter, Shrestha, Peiffer, & Sfanos, 2018; Rose, 2005; Ruggiero et al., 2018; Sakagami et al., 2008; H. N. Shah, Gharbia, & O'Toole, 1992; V. P. Singh, Sharma, Babu, Rizwanulla, & Singla, 2013; Thomas, Suzuki, & Zhao, 2015; Tlaskalova-Hogenova et al., 2004; Vasishta, Anjaneyalu, Sivaswamy, & Anjaneyalu, 2020; Vieira, Castelo, Ribeiro, & Ferreira, 2017; Vitetta, Briskey, Hayes, Shing, & Peake, 2012; Zandieh et al., 2014; Zitvogel & Kroemer, 2021) | 44 |
| cancer therapy studies | (Abdolkarimi et al., 2017; Abed et al., 2017; Aghajanian et al., 2015; Ai, Xu, Yang, He, & Luo, 2019; Akahane et al., 2018; Alexandrescu, Dasanu, & Kauffman, 2009; K. Anderson & Eskander, 2018; Asmane et al., 2011; Baldini et al., 2012; Beltramini et al., 2012; Bodnar, Gornas, & Szczylik, 2011; Bulbul & Kucukguzel, 2019; Burris et al., 2011; Burtness, 2017; Cartron et al., 2014; Charles, 2018; Chaurasiya & Mishra, 2018; J. Chen, Wei, et al., 2020; Chien, Lin, Cheng, Wen, & Yang, 2013; Chung et al., 2017; Chura, Van Iseghem, Downs, Carson, & Judson, 2007; Duan et al., 2020; El-Rabbany et al., 2019; Eliyas, Al-Khayatt, Porter, & Briggs, 2013; Flores Cuéllar, Sánchez Yáñez, & González Contreras, 2011; Frumovitz & Sood, 2007; Gnant et al., 2009; M. S. Gordon et al., 2014; Gubbi, Kendrick, & Finkler, 2014; Habib et al., 2016; Hashiguchi et al., 2015; Holgado, Alvarez-Fuentes, Fernandez-Arevalo, & Arias, 2011; Howard et al., 2017; Y. Hu et al., 2013; Ishak, Aad, Kyei, & Farhat, 2014; Ivy, Liu, Lee, Matulonis, & Kohn, 2016; Jaladat et al., 2015; Joharatnam-Hogan et al., 2019; Kamba & McDonald, 2007; Kavanagh et al., 2010; Kobayashi, Furukawa, Takahashi, & Murata, 2003; Koganemaru et al., 2012; Lelievre et al., 2018; Lorusso et al., 2021; Love, Patterson, Prose, & Atwater, 2012; Martin, Urban, Liao, & Goff, 2016; Montebugnoli, Checchi, & Marangolo, 1991; Muniraj, Siddharth, & Sharma, 2019; Ommer et al., 2020; Papadopoulou et al., 2018; P. Patel & Sheth, 2013; Peng et al., 2014; Penson et al., 2014; Prasad, Huang, Prasad, Miller, & Brenner, 2017; Richards, Pantanowitz, & Dezube, 2011; Risse et al., 1995; Rosen, Gordon, Robert, & Matei, 2014; Ross, Miller, & Rojas Hernandez, 2017; Sandomenico et al., 2012; Scott & Thomas, 2008; Shan, Shen, & Wang, 2020; Smith, Robin, & Ford, 2012; Soliman et al., 2019; Soory, 2010; Srikanth & Chen, 2016; Stone, Sood, & Coleman, 2010; Sugiyama et al., 2017; Sunakawa et al., 2014; Sutherland et al., 2018; K. Suzuki et al., 1989; Tao et al., 2019; Tao et al., 2018; Theou-Anton, Faivre, Dreyer, & Raymond, 2009; Tripathi, Kaymakcalan, LeBoeuf, & Harshman, 2016; Trost et al., 2013; van Veelen, Korsse, van de Laar, & Peppelenbosch, 2011; Vardas et al., 2018; Vijayaraghavan, Karunanithi, Karthikeyan, & Basu, 2018; Voss et al., 2017; Watts et al., 2020; Wibowo, Pollock, Hollis, & Wassersug, 2016; Workalemahu, Abdela, & Yenit, 2020; J. Y. Wu et al., 2010; P. Y. Wu et al., 2020; Wuketich, Hienz, & Marosi, 2012; Yamamoto, Natio, Hara, Kudo, & Miwa, 2016; P. Yang, 1983; Zhan, Li, & Ge, 2019; Y. Zhang et al., 2020; Zheng, Han, Yap, Xie, & Chen, 2005; S. Zhou et al., 2004; S. M. Zhu, Guo, Li, Luo, & Yao, 2013; Zinzani, 2006) | 93 |
| Case report | (Agosti, Espinoza, & Ramirez, 1989; Agrillo, Nastro Siniscalchi, Facchini, Filiaci, & Ungari, 2012; Alifrangis, Evans, Williams, & Seckl, 2011; Almazyad, Woo, & Villa, 2019; Almeida, Heckler, Fong, Lai-Cheong, & McGrath, 2013; Ammari & Fung, 2007; Asherson et al., 2008; Ashrafi, Derakhshandeh, Movahedian, & Moghaddas, 2017; Aug, Bhatti, Al-Tawil, Sani, & Ibrahim, 2016; Badros et al., 2012; Bakeen, Hiyarat, & Al-Ubaidy, 1976; Basile et al., 2019; Bathi, Kumar, & Natarajan, 2002; Baumgartner & Eggli, 1989; Bektas, Samanci, Cokgezer, Keskin, & Demirelli, 2019; Ben Ayed, el Mezni, Letourneau, & Najeh, 1987; Berends, Olgers, & Ter Maaten, 2016; Bezinelli et al., 2019; Bhatia, Vaid, Rawal, & Patole, 2007; Biesecker et al., 2020; Boddu et al., 2017; Bowles, Depala, & Beski, 2013; Brocheriou & Laufer, 1979; Chatelain, Parc, Christin-Maitre, Parc, & Flejou, 2002; Colombo et al., 2005; de Pina-Neto et al., 1998; Debarbieux et al., 2014; Divya, Moran, & Atkin, 2010; Elkhoury et al., 2004; Fitzgerald Jr, McInnes, & Manry, 1982; Giuliani, Lajolo, Lucchese, Marino, et al., 2010; Grimm et al., 2012; Haque, Kumar, & Beegle, 2009; Hassona, Almuhaisen, Almansour, & Scully, 2017; Healy, Tobin, Kirby, & Flint, 2006; Heinrich, McKeever, & Eisenschenk, 2011; Heng & Heng, 1995; Hinson, Siegel, & Stack, 2015; Hiraiwa & Izumi, 2013; James & Lupton, 1988; Kafadar, Erdinçler, & Erdinçler, 2006; Kallas, Green, Hewison, White, & Kline, 2010; Kamphuis et al., 2001; King, 2019; Lajara, Balakrishnan, Castrodad, Colanta, & Khader, 2019; Lidgi, Embon, Turani, & Sazbon, 1989; Manohar, Verma, Mannan, & Bhuvaneshwari, 2020; Mattheos, Caldwell, Petcu, Ivanovski, & Reher, 2013; Misaka, Kishimoto, Kawahigashi, Sata, & Nagao, 2016; Mobarki et al., 2020; K. Mori et al., 2017; Murillo, Bagan, Hens, Diaz, & Leopoldo, 2013; Naz, Aslan, Sonmez Tamer, & Naz, 2018; Nelson et al., 2009; Nicolatou-Galitis, Bafaloukos, et al., 2012; Nicolatou-Galitis, Psyrri, Pectasides, & Economopoulos, 2010; Ohashi et al., 2021; Ozet, Guran, & Beksac, 2008; A. Pai, Prasad, & Dyasanoor, 2012; I. Park & Kang, 2017; Prabakaran, Prabhu, Kar, & Basu, 2019; Razis, Karina, Karanastassi, & Fountzilas, 2006; Reap, McDonald, Balakrishnan, & Vakhariya, 2020; Richters, Grulich, Ellard, Hendry, & Kippax, 2003; Rittinger, Gottardi, & Wiesmayr, 2013; Rohart, Badelon, Fajnkuchen, Nghiem-Buffet, & Chaine, 2008; Ruhoy & Yates, 2016; H. Sakashita, Miyata, Miyamoto, & Kurumaya, 1996; M. Sakashita et al., 2017; Selvi, Faquin, Michaelson, & August, 2016; Sukhumthammarat, Putthapiban, & Vutthikraivit, 2019; Suryadevara et al., 2008; Thorwarth, Rupprecht, Schlegel, Neureiter, & Kessler, 2004; True et al., 1972; Van der Cruyssen, Grisar, Maes, & Politis, 2017; H. Wang, Sun, & Tan, 2015; J. Zhu et al., 2015; Zhumagaliyeva, Nurgaliyeva, & Karazhanova, 2018) | 78 |
| Other disease therapy | (A Loudon, 2013; Agnihotry, Thompson, Fedorowicz, van Zuuren, & Sprakel, 2019; Ahuja, 2003; American Diabetes, 2015; Ángel Descalzo, 2007; Antoniu, 2013; Bachmann, Crosby, Feldman, Ronkin, & Constantine, 2011; Beastall & Rainbow, 2008; Belden, 2003; Berger & Houff, 2009; Berth-Jones, 2005; Best, Xu, Patchen, & Cassano, 2019; Borchard & Orchard, 2008; Broussalis, Trinka, Kraus, McCoy, & Killer, 2013; Burden, Mullally, & Sandler, 2001; Burrowes & Van Houten, 2005; Button & Coles, 2010; Chai et al., 2020; Chakravarthy et al., 2015; Chiu & Tsai, 2011; Cook et al., 2011; Cotruta, Trifu, Costache, Popescu, & Florea, 2010; Cunningham, 2006; Dandona & Rosenberg, 2010; Davies, Longstreth, & Jamali, 2001; Eldabagh & Foley, 2016; Esposito et al., 2017; Esposito et al., 2009; Esposito & Worthington, 2013; Farrell, 2003; Figuero et al., 2020; Frydman & Fairley, 2011; Glade, 2013; Gore, Swerdloff, & Rajfer, 2005; Gould et al., 2018; Gross et al., 2015; Grusovin, Coulthard, Worthington, George, & Esposito, 2010; Hare, Arnott, & Satsangi, 2008; Hider et al., 2015; Holley, 2013; Hollis & Wagner, 2013; Hong, Han, Lee, Kim, & Jee, 2019; Hunyady et al., 2016; Ishizawa et al., 2016; Isono et al., 2009; Itabashi et al., 2011; Jakobsen et al., 2017; Jiang, Wang, Joshi, & Christoforidis, 2013; Jobanputra, Maggs, Homer, & Bevan, 2002; Johansson, 2016; Jorgensen et al., 2017; Kato, Lopes, Jaguar, Silva, & Alves, 2013; Kazlow Stern, Tripp, Ho, & Lebwohl, 2005; Kharkwal, Sharma, Huang, Dai, & Hamblin, 2011; Klavdianou et al., 2020; Kobos & Bussel, 2008; Kolios et al., 2016; Koopman, Beelen, Gilhus, de Visser, & Nollet, 2015; Kurup & Chan, 2005; Kyrgidis, Koloutsos, & Vahtsevanos, 2010; Lambert et al., 2020; Landesberg, Eisig, Fennoy, & Siris, 2009; Langley et al., 2018; Lau et al., 2012; Lawitschka et al., 2011; Lazarchik & Haywood, 2010; Lichtenstein, Abreu, Cohen, Tremaine, & American Gastroenterological, 2006; C. T. Lin et al., 2019; S. Y. Lin et al., 2017; Lodi et al., 2021; Lotti et al., 2020; Lustig & Cunningham, 2003; Ma et al., 2019; Machorowska-Pieniazek et al., 2013; Majumder, Sudharshan, & Biswas, 2009; Manna et al., 2008; Marcard et al., 2008; Marder & McCune, 2007; Mok, 2006, 2007; Morawiec et al., 2013; Moyad, 2003; Mukherjee, Babu, Rajesh, & Masthan, 2019; Nagi, Yashoda Devi, Rakesh, Reddy, & Patil, 2015; Nakashima & Akamine, 2005; Newnham et al., 2014; Oelke, Wagg, Takita, Buttner, & Viktrup, 2017; Olszewska et al., 2007; Ottria et al., 2018; E. J. Park et al., 2013; Perez & Patton, 2009; Perry et al., 2002; Peterlik & Cross, 2009; Peters et al., 2014; Ponseti et al., 2008; R. Powell et al., 2016; Quattrocchi et al., 2016; Rajendran et al., 2019; Riley & Lamont, 2013; Rizvi, 2007; Rodero et al., 2018; Rogers, Williams, & Roman, 2004; Rojas-Reyes, Granados Rugeles, & Charry-Anzola, 2014; Rosa, Mio, Andreadou, & Sumbayev, 2020; Saltzstein, Sieber, Morris, & Gallo, 2005; Schad, Axtner, Kroz, Matthes, & Steele, 2018; Schneider-Gold, Hartung, & Gold, 2006; Scholl, Kopp, Bohle, & Jensen-Jarolim, 2006; Sebaratnam & Murrell, 2014; Seftel, 2007; Semeraro, Morescalchi, Parmeggiani, Arcidiacono, & Costagliola, 2011; Seo, Kim, & Park, 2020; Shahrokni, Rajebi, & Saif, 2009; Soe et al., 2020; Sonu, Blonski, Lin, & Lichtenstein, 2010; Spector, 2009; Stein, Livada, & Tipton, 2014; Sterenborg & Robinson, 2010; Stock, Oyston, & Norman, 2013; Tanaka et al., 2013; Theodoraki & Bouloux, 2009; Thirumalai, Berkseth, & Amory, 2017; Urasaki et al., 2021; Valverde, 2008; van Dijk et al., 2018; Walsh et al., 2021; Walters, Willis, Cooper, & Craig, 2020; Watanabe et al., 2014; White & Lynch, 2007; Willis & Robertson, 2014; Woolacott et al., 2006; Yoshikawa et al., 2020; Young-Choi et al., 2013; R. Zhang & Naughton, 2010) | 134 |
| Drug efficacy and route of administration | (Aird, 2003; Alam, Singh, & Singh, 2011; Allison, 2008; Amatya et al., 2013; Arruebo, Vilaboa, & Santamaria, 2010; Beeley, Ansell, & Docherty, 1994; Beenken & Mohammadi, 2009; Belvisi & Bottomley, 2003; Brindha et al., 2017; Colson & De Broe, 2005; Crommelin, Mastrobattista, Hawe, Hoogendoorn, & Jiskoot, 2020; Cudic & Fields, 2009; D'Souza, Faraj, & DeLuca, 2005; Dorman et al., 2010; Dove, 2002; Emig et al., 2013; Eng-Chong et al., 2012; Frye, 2006; Garrido-Mesa, Zarzuelo, & Galvez, 2013; Gelosa, Castiglioni, Camera, & Sironi, 2020; A. J. Gordon, Conley, & Gordon, 2013; S. G. Harris, Padilla, Koumas, Ray, & Phipps, 2002; Hatz, Schremser, & Rogowski, 2014; Hemady, Chan, & Nguyen, 2005; J. Hu, Van den Steen, Sang, & Opdenakker, 2007; Iram, Khan, & Husain, 2017; Je, Schutz, & Choueiri, 2009; Jouyban, Fakhree, & Shayanfar, 2010; Jurenka, 2008; Kanakura et al., 2014; Kannel, 2008; Kapoor, Dureja, & Chadha, 2009; Kashani & Mearza, 2008; Krishnamurthy, Hoffman, & Del Priore, 2009; Kuiper-Geertsma & Derksen, 2003; Kültür & Sami, 2009; Kunnumakkara et al., 2017; Kwon et al., 2012; Lai, Tang, & Wang, 2013; Larson, Nussenblatt, & Sen, 2011; S. Lee et al., 2009; W. Y. Lee, Asadujjaman, & Jee, 2019; W. Li, Saji, Sato, Noda, & Toi, 2013; Lia, Shib, Tang, & Duan, 2009; J. Liu, Lu, Wu, Goyer, & Waalkes, 2008; Lovett & Ganta, 2010; Manika, Neelkamal, Sankireddy, Mittal, & Vinayak, 2015; Mitra & Wu, 2010; T. Mizuno et al., 2013; Moghaddasi, 2010; Mondal, Adhikari, Banerjee, Amin, & Jha, 2020; Mundargi, Babu, Rangaswamy, Patel, & Aminabhavi, 2008; S. S. Pai, Tilton, & Przybycien, 2009; G. C. Patel & Dalwadi, 2013; Patryk, Maciej, Malgorzata, & Wieslaw, 2020; Petty et al., 2009; Pizzo, Guiglia, Lo Russo, & Campisi, 2010; Reid & Holen, 2009; Rojas-Garcia, Alberu-Gomez, & Medina-Franco, 2010; Roudebush, Allen, Dodd, & Novotny, 2004; Sadarangani, Estes, & Steckelberg, 2015; Sahoo, Mandal, Dwivedi, & Kumar, 2020; Schaffer, Schaffer, & Bar-Sela, 2015; Schoubben, Ricci, & Giovagnoli, 2019; Shanmugam & Banerjee, 2011; Sharifi-Rad et al., 2020; Shimazaki et al., 2011; Shirataki et al., 2005; A. Singh, Kumar Sharma, & Malviya, 2012; Sinha & Trehan, 2005; Sun et al., 2020; Tsung & Burgess, 2012; Vandenbroucke & Libert, 2014; Vandooren, Van den Steen, & Opdenakker, 2013; Varshosaz, 2007; S. Wang, Zhang, Chen, & Wang, 2013; Wischke & Schwendeman, 2012) | 77 |
| Cancer metastasis | (Ali & Mohamed, 2016; Allon, Pessing, Kaplan, Allon, & Hirshberg, 2014; Alrumaih, Arian, Alhedyani, Al-Zaher, & Dababo, 2015; Altintas, Vardar, Aridogan, Doran, & Tuncer, 1995; D. A. Anderson, Woltman, Kovach, & Konety, 2004; Angiero & Stefani, 2008; Aoun, Hayek, & Nasseh, 2020; Apolo et al., 2017; Aswath, Balakrishnan, Shyamsundar, & Thirrugnanamurthy, 2017; Aydın, Alparslan, Ayık, Kayıkçıoğlu, & Öztürk, 2018; Bennardo, Buffone, Muraca, Antonelli, & Giudice, 2020; Brunello, Saia, Bedogni, Scaglione, & Basso, 2009; Buchner & Begleiter, 1980; Cassoni et al., 2014; B. Chen, Li, & Wang, 2018; J. Chen, Quan, et al., 2020; Chossegros, Blanc, Cheynet, Bataille, & Tessier, 1991; T. Choueiri, Agarwal, et al., 2016; T. Choueiri, Michaelson, et al., 2016; T. K. Choueiri, Agarwal, et al., 2015; T. K. Choueiri, Michaelson, et al., 2015; T. K. Choueiri et al., 2014; Cortes-Vazquez et al., 2020; D'Silva et al., 2006; Danon et al., 1999a, 1999b; de Courten, Irle, Samson, & Lombardi, 2001; De Pasquale et al., 2011; Doumas et al., 2000; Doval et al., 1994; Eccles, 2010; Eda, Saito, Yamamura, Kawahara, & Takahashi, 1973; Eivazi, Fakhrgoo, & Estakhri, 2011; Epstein, Knowling, & Le Riche, 1987; Fantasia & Chen, 1979; Fernandez-Barriales, Garcia-Montesinos, Garcia Reija, Mayorga Fernandez, & Saiz Bustillo, 2013; Fujinaga et al., 2014; Fusco et al., 2015; Genin et al., 2008; Gilabert et al., 2011a, 2011b; Gilabert et al., 2013; "[Gingival metastasis, mediastinal syndrome and acute abdomen]," 1982; Giuliani, Lajolo, Lucchese, Ricci, & Favia, 2010; Godby, Sonntag, & Cosentino, 1967; Hatziotis, Constantinidou, & Papanayotou, 1973; Hirshberg, Shnaiderman-Shapiro, Kaplan, & Berger, 2008; Hope, Morton, Newlands, Butler-Manuel, & Madhuri, 2017; Horstman, Gruhl, Smith, Ganti, & Shonka, 2018; Jacobs, Ruben, & Lyon, 1966; Kawashima et al., 2012; S. M. Kim et al., 2009; Kosem, Cankaya, & Kaya, 2004; Maestre-Rodriguez et al., 2009; Makos & Psomaderis, 2009; Mapelli et al., 2013; Martinez Conde, Lopez Cedrun, Aguirre Urizar, Rosell Cerro, & Llarena Ibarguren, 1990; Martinez Moragon et al., 1994; Medina, Barba, Torres, & Trujillo, 2001; M. Mori, Amano, Sakamoto, & Kimura, 1974; M. Mori, Sakamoto, Amano, & Kimura, 1974; Muller-Mattheis, Hagen, Frenzel, & Ackermann, 1989; Munakata, Sawair, Cheng, & Saku, 2009; Murata, Ikeda, Hasegawa, Nakagawa, & Nishiyama, 2019; Murgod, Girish, Shyamala, & Savita, 2015; Nicolatou-Galitis, Migkou, et al., 2012; Nifosi, Bressand, Nifosi, Nifosi, & Damseaux, 2017; Nishide & Kanamura, 2006; Oudard et al., 2016; Owosho et al., 2016; Pandhi, Beci, & Dhawar, 1975; Pastremoli, 1991; Pertusa Pena, Llarena Ibarguren, Zabala Egurrola, Lopez Cedrun, & Martinez Conde, 1989; Petrylak et al., 2020; Piattelli, Fioroni, & Rubini, 1999; Ravaud et al., 2017; Rizzardi, Schneider, Barresi, Brollo, & Melato, 2009; Roa & Mizrahi, 1972; Rozenblit, DeCarlo, Lin, & Nierodzik, 2017; Ruffion et al., 2000; Sáenz et al., 2007; Saimura et al., 2012; Samelis et al., 2011; Sasaki et al., 2008; Scolozzi et al., 2006; Sellin et al., 2015; Seoane et al., 2009; Shafiee et al., 2011; C. H. Shah et al., 2019; Sikka, Sikka, Kaur, & Shetty, 2013; Stojanovic, Krasic, Trajkovic, & Petrovic, 2020; Takasaki et al., 2018; Takayama, Nagata, Kai, Sugiyama, & Ozono, 2013; Tan, Alexe, & Reiss, 2009; Torregrossa et al., 2016; Tsianos, Karentzos, & Papadopoulos, 1987; Woo et al., 2017; Y. T. Wu, 1990; Yahyaoui et al., 2020; R.-H. Yang, Ting, & Chu, 2016; H. L. Zhang et al., 2015; W. Zhang et al., 2013; X. L. Zou et al., 2013) | 103 |
| No direct relationship between PD and UC | (Abiko et al., 2007; Amjadi et al., 2016; H. C. Anderson, Mulhall, & Garimella, 2010; Ataie-Kachoie, Pourgholami, & Morris, 2013; Atkinson, 2008; Bochenek et al., 2013; Brusca & Brusca, 2011; Buczko, Zalewska, & Szarmach, 2015; Caskey, 2006; dos Santos, da Silva, & Caxito, 2015; Fejzo, Burch, Mullin, MacGibbon, & Pasaniuc, 2020; Gaekwad & Gujjari, 2012; Gayathri, Muthukumar, Joseph, & Suresh, 2014; Gion & Fabricio, 2013; Goel, Chu, Sedlis, Friedman, & Blum, 2009; Hay et al., 2017; Javed, Bello Correra, Chotai, Tappuni, & Almas, 2010; J. Liu & Duan, 2012; Lohler, Gerstner, Bootz, & Walther, 2014; Mehdi, Al Bahrani, Al Lawati, Al Mandhari, & Al Lawati, 2017; Murray et al., 2013; Nct, 2015; Rajesh, Mangai, Babu, & Malathi, 2020; S. Sharma, Sharma, Gill, Shrivastav, & Shrivastav, 2013; Sonis, Treister, Chawla, Demetri, & Haluska, 2010; Thiruppathy, Kishore Kumar, Amaladas, & Pavani, 2019; Vogtmann et al., 2020; J. Yoon, Seo, Oh, & Yoon, 2016; Zielinski & Travis, 2004) | 29 |
| No mention of PD | ("Abstracts of the 11th Congress of the European College for the Study of Vulval Disease," 2016; Adjei Boakye et al., 2018; Aida, Takebayashi, & Matsui, 1987; Al-Muzian, Almuzian, Mohammed, Ulhaq, & Keightley, 2021; A. Amini, Masoumi Moghaddam, Morris, & Pourgholami, 2012; Aoki & Matsubara, 2013; Asada, 1974; Asahina, Shirai, Horita, & Saito, 2012; Bateman, Sun, Hood, Flint, & Conrads, 2010; Bauer et al., 2012; Bercaw, Sanchez, Byrd, Bhattacharjee, & Dietrich, 2010; Boussemart et al., 2014; Buckley, 2011, 2012; Bullon & Navarro, 2017; Cho et al., 2014; Clifford et al., 2008; Cullen et al., 2009; Dupuis & Coard, 2009; Etokebe et al., 2009; Fernandez-Medarde & Santos, 2011; Gabaldon & Haro-Gonzalez-Vico, 2019; Gillison et al., 2014; Goldsby, Taggart, & Ablin, 2006; Golenbiewski, Eudy, Clowse, & Allen, 2019; Hakeem et al., 2020; Hanna & Cruickshank, 1976; Hellwege, Torstenson, Edwards, & Velez Edwards, 2019; Hodel & Meier-Ruge, 1966; Hoversten et al., 2018; Hsu, Lin, & Chu, 2017; Iavazzo, Papakiritsis, & Gkegkes, 2016; Jain, Shetty, Juneja, & Narwal, 2016; Jankowska, 2011; Kaiserling, Ruck, & Xiao, 1995; C. J. Kim et al., 2008; S. J. Kim et al., 2013; Kravchenko, Akushevich, Abernethy, & Lyerly, 2012; LeRoith, 2011; Lester, Dysko, & Nemzek, 2004; Magden, Quackenbush, & VandeWoude, 2011; Magro et al., 2015; McManus, BoAbbas, Lampe, Holliday, & Shum, 2000; Michelet et al., 2008; Molina, Rabian, D'Agay, & Modai, 1992; Morra et al., 2011; Morris et al., 2014; Oh et al., 2010; Osaku et al., 2001; Othman, 2012; Ouchi et al., 2020; Owen & Reilly, 2018; Paraskevas, Veith, & Mikhailidis, 2019; Polascik, 2008; Rawls, Tompkins, Figueroa, & Melnick, 1968; Rial, Choi, Nguyen, Snyder, & Slepian, 2012; Rosen et al., 2012; Simkova, Kharaishvili, Korinkova, et al., 2016; Simkova, Kharaishvili, Slabakova, Murray, & Bouchal, 2016; Siracka, Durkovsky, Jancina, & Revesz, 1973; Strohle, Zanker, & Hahn, 2010; Sugimoto, Wong, Hirayama, Soga, & Tomita, 2010; Sunga, Eberl, Oeffinger, Hudson, & Mahoney, 2005; Y. Suzuki, Hisada, Hiraki, & Ando, 1974; Tweeddale, Scott, Fields, Roddick, & Ball, 1968; van Westreenen et al., 2005; Walker & Reed, 1973; Weng, Zeng, Wang, Liu, & He, 2017; Woodford, Jackson-Boeters, Darling, Shimizu, & Daley, 2013; W. K. Wu et al., 2014; Yacoub, Krishnan, Acevedo, Halliday, & Greene, 2015; Y. Yang, Tang, & Wei, 2013; M. Zhang, Zhu, & Davie, 2015; H. Zou et al., 2014) | 74 |
| No mention of UC | ("Abstracts of the 11th Congress of the European College for the Study of Vulval Disease," 2016; Adjei Boakye et al., 2018; Aida, Takebayashi, & Matsui, 1987; Al-Muzian, Almuzian, Mohammed, Ulhaq, & Keightley, 2021; A. Amini, Masoumi Moghaddam, Morris, & Pourgholami, 2012; Aoki & Matsubara, 2013; Asada, 1974; Asahina, Shirai, Horita, & Saito, 2012; Bateman, Sun, Hood, Flint, & Conrads, 2010; Bauer et al., 2012; Bercaw, Sanchez, Byrd, Bhattacharjee, & Dietrich, 2010; Boussemart et al., 2014; Buckley, 2011, 2012; Bullon & Navarro, 2017; Cho et al., 2014; Clifford et al., 2008; Cullen et al., 2009; Dupuis & Coard, 2009; Etokebe et al., 2009; Fernandez-Medarde & Santos, 2011; Gabaldon & Haro-Gonzalez-Vico, 2019; Gillison et al., 2014; Goldsby, Taggart, & Ablin, 2006; Golenbiewski, Eudy, Clowse, & Allen, 2019; Hakeem et al., 2020; Hanna & Cruickshank, 1976; Hellwege, Torstenson, Edwards, & Velez Edwards, 2019; Hodel & Meier-Ruge, 1966; Hoversten et al., 2018; Hsu, Lin, & Chu, 2017; Iavazzo, Papakiritsis, & Gkegkes, 2016; Jain, Shetty, Juneja, & Narwal, 2016; Jankowska, 2011; Kaiserling, Ruck, & Xiao, 1995; C. J. Kim et al., 2008; S. J. Kim et al., 2013; Kravchenko, Akushevich, Abernethy, & Lyerly, 2012; LeRoith, 2011; Lester, Dysko, & Nemzek, 2004; Magden, Quackenbush, & VandeWoude, 2011; Magro et al., 2015; McManus, BoAbbas, Lampe, Holliday, & Shum, 2000; Michelet et al., 2008; Molina, Rabian, D'Agay, & Modai, 1992; Morra et al., 2011; Morris et al., 2014; Oh et al., 2010; Osaku et al., 2001; Othman, 2012; Ouchi et al., 2020; Owen & Reilly, 2018; Paraskevas, Veith, & Mikhailidis, 2019; Polascik, 2008; Rawls, Tompkins, Figueroa, & Melnick, 1968; Rial, Choi, Nguyen, Snyder, & Slepian, 2012; Rosen et al., 2012; Simkova, Kharaishvili, Korinkova, et al., 2016; Simkova, Kharaishvili, Slabakova, Murray, & Bouchal, 2016; Siracka, Durkovsky, Jancina, & Revesz, 1973; Strohle, Zanker, & Hahn, 2010; Sugimoto, Wong, Hirayama, Soga, & Tomita, 2010; Sunga, Eberl, Oeffinger, Hudson, & Mahoney, 2005; Y. Suzuki, Hisada, Hiraki, & Ando, 1974; Tweeddale, Scott, Fields, Roddick, & Ball, 1968; van Westreenen et al., 2005; Walker & Reed, 1973; Weng, Zeng, Wang, Liu, & He, 2017; Woodford, Jackson-Boeters, Darling, Shimizu, & Daley, 2013; W. K. Wu et al., 2014; Yacoub, Krishnan, Acevedo, Halliday, & Greene, 2015; Y. Yang, Tang, & Wei, 2013; M. Zhang, Zhu, & Davie, 2015; H. Zou et al., 2014) | 78 |
| Osteonecrosis | ("Abstracts of the 11th Congress of the European College for the Study of Vulval Disease," 2016; Adjei Boakye et al., 2018; Aida, Takebayashi, & Matsui, 1987; Al-Muzian, Almuzian, Mohammed, Ulhaq, & Keightley, 2021; A. Amini, Masoumi Moghaddam, Morris, & Pourgholami, 2012; Aoki & Matsubara, 2013; Asada, 1974; Asahina, Shirai, Horita, & Saito, 2012; Bateman, Sun, Hood, Flint, & Conrads, 2010; Bauer et al., 2012; Bercaw, Sanchez, Byrd, Bhattacharjee, & Dietrich, 2010; Boussemart et al., 2014; Buckley, 2011, 2012; Bullon & Navarro, 2017; Cho et al., 2014; Clifford et al., 2008; Cullen et al., 2009; Dupuis & Coard, 2009; Etokebe et al., 2009; Fernandez-Medarde & Santos, 2011; Gabaldon & Haro-Gonzalez-Vico, 2019; Gillison et al., 2014; Goldsby, Taggart, & Ablin, 2006; Golenbiewski, Eudy, Clowse, & Allen, 2019; Hakeem et al., 2020; Hanna & Cruickshank, 1976; Hellwege, Torstenson, Edwards, & Velez Edwards, 2019; Hodel & Meier-Ruge, 1966; Hoversten et al., 2018; Hsu, Lin, & Chu, 2017; Iavazzo, Papakiritsis, & Gkegkes, 2016; Jain, Shetty, Juneja, & Narwal, 2016; Jankowska, 2011; Kaiserling, Ruck, & Xiao, 1995; C. J. Kim et al., 2008; S. J. Kim et al., 2013; Kravchenko, Akushevich, Abernethy, & Lyerly, 2012; LeRoith, 2011; Lester, Dysko, & Nemzek, 2004; Magden, Quackenbush, & VandeWoude, 2011; Magro et al., 2015; McManus, BoAbbas, Lampe, Holliday, & Shum, 2000; Michelet et al., 2008; Molina, Rabian, D'Agay, & Modai, 1992; Morra et al., 2011; Morris et al., 2014; Oh et al., 2010; Osaku et al., 2001; Othman, 2012; Ouchi et al., 2020; Owen & Reilly, 2018; Paraskevas, Veith, & Mikhailidis, 2019; Polascik, 2008; Rawls, Tompkins, Figueroa, & Melnick, 1968; Rial, Choi, Nguyen, Snyder, & Slepian, 2012; Rosen et al., 2012; Simkova, Kharaishvili, Korinkova, et al., 2016; Simkova, Kharaishvili, Slabakova, Murray, & Bouchal, 2016; Siracka, Durkovsky, Jancina, & Revesz, 1973; Strohle, Zanker, & Hahn, 2010; Sugimoto, Wong, Hirayama, Soga, & Tomita, 2010; Sunga, Eberl, Oeffinger, Hudson, & Mahoney, 2005; Y. Suzuki, Hisada, Hiraki, & Ando, 1974; Tweeddale, Scott, Fields, Roddick, & Ball, 1968; van Westreenen et al., 2005; Walker & Reed, 1973; Weng, Zeng, Wang, Liu, & He, 2017; Woodford, Jackson-Boeters, Darling, Shimizu, & Daley, 2013; W. K. Wu et al., 2014; Yacoub, Krishnan, Acevedo, Halliday, & Greene, 2015; Y. Yang, Tang, & Wei, 2013; M. Zhang, Zhu, & Davie, 2015; H. Zou et al., 2014) | 49 |
| Other disease | (Almouhawis, Leao, Fedele, & Porter, 2013; S. K. Arora, Gupta, Nijhawan, & Mandal, 2010; Ban, Nemeth, Szauter, Soos, & Balasko, 2018; Boras et al., 2019; Brix, Nassau, Patterson, Cousar, & Wick, 2010; Brook, 2011; Buysschaert, Medina, Bergman, Shah, & Lonier, 2015; Cameselle-Teijeiro et al., 2015; Carneiro-Leão, Fernandes, Carvalho, Lopes, & Koch, 2021; Carvalho et al., 2007; Catanzaro, Andrien, Labbé, & Nevessignsky, 2009; Cuddy et al., 2012; Derrick & Neill, 2010; Djalilian & Nussenblatt, 2002; Finch, 2010; Finer, 2015; Franks & Atabaki-Pasdar, 2017; Giurdanella, Nijenhuis, Diercks, Jonkman, & Pas, 2018; Hansen & Dahle, 2012; Hardy, Shepherd, Donnelly, McKee, & Morrison, 2012; Humphreys, Blodgett, & Roberts, 2015; Jain, Saini, & Kohli, 2013; Kokubo & Iwashima, 2015; Konishi, 2017; R. S. Liu, Liu, Bu, & Dong, 2000; Martini & Zulian, 2006; McCully, 2017; Micali et al., 2005; Morgan, Hannon, & Lakhoo, 2011; Ndukwe, Fatusi, & Ugboko, 2002; Owens et al., 2008; Pang, Gao, Liao, & Ying, 2020; Panopalis & Clarke, 2006; Pathak, Sonalika, Hs, & Tegginammani, 2017; Pavone et al., 2015; Rawat, Singh, & Chaubey, 2017; Rezazadeh et al., 2018; V. Singh, Gupta, Khatana, Bhagol, & Gupta, 2014; Sleiwah, Thomas, Crawford, & Stanek, 2017; Thway, Fisher, & Sebire, 2012; Tomeno et al., 2020; Toubi, Kessel, Bamberger, & Golan, 2004; Traish, Miner, Morgentaler, & Zitzmann, 2011; Velangi, 2016; Ventura et al., 2020; Verhulst, Loos, Gerdes, & Teeuw, 2019; Wei, Kawachi, Okereke, & Mukamal, 2016; Weyer, Dunlap, & Shah, 2016; Wilson & Plavsic, 2012; Wolach et al., 2008; S. Wu et al., 2021; J. Yang, Wise, & Fukuchi, 2020; M. S. Yoon et al., 2004; Zupin et al., 2015) | 54 |
| The subject of studies are tobacco /betel | (Akl et al., 2010; Aslam, Saleem, German, & Qureshi, 2014; "Betel-quid and areca-nut chewing," 2004; Ebbert, Elrashidi, & Stead, 2015; Elton-Marshall, Wijesingha, Kennedy, & Hammond, 2018; Neki, 2011; Shirley, Kaner, & Glesby, 2013; Siasos et al., 2014; Wald & Hackshaw, 1996; Waziry, Jawad, Ballout, Al Akel, & Akl, 2017) | 10 |
| The subject of studies are transplantation | (Alonso-Pulpon, Segovia, Gomez-Bueno, & Garcia-Pavia, 2012; Baez et al., 2013; Baez et al., 2016; Baughman et al., 2012; Bharat, 2019; Boratynska, Watorek, Smolska, Patrzalek, & Klinger, 2007; Braun, 2003; Bulut et al., 2005; Cezario et al., 2008; Cota, Oliveira, Costa, Cortelli, & Costa, 2008; Crane, Eltemamy, & Shoskes, 2019; Dave, Bahadur, Bhat, & Shah, 2015; De Braekeleer et al., 2010; de Oliveira Costa, Diniz Ferreira, de Miranda Cota, da Costa, & Aguiar, 2006; Gaber et al., 2008; Ghaninejad et al., 2009; Greco et al., 2005; Grimm et al., 2006; Hummel & Hetzer, 2004; Kitamura et al., 2019; Kuypers et al., 2005; Luk, 2004; M. Neuberger, 2003; H. Mizuno et al., 2017; S. Mizuno et al., 2018; S. Mizuno et al., 2017; Mourad et al., 2007; Murphy & Vikram, 2019; Oguz et al., 2000; Osiak et al., 2018; Osiak, Wychowanski, Grzeszczyk, Durlik, & Fiedor, 2020; Ostronoff et al., 2007; Pereira-Lopes et al., 2013; Popat, 2010; Quellmann et al., 2008; Shaqman, Ioannidou, Burleson, Hull, & Dongari-Bagtzoglou, 2010; Shen & Wu, 2007; S. Singh & Watt, 2012; V. K. Singh, Means, Pham, & Kloecker, 2016; Trevillian, 2006; Wakasugi, Uchida, & Uno, 2018; Q. Wang et al., 2009; Watson & Dark, 2012; Weimer et al., 2006; Wisanuyotin & Jiravuttipong, 2009; Wong & Pagalilauan, 2015) | 46 |
| Other studies no mention of PD and UC | ("17th International Congress of the Hungarian Society for Microbiology, Budapest, Hungary, July 8-10, 2015 Abstracts," 2015; "29th Annual Scientific Meeting of the Tucuman Biology Association, Tucuman, Argentina, October 17-19, 2012 Abstracts," 2013; Aalaei-Andabili, Fabbri, & Rezaei, 2013; Al-Qattan & Al-Qattan, 2018; Albers, Vuletic, & Cheung, 2012; Amais, Donati, & Zezzi Arruda, 2020; Amin et al., 2021; A. A. Amini & Nair, 2011; "Annual Meeting of the American College of Epidemiology Silver Spring, MD, USA September 7-9, 2014 Abstracts," 2014; Behera, Praharaj, Dehury, & Negi, 2015; Berdowska, 2004; Bonnet, Garnero, & Ferrari, 2016; Chey & Buchanan, 2008; "CME questions on the basic science issue," 2016; Czap, 2002; da Silva, 2019; Dang, Walker, Ford, & Valentine, 2014; Editors, 2013; "European Congress of Epidemiology: "Healthy Living", Maastricht, Netherlands, June 25-27, 2015 Abstracts," 2015; "Graphical abstract TOC," 2020; Han, 2013; Hart, 2016; He & Li, 2013; Ho et al., 2016; Hodge et al., 2014; Ishigaki et al., 2020; Ji et al., 2012; Kempe & Mader, 2012; Kennedy, 2004; S. R. Kim et al., 2013; Klammt et al., 2011; Kohli et al., 2021; Krauss et al., 2011; La Starza et al., 2015; Larsen, Minaya, Vaish, & Pena, 2018; Laurindo, Pescatore, & Fernandes Dde, 2012; Lei, Nowbar, Mariash, & Ingbar, 2003; Lewis & Illum, 2010; Licciardi et al., 2012; Lu, Tseng, Lee, Li, & Wang, 2010; Macey et al., 2020; Malathi, Mythili, & Vasanthi, 2014; Mali, 2014; Marais et al., 2006; Margadant, Kreft, Zambruno, & Sonnenberg, 2013; Massey, 2005; Mata, Renaud, & Mollereau, 2019; Melero, Grimaldi, Perez-Gracia, & Ascierto, 2013; Nicolatou-Galitis, 2018; Niklander, Bordagaray, Fernandez, & Hernandez, 2021; Noreen et al., 2012; Noren Hooten et al., 2020; Ojeda, Moreno-Guzman, Gonzalez-Cortes, Yanez-Sedeno, & Pingarron, 2014; Pascoal, Estevinho, Choupina, Sousa-Pimenta, & Estevinho, 2019; Perricone & Perricone, 2014; Philibert & Cattran, 2008; Pockley, Henderson, & Multhoff, 2014; N. Powell, Canavan, MacDonald, & Lord, 2010; Reece, 2009; Reigstad, Varhaug, & Lillehaug, 2005; Rojas, Oliva, & Santos, 2011; Saito et al., 2014; Satarug, Garrett, Sens, & Sens, 2010; Sato et al., 2012; Scholer, Langer, & Kuchenbauer, 2011; M. Sharma, Astekar, Soi, Manjunatha, & Shetty, 2015; Sigusch, 2013; J. A. Singh, Hossain, Mudano, et al., 2017; J. A. Singh, Hossain, Tanjong Ghogomu, et al., 2017; T. Singh & Newman, 2011; Siozopoulou & Vanhoenacker, 2020; Stipp, Tully, Murphy, Kahn, Helyar, Sellers, Warner, Morris, Taylor 3rd, et al., 2002; Stipp, Tully, Murphy, Kahn, Helyar, Sellers, Warner, Morris, Taylor, et al., 2002; Swinkels et al., 2011; Taguchi & Murakami, 2013; Tamai, Sugiyama, & Kiyoura, 2011; Techanukul et al., 2011; Temtem & Santos, 2012; Threadgill, 2015; Toyokuni & Akatsuka, 2007; Traber & Stevens, 2011; "Translational Science 2012 Meeting Abstracts," 2012; Tripathy, 2008; Tsuda et al., 2007; Tunuguntla, 2005; Vos et al., 2017; Vos et al., 2015; Wada et al., 2011; X. Wang, Chen, & Yuan, 2020; Weber et al., 2012; Wolf, Hill, & Slate, 2010; "Women and screening. Preventing, detecting common health problems," 2007; Xu et al., 2017) | 93 |
| **From 2021-04-01 to 2022-05-17** | | |
| **Reasons** | | No. of  studies |
| Animal studies | | 3 |
| Bacteria studies | | 7 |
| cancer therapy studies | | 14 |
| Case report | | 8 |
| Other disease therapy | | 8 |
| Drug efficacy and route of administration | | 0 |
| Cancer metastasis | | 4 |
| No direct relationship between PD and UC | | 3 |
| No mention of PD | | 7 |
| No mention of UC | | 11 |
| Osteonecrosis | | 7 |
| Other disease | | 8 |
| The subject of studies is tobacco /betel | | 1 |
| The subject of studies is transplantation | | 1 |
| Other studies no mention of PD and UC | | 20 |
| **Total** | | 102 |
|  | |  |
| Animal studies | (Ray et al., 2021; Rosa et al., 2021; Shiga et al., 2021) | 3 |
| Bacteria studies | (Dong, Li, Xiao, Cui, & Fan, 2022; Kato, Zhang, & Sun, 2022; Rahman, Lamarca, Hubner, Valle, & McNamara, 2021; Sharma et al., 2021; Sobocki et al., 2022; Varsha, Maheshwari, & Nampoothiri, 2021; Yuan et al., 2021) | 7 |
| cancer therapy studies | (Ala & Ala, 2021; Chen, Chen, Zhu, & Zhuang, 2021; Choueiri et al., 2021; Fujiwara et al., 2021; Irimia et al., 2021; Kia, Basirat, Saedi, & Arab, 2021; Kim et al., 2021; Kondo et al., 2021; Ladak, Sandhu, & Itrat, 2021; Lan, Liu, & Guo, 2021; Mou et al., 2022; Ruan & Han, 2021; Shimizu et al., 2022; Wang, Shan, & Shen, 2021) | 14 |
| Case report | (Carneiro-Leão, Fernandes, Carvalho, Lopes, & Koch, 2021; Jazdarehee, Huget-Penner, & Pawlowska, 2022; Jha & Chandi, 2021; Ngu, Tse, Chu, Ngan, & Chan, 2021; Schoenaker et al., 2021; Sowińska-Przepiera et al., 2021; Yamamoto, Wada, Ito, Kawase, & Tamura, 2022; Younan, Raad, Sawan, & Said, 2021) | 8 |
| Other disease therapy | (Akatsuka et al., 2021; Badmanaban et al., 2021; Hunt et al., 2022; Khader et al., 2021; Khaliq, Chobisa, Richard, Swinney, & Yeo, 2021; Kwon et al., 2021; Zhang, Zhao, & Zheng, 2021; Zhou et al., 2021) | 8 |
| Drug efficacy and route of administration |  | 0 |
| Cancer metastasis | (Fang, Yang, Li, Zhang, & Huang, 2021; Takehara et al., 2021; Wang, Shen, & Shan, 2021; Xu & Lu, 2021) | 4 |
| No direct relationship between PD and UC | (C. Fang et al., 2021; Malta et al., 2022; Wu, Sodji, & Oyelere, 2022) | 3 |
| No mention of PD | (Duijster, Franz, Neefjes, & Mughini-Gras, 2021; Z. Fang et al., 2021; Gondhowiardjo et al., 2021; Moazen et al., 2022; Özkur et al., 2021; Radaic et al., 2021; Spodzieja & Olczak-Kowalczyk, 2022) | 7 |
| No mention of UC | (Basilicata et al., 2022; P. Chen et al., 2021; Ferrillo et al., 2021; Fischer et al., 2021; Gonzalez-Moles & Ramos-Garcia, 2021; Mao et al., 2022; Meurman & Bascones-Martinez, 2021; Ngoude et al., 2021; Strait, Barnes, & Smith, 2021; Vernerova et al., 2021; Zhuang et al., 2021) | 11 |
| Osteonecrosis | (Avishai, Muchnik, Masri, Zlotogorski-Hurvitz, & Chaushu, 2022; Barbosa, Almeida Paz, & Braga, 2021; Buchbender, Bauerschmitz, Pirkl, Kesting, & Schmitt, 2022; Caldas, Antunes, Pegoraro, Guedes, & Santos, 2021; Hasegawa et al., 2021; Ueda et al., 2021; Wei et al., 2021) | 7 |
| Other disease | (Hsiao, Lee, Ho, Tseng, & Wang, 2021; Jung et al., 2022; Kadkhoda & Ghafouri-Fard, 2022; Lee et al., 2021; Luo et al., 2021; Prasad, Black, Zhu, Sharma, & Jacobe, 2021; Toyohiro et al., 2021; Watanabe et al., 2021) | 8 |
| The subject of studies is tobacco /betel | (Popa et al., 2021) | 1 |
| The subject of studies is transplantation | (Moest et al., 2021) | 1 |
| Other studies no mention of PD and UC | ("Abstract of the 47th National Conference of Association of Clinical Biochemists of India," 2021; Almawash, Osman, Mustafa, & El Hamd, 2022; Aubeux et al., 2021; Baltanás, García-Navas, & Santos, 2021; Carrieri et al., 2021; "CONTENTS," 2021; Das, Benko, Gill, & Dufour, 2021; Dziewas et al., 2021; Guti, Baidya, Banerjee, Adhikari, & Jha, 2021; Iglesias-Lopez, Obach, Vallano, & Agustí, 2021; Jovicic, 2021; Kehm, Baldensperger, Raupbach, & Höhn, 2021; Kulmann-Leal, Ellwanger, & Chies, 2021; Nazia et al., 2021; Parthasarathy, Menon, & Devaranavadagi, 2021; Russell et al., 2021; Tawde et al., 2021; W. Wang et al., 2021; Wen et al., 2021; Zheng et al., 2021) | 20 |

**references**

17th International Congress of the Hungarian Society for Microbiology, Budapest, Hungary, July 8-10, 2015 Abstracts. (2015). Acta Microbiologica Et Immunologica Hungarica, 62, 127-241.

29th Annual Scientific Meeting of the Tucuman Biology Association, Tucuman, Argentina, October 17-19, 2012 Abstracts. (2013). Biocell, 37(2), A37-A76.

39th Annual Conference of the American Society for Laser Medicine and Surgery, Inc. (2019). Lasers in Surgery and Medicine, 51.

A Loudon, J. (2013). Preventing and Correcting Communicable and Non-Communicable Chronic Disease via Amlexanox – Dual ‘No-Nonsense’ and Inflammatory Axis Targeting. Journal of Bioanalysis & Biomedicine, 05(05), 138-179. doi:10.4172/1948-593x.1000095

Aalaei-Andabili, S. H., Fabbri, M., & Rezaei, N. (2013). Reciprocal effects of Toll-like receptors and miRNAs on biological processes in human health and disease: a systematic review. Immunotherapy, 5(10), 1127-1142. doi:10.2217/imt.13.112

Abdolkarimi, B., Zareifar, S., Karimi, M., Salajegheh, P., Fazl, S., & Fathpour, G. (2017). How to treat extramedullary acute lymphoblastic leukemia. Iranian Journal of Medical Sciences, 42(3), 90.

Abdullah, B. H., Jabbar Abdul Qader, O. A., & Mussedi, O. S. (2016). Retrospective analysis of 1286 oral and maxillofacial biopsied lesions of Iraqi children over a 30 years period. Pediatric Dental Journal, 26(1), 16-20. doi:10.1016/j.pdj.2015.10.003

Abed, J., Maalouf, N., Parhi, L., Chaushu, S., Mandelboim, O., & Bachrach, G. (2017). Tumor Targeting by Fusobacterium nucleatum: A Pilot Study and Future Perspectives. Front Cell Infect Microbiol, 7, 295. doi:10.3389/fcimb.2017.00295

Abiko, Y., Saitoh, M., Nishimura, M., Yamazaki, M., Sawamura, D., & Kaku, T. (2007). Role of beta-defensins in oral epithelial health and disease. Med Mol Morphol, 40(4), 179-184. doi:10.1007/s00795-007-0381-8

Abstracts of the 11th Congress of the European College for the Study of Vulval Disease. (2016). Journal of Lower Genital Tract Disease, 20(4).

Adil, M., Iqbal, W., Adnan, F., Wazir, S., Khan, I., Khayam, M. U., . . . Khan, I. N. (2018). Association of Metronidazole with Cancer: A Potential Risk Factor or Inconsistent Deductions? Curr Drug Metab, 19(11), 902-909. doi:10.2174/1389200219666180329124130

Adjei Boakye, E., Buchanan, P., Hinyard, L., Osazuwa-Peters, N., Schootman, M., & Piccirillo, J. F. (2018). Incidence and Risk of Second Primary Malignant Neoplasm After a First Head and Neck Squamous Cell Carcinoma. JAMA Otolaryngol Head Neck Surg, 144(8), 727-737. doi:10.1001/jamaoto.2018.0993

Adornato, M. C., Morcos, I., & Rozanski, J. (2007). The treatment of bisphosphonate-associated osteonecrosis of the jaws with bone resection and autologous platelet-derived growth factors. J Am Dent Assoc, 138(7), 971-977. doi:10.14219/jada.archive.2007.0294

Aghajanian, C., Goff, B., Nycum, L. R., Wang, Y. V., Husain, A., & Blank, S. V. (2015). Final overall survival and safety analysis of OCEANS, a phase 3 trial of chemotherapy with or without bevacizumab in patients with platinum-sensitive recurrent ovarian cancer. Gynecol Oncol, 139(1), 10-16. doi:10.1016/j.ygyno.2015.08.004

Agnihotry, A., Thompson, W., Fedorowicz, Z., van Zuuren, E. J., & Sprakel, J. (2019). Antibiotic use for irreversible pulpitis. Cochrane Database Syst Rev, 5(5), CD004969. doi:10.1002/14651858.CD004969.pub5

Agosti, S. J., Espinoza, C. G., & Ramirez, G. (1989). Granulomatous myocarditis with unusual histologic features. South Med J, 82(9), 1180-1183. doi:10.1097/00007611-198909000-00032

Agrillo, A., Nastro Siniscalchi, E., Facchini, A., Filiaci, F., & Ungari, C. (2012). Osteonecrosis of the jaws in patients assuming bisphosphonates and sunitinib: two case reports. Eur Rev Med Pharmacol Sci, 16(7), 952-957.

Ahmad Akhoundi, M. S., Rokn, A., Bagheri, R., Momeni, N., & Hodjat, M. (2018). Urokinase-plasminogen activator protects periodontal ligament fibroblast from oxidative induced-apoptosis and DNA damage. J Periodontal Res, 53(5), 861-869. doi:10.1111/jre.12576

Ahuja, T. S. (2003). Doxycycline decreases proteinuria in glomerulonephritis. Am J Kidney Dis, 42(2), 376-380. doi:10.1016/s0272-6386(03)00662-0

Ai, L., Xu, Z., Yang, B., He, Q., & Luo, P. (2019). Sorafenib-associated hand-foot skin reaction: practical advice on diagnosis, mechanism, prevention, and management. Expert Rev Clin Pharmacol, 12(12), 1121-1127. doi:10.1080/17512433.2019.1689122

Aida, T., Takebayashi, S., & Matsui, K. (1987). [Brown tumor originating in the bladder wall and not accumulating 123I-MIBG]. Rinsho Hoshasen, 32(6), 715-716.

Aird, J. (2003). Controlled Release--SMi Conference. 12-13 February 2003, London,UK. IDrugs, 6(4), 334-336.

Akahane, M., Matsumoto, S., Kanagawa, Y., Mitoma, C., Uchi, H., Yoshimura, T., . . . Imamura, T. (2018). Long-Term Health Effects of PCBs and Related Compounds: A Comparative Analysis of Patients Suffering from Yusho and the General Population. Arch Environ Contam Toxicol, 74(2), 203-217. doi:10.1007/s00244-017-0486-6

Akl, E. A., Gaddam, S., Gunukula, S. K., Honeine, R., Jaoude, P. A., & Irani, J. (2010). The effects of waterpipe tobacco smoking on health outcomes: a systematic review. Int J Epidemiol, 39(3), 834-857. doi:10.1093/ije/dyq002

Al-Muzian, L., Almuzian, M., Mohammed, H., Ulhaq, A., & Keightley, A. J. (2021). Are developmentally missing teeth a predictive risk marker of malignant diseases in non-syndromic individuals? A systematic review. J Orthod, 1465312520984166. doi:10.1177/1465312520984166

Al-Qattan, M. M., & Al-Qattan, A. M. (2018). Fibromodulin: Structure, Physiological Functions, and an Emphasis on its Potential Clinical Applications in Various Diseases. J Coll Physicians Surg Pak, 28(10), 783-790. doi:3022

Alam, G., Singh, M. P., & Singh, A. (2011). Wound healing potential of some medicinal plants. International Journal of Pharmaceutical Sciences Review and Research, 9(1), 136-145.

Albers, J. J., Vuletic, S., & Cheung, M. C. (2012). Role of plasma phospholipid transfer protein in lipid and lipoprotein metabolism. Biochim Biophys Acta, 1821(3), 345-357. doi:10.1016/j.bbalip.2011.06.013

Albrecht, M., Kupfer, R., Reissmann, D. R., Muhlhauser, I., & Kopke, S. (2016). Oral health educational interventions for nursing home staff and residents. Cochrane Database Syst Rev, 9(9), CD010535. doi:10.1002/14651858.CD010535.pub2

Alexandrescu, D. T., Dasanu, C. A., & Kauffman, C. L. (2009). Acute scurvy during treatment with interleukin-2. Clin Exp Dermatol, 34(7), 811-814. doi:10.1111/j.1365-2230.2008.03052.x

Ali, R. A., & Mohamed, K. E. (2016). Metastatic Clear Cell Renal Cell Carcinoma Presenting with a Gingival Metastasis. Clin Pract, 6(2), 847. doi:10.4081/cp.2016.847

Alifrangis, C., Evans, R., Williams, J., & Seckl, M. J. (2011). An unusual gum lesion with a positive pregnancy test. Bmj, 343, d5009. doi:10.1136/bmj.d5009

Allison, S. D. (2008). Effect of structural relaxation on the preparation and drug release behavior of poly(lactic-co-glycolic)acid microparticle drug delivery systems. J Pharm Sci, 97(6), 2022-2035. doi:10.1002/jps.21124

Allon, I., Pessing, A., Kaplan, I., Allon, D. M., & Hirshberg, A. (2014). Metastatic tumors to the gingiva and the presence of teeth as a contributing factor: a literature analysis. J Periodontol, 85(1), 132-139. doi:10.1902/jop.2013.130118

Almazyad, A., Woo, S. B., & Villa, A. (2019). Persistent Pain and Gingival Swelling in a Middle-aged Woman. JAMA Otolaryngol Head Neck Surg, 145(7), 676-677. doi:10.1001/jamaoto.2019.0606

Almeida, H. L., Jr., Heckler, G. T., Fong, K., Lai-Cheong, J., & McGrath, J. (2013). Sporadic Kindler syndrome with a novel mutation. An Bras Dermatol, 88(6 Suppl 1), 212-215. doi:10.1590/abd1806-4841.20132173

Almouhawis, H. A., Leao, J. C., Fedele, S., & Porter, S. R. (2013). Wegener's granulomatosis: a review of clinical features and an update in diagnosis and treatment. J Oral Pathol Med, 42(7), 507-516. doi:10.1111/jop.12030

Alonso-Pulpon, L., Segovia, J., Gomez-Bueno, M., & Garcia-Pavia, P. (2012). Heart transplantation: organisational aspects and current trends in immunosuppression--a view from Spain. Heart, 98(11), 878-889. doi:10.1136/heartjnl-2011-300479

Alrumaih, R. A., Arian, A. A., Alhedyani, A. A., Al-Zaher, N., & Dababo, M. A. (2015). Hepatocellular carcinoma first presenting as a tumor of the oral cavity. Hematol Oncol Stem Cell Ther, 8(3), 130-135. doi:10.1016/j.hemonc.2015.03.001

Altintas, A., Vardar, M. A., Aridogan, N., Doran, F., & Tuncer, I. (1995). Choriocarcinoma metastatic to the maxillary gingiva. Eur J Surg Oncol, 21(5), 579-580. doi:10.1016/s0748-7983(95)97712-0

Alves, F., Prado, J. D., & Rocha, A. C. (2007). Clinical Features and Management of Jaw Osteonecrosis in Patients Receiving Bisphosphonate Therapy. Blood, 110(11), 4775-4775. doi:10.1182/blood.V110.11.4775.4775

Amais, R. S., Donati, G. L., & Zezzi Arruda, M. A. (2020). ICP-MS and trace element analysis as tools for better understanding medical conditions. TrAC Trends in Analytical Chemistry, 133. doi:10.1016/j.trac.2020.116094

Amatya, S., Park, E. J., Park, J. H., Kim, J. S., Seol, E., Lee, H., . . . Na, D. H. (2013). Drug release testing methods of polymeric particulate drug formulations. Journal of Pharmaceutical Investigation, 43(4), 259-266. doi:10.1007/s40005-013-0072-5

American Diabetes, A. (2015). (3) Initial evaluation and diabetes management planning. Diabetes Care, 38 Suppl, S17-19. doi:10.2337/dc15-S006

Amin, M., Tang, S., Shalamanova, L., Taylor, R. L., Wylie, S., Abdullah, B. M., & Whitehead, K. A. (2021). Polyamine biomarkers as indicators of human disease. Biomarkers, 26(2), 77-94. doi:10.1080/1354750X.2021.1875506

Amini, A., Masoumi Moghaddam, S., Morris, D. L., & Pourgholami, M. H. (2012). The critical role of vascular endothelial growth factor in tumor angiogenesis. Curr Cancer Drug Targets, 12(1), 23-43. doi:10.2174/156800912798888956

Amini, A. A., & Nair, L. S. (2011). Lactoferrin: a biologically active molecule for bone regeneration. Curr Med Chem, 18(8), 1220-1229. doi:10.2174/092986711795029744

Amjadi, O., Mousavi, T., Rafiei, A., Afzali, M. A., Yousefpour, M., & Ghaemi, A. (2016). Therapeutic and nutritional effects of pomegranate from the perspective of islamic texts, traditional and modern medicine. Journal of Mazandaran University of Medical Sciences, 25(134), 374-393.

Ammari, A. B., & Fung, D. E. (2007). Case report: Denys- Drash syndrome. Eur Arch Paediatr Dent, 8(4), 219-223. doi:10.1007/BF03262601

Anderson, D. A., Woltman, M. L., Kovach, G., & Konety, B. R. (2004). Long-term treatment of metastatic renal-cell carcinoma with fluorouracil. Lancet Oncol, 5(11), 690-692. doi:10.1016/S1470-2045(04)01611-0

Anderson, H. C., Mulhall, D., & Garimella, R. (2010). Role of extracellular membrane vesicles in the pathogenesis of various diseases, including cancer, renal diseases, atherosclerosis, and arthritis. Lab Invest, 90(11), 1549-1557. doi:10.1038/labinvest.2010.152

Anderson, K., & Eskander, R. N. (2018). Immune Checkpoint Inhibition in the Treatment of Gynecologic Cancer. Current Obstetrics and Gynecology Reports, 7(1), 6-19. doi:10.1007/s13669-018-0231-9

Andrukhov, O., Matejka, M., & Rausch-Fan, X. (2010). Effect of cyclosporin A on proliferation and differentiation of human periodontal ligament cells. Acta Odontol Scand, 68(6), 329-334. doi:10.3109/00016357.2010.514717

Ángel Descalzo, M. (2007). Registro Español de Acontecimientos Adversos de Terapias Biológicas en Enfermedades Reumáticas (BIOBADASER): informe de la situación, 26 de enero de 2006. Reumatologia Clinica, 3(1), 4-20. doi:10.1016/s1699-258x(07)73593-3

Angiero, F., & Stefani, M. (2008). Metastatic embryonal carcinoma in the maxillary gingiva. Anticancer Res, 28(2B), 1181-1186.

Annual Meeting of the American College of Epidemiology Silver Spring, MD, USA September 7-9, 2014 Abstracts. (2014). Annals of Epidemiology, 24(9), 682-702.

Antoniu, S. A. (2013). Discontinued drugs for pulmonary, allergy, gastrointestinal, arthritis (2012). Expert Opin Investig Drugs, 22(11), 1453-1464. doi:10.1517/13543784.2013.836489

Aoki, Y., & Matsubara, Y. (2013). Ras/MAPK syndromes and childhood hemato-oncological diseases. Int J Hematol, 97(1), 30-36. doi:10.1007/s12185-012-1239-y

Aoun, G., Hayek, E., & Nasseh, I. (2020). Mandibular Metastasis of a Recurrent Poorly Differentiated Urothelial Bladder Carcinoma. J Clin Imaging Sci, 10(1), 27. doi:10.25259/JCIS_46_2020

Apolo, A. B., Karzai, F. H., Trepel, J. B., Alarcon, S., Lee, S., Lee, M. J., . . . Dahut, W. L. (2017). A Phase II Clinical Trial of TRC105 (Anti-Endoglin Antibody) in Adults With Advanced/Metastatic Urothelial Carcinoma. Clin Genitourin Cancer, 15(1), 77-85. doi:10.1016/j.clgc.2016.05.010

Arora, A., Khattri, S., Ismail, N. M., Kumbargere Nagraj, S., & Eachempati, P. (2019). School dental screening programmes for oral health. Cochrane Database Syst Rev, 8(8), CD012595. doi:10.1002/14651858.CD012595.pub3

Arora, S. K., Gupta, N., Nijhawan, R., & Mandal, A. K. (2010). Epithelioid cell granulomas in urine cytology smears: Same cause, different implications. Diagn Cytopathol, 38(10), 765-767. doi:10.1002/dc.21327

Arruebo, M., Vilaboa, N., & Santamaria, J. (2010). Drug delivery from internally implanted biomedical devices used in traumatology and in orthopedic surgery. Expert Opin Drug Deliv, 7(5), 589-603. doi:10.1517/17425241003671544

Arweiler, N. B., Pergola, G., Kuenz, J., Hellwig, E., Sculean, A., & Auschill, T. M. (2011). Clinical and antibacterial effect of an anti-inflammatory toothpaste formulation with Scutellaria baicalensis extract on experimental gingivitis. Clin Oral Investig, 15(6), 909-913. doi:10.1007/s00784-010-0471-1

Asada, T. (1974). Treatment of human cancer with mumps virus. Cancer, 34(6), 1907-1928. doi:10.1002/1097-0142(197412)34:6<1907::aid-cncr2820340609>3.0.co;2-4

Asahina, A., Shirai, A., Horita, A., & Saito, I. (2012). Annular elastolytic giant cell granuloma associated with prostate carcinoma: demonstration of human metalloelastase (MMP-12) expression. Clin Exp Dermatol, 37(1), 70-72. doi:10.1111/j.1365-2230.2011.04110.x

Asherson, R. A., Espinosa, G., Menahem, S., Yinh, J., Bucciarelli, S., Bosch, X., & Cervera, R. (2008). Relapsing catastrophic antiphospholipid syndrome: report of three cases. Semin Arthritis Rheum, 37(6), 366-372. doi:10.1016/j.semarthrit.2007.08.001

Ashrafi, F., Derakhshandeh, A., Movahedian, B., & Moghaddas, A. (2017). Osteonecrosis of the Jaws in Patient Received Bisphosphonates and Sunitinib Separately: A Case Report. J Res Pharm Pract, 6(3), 182-185. doi:10.4103/jrpp.JRPP_17_36

Aslam, H. M., Saleem, S., German, S., & Qureshi, W. A. (2014). Harmful effects of shisha: literature review. Int Arch Med, 7(1), 16. doi:10.1186/1755-7682-7-16

Asmane, I., Kurtz, J. E., Bajard, A., Guastalla, J. P., Meeus, P., Tredan, O., . . . Ray-Coquard, I. (2011). Bevacizumab plus microtubule targeting agents in heavily pre-treated ovarian cancer patients: a retrospective study. Bull Cancer, 98(9), 80-89. doi:10.1684/bdc.2011.1436

Assaf, A. T., Smeets, R., Riecke, B., Weise, E., Grobe, A., Blessmann, M., . . . Gerhards, F. (2013). Incidence of bisphosphonate-related osteonecrosis of the jaw in consideration of primary diseases and concomitant therapies. Anticancer Res, 33(9), 3917-3924.

Aswath, N., Balakrishnan, C., Shyamsundar, V., & Thirrugnanamurthy, S. (2017). Oral gingival metastasis: A diagnostic dilemma. Indian J Dent Res, 28(3), 344-347. doi:10.4103/ijdr.IJDR_261_16

Ataie-Kachoie, P., Pourgholami, M. H., & Morris, D. L. (2013). Inhibition of the IL-6 signaling pathway: a strategy to combat chronic inflammatory diseases and cancer. Cytokine Growth Factor Rev, 24(2), 163-173. doi:10.1016/j.cytogfr.2012.09.001

Atkinson, T. J. (2008). Toll-like receptors, transduction-effector pathways, and disease diversity: evidence of an immunobiological paradigm explaining all human illness? Int Rev Immunol, 27(4), 255-281. doi:10.1080/08830180801959072

Aug, P., Bhatti, S., Al-Tawil, S., Sani, N., & Ibrahim, Z. (2016). A rare presentation of prostate adenocarcinoma. BJU International, 118, 37.

Aydın, A. A., Alparslan, A. Ş., Ayık, H., Kayıkçıoğlu, E., & Öztürk, B. (2018). A challenging palliation via novel therapeutic approach for unusual presenting with gingival metastasis from renal cell carcinoma relapsed eight years after nephrectomy. The European Research Journal, 4(4), 416-420. doi:10.18621/eurj.348318

Azuma, M. M., Gomes-Filho, J. E., Prieto, A. K. C., Samuel, R. O., de Lima, V. M. F., Sumida, D. H., . . . Cintra, L. T. A. (2017). Diabetes increases interleukin-17 levels in periapical, hepatic, and renal tissues in rats. Arch Oral Biol, 83, 230-235. doi:10.1016/j.archoralbio.2017.08.001

Bachmann, G., Crosby, U., Feldman, R. A., Ronkin, S., & Constantine, G. D. (2011). Effects of bazedoxifene in nonflushing postmenopausal women: a randomized phase 2 trial. Menopause, 18(5), 508-514. doi:10.1097/gme.0b013e3181fa358b

Badros, A. Z., Philip, S., Lesho, P., Weikel, D., Goloubeva, O., Sadowska, M., . . . Meiller, T. (2012). Prospective Observational Study of 110 Multiple Myeloma (MM) Patients On Monthly Versus 3 Monthly Infusion of Zoledronic Acid. Blood, 120(21), 4245-4245. doi:10.1182/blood.V120.21.4245.4245

Baez, C. F., Guimaraes, M. A., Martins, R. A., Zalona, A. C., Cossatis, J. J., Zalis, M. G., . . . Varella, R. B. (2013). Detection of Merkel cell polyomavirus in oral samples of renal transplant recipients without Merkel cell carcinoma. J Med Virol, 85(11), 2016-2019. doi:10.1002/jmv.23687

Baez, C. F., Savassi-Ribas, F., Rocha, W. M., Almeida, S. G., Goncalves, M. T., Guimaraes, M. A., . . . Varella, R. B. (2016). Association of Epstein-Barr Virus (Ebv) but Not Human Papillomavirus (Hpv) with Gingivitis and/or Periodontitis in Transplanted Individuals. Rev Inst Med Trop Sao Paulo, 58, 58. doi:10.1590/S1678-9946201658058

Bakeen, G., Hiyarat, A. M., & Al-Ubaidy, S. S. (1976). Chorioepithelioma presenting as a bleeding gingival mass. Oral Surg Oral Med Oral Pathol, 41(4), 467-471. doi:10.1016/0030-4220(76)90274-7

Baldini, S., Fambrini, M., Carrai, V., Rigacci, L., Alterini, R., & Bosi, A. (2012). Romiplostim overcomes refractory secondary immune thrombocytopenia in a patient affected by serous ovarian carcinoma. International Cancer Conference Journal, 1(4), 239-242. doi:10.1007/s13691-012-0049-7

Balle, C., Esra, R., Havyarimana, E., Jaumdally, S. Z., Lennard, K., Konstantinus, I. N., . . . Jaspan, H. B. (2020). Relationship between the Oral and Vaginal Microbiota of South African Adolescents with High Prevalence of Bacterial Vaginosis. Microorganisms, 8(7). doi:10.3390/microorganisms8071004

Ban, A., Nemeth, Z. F., Szauter, A., Soos, S., & Balasko, M. (2018). [Prevalence and severity of chronic parodontitis and oral mucosal lesions in chronic obstructive lung disease]. Orv Hetil, 159(21), 831-836. doi:10.1556/650.2018.31037

Basi, D. L., Hughes, P. J., Thumbigere-Math, V., Sabino, M., Mariash, A., Lunos, S. A., . . . Gopalakrishnan, R. (2011). Matrix metalloproteinase-9 expression in alveolar extraction sockets of Zoledronic acid-treated rats. J Oral Maxillofac Surg, 69(11), 2698-2707. doi:10.1016/j.joms.2011.02.065

Basile, S., Pinelli, S., Bottone, P., Artini, P. G., Plotti, F., & Panici, P. B. (2019). 'Primary gingival and later primary vulval carcinomas arising in lichen planus: report of a case and clinical suggestions for diagnosis of a neglected disease'. Gynecol Endocrinol, 35(11), 938-940. doi:10.1080/09513590.2019.1617265

Bateman, N. W., Sun, M., Hood, B. L., Flint, M. S., & Conrads, T. P. (2010). Defining central themes in breast cancer biology by differential proteomics: conserved regulation of cell spreading and focal adhesion kinase. J Proteome Res, 9(10), 5311-5324. doi:10.1021/pr100580e

Bathi, R. J., Kumar, Y. P., & Natarajan, K. (2002). Cowden's syndrome: a case report. Quintessence Int, 33(1), 75-80.

Bauer, J. S., Beck, N., Kiefer, J., Stockmann, P., Wichmann, M., & Eitner, S. (2012). Awareness and education of patients receiving bisphosphonates. J Craniomaxillofac Surg, 40(3), 277-282. doi:10.1016/j.jcms.2011.04.011

Baughman, R. P., Meyer, K. C., Nathanson, I., Angel, L., Bhorade, S. M., Chan, K. M., . . . Whelan, T. (2012). Monitoring of nonsteroidal immunosuppressive drugs in patients with lung disease and lung transplant recipients: American College of Chest Physicians evidence-based clinical practice guidelines. Chest, 142(5), e1S-e111S. doi:10.1378/chest.12-1044

Baumgartner, J. C., & Eggli, D. (1989). The use of bone imaging to detect a periapical lesion of endodontic origin. J Endod, 15(10), 493-495. doi:10.1016/s0099-2399(89)80032-9

Beastall, G., & Rainbow, S. (2008). Vitamin D reinvented: implications for clinical chemistry. Clin Chem, 54(4), 630-632. doi:10.1373/clinchem.2007.101618

Becerik, S., Celec, P., Gurkan, A., Ozturk, V. O., Kamodyova, N., Atilla, G., & Emingil, G. (2016). Gingival Crevicular Fluid and Plasma Levels of Transglutaminase-2 and Oxidative Stress Markers in Cyclosporin A-Induced Gingival Overgrowth. J Periodontol, 87(12), 1508-1516. doi:10.1902/jop.2016.160031

Beeley, N. R. A., Ansell, P. R. J., & Docherty, A. J. P. (1994). Inhibitors of matrix metalloproteinases (MMP's). Current Opinion in Therapeutic Patents, 4(1), 7-16.

Beenken, A., & Mohammadi, M. (2009). The FGF family: biology, pathophysiology and therapy. Nat Rev Drug Discov, 8(3), 235-253. doi:10.1038/nrd2792

Behera, S. K., Praharaj, A. B., Dehury, B., & Negi, S. (2015). Exploring the role and diversity of mucins in health and disease with special insight into non-communicable diseases. Glycoconj J, 32(8), 575-613. doi:10.1007/s10719-015-9606-6

Bektas, M., Samanci, N. S., Cokgezer, S., Keskin, D., & Demirelli, F. H. (2019). A severe case of bevacizumab-induced thrombotic microangiopathy. J Oncol Pharm Pract, 25(7), 1754-1757. doi:10.1177/1078155218800371

Belden, H. (2003). Here's a novel delivery for new testosterone product. Drug Topics, 147(1).

Beltramini, G. A., Massarelli, O., Demarchi, M., Copelli, C., Cassoni, A., Valentini, V., . . . Baj, A. (2012). Is neck dissection needed in squamous-cell carcinoma of the maxillary gingiva, alveolus, and hard palate? A multicentre Italian study of 65 cases and literature review. Oral Oncol, 48(2), 97-101. doi:10.1016/j.oraloncology.2011.08.012

Belvisi, M. G., & Bottomley, K. M. (2003). The role of matrix metalloproteinases (MMPs) in the pathophysiology of chronic obstructive pulmonary disease (COPD): a therapeutic role for inhibitors of MMPs? Inflamm Res, 52(3), 95-100. doi:10.1007/s000110300020

Ben Ayed, M., el Mezni, F., Letourneau, A., & Najeh, H. (1987). [Granular cell tumor. Apropos of 3 cases]. Tunis Med, 65(5), 353-358.

Bennardo, F., Buffone, C., Muraca, D., Antonelli, A., & Giudice, A. (2020). Medication-Related Osteonecrosis of the Jaw with Spontaneous Hemimaxilla Exfoliation: Report of a Case in Metastatic Renal Cancer Patient under Multidrug Therapy. Case Rep Med, 2020, 8093293. doi:10.1155/2020/8093293

Bercaw, J. L., Sanchez, J., Byrd, R. H., Bhattacharjee, M. B., & Dietrich, J. E. (2010). Sex cord tumor with annular tubules in a young adolescent with Von Willebrand's disease. J Pediatr Adolesc Gynecol, 23(3), e111-114. doi:10.1016/j.jpag.2009.09.006

Berdowska, I. (2004). Cysteine proteases as disease markers. Clin Chim Acta, 342(1-2), 41-69. doi:10.1016/j.cccn.2003.12.016

Berends, A. M., Olgers, T. J., & Ter Maaten, J. C. (2016). A Woman With a Purpuric Rash and Weakness. J Emerg Med, 50(5), e239-240. doi:10.1016/j.jemermed.2016.02.003

Berger, J. R., & Houff, S. (2009). Opportunistic infections and other risks with newer multiple sclerosis therapies. Ann Neurol, 65(4), 367-377. doi:10.1002/ana.21630

Bermudez, M., Imaz-Rosshandler, I., Rangel-Escareno, C., Zeichner-David, M., Arzate, H., & Mercado-Celis, G. E. (2015). CEMP1 Induces Transformation in Human Gingival Fibroblasts. PLoS One, 10(5), e0127286. doi:10.1371/journal.pone.0127286

Berth-Jones, J. (2005). The use of ciclosporin in psoriasis. J Dermatolog Treat, 16(5-6), 258-277. doi:10.1080/09546630500423914

Best, C. M., Xu, J., Patchen, B. K., & Cassano, P. A. (2019). Vitamin D supplementation in pregnant or breastfeeding women or young children for preventing asthma. Cochrane Database of Systematic Reviews(8). doi:10.1002/14651858.Cd013396

Betel-quid and areca-nut chewing. (2004). IARC Monographs on the Evaluation of Carcinogenic Risks to Humans, 85, 41-278.

Beth-Tasdogan, N. H., Mayer, B., Hussein, H., & Zolk, O. (2017). Interventions for managing medication-related osteonecrosis of the jaw. Cochrane Database Syst Rev, 10(10), CD012432. doi:10.1002/14651858.CD012432.pub2

Beyaz, L., Atasever, A., Apaydin, N., & Deniz, K. (2009). Metastases from a gingival Squamous Cell Carcinoma (SCC) in a dog. Revue De Medecine Veterinaire, 160(2), 74-78.

Bezinelli, L. M., Eduardo, F. P., Migliorati, C. A., Ferreira, M. H., Taranto, P., Sales, D. B., . . . Buzaid, A. C. (2019). A Severe, Refractory Case of Mucous Membrane Pemphigoid After Treatment With Pembrolizumab: Brief Communication. J Immunother, 42(9), 359-362. doi:10.1097/CJI.0000000000000280

Bharat, A. (2019). A Need for Targeted Immunosuppression after Lung Transplantation. Am J Respir Cell Mol Biol, 61(3), 279-280. doi:10.1165/rcmb.2019-0100ED

Bhatia, K., Vaid, A. K., Rawal, S., & Patole, K. D. (2007). Pure choriocarcinoma of testis with rare gingival and skin metastases. Singapore Med J, 48(3), e77-80.

Biesecker, L. G., Edwards, M., O'Donnell, S., Doherty, P., MacDougall, T., Tith, K., . . . Schwartz, B. (2020). Clinical report: one year of treatment of Proteus syndrome with miransertib (ARQ 092). Cold Spring Harb Mol Case Stud, 6(1). doi:10.1101/mcs.a004549

Bochenek, G., Hasler, R., El Mokhtari, N. E., Konig, I. R., Loos, B. G., Jepsen, S., . . . Schaefer, A. S. (2013). The large non-coding RNA ANRIL, which is associated with atherosclerosis, periodontitis and several forms of cancer, regulates ADIPOR1, VAMP3 and C11ORF10. Hum Mol Genet, 22(22), 4516-4527. doi:10.1093/hmg/ddt299

Boddu, P., Falchi, L., Hosing, C., Newberry, K., Bose, P., & Verstovsek, S. (2017). The role of thrombocytapheresis in the contemporary management of hyperthrombocytosis in myeloproliferative neoplasms: A case-based review. Leuk Res, 58, 14-22. doi:10.1016/j.leukres.2017.03.008

Bodnar, L., Gornas, M., & Szczylik, C. (2011). Sorafenib as a third line therapy in patients with epithelial ovarian cancer or primary peritoneal cancer: a phase II study. Gynecol Oncol, 123(1), 33-36. doi:10.1016/j.ygyno.2011.06.019

Bonnet, N., Garnero, P., & Ferrari, S. (2016). Periostin action in bone. Mol Cell Endocrinol, 432(C), 75-82. doi:10.1016/j.mce.2015.12.014

Boras, V. V., Skrinjar, I., Vuletic, L. B., Bradamante, M., Bartenjev, I., & Hadzavdic, S. L. (2019). Oral and Vulvar Lichen Sclerosus. Acta Dermatovenerologica Croatica, 27(3), 195-197.

Boratynska, M., Watorek, E., Smolska, D., Patrzalek, D., & Klinger, M. (2007). Anticancer effect of sirolimus in renal allograft recipients with de novo malignancies. Transplant Proc, 39(9), 2736-2739. doi:10.1016/j.transproceed.2007.08.078

Borchard, K. L., & Orchard, D. (2008). Systemic therapy of paediatric atopic dermatitis: an update. Australas J Dermatol, 49(3), 123-134; quiz 135-126. doi:10.1111/j.1440-0960.2008.00451_1.x

Boussemart, L., Girault, I., Mateus, C., Thomas, M., Routier, E., Cazenave, H., . . . Robert, C. (2014). BRAF inhibitors induce skin and extra-cutaneous tumors via paradoxical activation of the MAPK pathway: Molecular study of 66 tumors and visualization of BRAF/CRAF protein dimers. Cancer Research, 74(19). doi:10.1158/1538-7445.AM2014-934

Bowles, L., Depala, A., & Beski, S. (2013). Gynaecological problems in women with severe inherited platelet disorders-a single centre experience. Journal of Thrombosis and Haemostasis, 11, 1095-1096.

Braun, W. E. (2003). Renal transplantation: basic concepts and evolution of therapy. J Clin Apher, 18(3), 141-152. doi:10.1002/jca.10070

Brazzell, J. L., & Borjesson, D. L. (2006). Intra-abdominal mass aspirate from an alopecic dog. Vet Clin Pathol, 35(2), 259-262. doi:10.1111/j.1939-165x.2006.tb00127.x

Brindha, S., Vincent, S., Velmurugan, D., Ananthakrishnan, D., Sundaramurthi, J. C., & Gnanadoss, J. J. (2017). Bioinformatics approach to prioritize known drugs towards repurposing for tuberculosis. Med Hypotheses, 103, 39-45. doi:10.1016/j.mehy.2017.04.005

Brix, W. K., Nassau, S. R., Patterson, J. W., Cousar, J. B., & Wick, M. R. (2010). Idiopathic lymphoplasmacellular mucositis-dermatitis. J Cutan Pathol, 37(4), 426-431. doi:10.1111/j.1600-0560.2009.01371.x

Brocheriou, C., & Laufer, J. (1979). [Buccal metastases from soft-tissue cancers. A report on 3 cases (author's transl)]. Rev Stomatol Chir Maxillofac, 80(5), 275-279.

Brook, I. (2011). Abscesses from actinomyces infection: why so difficult to diagnose? Expert Rev Anti Infect Ther, 9(12), 1097-1099. doi:10.1586/eri.11.142

Broussalis, E., Trinka, E., Kraus, J., McCoy, M., & Killer, M. (2013). Treatment strategies for vasculitis that affects the nervous system. Drug Discov Today, 18(17-18), 818-835. doi:10.1016/j.drudis.2013.05.009

Brunello, A., Saia, G., Bedogni, A., Scaglione, D., & Basso, U. (2009). Worsening of osteonecrosis of the jaw during treatment with sunitinib in a patient with metastatic renal cell carcinoma. Bone, 44(1), 173-175. doi:10.1016/j.bone.2008.08.132

Brusca, M. I., & Brusca, L. (2011). Influencia de las drogas para tratamiento prostatico en la portacion de Candida spp. en pacientes periodontales. Mycoses, 54, 65-66.

Buchner, A., & Begleiter, A. (1980). Metastatic renal cell carcinoma in the gingiva mimicking a hyperplastic lesion. Case report. J Periodontol, 51(7), 413-415. doi:10.1902/jop.1980.51.7.413

Buckley, K. S. (2011). Pediatric genitourinary tumors. Curr Opin Oncol, 23(3), 297-302. doi:10.1097/CCO.0b013e3283458613

Buckley, K. S. (2012). Pediatric genitourinary tumors. Curr Opin Oncol, 24(3), 291-296. doi:10.1097/CCO.0b013e32835265c9

Buczko, P., Zalewska, A., & Szarmach, I. (2015). Saliva and Oxidative Stress in Oral Cavity and in Some Systemic Disorders. Journal of Physiology and Pharmacology, 66(1), 3-9.

Bulbul, B., & Kucukguzel, I. (2019). Microsomal Prostaglandin E2 Synthase-1 as a New Macromolecular Drug Target in the Prevention of Inflammation and Cancer. Anticancer Agents Med Chem, 19(10), 1205-1222. doi:10.2174/1871520619666190227174137

Bullon, P., & Navarro, J. M. (2017). Inflammasome as a Key Pathogenic Mechanism in Endometriosis. Curr Drug Targets, 18(9), 997-1002. doi:10.2174/1389450117666160709013850

Bulut, S., Ozdemir, B. H., Alaaddinoglu, E. E., Oduncuoglu, F. B., Bulut, O. E., & Demirhan, B. (2005). Effect of cyclosporin A on apoptosis and expression of p53 and bcl-2 proteins in the gingiva of renal transplant patients. J Periodontol, 76(5), 691-695. doi:10.1902/jop.2005.76.5.691

Burden, D., Mullally, B., & Sandler, J. (2001). Orthodontic treatment of patients with medical disorders. Eur J Orthod, 23(4), 363-372. doi:10.1093/ejo/23.4.363

Burris, H., Stephenson, J., Otterson, G. A., Stein, M., McGreivy, J., Sun, Y. N., . . . Schwartzberg, L. S. (2011). Safety and pharmacokinetics of motesanib in combination with panitumumab and gemcitabine-Cisplatin in patients with advanced cancer. J Oncol, 2011, 853931. doi:10.1155/2011/853931

Burrowes, J. D., & Van Houten, G. (2005). Use of alternative medicine by patients with stage 5 chronic kidney disease. Adv Chronic Kidney Dis, 12(3), 312-325. doi:10.1016/j.ackd.2005.04.001

Burtness, B. (2017). Treatment de-intensification strategies for head and neck cancer. Clinical cancer research, 23(23). doi:10.1158/1557-3265.AACRAHNS17-IA17

Busk, M., Munk, O. L., Jakobsen, S., Wang, T., Skals, M., Steiniche, T., . . . Overgaard, J. (2010). Assessing hypoxia in animal tumor models based on pharmocokinetic analysis of dynamic FAZA PET. Acta Oncol, 49(7), 922-933. doi:10.3109/0284186X.2010.503970

Button, T., & Coles, A. J. (2010). Alemtuzumab for the treatment of multiple sclerosis. Future Neurology, 5(2), 177-188. doi:10.2217/fnl.09.81

Buysschaert, M., Medina, J. L., Bergman, M., Shah, A., & Lonier, J. (2015). Prediabetes and associated disorders. Endocrine, 48(2), 371-393. doi:10.1007/s12020-014-0436-2

Cameselle-Teijeiro, J., Fachal, C., Cabezas-Agricola, J. M., Alfonsin-Barreiro, N., Abdulkader, I., Vega-Gliemmo, A., & Hermo, J. A. (2015). Thyroid Pathology Findings in Cowden Syndrome: A Clue for the Diagnosis of the PTEN Hamartoma Tumor Syndrome. Am J Clin Pathol, 144(2), 322-328. doi:10.1309/AJCP84INGJUVTBME

Cao, Y., Qiao, M., Tian, Z., Yu, Y., Xu, B., Lao, W., . . . Li, W. (2018). Comparative Analyses of Subgingival Microbiome in Chronic Periodontitis Patients with and Without IgA Nephropathy by High Throughput 16S rRNA Sequencing. Cell Physiol Biochem, 47(2), 774-783. doi:10.1159/000490029

Carmagnola, D., Celestino, S., & Abati, S. (2008). Dental and periodontal history of oncologic patients on parenteral bisphosphonates with or without osteonecrosis of the jaws: a pilot study. Oral Surg Oral Med Oral Pathol Oral Radiol Endod, 106(6), e10-15. doi:10.1016/j.tripleo.2008.07.011

Carneiro-Leão, D., Fernandes, S., Carvalho, M., Lopes, M., & Koch, C. (2021). Abstract. Haemophilia, 27(S2), 18-181. doi:10.1111/hae.14236

Cartron, G., de Guibert, S., Dilhuydy, M. S., Morschhauser, F., Leblond, V., Dupuis, J., . . . Hallek, M. (2014). Obinutuzumab (GA101) in relapsed/refractory chronic lymphocytic leukemia: final data from the phase 1/2 GAUGUIN study. Blood, 124(14), 2196-2202. doi:10.1182/blood-2014-07-586610

Carvalho, E. M., Nascimento, M. C., Castro, N., Muniz, A. L. A., de Jesus, A. R., Porto, A. F., . . . Santos, S. B. (2007). Immunological response and proviral load as factors influencing disease expression in HTLV-1. American Journal of Tropical Medicine and Hygiene, 77(5), 80-80.

Caskey, M. F. (2006). Clinical manifestations associated with HTLV-I infection: A cross-sectional study. American Journal of Tropical Medicine and Hygiene, 75(5), 197-198.

Cassoni, A., Terenzi, V., Bartoli, D., Rajabtork Zadeh, O., Battisti, A., Pagnoni, M., . . . Valentini, V. (2014). Metastatic uterine leiomyosarcoma in the upper buccal gingiva misdiagnosed as an epulis. Case Rep Oncol Med, 2014, 402342. doi:10.1155/2014/402342

Catanzaro, D., Andrien, M., Labbé, M., & Nevessignsky, M. T. (2009). Abstracts of the 23rd European Immunogenetics and Histocompatibility Conference. Ulm, Germany. May 9-12, 2009. Tissue Antigens, 73(5), 385-523. doi:10.1111/j.1399-0039.2009.01255.x

Cengiz, M. I., Bagci, H., Cengiz, S., Yigit, S., & Cengiz, K. (2009). Periodontal disease in patients with familial Mediterranean fever: from inflammation to amyloidosis. J Periodontal Res, 44(3), 354-361. doi:10.1111/j.1600-0765.2008.01115.x

Cezario, E. S., Cota, L. O., Ferreira, S. D., Siqueira, F. M., Soares, R. V., Zenobio, E. G., & Costa, F. O. (2008). Gingival overgrowth in renal transplant subjects medicated with tacrolimus in the absence of calcium channel blockers. Transplantation, 85(2), 232-236. doi:10.1097/TP.0b013e3181604fad

Chai, K. L., Valk, S. J., Piechotta, V., Kimber, C., Monsef, I., Doree, C., . . . Skoetz, N. (2020). Convalescent plasma or hyperimmune immunoglobulin for people with COVID-19: a living systematic review. Cochrane Database Syst Rev, 10(10), CD013600. doi:10.1002/14651858.CD013600.pub3

Chakravarthy, U., Harding, S. P., Rogers, C. A., Downes, S., Lotery, A. J., Dakin, H. A., . . . Reeves, B. C. (2015). A randomised controlled trial to assess the clinical effectiveness and cost-effectiveness of alternative treatments to Inhibit VEGF in Age-related choroidal Neovascularisation (IVAN). Health Technol Assess, 19(78), 1-298. doi:10.3310/hta19780

Chandler, M. (2014). Probiotics - not all created equally. J Small Anim Pract, 55(9), 439-441. doi:10.1111/jsap.12263

Chang, Y., Woo, H. G., Park, J., Lee, J. S., & Song, T. J. (2020). Improved oral hygiene care is associated with decreased risk of occurrence for atrial fibrillation and heart failure: A nationwide population-based cohort study. Eur J Prev Cardiol, 27(17), 1835-1845. doi:10.1177/2047487319886018

Charles, T. (2018). 12th International HHT Scientific Conference : June 8-11, 2017, Dubrovnik, Croatia. Angiogenesis, 21(1), 111-167. doi:10.1007/s10456-017-9584-3

Chase, K., Lawler, D. F., McGill, L. D., Miller, S., Nielsen, M., & Lark, K. G. (2011). Age relationships of postmortem observations in Portuguese Water Dogs. Age (Dordr), 33(3), 461-473. doi:10.1007/s11357-010-9181-5

Chatelain, D., Parc, Y., Christin-Maitre, S., Parc, R., & Flejou, J. F. (2002). Mixed ductal-pancreatic polypeptide-cell carcinoma of the pancreas. Histopathology, 41(2), 122-126. doi:10.1046/j.1365-2559.2002.01447.x

Chaudhary, P. P., Conway, P. L., & Schlundt, J. (2018). Methanogens in humans: potentially beneficial or harmful for health. Appl Microbiol Biotechnol, 102(7), 3095-3104. doi:10.1007/s00253-018-8871-2

Chaurasiya, S., & Mishra, V. (2018). Biodegradable nanoparticles as theranostics of ovarian cancer: an overview. J Pharm Pharmacol, 70(4), 435-449. doi:10.1111/jphp.12860

Chee, B., Park, B., Fitzsimmons, T., Coates, A. M., & Bartold, P. M. (2016). Omega-3 fatty acids as an adjunct for periodontal therapy-a review. Clin Oral Investig, 20(5), 879-894. doi:10.1007/s00784-016-1750-2

Chen, B., Li, W. T., & Wang, F. I. (2018). A blastema-predominant canine renal nephroblastoma with gingival metastasis: case report and literature review. J Vet Diagn Invest, 30(3), 430-437. doi:10.1177/1040638718762560

Chen, J., Bookbinder, M., Ryan, M. E., Golub, L. M., Ashley, R., & Ramamurthy, N. S. (2001). Biodistribution of radiolabeled [(3)H] CMT-3 in rats. Curr Med Chem, 8(3), 253-256. doi:10.2174/0929867013373615

Chen, J., Domingue, J. C., & Sears, C. L. (2017). Microbiota dysbiosis in select human cancers: Evidence of association and causality. Semin Immunol, 32(C), 25-34. doi:10.1016/j.smim.2017.08.001

Chen, J., Quan, M., Chen, Z., Zeng, T., Li, Y., Zhou, Y., . . . Gao, Y. (2020). Camrelizumab in advanced or metastatic solid tumour patients with DNA mismatch repair deficient or microsatellite instability high: an open-label prospective pivotal trial. J Cancer Res Clin Oncol, 146(10), 2651-2657. doi:10.1007/s00432-020-03251-5

Chen, J., Wei, W., Zheng, L., Li, H., Feng, Y., Wan, T., . . . Zhang, Y. (2020). 841P Phase II study of anlotinib plus pemetrexed for platinum-resistant epithelial ovarian cancer. Annals of Oncology, 31, S630-S631. doi:10.1016/j.annonc.2020.08.980

Chey, H., & Buchanan, S. (2008). Toxins in everyday life. Prim Care, 35(4), 707-727. doi:10.1016/j.pop.2008.07.001

Chien, M. H., Lin, C. W., Cheng, C. W., Wen, Y. C., & Yang, S. F. (2013). Matrix metalloproteinase-2 as a target for head and neck cancer therapy. Expert Opin Ther Targets, 17(2), 203-216. doi:10.1517/14728222.2013.740012

Chiu, H. Y., & Tsai, T. F. (2011). Topical use of systemic drugs in dermatology: a comprehensive review. J Am Acad Dermatol, 65(5), 1048 e1041-1022. doi:10.1016/j.jaad.2010.08.034

Cho, E. J., Lee, H. J., Park, K. U., Kim, U. Y., Lee, H. J., & Bang, S. M. (2014). Immune thrombocytopenic purpura in a patient with renal cell carcinoma. Korean J Intern Med, 29(5), 671-674. doi:10.3904/kjim.2014.29.5.671

Choi, H. M., Han, K., Park, Y. G., & Park, J. B. (2016). Associations between the number of natural teeth and renal dysfunction. Medicine (Baltimore), 95(34), e4681. doi:10.1097/MD.0000000000004681

Chossegros, C., Blanc, J. L., Cheynet, F., Bataille, J. F., & Tessier, H. (1991). [Metastatic localization in the buccal cavity. Case report and literature review]. Rev Stomatol Chir Maxillofac, 92(3), 160-164.

Choueiri, T., Agarwal, N., Ho, T., Pal, S. K., Seon, B., Jivani, M., . . . Theuer, C. (2016). TRAXAR study: a randomized phase 2 trial of axitinib and TRC105 versus axitinib alone in patients with advanced or metastatic renal cell carcinoma (mRCC). Annals of Oncology, 27. doi:10.1093/annonc/mdw373.78

Choueiri, T., Michaelson, M. D., Posadas, E., Sonpavde, G., McDermott, D., Seon, B., . . . Theuer, C. (2016). A phase 1b dose-escalation study of TRC105 (endoglin antibody) in combination with axitinib in patients with metastatic renal cell carcinoma (mRCC). Annals of Oncology, 27. doi:10.1093/annonc/mdw373.32

Choueiri, T. K., Agarwal, N., Ho, T. H., PachynskI, R., Pal, S., Ryan, C. W., . . . Theuer, C. P. (2015). TRAXAR study: A randomized phase 2 trial of axitinib and TRC105 versus AXitinib alone in patients with advanced or metastatic renal cell carcinoma (mRCC). BJU International, 116, 5-6.

Choueiri, T. K., Michaelson, M. D., Posadas, E. M., Sonpavde, G. P., McDermott, D. F., Sean, B. K., . . . Theuer, C. P. (2015). A phase 1b dose-escalation study of TRC105 (anti-Endoglin Antibody) in combination with axitinib in patients with metastatic renal cell carcinoma (mRCC). BJU International, 116, 4-5.

Choueiri, T. K., Posadas, E. M., Sonpavde, G., Figlin, R. A., Walsh, M. K., Wall, K. C., . . . Theuer, C. P. (2014). A phase 1b dose-escalation study of TRC105 (anti-endoglin antibody) in combination with axitinib in patients with metastatic renal cell carcinoma (mRCC). Journal of Clinical Oncology, 32(15).

Chung, V., Mansfield, A. S., Braiteh, F., Richards, D., Durivage, H., Ungerleider, R. S., . . . Kovach, J. S. (2017). Safety, Tolerability, and Preliminary Activity of LB-100, an Inhibitor of Protein Phosphatase 2A, in Patients with Relapsed Solid Tumors: An Open-Label, Dose Escalation, First-in-Human, Phase I Trial. Clin Cancer Res, 23(13), 3277-3284. doi:10.1158/1078-0432.CCR-16-2299

Chura, J. C., Van Iseghem, K., Downs, L. S., Jr., Carson, L. F., & Judson, P. L. (2007). Bevacizumab plus cyclophosphamide in heavily pretreated patients with recurrent ovarian cancer. Gynecol Oncol, 107(2), 326-330. doi:10.1016/j.ygyno.2007.07.017

Clifford, N., Smith, L. M., Powell, J., Gattenlohner, S., Marx, A., & O'Connor, R. (2008). The EphA3 receptor is expressed in a subset of rhabdomyosarcoma cell lines and suppresses cell adhesion and migration. J Cell Biochem, 105(5), 1250-1259. doi:10.1002/jcb.21926

CME questions on the basic science issue. (2016). Orthopaedics and Trauma, 30(3), 279-280. doi:10.1016/j.mporth.2016.07.011

Cohen, D., Reif, J. S., Brodey, R. S., & Keiser, H. (1974). Epidemiological analysis of the most prevalent sites and types of canine neoplasia observed in a veterinary hospital. Cancer Res, 34(11), 2859-2868.

Colombo, P., Tondulli, L., Masci, G., Muzza, A., Rimassa, L., Petrella, D., & Santoro, A. (2005). Oral ulcer as an exclusive sign of gastric cancer: report of a rare case. Bmc Cancer, 5, 117. doi:10.1186/1471-2407-5-117

Colson, C. R., & De Broe, M. E. (2005). Kidney injury from alternative medicines. Adv Chronic Kidney Dis, 12(3), 261-275. doi:10.1016/j.ackd.2005.03.006

Conway de Macario, E., & Macario, A. J. (2009). Methanogenic archaea in health and disease: a novel paradigm of microbial pathogenesis. Int J Med Microbiol, 299(2), 99-108. doi:10.1016/j.ijmm.2008.06.011

Cook, S., Vermersch, P., Comi, G., Giovannoni, G., Rammohan, K., Rieckmann, P., . . . Group, C. S. (2011). Safety and tolerability of cladribine tablets in multiple sclerosis: the CLARITY (CLAdRIbine Tablets treating multiple sclerosis orallY) study. Mult Scler, 17(5), 578-593. doi:10.1177/1352458510391344

Cortes-Vazquez, Y. D., Cortes-Vazquez, A. R., Priego-Nino, A., Fernandez-Vivar, E., Silva-Bravo, F., & Gutierrez-Quiroz, C. (2020). Clear-cell carcinoma with sarcomatoid pattern, associated with gingival metastasis. Case report and review of the literature. Cir Cir, 88(Suppl 1), 54-58. doi:10.24875/CIRU.20001459

Cota, L. O., Oliveira, A. P., Costa, J. E., Cortelli, S. C., & Costa, F. O. (2008). Gingival status of Brazilian renal transplant recipients under sirolimus-based regimens. J Periodontol, 79(11), 2060-2068. doi:10.1902/jop.2008.080194

Cotruta, C., Trifu, V., Costache, D., Popescu, D., & Florea, C. (2010). Successful treatment of multiple facial angiofibromas with dermabrasion and CO2 laser. Archives of the Balkan Medical Union, 45(2), 169-171.

Crane, A., Eltemamy, M., & Shoskes, D. (2019). Transplant immunosuppressive drugs in urology. Transl Androl Urol, 8(2), 109-117. doi:10.21037/tau.2018.07.12

Crommelin, D. J. A., Mastrobattista, E., Hawe, A., Hoogendoorn, K. H., & Jiskoot, W. (2020). Shifting Paradigms Revisited: Biotechnology and the Pharmaceutical Sciences. J Pharm Sci, 109(1), 30-43. doi:10.1016/j.xphs.2019.08.010

Cuddy, K. K., Daley, T., Lapointe, H., Cobb, G., Jackson-Boeters, L., & Darling, M. (2012). Prostate Specific Antigen (Human Kallikrein Protein 3) Expression in Maxillofacial Cysts and Tumours. Journal of Oral and Maxillofacial Surgery, 70(9), e1-e2. doi:10.1016/j.joms.2012.07.009

Cudic, M., & Fields, G. B. (2009). Extracellular proteases as targets for drug development. Curr Protein Pept Sci, 10(4), 297-307. doi:10.2174/138920309788922207

Cullen, M., Seaman, S., Chaudhary, A., Yang, M. Y., Hilton, M. B., Logsdon, D., . . . St Croix, B. (2009). Host-derived tumor endothelial marker 8 promotes the growth of melanoma. Cancer Res, 69(15), 6021-6026. doi:10.1158/0008-5472.CAN-09-1086

Cummins, J., & Tangney, M. (2013). Bacteria and tumours: causative agents or opportunistic inhabitants? Infect Agent Cancer, 8(1), 11. doi:10.1186/1750-9378-8-11

Cunningham, G. R. (2006). Testosterone replacement therapy for late-onset hypogonadism. Nat Clin Pract Urol, 3(5), 260-267. doi:10.1038/ncpuro0479

Czap, A. (2002). The thunder of hooves in a tunnel. Altern Med Rev, 7(2), 93.

D'Silva, N. J., Summerlin, D. J., Cordell, K. G., Abdelsayed, R. A., Tomich, C. E., Hanks, C. T., . . . Meyrowitz, S. (2006). Metastatic tumors in the jaws: a retrospective study of 114 cases. J Am Dent Assoc, 137(12), 1667-1672. doi:10.14219/jada.archive.2006.0112

D'Souza, S. S., Faraj, J. A., & DeLuca, P. P. (2005). A model-dependent approach to correlate accelerated with real-time release from biodegradable microspheres. AAPS PharmSciTech, 6(4), E553-564. doi:10.1208/pt060470

da Silva, R. R. (2019). Investigating the specificity of peptidases: Scientific relevance and functional implications for cellular dynamics. J Cell Biochem, 120(4), 4800-4801. doi:10.1002/jcb.28095

Daltaban, O., Saygun, I., & Bolu, E. (2006). Periodontal status in men with hypergonadotropic hypogonadism: effects of testosterone deficiency. J Periodontol, 77(7), 1179-1183. doi:10.1902/jop.2006.050286

Dandona, P., & Rosenberg, M. T. (2010). A practical guide to male hypogonadism in the primary care setting. Int J Clin Pract, 64(6), 682-696. doi:10.1111/j.1742-1241.2010.02355.x

Dang, T. S., Walker, M., Ford, D., & Valentine, R. A. (2014). Nutrigenomics: the role of nutrients in gene expression. Periodontol 2000, 64(1), 154-160. doi:10.1111/prd.12001

Danon, O., Boukobza, B., Gombaud, D., Kazerouni, F., Laurette, F., & Bachmeyer, C. (1999a). Gingival metastasis disclosing a renal adenocarcinoma [1]. Presse Medicale, 28(27), 1466.

Danon, O., Boukobza, B., Gombaud, D., Kazerouni, F., Laurette, F., & Bachmeyer, C. (1999b). [Gingival metastasis revealing a renal adenocarcinoma]. Presse Med, 28(27), 1466.

Dave, K., Bahadur, M. M., Bhat, G. M., & Shah, S. (2015). Extramedullary plasmocytoma: A rare malignancy in renal transplant recipient. Indian Journal of Transplantation, 9(1), 30-33. doi:10.1016/j.ijt.2015.04.005

Davies, N. M., Longstreth, J., & Jamali, F. (2001). Misoprostol therapeutics revisited. Pharmacotherapy, 21(1), 60-73. doi:10.1592/phco.21.1.60.34442

De Braekeleer, E., Douet-Guilbert, N., Basinko, A., Le Bris, M. J., Morel, F., Berthou, C., . . . De Braekeleer, M. (2010). Distinct clonal anomalies involving RUNX1 in acute myeloid leukemia at diagnosis and after bone marrow transplantation. Ann Hematol, 89(12), 1277-1281. doi:10.1007/s00277-010-0937-x

de Courten, A., Irle, C., Samson, J., & Lombardi, T. (2001). Metastatic transitional cell carcinoma of the urinary bladder presenting as a mandibular gingival swelling. J Periodontol, 72(5), 688-690. doi:10.1902/jop.2001.72.5.688

de Oliveira Costa, F., Diniz Ferreira, S., de Miranda Cota, L. O., da Costa, J. E., & Aguiar, M. A. (2006). Prevalence, severity, and risk variables associated with gingival overgrowth in renal transplant subjects treated under tacrolimus or cyclosporin regimens. J Periodontol, 77(6), 969-975. doi:10.1902/jop.2006.050327

De Pasquale, M. D., Pessolano, R., Boldrini, R., Ilari, I., Donfrancesco, A., Cortesi, E., & Jenkner, A. (2011). Continuing response to subsequent treatment lines with tyrosine kinase inhibitors in an adolescent with metastatic renal cell carcinoma. J Pediatr Hematol Oncol, 33(5), e176-179. doi:10.1097/MPH.0b013e3182028fd9

de Pina-Neto, J. M., de Souza, N. V., Velludo, M. A., Perosa, G. B., de Freitas, M. M., & Colafemina, J. F. (1998). Retinal changes and tumorigenesis in Ramon syndrome: follow-up of a Brazilian family. Am J Med Genet, 77(1), 43-46.

Debarbieux, S., Perrot, J. L., Erfan, N., Ronger-Savle, S., Labeille, B., Cinotti, E., . . . Groupe d'Imagerie Cutanee Non Invasive de la Societe Francaise de, D. (2014). Reflectance confocal microscopy of mucosal pigmented macules: a review of 56 cases including 10 macular melanomas. Br J Dermatol, 170(6), 1276-1284. doi:10.1111/bjd.12803

Deluchi, P., Ledur, G. R., Pavarini, S. P., & Poppl, A. G. (2018). Granulosa Cell Tumor Associated with Occult Hyperadrenocorticism in a Yorkshire Terrier Bitch. Acta Scientiae Veterinariae, 46.

Derrick, E. K., & Neill, S. (2010). Vulval dermatoses. Medicine, 38(6), 300-305. doi:10.1016/j.mpmed.2010.03.005

Di Cerbo, A., Palmieri, B., Aponte, M., Morales-Medina, J. C., & Iannitti, T. (2016). Mechanisms and therapeutic effectiveness of lactobacilli. J Clin Pathol, 69(3), 187-203. doi:10.1136/jclinpath-2015-202976

Divya, K. S., Moran, N. A., & Atkin, P. A. (2010). Numb chin syndrome: a case series and discussion. Br Dent J, 208(4), 157-160. doi:10.1038/sj.bdj.2010.157

Djalilian, A. R., & Nussenblatt, R. B. (2002). Immunosuppression in uveitis. Ophthalmol Clin North Am, 15(3), 395-404, viii. doi:10.1016/s0896-1549(02)00036-6

Doolittle, J. M., & Webster-Cyriaque, J. (2014). Polymicrobial infection and bacterium-mediated epigenetic modification of DNA tumor viruses contribute to pathogenesis. mBio, 5(3), e01015-01014. doi:10.1128/mBio.01015-14

Dorman, G., Cseh, S., Hajdu, I., Barna, L., Konya, D., Kupai, K., . . . Ferdinandy, P. (2010). Matrix metalloproteinase inhibitors: a critical appraisal of design principles and proposed therapeutic utility. Drugs, 70(8), 949-964. doi:10.2165/11318390-000000000-00000

dos Santos, M. R. G., da Silva, J. H. S., & Caxito, M. L. C. (2015). Brief review on the medicinal uses and antimicrobial activity of different parts of Schinus terebinthifolius raddi. International Journal of Pharmacy and Pharmaceutical Sciences, 7(12), 1-7.

Doumas, K., Strepetis, K., Trikeriotis, D., Andriopoulos, N., Sapaziotis, D., & Lykourinas, M. (2000). Exophytic mass of the gingiva as initial manifestation of metastatic prostatic carcinoma. Journal of B.U.ON., 5(1), 103-104.

Doval, D. C., Naresh, K. N., Sabitha, K. S., Vijaykumar, M., Bapsy, P. P., Anantha, N., & Kumarswamy, S. V. (1994). Carcinoma of the urinary bladder metastatic to the oral cavity. Indian J Cancer, 31(1), 8-11.

Dove, A. (2002). MMP inhibitors: glimmers of hope amidst clinical failures. Nat Med, 8(2), 95. doi:10.1038/nm0202-95

Duan, X., Chen, X., Gupta, M., Seriwatanachai, D., Xue, H., Xiong, Q., . . . Yuan, Q. (2020). Salivary microbiome in patients undergoing hemodialysis and its associations with the duration of the dialysis. BMC Nephrol, 21(1), 414. doi:10.1186/s12882-020-02009-y

Dupuis, C., & Coard, K. C. M. (2009). A review of granular cell tumours at the University Hospital of the West Indies: 1965-2006. West Indian Medical Journal, 58(2), 138-141.

Dyer, T. A., Brocklehurst, P., Glenny, A. M., Davies, L., Tickle, M., Issac, A., & Robinson, P. G. (2014). Dental auxiliaries for dental care traditionally provided by dentists. Cochrane Database Syst Rev(8), CD010076. doi:10.1002/14651858.CD010076.pub2

Ebbert, J. O., Elrashidi, M. Y., & Stead, L. F. (2015). Interventions for smokeless tobacco use cessation. Cochrane Database Syst Rev(10), CD004306. doi:10.1002/14651858.CD004306.pub5

Ebersole, J. L., Graves, C. L., Gonzalez, O. A., Dawson, D., 3rd, Morford, L. A., Huja, P. E., . . . Wallet, S. M. (2016). Aging, inflammation, immunity and periodontal disease. Periodontol 2000, 72(1), 54-75. doi:10.1111/prd.12135

Eccles, S. A. (2010). Metastasis and the tumor microenvironment: A joint metastasis research society-AACR conference - Research on metastasis: Part 2. IDrugs, 13(11), 768-771.

Eda, S., Saito, T., Yamamura, T., Kawahara, H., & Takahashi, S. (1973). [Two cases of renal-cell carcinoma metastatic to the gingiva (author's transl)]. Shikwa Gakuho, 73(11), 1667-1674.

Editors, G. (2013). Proceedings of the 35th National Congress of the Italian Society of Histochemistry. European Journal of Histochemistry, 57(1s), 1-18. doi:10.4081/ejh.2013.s1

Efenberger, M., Agier, J., Pawlowska, E., & Brzezinska-Blaszczyk, E. (2015). Archaea prevalence in inflamed pulp tissues. Cent Eur J Immunol, 40(2), 194-200. doi:10.5114/ceji.2015.51358

Eivazi, Z. J., Fakhrgoo, A., & Estakhri, R. (2011). Gingival metastasis of renal cell Carcinoma. Iranian Journal of Cancer Prevention, 4(1), 44-47.

El-Rabbany, M., Duchnay, M., Raziee, H. R., Zych, M., Tenenbaum, H., Shah, P. S., & Azarpazhooh, A. (2019). Interventions for preventing osteoradionecrosis of the jaws in adults receiving head and neck radiotherapy. Cochrane Database Syst Rev, 2019(11). doi:10.1002/14651858.CD011559.pub2

Eldabagh, N., & Foley, J. (2016). Hierarchical nanoparticles in photodynamic therapy. Abstracts of Papers of the American Chemical Society, 252.

Eliyas, S., Al-Khayatt, A., Porter, R. W., & Briggs, P. (2013). Dental extractions prior to radiotherapy to the jaws for reducing post-radiotherapy dental complications. Cochrane Database Syst Rev(2), CD008857. doi:10.1002/14651858.CD008857.pub2

Elkhoury, J., Cacchillo, D. A., Tatakis, D. N., Kalmar, J. R., Allen, C. M., & Sedghizadeh, P. P. (2004). Undifferentiated malignant neoplasm involving the interdental gingiva: a case report. J Periodontol, 75(9), 1295-1299. doi:10.1902/jop.2004.75.9.1295

Elton-Marshall, T., Wijesingha, R., Kennedy, R. D., & Hammond, D. (2018). Disparities in knowledge about the health effects of smoking among adolescents following the release of new pictorial health warning labels. Prev Med, 111, 358-365. doi:10.1016/j.ypmed.2017.11.025

Emig, D., Ivliev, A., Pustovalova, O., Lancashire, L., Bureeva, S., Nikolsky, Y., & Bessarabova, M. (2013). Drug target prediction and repositioning using an integrated network-based approach. PLoS One, 8(4), e60618. doi:10.1371/journal.pone.0060618

Eng-Chong, T., Yean-Kee, L., Chin-Fei, C., Choon-Han, H., Sher-Ming, W., Li-Ping, C. T., . . . Yusof, R. (2012). Boesenbergia rotunda: From Ethnomedicine to Drug Discovery. Evid Based Complement Alternat Med, 2012, 473637. doi:10.1155/2012/473637

Epstein, J. B., Knowling, M. A., & Le Riche, J. C. (1987). Multiple gingival metastases from angiosarcoma of the breast. Oral Surg Oral Med Oral Pathol, 64(5), 554-557. doi:10.1016/0030-4220(87)90032-6

Esposito, M., Gisondi, P., Conti, A., Giunta, A., Del Giglio, M., Di Mercurio, M., . . . Girolomoni, G. (2017). Dose adjustment of biologic therapies for psoriasis in dermatological practice: a retrospective study. J Eur Acad Dermatol Venereol, 31(5), 863-869. doi:10.1111/jdv.14145

Esposito, M., Grusovin, M. G., Felice, P., Karatzopoulos, G., Worthington, H. V., & Coulthard, P. (2009). Interventions for replacing missing teeth: horizontal and vertical bone augmentation techniques for dental implant treatment. Cochrane Database Syst Rev(4), CD003607. doi:10.1002/14651858.CD003607.pub4

Esposito, M., & Worthington, H. V. (2013). Interventions for replacing missing teeth: dental implants in zygomatic bone for the rehabilitation of the severely deficient edentulous maxilla. Cochrane Database Syst Rev(9), CD004151. doi:10.1002/14651858.CD004151.pub3

Etokebe, G. E., Knezevic, J., Petricevic, B., Pavelic, J., Vrbanec, D., & Dembic, Z. (2009). Single-nucleotide polymorphisms in genes encoding toll-like receptor -2, -3, -4, and -9 in case-control study with breast cancer. Genet Test Mol Biomarkers, 13(6), 729-734. doi:10.1089/gtmb.2009.0045

European Congress of Epidemiology: "Healthy Living", Maastricht, Netherlands, June 25-27, 2015 Abstracts. (2015). European Journal of Epidemiology, 30(8), 721-1001.

Eyeson, J. D., Tenant-Flowers, M., Cooper, D. J., Johnson, N. W., & Warnakulasuriya, K. A. (2002). Oral manifestations of an HIV positive cohort in the era of highly active anti-retroviral therapy (HAART) in South London. J Oral Pathol Med, 31(3), 169-174. doi:10.1034/j.1600-0714.2002.310308.x

Fan, X., Alekseyenko, A. V., Wu, J., Peters, B. A., Jacobs, E. J., Gapstur, S. M., . . . Ahn, J. (2018). Human oral microbiome and prospective risk for pancreatic cancer: a population-based nested case-control study. Gut, 67(1), 120-127. doi:10.1136/gutjnl-2016-312580

Fang, C., Wu, L., Zhu, C., Xie, W. Z., Hu, H., & Zeng, X. T. (2020). A potential therapeutic strategy for prostatic disease by targeting the oral microbiome. Med Res Rev. doi:10.1002/med.21778

Fantasia, J. E., & Chen, L. (1979). A testicular tumor with gingival metastasis. Oral Surg Oral Med Oral Pathol, 48(1), 64-68. doi:10.1016/0030-4220(79)90238-x

Farrell, E. (2003). Medical choices available for management of menopause. Best Pract Res Clin Endocrinol Metab, 17(1), 1-16. doi:10.1016/s1521-690x(02)00087-8

Fedele, M., Gualillo, O., & Vecchione, A. (2012). Animal models of human pathology 2012. J Biomed Biotechnol, 2012, 404130. doi:10.1155/2012/404130

Fedele, S., Porter, S. R., D'Aiuto, F., Aljohani, S., Vescovi, P., Manfredi, M., . . . Yarom, N. (2010). Nonexposed variant of bisphosphonate-associated osteonecrosis of the jaw: a case series. Am J Med, 123(11), 1060-1064. doi:10.1016/j.amjmed.2010.04.033

Fee, P. A., Macey, R., Walsh, T., Clarkson, J. E., & Ricketts, D. (2020). Tests to detect and inform the diagnosis of root caries. Cochrane Database Syst Rev, 12(12), CD013806. doi:10.1002/14651858.CD013806

Fee, P. A., Riley, P., Worthington, H. V., Clarkson, J. E., Boyers, D., & Beirne, P. V. (2020). Recall intervals for oral health in primary care patients. Cochrane Database Syst Rev, 10(10), CD004346. doi:10.1002/14651858.CD004346.pub5

Fehm, T., Beck, V., Banys, M., Lipp, H. P., Hairass, M., Reinert, S., . . . Krimmel, M. (2009). Bisphosphonate-induced osteonecrosis of the jaw (ONJ): Incidence and risk factors in patients with breast cancer and gynecological malignancies. Gynecol Oncol, 112(3), 605-609. doi:10.1016/j.ygyno.2008.11.029

Fejzo, M. S., Burch, K., Mullin, P., MacGibbon, K. W., & Pasaniuc, B. (2020). Nausea and vomiting of pregnancy and hg risk genes associated with blood levels, sleep, stomach pain, and other traits. Obstetrics and Gynecology, 135, 151S-152S.

Fernandez-Barriales, M., Garcia-Montesinos, B., Garcia Reija, F., Mayorga Fernandez, M., & Saiz Bustillo, R. (2013). Metastatic leiomyosarcoma of the oral region from a uterine primary: a case report and review of the literature. J Oral Maxillofac Surg, 71(9), 1626-1633. doi:10.1016/j.joms.2013.03.003

Fernandez-Medarde, A., & Santos, E. (2011). Ras in cancer and developmental diseases. Genes Cancer, 2(3), 344-358. doi:10.1177/1947601911411084

Figuero, E., Roldan, S., Serrano, J., Escribano, M., Martin, C., & Preshaw, P. M. (2020). Efficacy of adjunctive therapies in patients with gingival inflammation: A systematic review and meta-analysis. J Clin Periodontol, 47 Suppl 22(S22), 125-143. doi:10.1111/jcpe.13244

Finch, C. E. (2010). Evolution in health and medicine Sackler colloquium: Evolution of the human lifespan and diseases of aging: roles of infection, inflammation, and nutrition. Proc Natl Acad Sci U S A, 107 Suppl 1(SUPPL. 1), 1718-1724. doi:10.1073/pnas.0909606106

Finer, N. (2015). Medical consequences of obesity. Medicine, 43(2), 88-93. doi:10.1016/j.mpmed.2014.11.003

Fisher, M. A., & Taylor, G. W. (2009). A prediction model for chronic kidney disease includes periodontal disease. J Periodontol, 80(1), 16-23. doi:10.1902/jop.2009.080226

Fitzgerald Jr, R. H., McInnes, B. K., & Manry, H. C. (1982). Renal cell carcinoma involving oral soft tissues. Journal of Oral and Maxillofacial Surgery, 40(9), 604-606.

Flores Cuéllar, Á., Sánchez Yáñez, E., & González Contreras, J. (2011). Bevacizumab in the treatment for platinum-resistant epithelial ovarian carcinoma. Atencion Farmaceutica, 13(1), 8-17.

Francini, F., Pascucci, A., Francini, E., Miano, S. T., Bargagli, G., Ruggiero, G., & Petrioli, R. (2011). Osteonecrosis of the jaw in patients with cancer who received zoledronic acid and bevacizumab. J Am Dent Assoc, 142(5), 506-513. doi:10.14219/jada.archive.2011.0220

Franks, P. W., & Atabaki-Pasdar, N. (2017). Causal inference in obesity research. J Intern Med, 281(3), 222-232. doi:10.1111/joim.12577

Friedlander, A. H. (2002). The physiology, medical management and oral implications of menopause. J Am Dent Assoc, 133(1), 73-81. doi:10.14219/jada.archive.2002.0025

Friedrich, R. E., & Blake, F. A. (2007). Avascular mandibular osteonecrosis in association with bisphosphonate therapy: a report on four patients. Anticancer Res, 27(4A), 1841-1845.

Frumovitz, M., & Sood, A. K. (2007). Vascular endothelial growth factor (VEGF) pathway as a therapeutic target in gynecologic malignancies. Gynecol Oncol, 104(3), 768-778. doi:10.1016/j.ygyno.2006.10.062

Frydman, A. S., & Fairley, J. A. (2011). New and innovative interventions in the management of pemphigus. G Ital Dermatol Venereol, 146(3), 211-224.

Frye, C. A. (2006). An overview of oral contraceptives: mechanism of action and clinical use. Neurology, 66(6 Suppl 3), S29-36. doi:10.1212/wnl.66.66_suppl_3.s29

Fu, E., Cheng, C. M., Chung, C. H., Lee, W. C., Chen, W. L., Sun, G. H., & Chien, W. C. (2021). Association of chronic periodontitis with prostatic hyperplasia and prostatitis: A population-based cohort study in Taiwan. J Periodontol, 92(1), 72-86. doi:10.1002/JPER.19-0706

Fujinaga, T., Kumamaru, W., Sugiura, T., Kobayashi, Y., Ohyama, Y., Ikari, T., . . . Mori, Y. (2014). Biological characterization and analysis of metastasis-related genes in cell lines derived from the primary lesion and lymph node metastasis of a squamous cell carcinoma arising in the mandibular gingiva. Int J Oncol, 44(5), 1614-1624. doi:10.3892/ijo.2014.2332

Fusco, V., Porta, C., Saia, G., Paglino, C., Bettini, G., Scoletta, M., . . . Bedogni, A. (2015). Osteonecrosis of the Jaw in Patients With Metastatic Renal Cell Cancer Treated With Bisphosphonates and Targeted Agents: Results of an Italian Multicenter Study and Review of the Literature. Clin Genitourin Cancer, 13(4), 287-294. doi:10.1016/j.clgc.2014.12.002

Fusco, V., Tommasi, L., Muni, A., Rouhanifar, H., Testori, O., Fasciolo, A., . . . Pertino, A. (2013). Role of bone scan in routine practice for early recognition of bisphosphonate-related osteonecrosis of the jaws (ONJ): Pro and con. Journal of Clinical Oncology, 31(15).

Gabaldon, V. H., & Haro-Gonzalez-Vico, V. (2019). Lack of an association between generalized granuloma annulare and malignancy: A case-control study. J Am Acad Dermatol, 80(6), 1799-1800. doi:10.1016/j.jaad.2019.02.056

Gaber, A. O., Kahan, B. D., Van Buren, C., Schulman, S. L., Scarola, J., Neylan, J. F., & Sirolimus High-Risk Study, G. (2008). Comparison of sirolimus plus tacrolimus versus sirolimus plus cyclosporine in high-risk renal allograft recipients: results from an open-label, randomized trial. Transplantation, 86(9), 1187-1195. doi:10.1097/TP.0b013e318187bab0

Gaekwad, S. S., & Gujjari, S. K. (2012). Cytomegalovirus occurrence in chronic periodontitis and in carcinoma of the cervix: an exploratory study. J Clin Diagn Res, 6(8), 1442-1447. doi:10.7860/JCDR/2012/4389.2383

Gargano, L. M., & Hughes, J. M. (2014). Microbial Origins of Chronic Diseases. In Annual Review of Public Health, Vol 35 (Vol. 35, pp. 65-82).

Garrido-Mesa, N., Zarzuelo, A., & Galvez, J. (2013). Minocycline: far beyond an antibiotic. Br J Pharmacol, 169(2), 337-352. doi:10.1111/bph.12139

Gavalda, C., & Bagan, J. V. (2016). Concept, diagnosis and classification of bisphosphonate-associated osteonecrosis of the jaws. A review of the literature. Med Oral Patol Oral Cir Bucal, 21(3), e260-270. doi:10.4317/medoral.21001

Gayathri, G., Muthukumar, S., Joseph, L. D., & Suresh, R. (2014). Immunolocalization of heme oxygenase-1 in periodontal diseases. Indian J Dent Res, 25(5), 567-571. doi:10.4103/0970-9290.147080

Gazolla, C. M., Ribeiro, A., Moyses, M. R., Oliveira, L. A., Pereira, L. J., & Sallum, A. W. (2007). Evaluation of the incidence of preterm low birth weight in patients undergoing periodontal therapy. J Periodontol, 78(5), 842-848. doi:10.1902/jop.2007.060295

Gelosa, P., Castiglioni, L., Camera, M., & Sironi, L. (2020). Repurposing of drugs approved for cardiovascular diseases: Opportunity or mirage? Biochem Pharmacol, 177, 113895. doi:10.1016/j.bcp.2020.113895

Genin, O., Rechavi, G., Nagler, A., Ben-Itzhak, O., Nazemi, K. J., & Pines, M. (2008). Myofibroblasts in pulmonary and brain metastases of alveolar soft-part sarcoma: a novel target for treatment? Neoplasia, 10(9), 940-948. doi:10.1593/neo.08456

Ghaninejad, H., Ehsani, A. H., Ghiasi, M., Noormohammadpour, P., Najafi, E., Naderi, G., . . . Kiani, P. (2009). Benign and malignant skin lesions in renal transplant recipients. Indian J Dermatol, 54(3), 247-250. doi:10.4103/0019-5154.55634

Ghidini, G., Manfredi, M., Giovannacci, I., Mergoni, G., Sarraj, A., Mureddu, M., . . . Vescovi, P. (2017). Medication-related osteonecrosis of the jaw: risk factors in patients under biphosphonate versus patients under antiresorptive-antiangiogenic drugs. Minerva Stomatol, 66(4), 135-140. doi:10.23736/S0026-4970.17.04056-0

Gilabert, M., Provansal, M., Cappiello, M., Walz, J., Brunelle, S., Salem, N., & Gravis, G. (2011a). 7145 POSTER Self Assessment of Buccodental Toxicity – Comparison of Patients With Metastatic Renal-cell Carcinoma (RCC) Treated With Sunitinib to Patients Treated With Chemotherapy. European Journal of Cancer, 47, S518. doi:10.1016/s0959-8049(11)72060-3

Gilabert, M., Provansal, M., Cappiello, M., Walz, J., Brunelle, S., Salem, N., & Gravis, G. (2011b). Self assessment of buccodental toxicity: Comparison of patients with metastatic renal cell carcinoma (RCC) treated with sunitinib with patients treated with chemotherapy. Journal of Clinical Oncology, 29(15).

Gilabert, M., Provansal, M., Cappiello, M., Walz, Y., Salem, N., Tarpin, C., . . . Gravis, G. (2013). Buccodental side effects of sunitinib in patients with metastatic renal cell carcinoma. Br J Cancer, 109(7), 1750-1754. doi:10.1038/bjc.2013.516

Gillison, M. L., Castellsague, X., Chaturvedi, A., Goodman, M. T., Snijders, P., Tommasino, M., . . . Franceschi, S. (2014). Eurogin Roadmap: comparative epidemiology of HPV infection and associated cancers of the head and neck and cervix. Int J Cancer, 134(3), 497-507. doi:10.1002/ijc.28201

[Gingival metastasis, mediastinal syndrome and acute abdomen]. (1982). Medicina (B Aires), 42(1), 79-89.

Gion, M., & Fabricio, A. S. (2013). Inflammation markers: new actors in the cancer biomarker tale. Int J Biol Markers, 28(1), 1-2. doi:10.5301/JBM.2013.11045

Giuliani, M., Lajolo, C., Lucchese, A., Marino, M. C., Ricci, R., & Favia, G. (2010). Short Submitted Peer Reviewed Abstracts. Oral Diseases, 16(6), 516-572. doi:10.1111/j.1601-0825.2010.01743.x

Giuliani, M., Lajolo, C., Lucchese, A., Ricci, R., & Favia, G. (2010). Oral metastases as first manifestation of renal cell carcinoma: Presentation of clinical cases. Anticancer Research, 30(4), 1527.

Giurdanella, F., Nijenhuis, A. M., Diercks, G. F. H., Jonkman, M. F., & Pas, H. H. (2018). Keratinocyte Binding Assay Identifies Anti-Desmosomal Pemphigus Antibodies Where Other Tests Are Negative. Front Immunol, 9(APR), 839. doi:10.3389/fimmu.2018.00839

Glade, M. J. (2013). Vitamin D: health panacea or false prophet? Nutrition, 29(1), 37-41. doi:10.1016/j.nut.2012.05.010

Gnant, M., Mlineritsch, B., Schippinger, W., Luschin-Ebengreuth, G., Postlberger, S., Menzel, C., . . . Marth, C. (2009). Endocrine therapy plus zoledronic acid in premenopausal breast cancer. N Engl J Med, 360(7), 679-691. doi:10.1056/NEJMoa0806285

Godby, A. F., Sonntag, R. W., & Cosentino, B. J. (1967). Hypernephroma with metastasis to the mandibular gingiva. Report of a case. Oral Surg Oral Med Oral Pathol, 23(5), 696-700. doi:10.1016/0030-4220(67)90354-4

Goel, S., Chu, M., Sedlis, E., Friedman, K., & Blum, M. (2009). Abstracts of the 80th Annual Meeting of the American Thyroid Association. Palm Beach, Florida, USA. September 23-27, 2009. Thyroid, 19 Suppl 1, S21-93, S100-116. doi:10.1089/thy.2009.1589

Goepfert, A. R., Jeffcoat, M. K., Andrews, W. W., Faye-Petersen, O., Cliver, S. P., Goldenberg, R. L., & Hauth, J. C. (2004). Periodontal disease and upper genital tract inflammation in early spontaneous preterm birth. Obstet Gynecol, 104(4), 777-783. doi:10.1097/01.AOG.0000139836.47777.6d

Goldenberg, R. L., Culhane, J. F., Iams, J. D., & Romero, R. (2008). Epidemiology and causes of preterm birth. Lancet, 371(9606), 75-84. doi:10.1016/S0140-6736(08)60074-4

Goldsby, R. E., Taggart, D. R., & Ablin, A. R. (2006). Surviving childhood cancer: the impact on life. Paediatr Drugs, 8(2), 71-84. doi:10.2165/00148581-200608020-00001

Golenbiewski, J., Eudy, A., Clowse, M., & Allen, N. (2019). Hospital Admissions and Mortality in Patients with ANCA-associated Vasculitis. Arthritis & Rheumatology, 71.

Gomes-Filho, I. S., Batista, J. E. T., Trindade, S. C., Passos-Soares, J. S., Cerqueira, E. M. M., Costa, T. S. D., . . . Cruz, S. S. D. (2020). Obesity and periodontitis are not associated in pregnant women. J Periodontal Res, 55(1), 77-84. doi:10.1111/jre.12690

Gondivkar, S. M., Gondivkar, R. S., Gadbail, A. R., Chole, R., Mankar, M., & Yuwanati, M. (2013). Chronic periodontitis and the risk of head and neck squamous cell carcinoma: facts and figures. Exp Oncol, 35(3), 163-167.

Gordon, A. J., Conley, J. W., & Gordon, J. M. (2013). Medical consequences of marijuana use: a review of current literature. Curr Psychiatry Rep, 15(12), 419. doi:10.1007/s11920-013-0419-7

Gordon, M. S., Robert, F., Matei, D., Mendelson, D. S., Goldman, J. W., Chiorean, E. G., . . . Rosen, L. S. (2014). An open-label phase Ib dose-escalation study of TRC105 (anti-endoglin antibody) with bevacizumab in patients with advanced cancer. Clin Cancer Res, 20(23), 5918-5926. doi:10.1158/1078-0432.CCR-14-1143

Gore, J. L., Swerdloff, R. S., & Rajfer, J. (2005). Androgen deficiency in the etiology and treatment of erectile dysfunction. Urol Clin North Am, 32(4), 457-468, vi-vii. doi:10.1016/j.ucl.2005.08.002

Górski, B., & Bryłka, M. (2012). Bisphosphonates - Risk assessment of osteonecrosis of the jaw and potential benefits for periodontal therapy. Dental and Medical Problems, 49(4), 576-582.

Gould, E., Klos, J., Price, J., Harris, T., Vaden, S., & Tolbert, M. K. (2018). Retrospective analysis of the effect of acid-suppressant therapy on clinicopathologic parameters of cats with chronic kidney disease. J Feline Med Surg, 20(6), 520-527. doi:10.1177/1098612X17718132

Graphical abstract TOC. (2020). Tetrahedron Lett, 61(40), 152456. doi:10.1016/S0040-4039(20)30936-9

Greco, A. J., Baluarte, J. H., Meyers, K. E., Sellers, M. T., Suchi, M., Biegel, J. A., & Kaplan, B. S. (2005). Chromophobe renal cell carcinoma in a pediatric living-related kidney transplant recipient. Am J Kidney Dis, 45(6), e105-108. doi:10.1053/j.ajkd.2005.03.012

Grewal, V. S., & Fayans, E. P. (2008). Bisphosphonate-associated osteonecrosis: a clinician's reference to patient management. Todays FDA, 20(8), 38-41, 43-36.

Grimm, M., Henopp, T., Hoefert, S., Schaefer, F., Kluba, S., Krimmel, M., & Reinert, S. (2012). Multiple osteolytic lesions of intraosseous adenoid cystic carcinoma in the mandible mimicking apical periodontitis. Int Endod J, 45(12), 1156-1164. doi:10.1111/j.1365-2591.2012.02079.x

Grimm, M., Rinaldi, M., Yonan, N. A., Arpesella, G., Arizon Del Prado, J. M., Pulpon, L. A., . . . Laufer, G. (2006). Superior prevention of acute rejection by tacrolimus vs. cyclosporine in heart transplant recipients--a large European trial. Am J Transplant, 6(6), 1387-1397. doi:10.1111/j.1600-6143.2006.01300.x

Gross, A., Kay, T. M., Paquin, J. P., Blanchette, S., Lalonde, P., Christie, T., . . . Cervical Overview, G. (2015). Exercises for mechanical neck disorders. Cochrane Database Syst Rev, 1(1), CD004250. doi:10.1002/14651858.CD004250.pub5

Grusovin, M. G., Coulthard, P., Worthington, H. V., George, P., & Esposito, M. (2010). Interventions for replacing missing teeth: maintaining and recovering soft tissue health around dental implants. Cochrane Database Syst Rev(8), CD003069. doi:10.1002/14651858.CD003069.pub4

Gubbi, A., Kendrick, J. E., & Finkler, N. J. (2014). The role of bevacizumab in recurrent, platinum-sensitive ovarian cancer. Expert Rev Anticancer Ther, 14(10), 1105-1113. doi:10.1586/14737140.2014.956095

Guerrero-Preston, R., White, J. R., Godoy-Vitorino, F., Rodriguez-Hilario, A., Navarro, K., Gonzalez, H., . . . Sidransky, D. (2017). High-resolution microbiome profiling uncovers Fusobacterium nucleatum, Lactobacillus gasseri/johnsonii, and Lactobacillus vaginalis associated to oral and oropharyngeal cancer in saliva from HPV positive and HPV negative patients treated with surgery and chemo-radiation. Oncotarget, 8(67), 110931-110948. doi:10.18632/oncotarget.20677

Habib, S. L., Al-Obaidi, N. Y., Nowacki, M., Pietkun, K., Zegarska, B., Kloskowski, T., . . . Liang, S. (2016). Is mTOR Inhibitor Good Enough for Treatment All Tumors in TSC Patients? J Cancer, 7(12), 1621-1631. doi:10.7150/jca.14747

Haidrus, R., Nayak, S. U., & Kamath, D. G. (2020). Impact of Nutrient-Gene-Interaction on Periodontal DiseasesAn Over View. Indian Journal of Forensic Medicine & Toxicology, 14(4), 457-463. doi:10.37506/ijfmt.v14i4.11520

Hakeem, A., Fitzpatrick, S. G., Gonsalves, C. A., Isom, J., Islam, M. N., Bhattacharyya, I., . . . Drew, P. A. (2020). p16INK4a as a proliferation marker unrelated to HPV expression in odontogenic cysts and tumors. J Oral Pathol Med, 49(1), 72-81. doi:10.1111/jop.12972

Hammami, S., Krichen Makni, S., Ellouze, S., Mnif, H., Fakhfakh, I., Gouiaa, N., . . . Sellami-Boudawara, T. (2007). [A gingival swelling]. Rev Stomatol Chir Maxillofac, 108(5), 471-472. doi:10.1016/j.stomax.2007.01.009

Han, J. (2013). Review of Major Directions in Non-Equilibrium Atmospheric Plasma Treatments in Medical, Biological, and Bioengineering Applications. Plasma Medicine, 3(3), 175-243. doi:10.1615/PlasmaMed.2014012248

Hanna, W. M., & Cruickshank, B. (1976). Starch granulomas after transurethral resection of bladder tumors. Urology, 8(5), 507-510. doi:10.1016/0090-4295(76)90291-0

Hansen, C. B., & Dahle, K. W. (2012). Cutaneous lupus erythematosus. Dermatol Ther, 25(2), 99-111. doi:10.1111/j.1529-8019.2012.01508.x

Haque, A. M., Kumar, J., & Beegle, S. H. (2009). Lung abscess caused by Solobacterium moorei in a patient with severe COPD. Chest, 136(4).

Hardy, R., Shepherd, C. W., Donnelly, D. E., McKee, S. A., & Morrison, P. J. (2012). Constellation of five facial features of tuberous sclerosis in a child with a TSC2 1808A>G mutation. Oncologist, 17(7), 925-926. doi:10.1634/theoncologist.2011-0407

Hare, N. C., Arnott, I. D., & Satsangi, J. (2008). Therapeutic options in acute severe ulcerative colitis. Expert Rev Gastroenterol Hepatol, 2(3), 357-370. doi:10.1586/17474124.2.3.357

Harris, R., Gamboa, A., Dailey, Y., & Ashcroft, A. (2012). One-to-one dietary interventions undertaken in a dental setting to change dietary behaviour. Cochrane Database Syst Rev(3), CD006540. doi:10.1002/14651858.CD006540.pub2

Harris, S. G., Padilla, J., Koumas, L., Ray, D., & Phipps, R. P. (2002). Prostaglandins as modulators of immunity. Trends in Immunology, 23(3), 144-150. doi:10.1016/s1471-4906(01)02154-8

Hart, R. J. (2016). Physiological Aspects of Female Fertility: Role of the Environment, Modern Lifestyle, and Genetics. Physiol Rev, 96(3), 873-909. doi:10.1152/physrev.00023.2015

Hasegawa-Nakamura, K., Tateishi, F., Nakamura, T., Nakajima, Y., Kawamata, K., Douchi, T., . . . Noguchi, K. (2011). The possible mechanism of preterm birth associated with periodontopathic Porphyromonas gingivalis. J Periodontal Res, 46(4), 497-504. doi:10.1111/j.1600-0765.2011.01366.x

Hashiguchi, Y., Fukuda, T., Ichimura, T., Matsumoto, Y., Yasui, T., Sumi, T., & Ishiko, O. (2015). Chemotherapy-induced thrombocytopenia and clinical bleeding in patients with gynecologic malignancy. Eur J Gynaecol Oncol, 36(2), 168-173. doi:10.12892/cjgo2595.2015

Hassona, Y., Almuhaisen, G., Almansour, A., & Scully, C. (2017). Lymphoma presenting as a toothache: a wolf in sheep's clothing. BMJ Case Rep, 2017. doi:10.1136/bcr-2016-218686

Hatz, M. H., Schremser, K., & Rogowski, W. H. (2014). Is individualized medicine more cost-effective? A systematic review. PharmacoEconomics, 32(5), 443-455. doi:10.1007/s40273-014-0143-0

Hatziotis, J. C., Constantinidou, H., & Papanayotou, P. H. (1973). Metastatic tumors of the oral soft tissues. Review of the literature and report of a case. Oral Surg Oral Med Oral Pathol, 36(4), 544-550. doi:10.1016/0030-4220(73)90312-5

Havas, D. (2016). Immune biomarkers of medication (bisphosphonates) related osteonecrosis of the jaws (MRONJ). Journal of Oral and Maxillofacial Surgery, 74(9), e69-e70.

Hay, S. I., Abajobir, A. A., Abate, K. H., Abbafati, C., Abbas, K. M., Abd-Allah, F., . . . Murray, C. J. L. (2017). Global, regional, and national disability-adjusted life-years (DALYs) for 333 diseases and injuries and healthy life expectancy (HALE) for 195 countries and territories, 1990–2016: a systematic analysis for the Global Burden of Disease Study 2016. The Lancet, 390(10100), 1260-1344. doi:10.1016/s0140-6736(17)32130-x

He, J. H., & Li, Y. G. (2013). Characteristics of antisense non-coding RNA in the INK4 locus and its roles in disease. Chin Med Sci J, 28(2), 122-123. doi:10.1016/s1001-9294(13)60034-6

Healy, C. M., Tobin, A. M., Kirby, B., & Flint, S. R. (2006). Oral lesions as an initial manifestation of dermatomyositis with occult malignancy. Oral Surg Oral Med Oral Pathol Oral Radiol Endod, 101(2), 184-187. doi:10.1016/j.tripleo.2005.08.030

Heinrich, N. A., McKeever, P. J., & Eisenschenk, M. C. (2011). Adverse events in 50 cats with allergic dermatitis receiving ciclosporin. Vet Dermatol, 22(6), 511-520. doi:10.1111/j.1365-3164.2011.00983.x

Hellwege, J. N., Torstenson, E. S., Edwards, T. L., & Velez Edwards, D. R. (2019). 2019 | Scientific Abstracts. Reproductive Sciences, 26(1_suppl), 62A-390A. doi:10.1177/1933719119834079

Hemady, R. K., Chan, A. S., & Nguyen, A. T. (2005). Immunosuppressive agents and nonsteroidal anti-inflammatory drugs for ocular immune and inflammatory disorders. Ophthalmol Clin North Am, 18(4), 511-528, vi. doi:10.1016/j.ohc.2005.07.002

Heng, C. K., & Heng, J. (1995). Implications of malignant lymphoma on a periapical mandibular lesion. Gen Dent, 43(5), 454-458.

Heo, J. S., Ahn, K. H., & Park, J. S. (2020). Radiological screening of maternal periodontitis for predicting adverse pregnancy and neonatal outcomes. Sci Rep, 10(1), 21266. doi:10.1038/s41598-020-78385-0

Hider, P., Wilson, L., Rose, J., Weiser, T. G., Gruen, R., & Bickler, S. W. (2015). The role of facility-based surgical services in addressing the national burden of disease in New Zealand: An index of surgical incidence based on country-specific disease prevalence. Surgery, 158(1), 44-54. doi:10.1016/j.surg.2015.04.005

Hinson, A. M., Siegel, E. R., & Stack, B. C., Jr. (2015). Temporal correlation between bisphosphonate termination and symptom resolution in osteonecrosis of the jaw: a pooled case report analysis. J Oral Maxillofac Surg, 73(1), 53-62. doi:10.1016/j.joms.2014.07.012

Hiraiwa, T., & Izumi, M. (2013). Successful management of Rothia aeria endocarditis with renal transplantation patient: A case report. European Journal of Internal Medicine, 24, e204. doi:10.1016/j.ejim.2013.08.521

Hirshberg, A., Shnaiderman-Shapiro, A., Kaplan, I., & Berger, R. (2008). Metastatic tumours to the oral cavity - pathogenesis and analysis of 673 cases. Oral Oncol, 44(8), 743-752. doi:10.1016/j.oraloncology.2007.09.012

Ho, C. W., Lin, C. Y., Liaw, Y. W., Chiang, H. L., Chin, Y. T., Huang, R. L., . . . Hwang, J. (2016). The cytokine-cosmc signaling axis upregulates the tumor-associated carbohydrate antigen Tn. Oncotarget, 7(38), 61930-61944. doi:10.18632/oncotarget.11324

Hodel, C., & Meier-Ruge, W. (1966). Enzyme histochemical investigations on giant cells of specific and nonspecific granulation tissue, and of malignant tumours. Pathol Eur, 1(1), 425-432.

Hodge, J. C., Pearce, K. E., Wang, X., Wiktor, A. E., Oliveira, A. M., & Greipp, P. T. (2014). Molecular cytogenetic analysis for TFE3 rearrangement in Xp11.2 renal cell carcinoma and alveolar soft part sarcoma: validation and clinical experience with 75 cases. Mod Pathol, 27(1), 113-127. doi:10.1038/modpathol.2013.83

Hoefert, S., Yuan, A., Munz, A., Grimm, M., Elayouti, A., & Reinert, S. (2017). Clinical course and therapeutic outcomes of operatively and non-operatively managed patients with denosumab-related osteonecrosis of the jaw (DRONJ). J Craniomaxillofac Surg, 45(4), 570-578. doi:10.1016/j.jcms.2017.01.013

Holgado, M. A., Alvarez-Fuentes, J., Fernandez-Arevalo, M., & Arias, J. L. (2011). Possibilities of poly(D,L-lactide-co-glycolide) in the formulation of nanomedicines against cancer. Curr Drug Targets, 12(8), 1096-1111. doi:10.2174/138945011795906606

Holley, J. L. (2013). General medical care of the dialysis patient: Core Curriculum 2013. Am J Kidney Dis, 61(1), 171-183. doi:10.1053/j.ajkd.2012.07.023

Hollis, B. W., & Wagner, C. L. (2013). Clinical review: The role of the parent compound vitamin D with respect to metabolism and function: Why clinical dose intervals can affect clinical outcomes. J Clin Endocrinol Metab, 98(12), 4619-4628. doi:10.1210/jc.2013-2653

Hong, Y. H., Han, S. J., Lee, D., Kim, S. K., & Jee, B. C. (2019). Adverse symptoms during short-term use of ulipristal acetate in women with uterine myomas and/or adenomyosis. J Obstet Gynaecol Res, 45(4), 865-870. doi:10.1111/jog.13917

Hope, I., Morton, K., Newlands, C., Butler-Manuel, S., & Madhuri, T. K. (2017). Lockjaw from a metastatic uterine leiomyosarcoma- case report and review of the literature. BMC Womens Health, 17(1), 119. doi:10.1186/s12905-017-0472-1

Horstman, H., Gruhl, J., Smith, L., Ganti, A. K., & Shonka, N. A. (2018). Safety of long-term anticoagulation in patients with brain metastases. Med Oncol, 35(4), 43. doi:10.1007/s12032-018-1101-z

Hoversten, K., Vallapureddy, R., Lasho, T., Finke, C., Ketterling, R., Hanson, C., . . . Patnaik, M. M. (2018). Nonhepatosplenic extramedullary manifestations of chronic myelomonocytic leukemia: clinical, molecular and prognostic correlates. Leuk Lymphoma, 59(12), 2998-3001. doi:10.1080/10428194.2018.1452212

Howard, D. R., Munir, T., McParland, L., Rawstron, A. C., Chalmers, A., Gregory, W. M., . . . Hillmen, P. (2017). Clinical effectiveness and cost-effectiveness results from the randomised, Phase IIB trial in previously untreated patients with chronic lymphocytic leukaemia to compare fludarabine, cyclophosphamide and rituximab with fludarabine, cyclophosphamide, mitoxantrone and low-dose rituximab: the Attenuated dose Rituximab with ChemoTherapy In Chronic lymphocytic leukaemia (ARCTIC) trial. Health Technol Assess, 21(28), 1-374. doi:10.3310/hta21280

Hsu, J. C., Lin, P. C., & Chu, H. J. (2017). Abstract. Pharmacoepidemiology and Drug Safety, 26, 3-636. doi:10.1002/pds.4275

Hu, J., Van den Steen, P. E., Sang, Q. X., & Opdenakker, G. (2007). Matrix metalloproteinase inhibitors as therapy for inflammatory and vascular diseases. Nat Rev Drug Discov, 6(6), 480-498. doi:10.1038/nrd2308

Hu, Y., Wang, J., Tao, H., Wu, B., Sun, J., Cheng, Y., . . . Li, R. (2013). Increased risk of high-grade hemorrhage in cancer patients treated with gemcitabine: a meta-analysis of 20 randomized controlled trials. PLoS One, 8(9), e74872. doi:10.1371/journal.pone.0074872

Huang, I. S., Huang, S. E., Kao, W. T., Chiang, C. Y., Chang, T., Lin, C. I., . . . Chung, H. J. (2018). Patients with chronic periodontitis are more likely to develop upper urinary tract stone: a nation-wide population-based eight-year follow up study. Peerj, 6, e5287. doi:10.7717/peerj.5287

Huang, Y., Michaud, D. S., Lu, J., Carter, H. B., & Platz, E. A. (2019). The association between clinically determined periodontal disease and prostate-specific antigen concentration in men without prostate cancer: the 2009-2010 National Health and Nutrition Examination Survey. Cancer Causes Control, 30(12), 1293-1300. doi:10.1007/s10552-019-01238-3

Hummel, M., & Hetzer, R. (2004). Impact of cyclosporine on cardiac transplantation in Berlin. Transplant Proc, 36(2 Suppl), 337S-345S. doi:10.1016/j.transproceed.2004.01.029

Humphreys, K., Blodgett, J. C., & Roberts, L. W. (2015). The exclusion of people with psychiatric disorders from medical research. J Psychiatr Res, 70, 28-32. doi:10.1016/j.jpsychires.2015.08.005

Hung, K., Montalvao, C., Tanaka, R., Kawai, T., & Bornstein, M. M. (2020). The use and performance of artificial intelligence applications in dental and maxillofacial radiology: A systematic review. Dentomaxillofac Radiol, 49(1), 20190107. doi:10.1259/dmfr.20190107

Hunyady, B., Abonyi, M., Csefko, K., Gervain, J., Haragh, A., Horvath, G., . . . Makara, M. (2016). [Efficacy and safety of boceprevir based triple therapy in Hungarian patients with hepatitis C genotype 1 infection, advanced stage fibrosis and prior treatment failure]. Orv Hetil, 157(34), 1366-1374. doi:10.1556/650.2016.30538

Huston, W. M., & Tachedjian, G. (2020). Editorial: Interplay of Infection and Microbiome. Front Cell Infect Microbiol, 10, 304. doi:10.3389/fcimb.2020.00304

Iavazzo, C., Papakiritsis, M., & Gkegkes, I. D. (2016). Hypodontia and ovarian cancer: A systematic review. J Turk Ger Gynecol Assoc, 17(1), 41-44. doi:10.5152/jtgga.2015.15174

Ichimaru, R., Tominari, T., Yoshinouchi, S., Matsumoto, C., Watanabe, K., Hirata, M., . . . Inada, M. (2018). Raloxifene reduces the risk of local alveolar bone destruction in a mouse model of periodontitis combined with systemic postmenopausal osteoporosis. Arch Oral Biol, 85, 98-103. doi:10.1016/j.archoralbio.2017.09.017

Inaba, H., Tagashira, M., Kanda, T., Murakami, Y., Amano, A., & Matsumoto-Nakano, M. (2016). Apple- and Hop-Polyphenols Inhibit Porphyromonas gingivalis-Mediated Precursor of Matrix Metalloproteinase-9 Activation and Invasion of Oral Squamous Cell Carcinoma Cells. J Periodontol, 87(9), 1103-1111. doi:10.1902/jop.2016.160047

Iram, F., Khan, S. A., & Husain, A. (2017). Phytochemistry and potential therapeutic actions of Boswellic acids: A mini-review. Asian Pacific Journal of Tropical Biomedicine, 7(6), 513-523. doi:10.1016/j.apjtb.2017.05.001

Irct20151013024509N. (2018). The effect of periodontal treatment on blood parameters. http://www.who.int/trialsearch/Trial2.aspx?TrialID=IRCT20151013024509N3.

Ishak, R. S., Aad, S. A., Kyei, A., & Farhat, F. S. (2014). Cutaneous manifestations of anti-angiogenic therapy in oncology: Review with focus on VEGF inhibitors. Crit Rev Oncol Hematol, 90(2), 152-164. doi:10.1016/j.critrevonc.2013.11.007

Ishigaki, K., Akiyama, M., Kanai, M., Takahashi, A., Kawakami, E., Sugishita, H., . . . Kamatani, Y. (2020). Large-scale genome-wide association study in a Japanese population identifies novel susceptibility loci across different diseases. Nat Genet, 52(7), 669-679. doi:10.1038/s41588-020-0640-3

Ishizawa, K., Usuki, K., Ando, K., Ueda, Y., Kiguchi, T., Uike, N., . . . Iida, H. (2016). Multicenter, Open-Label Phase I Clinical Study of Oral Rigosertib in Japanese Patients with Recurrent/Relapsed or Refractory Myelodysplastic Syndromes: Tolerability, Efficacy, and Pharmacokinetic Profiles. Blood, 128(22), 5538-5538. doi:10.1182/blood.V128.22.5538.5538

Isono, T., Kim, C. J., Ando, Y., Sakurai, H., Okada, Y., & Inoue, H. (2009). Suppression of cell invasiveness by periostin via TAB1/TAK1. Int J Oncol, 35(2), 425-432. doi:10.3892/ijo_00000355

Itabashi, A., Yoh, K., Chines, A. A., Miki, T., Takada, M., Sato, H., . . . Ohta, H. (2011). Effects of bazedoxifene on bone mineral density, bone turnover, and safety in postmenopausal Japanese women with osteoporosis. J Bone Miner Res, 26(3), 519-529. doi:10.1002/jbmr.252

Ivy, S. P., Liu, J. F., Lee, J. M., Matulonis, U. A., & Kohn, E. C. (2016). Cediranib, a pan-VEGFR inhibitor, and olaparib, a PARP inhibitor, in combination therapy for high grade serous ovarian cancer. Expert Opin Investig Drugs, 25(5), 597-611. doi:10.1517/13543784.2016.1156857

Jacobs, H., Ruben, M. P., & Lyon, J. (1966). Renal-cell carcinoma metastatic to the mandible and gingiva. Oral Surg Oral Med Oral Pathol, 22(5), 649-653. doi:10.1016/0030-4220(66)90170-8

Jain, A., Saini, V., & Kohli, D. V. (2013). Edible transgenic plant vaccines for different diseases. Curr Pharm Biotechnol, 14(6), 594-614. doi:10.2174/138920101131400225

Jain, A., Shetty, D. C., Juneja, S., & Narwal, N. (2016). Molecular Characterization of Clear Cell Lesions of Head and Neck. J Clin Diagn Res, 10(5), ZE18-23. doi:10.7860/JCDR/2016/14394.7867

Jakobsen, J. C., Nielsen, E. E., Feinberg, J., Katakam, K. K., Fobian, K., Hauser, G., . . . Gluud, C. (2017). Direct-acting antivirals for chronic hepatitis C. Cochrane Database Syst Rev, 9(9), CD012143. doi:10.1002/14651858.CD012143.pub3

Jaladat, A. M., Atarzadeh, F., Rezaeizadeh, H., Mofid, B., Mosalaie, A., Farhan, F., & Amin, G. (2015). Botanicals: an alternative remedy to radiotherapy-induced dysuria. Complement Ther Med, 23(1), 90-99. doi:10.1016/j.ctim.2014.11.004

James, W. D., & Lupton, G. P. (1988). Acquired dyskeratotic leukoplakia. Arch Dermatol, 124(1), 117-120.

Jankowska, M. (2011). Sexual functioning of testicular cancer survivors and their partners - A review of literature. Rep Pract Oncol Radiother, 17(1), 54-62. doi:10.1016/j.rpor.2011.11.001

Javed, F., Bello Correra, F. O., Chotai, M., Tappuni, A. R., & Almas, K. (2010). Systemic conditions associated with areca nut usage: a literature review. Scand J Public Health, 38(8), 838-844. doi:10.1177/1403494810379291

Je, Y., Schutz, F. A., & Choueiri, T. K. (2009). Risk of bleeding with vascular endothelial growth factor receptor tyrosine-kinase inhibitors sunitinib and sorafenib: a systematic review and meta-analysis of clinical trials. Lancet Oncol, 10(10), 967-974. doi:10.1016/S1470-2045(09)70222-0

Jewett, A., Man, Y. G., & Tseng, H. C. (2013). Dual functions of natural killer cells in selection and differentiation of stem cells; role in regulation of inflammation and regeneration of tissues. J Cancer, 4(1), 12-24. doi:10.7150/jca.5519

Ji, H. L., Zhao, R. Z., Chen, Z. X., Shetty, S., Idell, S., & Matalon, S. (2012). delta ENaC: a novel divergent amiloride-inhibitable sodium channel. Am J Physiol Lung Cell Mol Physiol, 303(12), L1013-1026. doi:10.1152/ajplung.00206.2012

Jia, B., Qiu, X., Chen, J., Sun, X., Zheng, X., Zhao, J., . . . Wang, Z. (2019). A feed-forward regulatory network lncPCAT1/miR-106a-5p/E2F5 regulates the osteogenic differentiation of periodontal ligament stem cells. J Cell Physiol, 234(11), 19523-19538. doi:10.1002/jcp.28550

Jiang, A., Wang, J., Joshi, M., & Christoforidis, J. B. (2013). Systemic treatments for noninfectious vitreous inflammation. Mediators Inflamm, 2013, 515312. doi:10.1155/2013/515312

Jinga, V., Braticevici, B., Moisanu, D., Tanase, F., Gabara, A., & Radavoi, D. (2012). Zoledronic acid therapy and osteonecrosis of the jaw in prostate cancer treatment. European Urology, Supplements, 11(4), 124.

Jobanputra, P., Maggs, F., Homer, D., & Bevan, J. (2002). Monitoring and assessing the safety of disease-modifying antirheumatic drugs: a West Midlands experience. Drug Saf, 25(15), 1099-1105. doi:10.2165/00002018-200225150-00004

Johansson, C. (2016). The 2015 Pregnancy Summit, London, UK. Womens Health (Lond), 12(2), 167-170. doi:10.2217/whe.15.107

Joharatnam-Hogan, N., Cafferty, F., Hubner, R., Swinson, D., Sothi, S., Gupta, K., . . . Steele, R. J. C. (2019). Aspirin as an adjuvant treatment for cancer: feasibility results from the Add-Aspirin randomised trial. The Lancet Gastroenterology & Hepatology, 4(11), 854-862. doi:10.1016/s2468-1253(19)30289-4

Johnson, D. H. (2020). Geriatric Hedgehogs. Vet Clin North Am Exot Anim Pract, 23(3), 615-637. doi:10.1016/j.cvex.2020.05.005

Jorgensen, K. K., Olsen, I. C., Goll, G. L., Lorentzen, M., Bolstad, N., Haavardsholm, E. A., . . . group, N.-S. s. (2017). Switching from originator infliximab to biosimilar CT-P13 compared with maintained treatment with originator infliximab (NOR-SWITCH): a 52-week, randomised, double-blind, non-inferiority trial. Lancet, 389(10086), 2304-2316. doi:10.1016/S0140-6736(17)30068-5

Jouyban, A., Fakhree, M. A., & Shayanfar, A. (2010). Review of pharmaceutical applications of N-methyl-2-pyrrolidone. J Pharm Pharm Sci, 13(4), 524-535. doi:10.18433/j3p306

Jung, S. Y., Suh, H. S., Park, J. W., & Kwon, J. W. (2019). Drug holiday patterns and bisphosphonate-related osteonecrosis of the jaw. Oral Dis, 25(2), 471-480. doi:10.1111/odi.12966

Jurenka, J. S. (2008). Therapeutic applications of pomegranate (Punica granatum L.): a review. Altern Med Rev, 13(2), 128-144.

Kafadar, A., Erdinçler, P., & Erdinçler, U. D. S. (2006). Lhermitte-Duclos disease and Cowden's syndrome: Importance of the clinical association. Turkish Neurosurgery, 16(1), 25-29.

Kaiserling, E., Ruck, P., & Xiao, J. C. (1995). Congenital epulis and granular cell tumor: a histologic and immunohistochemical study. Oral Surg Oral Med Oral Pathol Oral Radiol Endod, 80(6), 687-697. doi:10.1016/s1079-2104(05)80253-7

Kajizono, M., Sada, H., Sugiura, Y., Soga, Y., Kitamura, Y., Matsuoka, J., & Sendo, T. (2015). Incidence and Risk Factors of Osteonecrosis of the Jaw in Advanced Cancer Patients after Treatment with Zoledronic Acid or Denosumab: A Retrospective Cohort Study. Biol Pharm Bull, 38(12), 1850-1855. doi:10.1248/bpb.b15-00385

Kallas, M., Green, F., Hewison, M., White, C., & Kline, G. (2010). Rare causes of calcitriol-mediated hypercalcemia: a case report and literature review. J Clin Endocrinol Metab, 95(7), 3111-3117. doi:10.1210/jc.2009-2673

Kamba, T., & McDonald, D. M. (2007). Mechanisms of adverse effects of anti-VEGF therapy for cancer. Br J Cancer, 96(12), 1788-1795. doi:10.1038/sj.bjc.6603813

Kamphuis, J. T., Buiting, A. G., Misere, J. F., van Berge Henegouwen, D. P., van Soolingen, D., & Rensma, P. L. (2001). BCG immunotherapy: be cautious of granulomas. Disseminated BCG infection and mycotic aneurysm as late complications of intravesical BCG instillations. Neth J Med, 58(2), 71-75. doi:10.1016/s0300-2977(00)00098-x

Kanakura, Y., Miyakawa, Y., Wilde, P., Smith, J., Achenbach, H., & Okamoto, S. (2014). Phase III, single-arm study investigating the efficacy, safety, and tolerability of anagrelide as a second-line treatment in high-risk Japanese patients with essential thrombocythemia. Int J Hematol, 100(4), 353-360. doi:10.1007/s12185-014-1631-x

Kannel, W. B. (2008). Challenges to consider in practicing specialized medicine. Ochsner J, 8(3), 105-107.

Kapoor, V. K., Dureja, J., & Chadha, R. (2009). Synthetic drugs with anti-ageing effects. Drug Discov Today, 14(17-18), 899-904. doi:10.1016/j.drudis.2009.07.006

Kashani, S., & Mearza, A. A. (2008). Uses and safety profile of ciclosporin in ophthalmology. Expert Opin Drug Saf, 7(1), 79-89. doi:10.1517/14740338.7.1.79

Kato, G. F., Lopes, R. N., Jaguar, G. C., Silva, A. P., & Alves, F. A. (2013). Evaluation of socket healing in patients undergoing bisphosphonate therapy: experience of a single Institution. Med Oral Patol Oral Cir Bucal, 18(4), e650-656. doi:10.4317/medoral.18787

Kavanagh, J. J., Levenback, C. F., Ramirez, P. T., Wolf, J. L., Moore, C. L., Jones, M. R., . . . Bast, R. C., Jr. (2010). Phase 2 study of canfosfamide in combination with pegylated liposomal doxorubicin in platinum and paclitaxel refractory or resistant epithelial ovarian cancer. J Hematol Oncol, 3, 9. doi:10.1186/1756-8722-3-9

Kawashima, A., Tsujimura, A., Takayama, H., Arai, Y., Nin, M., Tanigawa, G., . . . Osaka Renal Cell Carcinoma Clinical Study, C. (2012). Importance of continuing therapy and maintaining one-month relative dose intensity in sunitinib therapy for metastatic renal cell carcinoma. Med Oncol, 29(5), 3298-3305. doi:10.1007/s12032-012-0236-6

Kazlow Stern, D., Tripp, J. M., Ho, V. C., & Lebwohl, M. (2005). The use of systemic immune moderators in dermatology: an update. Dermatol Clin, 23(2), 259-300. doi:10.1016/j.det.2004.09.006

Kelleher, F. C., McKenna, M., Collins, C., Brady, G., Collins, I., & Crown, J. (2007). Bisphosphonate induced osteonecrosis of the jaws: unravelling uncertainty in disease causality. Acta Oncol, 46(5), 702-704. doi:10.1080/02841860600979021

Kempe, S., & Mader, K. (2012). In situ forming implants - an attractive formulation principle for parenteral depot formulations. J Control Release, 161(2), 668-679. doi:10.1016/j.jconrel.2012.04.016

Kennedy, A. (2004). Absorption and health effects of the Bowman-Birk inhibitor (BBI). Journal of Nutrition, 134(5), 1237S-1238S.

Kharkwal, G. B., Sharma, S. K., Huang, Y. Y., Dai, T., & Hamblin, M. R. (2011). Photodynamic therapy for infections: clinical applications. Lasers Surg Med, 43(7), 755-767. doi:10.1002/lsm.21080

Kii, I. (2019). Practical Application of Periostin as a Biomarker for Pathological Conditions. In Periostin (Vol. 1132, pp. 195-204).

Kim, C. J., Isono, T., Tambe, Y., Chano, T., Okabe, H., Okada, Y., & Inoue, H. (2008). Role of alternative splicing of periostin in human bladder carcinogenesis. Int J Oncol, 32(1), 161-169.

Kim, C. J., Tambe, Y., Mukaisho, K. I., Sugihara, H., Kageyama, S., Kawauchi, A., & Inoue, H. (2017). Periostin suppresses in vivo invasiveness via PDK1/Akt/mTOR signaling pathway in a mouse orthotopic model of bladder cancer. Oncol Lett, 13(6), 4276-4284. doi:10.3892/ol.2017.6004

Kim, E., Choe, C., Yoo, J. G., Oh, S. I., Jung, Y., Cho, A., . . . Do, Y. J. (2018). Major medical causes by breed and life stage for dogs presented at veterinary clinics in the Republic of Korea: a survey of electronic medical records. Peerj, 6, e5161. doi:10.7717/peerj.5161

Kim, S. J., Kang, H. J., Kim, J. S., Eom, H. S., Huh, J., Ko, Y. H., . . . Kim, W. S. (2013). A phase I study of everolimus and CHOP in newly diagnosed peripheral T-cell lymphomas. Invest New Drugs, 31(6), 1514-1521. doi:10.1007/s10637-013-0015-z

Kim, S. M., Myoung, H., Choung, P. H., Kim, M. J., Lee, S. K., & Lee, J. H. (2009). Metastatic leiomyosarcoma in the oral cavity: case report with protein expression profiles. J Craniomaxillofac Surg, 37(8), 454-460. doi:10.1016/j.jcms.2009.06.010

Kim, S. R., Jeon, H. J., Park, H. J., Kim, M. K., Choi, W. S., Jang, H. O., . . . Bae, M. K. (2013). Glycyrrhetinic acid inhibits Porphyromonas gingivalis lipopolysaccharide-induced vascular permeability via the suppression of interleukin-8. Inflamm Res, 62(2), 145-154. doi:10.1007/s00011-012-0560-5

King, L. (2019). Abstracts for the 43rd Human Genetics Society of Australasia Annual Scientific Meeting. Twin Res Hum Genet, 22(5), 371-418. doi:10.1017/thg.2019.81

Kitamura, M., Mochizuki, Y., Miyata, Y., Obata, Y., Mitsunari, K., Matsuo, T., . . . Sakai, H. (2019). Pathological Characteristics of Periodontal Disease in Patients with Chronic Kidney Disease and Kidney Transplantation. Int J Mol Sci, 20(14). doi:10.3390/ijms20143413

Kizub, D., Miao, J., Schubert, M. M., Paterson, A. H. G., Clemons, M. J., Dees, E. C., . . . et al. (2019). Factors associated with osteonecrosis of the jaw in women with breast cancer receiving high-dose bisphosphonates to prevent breast cancer metastases as part of the SWOG 0307 trial. Journal of Clinical Oncology, 37. doi:10.1200/JCO.2019.37.15-suppl.552

Klammt, J., Kobelt, L., Aktas, D., Durak, I., Gokbuget, A., Hughes, Q., . . . Schuster, V. (2011). Identification of three novel plasminogen (PLG) gene mutations in a series of 23 patients with low PLG activity. Thromb Haemost, 105(3), 454-460. doi:10.1160/TH10-04-0216

Klavdianou, K., Lazarini, A., Grivas, A., Tseronis, D., Tsalapaki, C., Rapsomaniki, P., . . . Vassilopoulos, D. (2020). Real Life Efficacy and Safety of Secukinumab in Biologic-Experienced Patients With Psoriatic Arthritis. Front Med (Lausanne), 7, 288. doi:10.3389/fmed.2020.00288

Kobayashi, K., Furukawa, A., Takahashi, M., & Murata, K. (2003). Neoadjuvant intra-arterial chemotherapy for locally advanced uterine cervical cancer: clinical efficacy and factors influencing response. Cardiovasc Intervent Radiol, 26(3), 234-241. doi:10.1007/s00270-003-0506-y

Kobos, R., & Bussel, J. B. (2008). Overview of thrombopoietic agents in the treatment of thrombocytopenia. Clin Lymphoma Myeloma, 8(1), 33-43. doi:10.3816/CLM.2008.n.002

Koganemaru, M., Abe, T., Anai, H., Tanaka, N., Nonoshita, M., Iwamoto, R., . . . Hayabuchi, N. (2012). A newly developed double lumen microballoon catheter with a side hole: initial experience of intraarterial infusion chemotherapy and/or embolization. Jpn J Radiol, 30(10), 870-874. doi:10.1007/s11604-012-0128-x

Kohli, M., Schiller, I., Dendukuri, N., Yao, M., Dheda, K., Denkinger, C. M., . . . Steingart, K. R. (2021). Xpert MTB/RIF Ultra and Xpert MTB/RIF assays for extrapulmonary tuberculosis and rifampicin resistance in adults. Cochrane Database Syst Rev, 1(1), CD012768. doi:10.1002/14651858.CD012768.pub3

Kokubo, Y., & Iwashima, Y. (2015). Higher blood pressure as a risk factor for diseases other than stroke and ischemic heart disease. Hypertension, 66(2), 254-259. doi:10.1161/HYPERTENSIONAHA.115.03480

Kolios, A. G., Yawalkar, N., Anliker, M., Boehncke, W. H., Borradori, L., Conrad, C., . . . Navarini, A. A. (2016). Swiss S1 Guidelines on the Systemic Treatment of Psoriasis Vulgaris. Dermatology, 232(4), 385-406. doi:10.1159/000445681

Konishi, T. (2017). Abstracts of the MASCC/ISOO 2017 Annual Meeting. Support Care Cancer, 25(2), 21-266. doi:10.1007/s00520-017-3704-x

Koopman, F. S., Beelen, A., Gilhus, N. E., de Visser, M., & Nollet, F. (2015). Treatment for postpolio syndrome. Cochrane Database Syst Rev, 2015(5), CD007818. doi:10.1002/14651858.CD007818.pub3

Kosem, M., Cankaya, H., & Kaya, Z. (2004). Choriocarcinoma metastatic to mandibular gingiva: case report and review of metastatic gingival tumours. J Otolaryngol, 33(5), 310-314. doi:10.2310/7070.2004.03094

Kramer, B., Kebschull, M., Nowak, M., Demmer, R. T., Haupt, M., Korner, C., . . . Papapanou, P. N. (2013). Role of the NK cell-activating receptor CRACC in periodontitis. Infect Immun, 81(3), 690-696. doi:10.1128/IAI.00895-12

Krauss, E., Rauthe, S., Gattenlohner, S., Reuther, T., Kochel, M., Kriegebaum, U., . . . Muller-Richter, U. D. (2011). MAGE-A antigens in lesions of the oral mucosa. Clin Oral Investig, 15(3), 315-320. doi:10.1007/s00784-010-0387-9

Kravchenko, J., Akushevich, I., Abernethy, A. P., & Lyerly, H. K. (2012). Evaluating the number of stages in development of squamous cell and adenocarcinomas across cancer sites using human population-based cancer modeling. PLoS One, 7(5), e37430. doi:10.1371/journal.pone.0037430

Krimmel, M., Ripperger, J., Hairass, M., Hoefert, S., Kluba, S., & Reinert, S. (2014). Does dental and oral health influence the development and course of bisphosphonate-related osteonecrosis of the jaws (BRONJ)? Oral Maxillofac Surg, 18(2), 213-218. doi:10.1007/s10006-013-0408-3

Krishnamurthy, K., Hoffman, C., & Del Priore, J. (2009). Bloodroot necrosis. Journal of the American Academy of Dermatology, 60(3), AB44. doi:10.1016/j.jaad.2008.11.213

Krishnan, A., Arslanoglu, A., Yildirm, N., Silbergleit, R., & Aygun, N. (2009). Imaging findings of bisphosphonate-related osteonecrosis of the jaw with emphasis on early magnetic resonance imaging findings. J Comput Assist Tomogr, 33(2), 298-304. doi:10.1097/RCT.0b013e31817e4986

Kruck, S., Hennenlotter, J., Amend, B., Geiger, M., Filipova, E., Neumann, T., . . . Bedke, J. (2017). Chronic Periodontitis Does Not Impact Serum Levels of Prostate-specific Antigen. Anticancer Res, 37(6), 3163-3167. doi:10.21873/anticanres.11675

Kuiper-Geertsma, D. G., & Derksen, R. H. (2003). Newer drugs for the treatment of lupus nephritis. Drugs, 63(2), 167-180. doi:10.2165/00003495-200363020-00004

Kültür, S., & Sami, S. N. (2009). Medicinal plants used in Isperih (Razgrad-Bulgaria) district. Turkish Journal of Pharmaceutical Sciences, 6(2), 107-124.

Kunnumakkara, A. B., Bordoloi, D., Padmavathi, G., Monisha, J., Roy, N. K., Prasad, S., & Aggarwal, B. B. (2017). Curcumin, the golden nutraceutical: multitargeting for multiple chronic diseases. Br J Pharmacol, 174(11), 1325-1348. doi:10.1111/bph.13621

Kuroe, A., Taniguchi, A., Sekiguchi, A., Ogura, M., Murayama, Y., Nishimura, F., . . . Nakai, Y. (2004). Prevalence of periodontal bacterial infection in non-obese Japanese type 2 diabetic patients: relationship with C-reactive protein and albuminuria. Horm Metab Res, 36(2), 116-118. doi:10.1055/s-2004-814221

Kurup, S. K., & Chan, C. C. (2005). Immunotherapeutic approaches in ocular inflammatory diseases. Arch Immunol Ther Exp (Warsz), 53(6), 484-496.

Kuypers, D. R., Malaise, J., Claes, K., Evenepoel, P., Maes, B., Coosemans, W., . . . Euro, S. P. K. S. G. (2005). Secondary effects of immunosuppressive drugs after simultaneous pancreas-kidney transplantation. Nephrol Dial Transplant, 20 Suppl 2(SUPPL. 2), ii33-39, ii62. doi:10.1093/ndt/gfh1080

Kwon, O. W., Lee, F. L., Chung, H., Lai, C. C., Sheu, S. J., Yoon, Y. H., & group, E. I. s. (2012). EXTEND III: efficacy and safety of ranibizumab in South Korean and Taiwanese patients with subfoveal CNV secondary to AMD. Graefes Arch Clin Exp Ophthalmol, 250(10), 1467-1476. doi:10.1007/s00417-012-1970-3

Kyrgidis, A., Koloutsos, G., & Vahtsevanos, K. (2010). Dental extractions in patients receiving bisphosphonate therapy. J Oral Maxillofac Surg, 68(7), 1704-1706; author reply 1706. doi:10.1016/j.joms.2010.01.028

La Starza, R., Nofrini, V., Pierini, T., Pierini, V., Zin, A., Bisogno, G., . . . Mecucci, C. (2015). Molecular Cytogenetics Detect an Unbalanced t(2;13)(q36;q14) and PAX3-FOXO1 Fusion in Rhabdomyosarcoma With Mixed Embryonal/Alveolar Features. Pediatr Blood Cancer, 62(12), 2238-2241. doi:10.1002/pbc.25664

Lai, J. N., Tang, J. L., & Wang, J. D. (2013). Observational studies on evaluating the safety and adverse effects of traditional chinese medicine. Evid Based Complement Alternat Med, 2013, 697893. doi:10.1155/2013/697893

Lajara, S., Balakrishnan, R., Castrodad, C., Colanta, A., & Khader, S. (2019). Abstracts : 31 (st) European Congress of Pathology. Virchows Arch, 475(Suppl 1), 1-436. doi:10.1007/s00428-019-02631-8

Lambert, V., Boylan, P., Boran, L., Hicks, P., Kirubakaran, R., Devane, D., & Matthews, A. (2020). Virtual reality distraction for acute pain in children. Cochrane Database Syst Rev, 10(10), CD010686. doi:10.1002/14651858.CD010686.pub2

Landesberg, R., Eisig, S., Fennoy, I., & Siris, E. (2009). Alternative indications for bisphosphonate therapy. J Oral Maxillofac Surg, 67(5 Suppl), 27-34. doi:10.1016/j.joms.2008.12.006

Langley, R. G., Tsai, T. F., Flavin, S., Song, M., Randazzo, B., Wasfi, Y., . . . Puig, L. (2018). Efficacy and safety of guselkumab in patients with psoriasis who have an inadequate response to ustekinumab: results of the randomized, double-blind, phase III NAVIGATE trial. Br J Dermatol, 178(1), 114-123. doi:10.1111/bjd.15750

Larsen, K. M., Minaya, M. K., Vaish, V., & Pena, M. M. O. (2018). The Role of IL-33/ST2 Pathway in Tumorigenesis. Int J Mol Sci, 19(9). doi:10.3390/ijms19092676

Larson, T., Nussenblatt, R. B., & Sen, H. N. (2011). Emerging drugs for uveitis. Expert Opin Emerg Drugs, 16(2), 309-322. doi:10.1517/14728214.2011.537824

Lau, C. S., Talug, J., Williams, S. B., Josephson, D. Y., Ruel, N. H., Chan, K. G., & Wilson, T. G. (2012). Robotic-assisted laparoscopic radical cystectomy in the octogenarian. Int J Med Robot, 8(2), 247-252. doi:10.1002/rcs.460

Laurindo, F. R., Pescatore, L. A., & Fernandes Dde, C. (2012). Protein disulfide isomerase in redox cell signaling and homeostasis. Free Radic Biol Med, 52(9), 1954-1969. doi:10.1016/j.freeradbiomed.2012.02.037

Lawitschka, A., Peters, C., Seidel, M. G., Havranek, A., Heitger, A., Fazekas, T., . . . Matthes-Martin, S. (2011). Long-term remission in pediatric Wegener granulomatosis following allo-SCT after reduced-intensity conditioning. Bone Marrow Transplant, 46(3), 462-463. doi:10.1038/bmt.2010.126

Lazarchik, D. A., & Haywood, V. B. (2010). Use of tray-applied 10 percent carbamide peroxide gels for improving oral health in patients with special-care needs. J Am Dent Assoc, 141(6), 639-646. doi:10.14219/jada.archive.2010.0251

Leal, J. B., & Carracho, J. L. (1968). [Considerations on dental treatments during pregnancy]. Rev Stomatol Chir Maxillofac, 69(8), 703-708.

Lee, J. H., Choi, J. K., Jeong, S. N., & Choi, S. H. (2018). Charlson comorbidity index as a predictor of periodontal disease in elderly participants. J Periodontal Implant Sci, 48(2), 92-102. doi:10.5051/jpis.2018.48.2.92

Lee, S., Chung, Y. J., Kim, B. H., Shim, J. H., Yoon, S. H., Shin, S. G., . . . Yu, K. S. (2009). Comparative pharmacokinetic evaluation of two formulations of bicalutamide 50-mg tablets: an open-label, randomized-sequence, single-dose, two-period crossover study in healthy Korean male volunteers. Clin Ther, 31(12), 3000-3008. doi:10.1016/j.clinthera.2009.12.004

Lee, W. Y., Asadujjaman, M., & Jee, J.-P. (2019). Long acting injectable formulations: the state of the arts and challenges of poly(lactic-co-glycolic acid) microsphere, hydrogel, organogel and liquid crystal. Journal of Pharmaceutical Investigation, 49(4), 459-476. doi:10.1007/s40005-019-00449-9

Lei, J., Nowbar, S., Mariash, C. N., & Ingbar, D. H. (2003). Thyroid hormone stimulates Na-K-ATPase activity and its plasma membrane insertion in rat alveolar epithelial cells. Am J Physiol Lung Cell Mol Physiol, 285(3), L762-772. doi:10.1152/ajplung.00376.2002

Lelievre, L., Clezardin, P., Magaud, L., Roche, L., Tubiana-Mathieu, N., Tigaud, J. D., . . . Mathevet, P. (2018). Comparative Study of Neoadjuvant Chemotherapy With and Without Zometa for Management of Locally Advanced Breast Cancer With Serum VEGF as Primary Endpoint: The NEOZOL Study. Clin Breast Cancer, 18(6), e1311-e1321. doi:10.1016/j.clbc.2018.07.005

Lenoir-Wijnkoop, I., Sanders, M. E., Cabana, M. D., Caglar, E., Corthier, G., Rayes, N., . . . Wolvers, D. A. (2007). Probiotic and prebiotic influence beyond the intestinal tract. Nutr Rev, 65(11), 469-489. doi:10.1111/j.1753-4887.2007.tb00272.x

LeRoith, D. (2011). Hormones and cancer: breast and prostate. Endocrinol Metab Clin North Am, 40(3), xiii-xvi. doi:10.1016/j.ecl.2011.05.015

Lester, P. A., Dysko, R. C., & Nemzek, J. A. (2004). What is your diagnosis? Uterine leiomyoma. J Am Vet Med Assoc, 225(8), 1179-1180. doi:10.2460/javma.2004.225.1179

Levin, L., & Luder, L. (2013). Jaw lesion and discomfort. Jama, 310(20), 2195-2196. doi:10.1001/jama.2013.282405

Lewis, A. L., & Illum, L. (2010). Formulation strategies for sustained release of proteins. Ther Deliv, 1(3), 457-479. doi:10.4155/tde.10.17

Li, S. (2011). Gingival bleeding as a presenting sign of primary fibrinogenolysis. Oral Surg Oral Med Oral Pathol Oral Radiol Endod, 112(1), 3. doi:10.1016/j.tripleo.2011.01.044

Li, W., Saji, S., Sato, F., Noda, M., & Toi, M. (2013). Potential clinical applications of matrix metalloproteinase inhibitors and their future prospects. Int J Biol Markers, 28(2), 117-130. doi:10.5301/jbm.5000026

Lia, N. G., Shib, Z. H., Tang, Y. P., & Duan, J. A. (2009). Selective matrix metalloproteinase inhibitors for cancer. Curr Med Chem, 16(29), 3805-3827. doi:10.2174/092986709789178037

Liang, G., Wang, H., Shi, H., Zhu, M., An, J., Qi, Y., . . . Gao, S. (2020). Porphyromonas gingivalis Promotes the Proliferation and Migration of Esophageal Squamous Cell Carcinoma through the miR-194/GRHL3/PTEN/Akt Axis. ACS Infect Dis, 6(5), 871-881. doi:10.1021/acsinfecdis.0c00007

Liang, Z., Xie, W., Wu, R., Geng, H., Zhao, L., Xie, C., . . . Han, H. (2015). Inhibition of tobacco smoke-induced bladder MAPK activation and epithelial-mesenchymal transition in mice by curcumin. Int J Clin Exp Pathol, 8(5), 4503-4513.

Liao, Y., Luo, D., Peng, K., & Zeng, Y. (2021). Cyclophilin A: a key player for etiological agent infection. Appl Microbiol Biotechnol, 105(4), 1365-1377. doi:10.1007/s00253-021-11115-2

Licciardi, P. V., Kwa, F. A., Ververis, K., Di Costanzo, N., Balcerczyk, A., Tang, M. L., . . . Karagiannis, T. C. (2012). Influence of natural and synthetic histone deacetylase inhibitors on chromatin. Antioxid Redox Signal, 17(2), 340-354. doi:10.1089/ars.2011.4480

Lichtenstein, G. R., Abreu, M. T., Cohen, R., Tremaine, W., & American Gastroenterological, A. (2006). American Gastroenterological Association Institute technical review on corticosteroids, immunomodulators, and infliximab in inflammatory bowel disease. Gastroenterology, 130(3), 940-987. doi:10.1053/j.gastro.2006.01.048

Lidgi, S., Embon, O. M., Turani, H., & Sazbon, A. I. (1989). Giant cell reparative granuloma of the bladder associated with transitional cell carcinoma. J Urol, 142(1), 120-122. doi:10.1016/s0022-5347(17)38680-9

Lin, C. T., Huang, W. N., Hsieh, C. W., Chen, Y. M., Chen, D. Y., Hsieh, T. Y., & Chen, Y. H. (2019). Safety and effectiveness of tocilizumab in treating patients with rheumatoid arthritis - A three-year study in Taiwan. J Microbiol Immunol Infect, 52(1), 141-150. doi:10.1016/j.jmii.2017.04.002

Lin, S. Y., Lin, C. L., Chang, C. H., Wu, H. C., Chen, W. C., Wang, I. K., . . . Kao, C. H. (2017). Comparative risk of chronic kidney diseases in patients with urolithiasis and urological interventions: a longitudinal population-based study. Urolithiasis, 45(5), 465-472. doi:10.1007/s00240-016-0929-y

Lira, S. C. (2007). Bisphosphonate-associated mandibular osteonecrosis. Ginecologia y Obstetricia de Mexico, 75(11), 655-660.

Little, J. W. (2000). The impact on dentistry of recent advances in the management of hypertension. Oral Surg Oral Med Oral Pathol Oral Radiol Endod, 90(5), 591-599. doi:10.1067/moe.2000.109517

Liu, J., & Duan, Y. (2012). Saliva: a potential media for disease diagnostics and monitoring. Oral Oncol, 48(7), 569-577. doi:10.1016/j.oraloncology.2012.01.021

Liu, J., Lu, Y., Wu, Q., Goyer, R. A., & Waalkes, M. P. (2008). Mineral arsenicals in traditional medicines: orpiment, realgar, and arsenolite. J Pharmacol Exp Ther, 326(2), 363-368. doi:10.1124/jpet.108.139543

Liu, K., Meng, H., & Hou, J. (2012). Activity of 25-hydroxylase in human gingival fibroblasts and periodontal ligament cells. PLoS One, 7(12), e52053. doi:10.1371/journal.pone.0052053

Liu, R. S., Liu, H. C., Bu, J. Q., & Dong, S. N. (2000). Burkitt's lymphoma presenting with jaw lesions. J Periodontol, 71(4), 646-649. doi:10.1902/jop.2000.71.4.646

Lodi, G., Azzi, L., Varoni, E. M., Pentenero, M., Del Fabbro, M., Carrassi, A., . . . Manfredi, M. (2021). Antibiotics to prevent complications following tooth extractions. Cochrane Database Syst Rev, 2(2), CD003811. doi:10.1002/14651858.CD003811.pub3

Lohler, J., Gerstner, A. O., Bootz, F., & Walther, L. E. (2014). Incidence and localization of abnormal mucosa findings in patients consulting ENT outpatient clinics and data analysis of a cancer registry. Eur Arch Otorhinolaryngol, 271(5), 1289-1297. doi:10.1007/s00405-013-2738-z

Lorenzo-Pouso, A. I., Perez-Sayans, M., Chamorro-Petronacci, C., Gandara-Vila, P., Lopez-Jornet, P., Carballo, J., & Garcia-Garcia, A. (2020). Association between periodontitis and medication-related osteonecrosis of the jaw: A systematic review and meta-analysis. J Oral Pathol Med, 49(3), 190-200. doi:10.1111/jop.12963

Lorusso, D., Maltese, G., Sabatucci, I., Cresta, S., Matteo, C., Ceruti, T., . . . de Braud, F. (2021). Phase I Study of Rucaparib in Combination with Bevacizumab in Ovarian Cancer Patients: Maximum Tolerated Dose and Pharmacokinetic Profile. Target Oncol, 16(1), 59-68. doi:10.1007/s11523-020-00780-4

Lotti, T., Agarwal, K., Podder, I., Satolli, F., Kassir, M., Schwartz, R. A., . . . Goldust, M. (2020). Safety of the current drug treatments for vitiligo. Expert Opin Drug Saf, 19(4), 499-511. doi:10.1080/14740338.2020.1729737

Love, P. B., Patterson, S. S., Prose, N. S., & Atwater, A. R. (2012). Adverse cutaneous reactions to chemotherapy agents. Journal of Drugs in Dermatology, 11(9), 1120-1121.

Lovett, E., & Ganta, N. (2010). Advising patients about herbs and nutraceuticals: tips for primary care providers. Prim Care, 37(1), 13-30. doi:10.1016/j.pop.2009.09.007

Lowenstine, L. J., McManamon, R., & Terio, K. A. (2016). Comparative Pathology of Aging Great Apes: Bonobos, Chimpanzees, Gorillas, and Orangutans. Vet Pathol, 53(2), 250-276. doi:10.1177/0300985815612154

Lu, H. K., Tseng, C. C., Lee, Y. H., Li, C. L., & Wang, L. F. (2010). Flutamide inhibits nifedipine- and interleukin-1 beta-induced collagen overproduction in gingival fibroblasts. J Periodontal Res, 45(4), 451-457. doi:10.1111/j.1600-0765.2009.01255.x

Luk, W. S. (2004). The HRQoL of renal transplant patients. J Clin Nurs, 13(2), 201-209. doi:10.1046/j.1365-2702.2003.00867.x

Lustig, M. J., & Cunningham, E. T., Jr. (2003). Use of immunosuppressive agents in uveitis. Curr Opin Ophthalmol, 14(6), 399-412. doi:10.1097/00055735-200312000-00014

M. Neuberger, J. (2003). Liver transplantation. Best Practice & Research Clinical Gastroenterology, 17(2), 277-289. doi:10.1016/s1521-6918(02)00144-0

Ma, K., Hong, B., Zhou, J., Gong, Y., Wang, J., Liu, S., . . . Gong, K. (2019). The Efficacy and Safety of Tyrosine Kinase Inhibitors for Von Hippel-Lindau Disease: A Retrospective Study of 32 Patients. Front Oncol, 9(4), 1122. doi:10.3389/fonc.2019.01122

Macey, R., Walsh, T., Riley, P., Glenny, A. M., Worthington, H. V., Fee, P. A., . . . Ricketts, D. (2020). Fluorescence devices for the detection of dental caries. Cochrane Database Syst Rev, 12(12), CD013811. doi:10.1002/14651858.CD013811

Machorowska-Pieniazek, A., Morawiec, T., Mertas, A., Tanasiewicz, M., Dziedzic, A., & Krol, W. (2013). Influence of propolis on hygiene, gingival condition, and oral microflora in patients with cleft lip and palate treated with fixed orthodontic appliances. Evid Based Complement Alternat Med, 2013, 183915. doi:10.1155/2013/183915

Madrid, C., Bouferrache, K., Abarca, M., Jaques, B., & Broome, M. (2010). Bisphosphonate-related osteonecrosis of the jaws: how to manage cancer patients. Oral Oncol, 46(6), 468-470. doi:10.1016/j.oraloncology.2010.03.016

Maestre-Rodriguez, O., Gonzalez-Garcia, R., Mateo-Arias, J., Moreno-Garcia, C., Serrano-Gil, H., Villanueva-Alcojol, L., . . . Monje-Gil, F. (2009). Metastasis of renal clear-cell carcinoma to the oral mucosa, an atypical location. Med Oral Patol Oral Cir Bucal, 14(11), e601-604. doi:10.4317/medoral.14.e601

Magden, E., Quackenbush, S. L., & VandeWoude, S. (2011). FIV associated neoplasms--a mini-review. Vet Immunol Immunopathol, 143(3-4), 227-234. doi:10.1016/j.vetimm.2011.06.016

Magremanne, M. (2008). [Osteoporosis, bisphosphonates and jaws osteochemonecrosis]. Rev Med Brux, 29(4), 262-266.

Magro, G., Brancato, F., Musumeci, G., Alaggio, R., Parenti, R., & Salvatorelli, L. (2015). Cyclin D1 is a useful marker for soft tissue Ewing's sarcoma/peripheral Primitive Neuroectodermal Tumor in children and adolescents: A comparative immunohistochemical study with rhabdomyosarcoma. Acta Histochem, 117(4-5), 460-467. doi:10.1016/j.acthis.2015.01.005

Magulick, J. P., Paredes, A. H., Ringdahl, S., & Chong, C. H. (2018). The Relationship between Non-Alcoholic Fatty Liver Disease and Periodontal Disease. Gastroenterology, 154(6), S1170-S1170.

Majumder, P. D., Sudharshan, S., & Biswas, J. (2009). Anti-inflammatory therapy in uveitis. Recent Pat Inflamm Allergy Drug Discov, 3(3), 188-194. doi:10.2174/187221309789257405

Makos, C. P., & Psomaderis, K. (2009). A literature review in renal carcinoma metastasis to the oral mucosa and a new report of an epulis-like metastasis. J Oral Maxillofac Surg, 67(3), 653-660. doi:10.1016/j.joms.2008.10.006

Malathi, N., Mythili, S., & Vasanthi, H. R. (2014). Salivary diagnostics: a brief review. ISRN Dent, 2014, 158786. doi:10.1155/2014/158786

Mali, S. B. (2014). Proteomics for oral cancer. Oral Oncol, 50(11), e67. doi:10.1016/j.oraloncology.2014.09.007

Manfredi, M., Mergoni, G., Goldoni, M., Salvagni, S., Merigo, E., Meleti, M., & Vescovi, P. (2017). A 5-year retrospective longitudinal study on the incidence and the risk factors of osteonecrosis of the jaws in patients treated with zoledronic acid for bone metastases from solid tumors. Med Oral Patol Oral Cir Bucal, 22(3), e342-e348. doi:10.4317/medoral.21728

Manika, Neelkamal, Sankireddy, S., Mittal, S., & Vinayak, V. (2015). Curcumin - a Solid Gold in Medicine and Dentistry. Indian Journal of Public Health Research & Development, 6(3), 78-82. doi:10.5958/0976-5506.2015.00143.6

Manna, R., Cadoni, G., Ferri, E., Verrecchia, E., Giovinale, M., Fonnesu, C., . . . Paludetti, G. (2008). Wegener's granulomatosis: an update on diagnosis and therapy. Expert Rev Clin Immunol, 4(4), 481-495. doi:10.1586/1744666X.4.4.481

Manohar, B., Verma, N., Mannan, N., & Bhuvaneshwari, S. (2020). Adenomatoid odontogenic tumor mimicking a lateral periodontal cyst - A rare case report in the mandible. J Indian Soc Periodontol, 24(5), 473-476. doi:10.4103/jisp.jisp_79_20

Mapelli, P., Mangili, G., Picchio, M., Rabaiotti, E., Gianolli, L., Messa, C., & Candiani, M. (2013). Sarcoidosis mimicking metastatic gynaecological malignancies: a diagnostic and therapeutic challenge? Rev Esp Med Nucl Imagen Mol, 32(5), 314-317. doi:10.1016/j.remn.2012.11.001

Marais, D. J., Sampson, C., Jeftha, A., Dhaya, D., Passmore, J. A., Denny, L., . . . Williamson, A. L. (2006). More men than women make mucosal IgA antibodies to Human papillomavirus type 16 (HPV-16) and HPV-18: a study of oral HPV and oral HPV antibodies in a normal healthy population. BMC Infect Dis, 6, 95. doi:10.1186/1471-2334-6-95

Marcard, T., Ivens, K., Grabensee, B., Willers, R., Helmchen, U., Rump, L. C., & Blume, C. (2008). Early conversion from cyclosporine to tacrolimus increases renal graft function in chronic allograft nephropathy at BANFF stages I and II. Transpl Int, 21(12), 1153-1162. doi:10.1111/j.1432-2277.2008.00731.x

Marder, W., & McCune, W. J. (2007). Advances in immunosuppressive therapy. Semin Respir Crit Care Med, 28(4), 398-417. doi:10.1055/s-2007-985612

Margadant, C., Kreft, M., Zambruno, G., & Sonnenberg, A. (2013). Kindlin-1 regulates integrin dynamics and adhesion turnover. PLoS One, 8(6), e65341. doi:10.1371/journal.pone.0065341

Martin, J. Y., Urban, R. R., Liao, J. B., & Goff, B. A. (2016). Bevacizumab toxicity in heavily pretreated recurrent epithelial ovarian, fallopian tube, and primary peritoneal cancers. J Gynecol Oncol, 27(5), e47. doi:10.3802/jgo.2016.27.e47

Martinez Conde, R., Lopez Cedrun, J. L., Aguirre Urizar, J. M., Rosell Cerro, M., & Llarena Ibarguren, R. (1990). [Metastatic hypernephroma in oral soft tissue]. Av Odontoestomatol, 6(5), 280-281, 284-285.

Martinez Moragon, E., Aparicio Urtasun, J., Sanchis Aldas, J., Rogado Gonzalez, M. C., de Diego Damia, A., & Perpina Tordera, M. (1994). [Endobronchial metastasis. Clinical aspects, diagnosis and course in a series of 27 cases]. Rev Clin Esp, 194(12), 1013-1017.

Martini, G., & Zulian, F. (2006). Juvenile idiopathic arthritis: current and future treatment options. Expert Opin Pharmacother, 7(4), 387-399. doi:10.1517/14656566.7.4.387

Marwaha, A. K., Morris, J. A., & Rigby, R. J. (2020). Hypothesis: Bacterial induced inflammation disrupts the orderly progression of the stem cell hierarchy and has a role in the pathogenesis of breast cancer. Med Hypotheses, 136, 109530. doi:10.1016/j.mehy.2019.109530

Marx, R. E. (2009). Reconstruction of defects caused by bisphosphonate-induced osteonecrosis of the jaws. J Oral Maxillofac Surg, 67(5 Suppl), 107-119. doi:10.1016/j.joms.2008.12.007

Marx, R. E., Sawatari, Y., Fortin, M., & Broumand, V. (2005). Bisphosphonate-induced exposed bone (osteonecrosis/osteopetrosis) of the jaws: risk factors, recognition, prevention, and treatment. J Oral Maxillofac Surg, 63(11), 1567-1575. doi:10.1016/j.joms.2005.07.010

Mas, A., & Mascaro, J. M., Jr. (2010). Chronic indurated gingival ulceration. Bisphosphonate-related osteonecrosis of the jaw (BRONJ). Arch Dermatol, 146(11), 1301-1306. doi:10.1001/archdermatol.2010.318-a

Massey, D. (2005). Potential pitfalls in diagnostic oral pathology: a review for the general surgical pathologist. Adv Anat Pathol, 12(6), 332-349. doi:10.1097/01.pap.0000194631.43254.00

Mata, X., Renaud, G., & Mollereau, C. (2019). The repertoire of family A-peptide GPCRs in archaic hominins. Peptides, 122, 170154. doi:10.1016/j.peptides.2019.170154

Mattheos, N., Caldwell, P., Petcu, E. B., Ivanovski, S., & Reher, P. (2013). Dental implant placement with bone augmentation in a patient who received intravenous bisphosphonate treatment for osteoporosis. J Can Dent Assoc, 79, d2.

Mawardi, H., Treister, N., Richardson, P., Anderson, K., Munshi, N., Faiella, R. A., & Woo, S. B. (2009). Sinus tracts--an early sign of bisphosphonate-associated osteonecrosis of the jaws? J Oral Maxillofac Surg, 67(3), 593-601. doi:10.1016/j.joms.2008.09.031

McCully, K. S. (2017). Hyperhomocysteinemia, Suppressed Immunity, and Altered Oxidative Metabolism Caused by Pathogenic Microbes in Atherosclerosis and Dementia. Front Aging Neurosci, 9(OCT), 324. doi:10.3389/fnagi.2017.00324

McManus, R., BoAbbas, Y., Lampe, H., Holliday, R., & Shum, D. (2000). Maxillary giant cell granuloma, pheochromocytoma, and hyperparathyroidism without medullary thyroid carcinoma. Ear Nose Throat J, 79(8), 590-593.

Medina, B. R., Barba, E. M., Torres, A. V., & Trujillo, S. M. (2001). Gingival metastases as first sign of a primary uterine angiosarcoma. J Oral Maxillofac Surg, 59(4), 467-471. doi:10.1053/joms.2001.21892

Mehdi, I., Al Bahrani, B. J., Al Lawati, T. M., Al Mandhari, Z., & Al Lawati, F. R. (2017). Breast cancer in a patient with Kindler's syndrome. Journal of the Pakistan Medical Association, 67(8), 1283-1286.

Melero, I., Grimaldi, A. M., Perez-Gracia, J. L., & Ascierto, P. A. (2013). Clinical development of immunostimulatory monoclonal antibodies and opportunities for combination. Clin Cancer Res, 19(5), 997-1008. doi:10.1158/1078-0432.CCR-12-2214

Micali, G., Nasca, M. R., Innocenzi, D., Frasin, L. A., Radi, O., Parma, P., . . . Schwartz, R. A. (2005). Association of palmoplantar keratoderma, cutaneous squamous cell carcinoma, dental anomalies, and hypogenitalism in four siblings with 46,XX karyotype: a new syndrome. J Am Acad Dermatol, 53(5 Suppl 1), S234-239. doi:10.1016/j.jaad.2005.02.033

Michelet, N., Spenatto, N., Viraben, R., Cuny, J. F., Mazet, J., Trechot, P., . . . Schmutz, J. L. (2008). [BCG infection of the glans penis after intravesical BCG therapy]. Ann Dermatol Venereol, 135(6-7), 479-483. doi:10.1016/j.annder.2007.06.012

Migliorati, C. A., Siegel, M. A., & Elting, L. S. (2006). Bisphosphonate-associated osteonecrosis: a long-term complication of bisphosphonate treatment. Lancet Oncol, 7(6), 508-514. doi:10.1016/S1470-2045(06)70726-4

Misaka, K., Kishimoto, T., Kawahigashi, Y., Sata, M., & Nagao, Y. (2016). Use of Direct-Acting Antivirals for the Treatment of Hepatitis C Virus-Associated Oral Lichen Planus: A Case Report. Case Rep Gastroenterol, 10(3), 617-622. doi:10.1159/000450679

Mitra, A., & Wu, Y. (2010). Use of In Vitro-In Vivo Correlation (IVIVC) to facilitate the development of polymer-based controlled release injectable formulation. Recent Pat Drug Deliv Formul, 4(2), 94-104. doi:10.2174/187221110791185024

Mizuno, H., Koya, J., Fujioka, Y., Ibaraki, T., Nakamura, F., Hayashi, A., . . . Kurokawa, M. (2017). Extranodal NK/T cell lymphoma in a living donor liver transplant recipient. Ann Hematol, 96(6), 1051-1052. doi:10.1007/s00277-017-2969-y

Mizuno, S., Hayasaki, A., Ito, T., Fujii, T., Iizawa, Y., Kato, H., . . . Isaji, S. (2018). De Novo Malignancy Following Adult-to-Adult Living Donor Liver Transplantation Focusing on Posttransplantation Lymphoproliferative Disorder. Transplant Proc, 50(9), 2699-2704. doi:10.1016/j.transproceed.2018.03.059

Mizuno, S., Iizawa, Y., Kato, H., Murata, Y., Tanemura, A., Kuriyama, N., . . . Isaji, S. (2017). Poster Abstracts. American journal of transplantation, 17, 411-815. doi:10.1111/ajt.14306

Mizuno, T., Ishikawa, K., Sato, W., Koike, T., Kushida, M., Miyagawa, Y., . . . Noda, Y. (2013). The risk factors of severe acute kidney injury induced by cisplatin. Oncology, 85(6), 364-369. doi:10.1159/000356587

Mobarki, M., Dumollard, J. M., Dal Col, P., Camy, F., Peoc'h, M., & Karpathiou, G. (2020). Granular cell tumor a study of 42 cases and systemic review of the literature. Pathol Res Pract, 216(4), 152865. doi:10.1016/j.prp.2020.152865

Moghaddasi, M. S. (2010). Saffron chemicals and medicine usage. Journal of Medicinal Plants Research, 4(6), 427-430.

Mok, C. C. (2006). Emerging drug therapies for systemic lupus erythematosus. Expert Opin Emerg Drugs, 11(4), 597-608. doi:10.1517/14728214.11.4.597

Mok, C. C. (2007). Management of systemic lupus erythematosus in Chinese patients. Expert Rev Clin Immunol, 3(6), 925-935. doi:10.1586/1744666X.3.6.925

Molina, J. M., Rabian, C., D'Agay, M. F., & Modai, J. (1992). Hypersensitivity systemic reaction following intravesical bacillus Calmette-Guerin: successful treatment with steroids. J Urol, 147(3), 695-697. doi:10.1016/s0022-5347(17)37354-8

Mondal, S., Adhikari, N., Banerjee, S., Amin, S. A., & Jha, T. (2020). Matrix metalloproteinase-9 (MMP-9) and its inhibitors in cancer: A minireview. Eur J Med Chem, 194, 112260. doi:10.1016/j.ejmech.2020.112260

Montebugnoli, L., Checchi, L., & Marangolo, M. (1991). [Lesions of the oral cavity during antineoplastic therapy]. Dent Cadmos, 59(7), 62-67.

Morawiec, T., Dziedzic, A., Niedzielska, I., Mertas, A., Tanasiewicz, M., Skaba, D., . . . Wieckiewicz, M. (2013). The biological activity of propolis-containing toothpaste on oral health environment in patients who underwent implant-supported prosthodontic rehabilitation. Evid Based Complement Alternat Med, 2013, 704947. doi:10.1155/2013/704947

Morgan, R. D., Hannon, E., & Lakhoo, K. (2011). Renal abscess in Papillion-Lefevre syndrome. Pediatr Surg Int, 27(12), 1381-1383. doi:10.1007/s00383-011-2931-3

Mori, K., Horinouchi, M., Domitsu, A., Shimotahira, T., Soutome, S., Yamaguchi, T., & Oho, T. (2017). Proper oral hygiene protocols decreased inflammation of gingivitis in a patient during chemotherapy with bevacizumab: a case report. Clin Case Rep, 5(8), 1352-1357. doi:10.1002/ccr3.1034

Mori, M., Amano, Y., Sakamoto, M., & Kimura, J. (1974). [A case of metastatic choriocarcinoma of the gingiva (author's transl)]. Nihon Koku Geka Gakkai Zasshi, 20(6), 621-626.

Mori, M., Sakamoto, M., Amano, Y., & Kimura, J. (1974). [Renal carcinoma with metastasis to gingiva: a review of the literature with report of a case (author's transl)]. Nihon Koku Geka Gakkai Zasshi, 20(6), 614-620. doi:10.5794/jjoms.20.614

Morra, L., Rechsteiner, M., Casagrande, S., Duc Luu, V., Santimaria, R., Diener, P. A., . . . Soltermann, A. (2011). Relevance of periostin splice variants in renal cell carcinoma. Am J Pathol, 179(3), 1513-1521. doi:10.1016/j.ajpath.2011.05.035

Morris, J. C., Tan, A. R., Olencki, T. E., Shapiro, G. I., Dezube, B. J., Reiss, M., . . . Lawrence, D. P. (2014). Phase I study of GC1008 (fresolimumab): a human anti-transforming growth factor-beta (TGFbeta) monoclonal antibody in patients with advanced malignant melanoma or renal cell carcinoma. PLoS One, 9(3), e90353. doi:10.1371/journal.pone.0090353

Mourad, G., Karras, A., Kamar, N., Garrigue, V., Legendre, C., Lefrancois, N., . . . French Myriade, F. R. S. G. (2007). Renal function with delayed or immediate cyclosporine microemulsion in combination with enteric-coated mycophenolate sodium and steroids: results of follow up to 30 months post-transplant. Clin Transplant, 21(3), 295-300. doi:10.1111/j.1399-0012.2007.00660.x

Mousa, H. A. (2016). Health Effects of Alkaline Diet and Water, Reduction of Digestive-tract Bacterial Load, and Earthing. Altern Ther Health Med, 22 Suppl 1, 24-33.

Moyad, M. A. (2003). The potential benefits of dietary and/or supplemental calcium and vitamin D. Urologic Oncology: Seminars and Original Investigations, 21(5), 384-391. doi:10.1016/s1078-1439(03)00108-x

Mukherjee, S., Babu, N. A., Rajesh, E., & Masthan, K. M. K. (2019). Complications of Bisphosphonate therapy in Dentistry. Indian Journal of Public Health Research & Development, 10(11), 3212-3217. doi:10.5958/0976-5506.2019.04412.7

Muller-Mattheis, V., Hagen, M., Frenzel, H., & Ackermann, R. (1989). [A rare form of metastasis of renal cell cancer. A case report of intra-oral soft tissue metastasis]. Urologe A, 28(6), 355-358.

Munakata, R., Sawair, F. A., Cheng, J., & Saku, T. (2009). Gingival metastasis of ovarian carcinoma: report of a case and review of the literature. Int J Oral Maxillofac Surg, 38(10), 1123-1126. doi:10.1016/j.ijom.2009.05.003

Mundargi, R. C., Babu, V. R., Rangaswamy, V., Patel, P., & Aminabhavi, T. M. (2008). Nano/micro technologies for delivering macromolecular therapeutics using poly(D,L-lactide-co-glycolide) and its derivatives. J Control Release, 125(3), 193-209. doi:10.1016/j.jconrel.2007.09.013

Muniraj, N., Siddharth, S., & Sharma, D. (2019). Bioactive Compounds: Multi-Targeting Silver Bullets for Preventing and Treating Breast Cancer. Cancers (Basel), 11(10). doi:10.3390/cancers11101563

Murai, A., Abou Asa, S., Kodama, A., Sakai, H., Hirata, A., & Yanai, T. (2012). Immunohistochemical analysis of the Akt/mTOR/4E-BP1 signalling pathway in canine haemangiomas and haemangiosarcomas. J Comp Pathol, 147(4), 430-440. doi:10.1016/j.jcpa.2012.05.002

Murata, M., Ikeda, Y., Hasegawa, G., Nakagawa, Y., & Nishiyama, T. (2019). Low-dose axitinib rechallenge with positive outcomes in a patient with metastatic renal cell carcinoma refractory to interferon alpha, sunitinib, axitinib, and nivolumab therapies: a case report. J Med Case Rep, 13(1), 98. doi:10.1186/s13256-019-2041-8

Murgod, S., Girish, H. C., Shyamala, K., & Savita, J. K. (2015). Metastatic tumors of the oral region: A review. Research Journal of Pharmaceutical, Biological and Chemical Sciences, 6(4), 1773-1779.

Murillo, J., Bagan, J. V., Hens, E., Diaz, J. M., & Leopoldo, M. (2013). Tumors metastasizing to the oral cavity: a study of 16 cases. J Oral Maxillofac Surg, 71(9), 1545-1551. doi:10.1016/j.joms.2013.03.017

Murphy, K. M., & Vikram, H. R. (2019). Heart transplantation for infective endocarditis: Viable option for a limited few? Transpl Infect Dis, 21(1), e13006. doi:10.1111/tid.13006

Murray, C. J., Atkinson, C., Bhalla, K., Birbeck, G., Burstein, R., Chou, D., . . . Collaborators, U. S. B. o. D. (2013). The state of US health, 1990-2010: burden of diseases, injuries, and risk factors. Jama, 310(6), 591-608. doi:10.1001/jama.2013.13805

Nagasawa, Y., Nomura, R., Misaki, T., Naka, S., Ito, S., Wato, K., . . . Nakano, K. (2019). Prevalence of Peridontal Disease Bacteria in Tonsils of Iga Nephropathy Patients. Nephrology Dialysis Transplantation, 34.

Nagi, R., Yashoda Devi, B. K., Rakesh, N., Reddy, S. S., & Patil, D. J. (2015). Clinical implications of prescribing nonsteroidal anti-inflammatory drugs in oral health care--a review. Oral Surg Oral Med Oral Pathol Oral Radiol, 119(3), 264-271. doi:10.1016/j.oooo.2014.12.002

Nakanishi, M., Yamate, J., Nakatsuji, S., Ide, M., Sawamoto, O., Kuwamura, M., . . . Sakuma, S. (2002). Establishment of a transplantable tumor line (IP) derived from rat pulmonary carcinoma, developing humoral hypercalcemia of malignancy in IP-bearing rats. Virchows Arch, 440(2), 195-204. doi:10.1007/s004280100484

Nakashima, M., & Akamine, A. (2005). The application of tissue engineering to regeneration of pulp and dentin in endodontics. J Endod, 31(10), 711-718. doi:10.1097/01.don.0000164138.49923.e5

Naz, H., Aslan, L., Sonmez Tamer, G., & Naz, C. (2018). [Case of pneumonia associated sepsis accompaning pulmonary myiasis]. Mikrobiyol Bul, 52(4), 439-443. doi:10.5578/mb.67395

Nct. (2004). MOTOR: maternal Oral Therapy to Reduce Obstetric Risk. https://clinicaltrials.gov/show/NCT00097656.

Nct. (2014). Alternative Antibiotic Regime in the Treatment of GAgP. https://clinicaltrials.gov/show/NCT02223702.

Nct. (2015). Non-surgical Periodontal Therapy and Myo-inositol in Polycystic Ovary Syndrome Women Having Chronic Periodontitis. https://clinicaltrials.gov/show/NCT02633462.

Ndukwe, K. C., Fatusi, O. A., & Ugboko, V. I. (2002). Craniocervical necrotizing fasciitis in Ile-Ife, Nigeria. Br J Oral Maxillofac Surg, 40(1), 64-67. doi:10.1054/bjom.2001.0715

Neki, N. S. (2011). Tobacco and health. Journal International Medical Sciences Academy, 24(2), 51-52.

Nelson, D. A., Bradley, J. K., Arya, R., Ianosi-Irimie, M., Marques-Baptista, A., & Merlin, M. A. (2009). Babesiosis as a rare cause of fever in the immunocompromised patient: a case report. Cases J, 2(7), 7420. doi:10.4076/1757-1626-2-7420

Newnham, J. P., Dickinson, J. E., Hart, R. J., Pennell, C. E., Arrese, C. A., & Keelan, J. A. (2014). Strategies to prevent preterm birth. Front Immunol, 5(NOV), 584. doi:10.3389/fimmu.2014.00584

Ngwarai, M. R., Ah Tow, L. E., Nicol, M. P., & Kaba, M. (2016). The human microbiome research in Africa – A systematic review. International Journal of Infectious Diseases, 45, 143-143. doi:10.1016/j.ijid.2016.02.349

Nichols, R. G., Peters, J. M., & Patterson, A. D. (2019). Interplay Between the Host, the Human Microbiome, and Drug Metabolism. Hum Genomics, 13(1), 27. doi:10.1186/s40246-019-0211-9

Nicolatou-Galitis, O. (2018). Abstracts of the MASCC/ISOO Annual Meeting 2018. Support Care Cancer, 26(2), 39-364. doi:10.1007/s00520-018-4193-2

Nicolatou-Galitis, O., Bafaloukos, D., Razis, E., Papadopoulou, E., Linardou, E., Grossi, I., . . . Migliorati, C. A. (2012). Abstracts of the 2012 International MASCC/ISOO (Multiple Association of Supportive Care in Cancer/International Society for Oral Oncology) Symposium. New York City, New York, USA. June 28-30, 2012. Support Care Cancer, 20 Suppl 1, 1-283. doi:10.1007/s00520-012-1479-7

Nicolatou-Galitis, O., Migkou, M., Psyrri, A., Bamias, A., Pectasides, D., Economopoulos, T., . . . Dimopoulos, M. A. (2012). Gingival bleeding and jaw bone necrosis in patients with metastatic renal cell carcinoma receiving sunitinib: report of 2 cases with clinical implications. Oral Surg Oral Med Oral Pathol Oral Radiol, 113(2), 234-238. doi:10.1016/j.tripleo.2011.08.024

Nicolatou-Galitis, O., Papadopoulou, E., Vardas, E., Kouri, M., Galiti, D., Galitis, E., . . . Ripamonti, C. I. (2020). Alveolar bone histological necrosis observed prior to extractions in patients, who received bone-targeting agents. Oral Dis, 26(5), 955-966. doi:10.1111/odi.13294

Nicolatou-Galitis, O., Psyrri, A., Pectasides, D., & Economopoulos, T. (2010). Abstracts of the 2010 International MASCC/ISOO (Multiple Association of Supportive Care in Cancer/International Society for Oral Oncology) Symposium. June 24026, 2010. Vancouver, Canada. Support Care Cancer, 18 Suppl 3, S77-220. doi:10.1007/s00520-010-0891-0

Niemiec, B. A. (2008). Periodontal disease. Top Companion Anim Med, 23(2), 72-80. doi:10.1053/j.tcam.2008.02.003

Nifosi, G., Bressand, H., Nifosi, A. F., Nifosi, L., & Damseaux, P. (2017). Epulis-Like Presentation of Gingival Renal Cancer Metastasis. Case Rep Oncol, 10(2), 758-763. doi:10.1159/000479500

Niklander, S., Bordagaray, M. J., Fernandez, A., & Hernandez, M. (2021). Vascular Endothelial Growth Factor: A Translational View in Oral Non-Communicable Diseases. Biomolecules, 11(1), 1-27. doi:10.3390/biom11010085

Nishide, N., & Kanamura, N. (2006). The value of carcinoembryonic antigen staining to determine the primary malignancy in metastatic carcinoma to the gingiva. Am J Clin Oncol, 29(3), 316-317. doi:10.1097/01.coc.0000164005.25685.13

Nishikawa, M., Honda, M., Kimura, R., Kobayashi, A., Yamaguchi, Y., Kobayashi, H., . . . Seto, K. (2019). Clinical impact of periodontal disease on postoperative complications in gastrointestinal cancer patients. Int J Clin Oncol, 24(12), 1558-1564. doi:10.1007/s10147-019-01513-y

Nisi, M., La Ferla, F., Karapetsa, D., Gennai, S., Ramaglia, L., Graziani, F., & Gabriele, M. (2016). Conservative surgical management of patients with bisphosphonate-related osteonecrosis of the jaws: a series of 120 patients. Br J Oral Maxillofac Surg, 54(8), 930-935. doi:10.1016/j.bjoms.2016.06.015

Noreen, M., Shah, M. A., Mall, S. M., Choudhary, S., Hussain, T., Ahmed, I., . . . Raza, M. I. (2012). TLR4 polymorphisms and disease susceptibility. Inflamm Res, 61(3), 177-188. doi:10.1007/s00011-011-0427-1

Noren Hooten, N., Yanez-Mo, M., DeRita, R., Russell, A., Quesenberry, P., Ramratnam, B., . . . Languino, L. R. (2020). Hitting the Bullseye: Are extracellular vesicles on target? J Extracell Vesicles, 10(1), e12032. doi:10.1002/jev2.12032

Nowak, M., Krämer, B., Haupt, M., Papapanou, P. N., Kebschull, J., Hoffmann, P., . . . Kebschull, M. (2012). Poster Sessions. Immunology, 137, 185-772. doi:10.1111/imm.12002

Nowak, M., Kramer, B., Haupt, M., Papapanou, P. N., Kebschull, J., Hoffmann, P., . . . Kebschull, M. (2013). Activation of invariant NK T cells in periodontitis lesions. J Immunol, 190(5), 2282-2291. doi:10.4049/jimmunol.1201215

O'Neill, D. G., Romans, C., Brodbelt, D. C., Church, D. B., Cerna, P., & Gunn-Moore, D. A. (2019). Persian cats under first opinion veterinary care in the UK: demography, mortality and disorders. Sci Rep, 9(1), 12952. doi:10.1038/s41598-019-49317-4

O'Ryan, F. S., Khoury, S., Liao, W., Han, M. M., Hui, R. L., Baer, D., . . . Lo, J. C. (2009). Intravenous bisphosphonate-related osteonecrosis of the jaw: bone scintigraphy as an early indicator. J Oral Maxillofac Surg, 67(7), 1363-1372. doi:10.1016/j.joms.2009.03.005

Oelke, M., Wagg, A., Takita, Y., Buttner, H., & Viktrup, L. (2017). Efficacy and safety of tadalafil 5 mg once daily in the treatment of lower urinary tract symptoms associated with benign prostatic hyperplasia in men aged >/=75 years: integrated analyses of pooled data from multinational, randomized, placebo-controlled clinical studies. BJU Int, 119(5), 793-803. doi:10.1111/bju.13744

Ogata, Y., Matsui, S., Kato, A., Zhou, L., Nakayama, Y., & Takai, H. (2014). MicroRNA expression in inflamed and noninflamed gingival tissues from Japanese patients. J Oral Sci, 56(4), 253-260. doi:10.2334/josnusd.56.253

Oguz, Y., Vural, A., Bulucu, F., Yenicesu, M., Caglar, K., & Oktenli, C. (2000). Noninfectious complications in renal transplant patients. Gulhane Medical Journal, 42(3), 263-268.

Oh, S. S., Chang, S. C., Cai, L., Cordon-Cardo, C., Ding, B. G., Greenland, S., . . . Zhang, Z. F. (2010). Single nucleotide polymorphisms of 8 inflammation-related genes and their associations with smoking-related cancers. Int J Cancer, 127(9), 2169-2182. doi:10.1002/ijc.25214

Ohadian Moghadam, S., & Momeni, S. A. (2021). Human microbiome and prostate cancer development: current insights into the prevention and treatment. Front Med, 15(1), 11-32. doi:10.1007/s11684-019-0731-7

Ohashi, Y., Yamamoto, Y., Yamada, A., Yamamoto, K., Arai, K., Namba, F., . . . Oka, M. (2021). Aortic Rupture due to Co-localization of Aortic Intimal Myofibroblastic Sarcoma and Urothelial Carcinoma: A Unique Case Report. Intern Med, 60(2), 269-274. doi:10.2169/internalmedicine.5191-20

Ojeda, I., Moreno-Guzman, M., Gonzalez-Cortes, A., Yanez-Sedeno, P., & Pingarron, J. M. (2014). Electrochemical magnetoimmunosensor for the ultrasensitive determination of interleukin-6 in saliva and urine using poly-HRP streptavidin conjugates as labels for signal amplification. Anal Bioanal Chem, 406(25), 6363-6371. doi:10.1007/s00216-014-8055-6

Olczak-Kowalczyk, D., Daszkiewicz, M., Krasuska, S., Dembowska-Baginska, B., Gozdowski, D., Daszkiewicz, P., . . . Semczuk, K. (2012). Bacteria and Candida yeasts in inflammations of the oral mucosa in children with secondary immunodeficiency. J Oral Pathol Med, 41(7), 568-576. doi:10.1111/j.1600-0714.2012.01146.x

Olsen, I. (2015). From the Acta Prize Lecture 2014: the periodontal-systemic connection seen from a microbiological standpoint. Acta Odontol Scand, 73(8), 563-568. doi:10.3109/00016357.2015.1007480

Olszewska, M., Kolacinska-Strasz, Z., Sulej, J., Labecka, H., Cwikla, J., Natorska, U., & Blaszczyk, M. (2007). Efficacy and safety of cyclophosphamide, azathioprine, and cyclosporine (ciclosporin) as adjuvant drugs in pemphigus vulgaris. Am J Clin Dermatol, 8(2), 85-92. doi:10.2165/00128071-200708020-00004

Ommer, J., Selfe, J. L., Wachtel, M., O'Brien, E. M., Laubscher, D., Roemmele, M., . . . Schafer, B. W. (2020). Aurora A Kinase Inhibition Destabilizes PAX3-FOXO1 and MYCN and Synergizes with Navitoclax to Induce Rhabdomyosarcoma Cell Death. Cancer Res, 80(4), 832-842. doi:10.1158/0008-5472.CAN-19-1479

Osaku, M., Ueda, M., Miyakawa, T., Toyoda, H., Uesato, K., Yamada, Y., . . . Kitajima, M. (2001). Correlation between EGF receptor expression and peplomycin cytotoxicity in squamous cell carcinoma cell lines. Oncol Rep, 8(4), 855-860. doi:10.3892/or.8.4.855

Osiak, M., Szubinska-Lelonkiewicz, D., Wychowanski, P., Karakulska-Prystupiuk, E., Jedrzejczak, W., Wojtowicz, A., & Fiedor, P. (2018). Frequency of Pathologic Changes in the Oral Cavity in Patients Subjected to Long-term Pharmacologic Immunosuppressive Therapy After Kidney, Liver, and Hematopoietic Cell Transplantation. Transplant Proc, 50(7), 2176-2178. doi:10.1016/j.transproceed.2018.04.003

Osiak, M., Wychowanski, P., Grzeszczyk, M., Durlik, M., & Fiedor, P. (2020). Differences in the Incidence of Pathologic Lesions on the Oral Mucosa in Patients Undergoing Hemodialysis vs Renal Organ Transplant Recipients Subjected to Long-term Pharmacologic Immunosuppressive Therapy. Transplant Proc, 52(8), 2409-2411. doi:10.1016/j.transproceed.2020.02.105

Ostronoff, M., Ostronoff, F., Calixto, R., Florencio, R., Florencio, M., Domingues, M. C., . . . Tagliari, C. (2007). Life-threatening hemolytic-uremic syndrome treated with rituximab in an allogeneic bone marrow transplant recipient. Bone Marrow Transplant, 39(10), 649-651. doi:10.1038/sj.bmt.1705657

Othman, N. H. (2012). Honey and cancer: sustainable inverse relationship particularly for developing nations-a review. Evid Based Complement Alternat Med, 2012, 410406. doi:10.1155/2012/410406

Ottria, L., Candotto, V., Cura, F., Baggi, L., Arcuri, C., Nardone, M., . . . Carinci, F. (2018). Human Papilloma Virus associated with oral cancer and preventive strategies: the role of vaccines. J Biol Regul Homeost Agents, 32(2 Suppl. 1), 61-65.

Ouchi, K., Miyachi, M., Yagyu, S., Kikuchi, K., Kuwahara, Y., Tsuchiya, K., . . . Hosoi, H. (2020). Oncogenic role of HMGA2 in fusion-negative rhabdomyosarcoma cells. Cancer Cell Int, 20(1), 192. doi:10.1186/s12935-020-01282-z

Oudard, S., Geoffrois, L., Guillot, A., Chevreau, C., Deville, J. L., Falkowski, S., . . . Gross-Goupil, M. (2016). Clinical activity of sunitinib rechallenge in metastatic renal cell carcinoma-Results of the REchallenge with SUnitinib in MEtastatic RCC (RESUME) Study. Eur J Cancer, 62, 28-35. doi:10.1016/j.ejca.2016.04.003

Owen, R., & Reilly, G. C. (2018). In vitro Models of Bone Remodelling and Associated Disorders. Front Bioeng Biotechnol, 6, 134. doi:10.3389/fbioe.2018.00134

Owens, M. D., Beckles, G. L., Ho, K. K., Gorrell, P., Brady, J., & Kaftarian, J. S. (2008). Women with diagnosed diabetes across the life stages: underuse of recommended preventive care services. J Womens Health (Larchmt), 17(9), 1415-1423. doi:10.1089/jwh.2008.1125

Owosho, A. A., Xu, B., Kadempour, A., Yom, S. K., Randazzo, J., Ghossein, R. A., . . . Estilo, C. L. (2016). Metastatic solid tumors to the jaw and oral soft tissue: A retrospective clinical analysis of 44 patients from a single institution. J Craniomaxillofac Surg, 44(8), 1047-1053. doi:10.1016/j.jcms.2016.05.013

Ozet, A., Guran, S., & Beksac, M. (2008). Familial multiple myeloma associated with disorders of chronic inflammation: first report from Turkey. Clin Lymphoma Myeloma, 8(4), 246-248. doi:10.3816/CLM.2008.n.033

Pai, A., Prasad, S., & Dyasanoor, S. (2012). Acute leukemias: a dentist's perspective. Minerva Stomatol, 61(5), 233-238.

Pai, S. S., Tilton, R. D., & Przybycien, T. M. (2009). Poly(ethylene glycol)-modified proteins: implications for poly(lactide-co-glycolide)-based microsphere delivery. AAPS J, 11(1), 88-98. doi:10.1208/s12248-009-9081-8

Palacios, C., Joshipura, K., & Willett, W. (2009). Nutrition and health: guidelines for dental practitioners. Oral Dis, 15(6), 369-381. doi:10.1111/j.1601-0825.2009.01571.x

Pandhi, R. K., Beci, T. R., & Dhawar, I. K. (1975). Leiomyosarcoma of the labium majus with extensive metastases. Dermatologica, 150(2), 70-74. doi:10.1159/000251405

Pang, D., Gao, Y., Liao, L., & Ying, X. (2020). Brain functional network alterations caused by a strong desire to void in healthy adults: a graph theory analysis study. Neurourol Urodyn, 39(7), 1966-1976. doi:10.1002/nau.24445

Panopalis, P., & Clarke, A. E. (2006). Systemic lupus erythematosus: clinical manifestations, treatment and economics. Expert Rev Pharmacoecon Outcomes Res, 6(5), 563-575. doi:10.1586/14737167.6.5.563

Papadopoulou, E., Nicolatou-Galitis, O., Kouri, M., Vardas, E., Demiri, M., Tryfonopoulos, D., . . . Ardavanis, A. (2018). Abstracts of the MASCC/ISOO Annual Meeting 2018. Support Care Cancer, 26(2), 39-364. doi:10.1007/s00520-018-4193-2

Paraskevas, K. I., Veith, F. J., & Mikhailidis, D. P. (2019). The Association Between Abdominal Aortic Aneurysms With Cardiovascular and Noncardiovascular Diseases. Angiology, 70(1), 8-11. doi:10.1177/0003319718785790

Parida, S., & Sharma, D. (2019). The power of small changes: Comprehensive analyses of microbial dysbiosis in breast cancer. Biochim Biophys Acta Rev Cancer, 1871(2), 392-405. doi:10.1016/j.bbcan.2019.04.001

Park, E. J., Amatya, S., Kim, M. S., Park, J. H., Seol, E., Lee, H., . . . Na, D. H. (2013). Long-acting injectable formulations of antipsychotic drugs for the treatment of schizophrenia. Arch Pharm Res, 36(6), 651-659. doi:10.1007/s12272-013-0105-7

Park, I., & Kang, S. (2017). Distal appendicular skeletal involvement of diffuse large B-cell lymphoma on technetium-99m methylenediphosphonate bone scintigraphy and (18)F-fluorodeoxyglucose positron emission tomography/computed tomography: a case report. J Med Case Rep, 11(1), 89. doi:10.1186/s13256-017-1246-y

Pascoal, A., Estevinho, M. M., Choupina, A. B., Sousa-Pimenta, M., & Estevinho, L. M. (2019). An overview of the bioactive compounds, therapeutic properties and toxic effects of apitoxin. Food Chem Toxicol, 134, 110864. doi:10.1016/j.fct.2019.110864

Pastremoli, A. (1991). [Gingival metastasis, the first clinical sign of a silent kidney carcinoma. A case report]. Minerva Stomatol, 40(12), 825-828.

Patel, G. C., & Dalwadi, C. A. (2013). Recent patents on stimuli responsive hydrogel drug delivery system. Recent Pat Drug Deliv Formul, 7(3), 206-215. doi:10.2174/1872211307666131118141600

Patel, P., & Sheth, N. R. (2013). Biodegradable polymers -Excipients for injectable drugs. Pharma Times, 45(3), 85-87.

Pathak, S., Sonalika, W. G., Hs, V., & Tegginammani, A. S. (2017). Premolar Cystic Ameloblastoma in a Child. J Coll Physicians Surg Pak, 27(1), 47-48. doi:2521

Patryk, K., Maciej, K., Malgorzata, K., & Wieslaw, K. (2020). Egg White Cystatin – A Review. Acta Veterinaria, 70(4), 401-422. doi:10.2478/acve-2020-0031

Pavone, P., Pratico, A. D., Falsaperla, R., Ruggieri, M., Zollino, M., Corsello, G., & Neri, G. (2015). Congenital generalized hypertrichosis: the skin as a clue to complex malformation syndromes. Ital J Pediatr, 41(1), 55. doi:10.1186/s13052-015-0161-3

Peng, L., Bu, Z., Zhou, Y., Ye, X., Liu, J., & Zhao, Q. (2014). Hemorrhagic events in cancer patients treated with aflibercept: a meta-analysis. Tumour Biol, 35(9), 9419-9427. doi:10.1007/s13277-014-2189-1

Penson, R. T., Moore, K. M., Fleming, G. F., Braly, P., Schimp, V., Nguyen, H., . . . McGuire, W. P. (2014). A phase II study of ramucirumab (IMC-1121B) in the treatment of persistent or recurrent epithelial ovarian, fallopian tube or primary peritoneal carcinoma. Gynecol Oncol, 134(3), 478-485. doi:10.1016/j.ygyno.2014.06.029

Pereira-Lopes, O., Sampaio-Maia, B., Sampaio, S., Vieira-Marques, P., Monteiro-da-Silva, F., Braga, A. C., . . . Pestana, M. (2013). Periodontal inflammation in renal transplant recipients receiving everolimus or tacrolimus - preliminary results. Oral Dis, 19(7), 666-672. doi:10.1111/odi.12051

Perez, O. A., & Patton, T. (2009). Novel therapies for pemphigus vulgaris: an overview. Drugs Aging, 26(10), 833-846. doi:10.2165/11316810-000000000-00000

Perricone, C., & Perricone, R. (2014). 'Autoimmunity cutting edge at the 21st century. Representation of the 9th International Congress of Autoimmunity, Nice, France 2014'. Autoimmun Rev, 13(11), 1079-1081. doi:10.1016/j.autrev.2014.10.015

Perry, P. J., Yates, W. R., Williams, R. D., Andersen, A. E., MacIndoe, J. H., Lund, B. C., & Holman, T. L. (2002). Testosterone therapy in late-life major depression in males. J Clin Psychiatry, 63(12), 1096-1101. doi:10.4088/jcp.v63n1202

Pertusa Pena, C., Llarena Ibarguren, R., Zabala Egurrola, J. A., Lopez Cedrun, J., & Martinez Conde, R. (1989). [Gingival metastasis: a rare presenting form of renal adenocarcinoma]. Arch Esp Urol, 42(4), 365-366.

Peterlik, M., & Cross, H. S. (2009). Vitamin D and calcium insufficiency-related chronic diseases: molecular and cellular pathophysiology. Eur J Clin Nutr, 63(12), 1377-1386. doi:10.1038/ejcn.2009.105

Peters, C. P., Eshuis, E. J., Toxopeus, F. M., Hellemons, M. E., Jansen, J. M., D'Haens, G. R., . . . North Holland, G. U. T. c. (2014). Adalimumab for Crohn's disease: long-term sustained benefit in a population-based cohort of 438 patients. J Crohns Colitis, 8(8), 866-875. doi:10.1016/j.crohns.2014.01.012

Petrylak, D. P., de Wit, R., Chi, K. N., Drakaki, A., Sternberg, C. N., Nishiyama, H., . . . Lowe, T. E. (2020). Ramucirumab plus docetaxel versus placebo plus docetaxel in patients with locally advanced or metastatic urothelial carcinoma after platinum-based therapy (RANGE): overall survival and updated results of a randomised, double-blind, phase 3 trial. The Lancet Oncology, 21(1), 105-120. doi:10.1016/s1470-2045(19)30668-0

Petty, W. J., Miller, A. A., McCoy, T. P., Gallagher, P. E., Tallant, E. A., & Torti, F. M. (2009). Phase I and pharmacokinetic study of angiotensin-(1-7), an endogenous antiangiogenic hormone. Clin Cancer Res, 15(23), 7398-7404. doi:10.1158/1078-0432.CCR-09-1957

Phal, P. M., Myall, R. W., Assael, L. A., & Weissman, J. L. (2007). Imaging findings of bisphosphonate-associated osteonecrosis of the jaws. AJNR Am J Neuroradiol, 28(6), 1139-1145. doi:10.3174/ajnr.A0518

Philibert, D., & Cattran, D. (2008). Remission of proteinuria in primary glomerulonephritis: we know the goal but do we know the price? Nat Clin Pract Nephrol, 4(10), 550-559. doi:10.1038/ncpneph0915

Piattelli, A., Fioroni, M., & Rubini, C. (1999). Gingival metastasis from a prostate adenocarcinoma: report of a case. J Periodontol, 70(4), 441-444. doi:10.1902/jop.1999.70.4.441

Pizzo, G., Guiglia, R., Lo Russo, L., & Campisi, G. (2010). Dentistry and internal medicine: from the focal infection theory to the periodontal medicine concept. Eur J Intern Med, 21(6), 496-502. doi:10.1016/j.ejim.2010.07.011

Pockley, A. G., Henderson, B., & Multhoff, G. (2014). Extracellular cell stress proteins as biomarkers of human disease. Biochem Soc Trans, 42(6), 1744-1751. doi:10.1042/BST20140205

Polascik, T. J. (2008). Bone health in prostate cancer patients receiving androgen-deprivation therapy: the role of bisphosphonates. Prostate Cancer Prostatic Dis, 11(1), 13-19. doi:10.1038/sj.pcan.4501019

Ponseti, J. M., Gamez, J., Azem, J., López-Cano, M., Vilallonga, R., & Armengol, M. (2008). Tacrolimus for myasthenia gravis: A clinical study of 212 patients. In (Vol. 1132, pp. 254-263).

Popat, R. (2010). Organ transplantation: Immunosuppression. Clinical Pharmacist, 2(2), 48-52.

Porter, C. M., Shrestha, E., Peiffer, L. B., & Sfanos, K. S. (2018). The microbiome in prostate inflammation and prostate cancer. Prostate Cancer Prostatic Dis, 21(3), 345-354. doi:10.1038/s41391-018-0041-1

Powell, N., Canavan, J. B., MacDonald, T. T., & Lord, G. M. (2010). Transcriptional regulation of the mucosal immune system mediated by T-bet. Mucosal Immunol, 3(6), 567-577. doi:10.1038/mi.2010.53

Powell, R., Scott, N. W., Manyande, A., Bruce, J., Vogele, C., Byrne-Davis, L. M., . . . Johnston, M. (2016). Psychological preparation and postoperative outcomes for adults undergoing surgery under general anaesthesia. Cochrane Database Syst Rev(5), CD008646. doi:10.1002/14651858.CD008646.pub2

Prabakaran, A., Prabhu, M., Kar, R., & Basu, D. (2019). 60th Annual Conference of Indian Society of Hematology & Blood Transfusion (ISHBT) October 2019. Indian Journal of Hematology and Blood Transfusion, 35(S1), 1-151. doi:10.1007/s12288-019-01207-5

Prasad, V., Huang, K. P., Prasad, S., Miller, K., & Brenner, W. (2017). Lu-177 DKFZ-617 PSMA for treatment of patients with progressive prostate cancer: Toxicity, efficacy and survival. NuklearMedizin, 56(2), A38.

Quattrocchi, E., Ostergaard, M., Taylor, P. C., van Vollenhoven, R. F., Chu, M., Mallett, S., . . . Kurrasch, R. (2016). Safety of Repeated Open-Label Treatment Courses of Intravenous Ofatumumab, a Human Anti-CD20 Monoclonal Antibody, in Rheumatoid Arthritis: Results from Three Clinical Trials. PLoS One, 11(6), e0157961. doi:10.1371/journal.pone.0157961

Quellmann, S., Schwarzer, G., Hubel, K., Greb, A., Engert, A., & Bohlius, J. (2008). Corticosteroids for preventing graft-versus-host disease after allogeneic myeloablative stem cell transplantation. Cochrane Database Syst Rev(3), CD004885. doi:10.1002/14651858.CD004885.pub2

Rajendran, M., Looney, S., Singh, N., Elashiry, M., Meghil, M. M., El-Awady, A. R., . . . Cutler, C. W. (2019). Systemic Antibiotic Therapy Reduces Circulating Inflammatory Dendritic Cells and Treg-Th17 Plasticity in Periodontitis. J Immunol, 202(9), 2690-2699. doi:10.4049/jimmunol.1900046

Rajesh, E., Mangai, T. A., Babu, N. A., & Malathi, L. (2020). Clear cell tumors of the oral cavity. European Journal of Molecular and Clinical Medicine, 7(5), 1429-1435.

Ravaud, A., Gomez-Roca, C., Picat, M. Q., Digue, L., Chevreau, C., Gimbert, A., . . . Delord, J. P. (2017). Phase I study of axitinib and everolimus in metastatic solid tumours and extension to metastatic renal cell carcinoma: Results of EVAX study. Eur J Cancer, 85, 39-48. doi:10.1016/j.ejca.2017.07.031

Rawat, J., Singh, S., & Chaubey, D. (2017). Spontaneous bladder rupture: unusual presentation in a haemophilic child. BMJ Case Rep, 2017. doi:10.1136/bcr-2017-220943

Rawls, W. E., Tompkins, W. A., Figueroa, M. E., & Melnick, J. L. (1968). Herpesvirus type 2: association with carcinoma of the cervix. Science, 161(3847), 1255-1256. doi:10.1126/science.161.3847.1255

Razis, E., Karina, M., Karanastassi, S., & Fountzilas, G. (2006). Three case reports of hand-foot syndrome with gefitinib. Cancer Invest, 24(5), 514-516. doi:10.1080/07357900600814847

Re, G., Barbero, R., & Cuniberti, B. (2009). The vanilloid receptor TRPV-1: a potential target in the management of pain and inflammation in domestic animals. Journal of Veterinary Pharmacology and Therapeutics, 32, 36-38.

Reap, L., McDonald, K., Balakrishnan, A., & Vakhariya, C. (2020). Kasabach-Merritt-like phenomenon in a massive uterine leiomyoma presenting with chronic disseminated intravascular coagulation: A case report. Case Rep Womens Health, 28, e00262. doi:10.1016/j.crwh.2020.e00262

Reddy, M. S. (2007). Reaching a better understanding of non-oral disease and the implication of periodontal infections. Periodontol 2000, 44, 9-14. doi:10.1111/j.1600-0757.2007.00213.x

Reece, A. S. (2009). Chronic toxicology of cannabis. Clin Toxicol (Phila), 47(6), 517-524. doi:10.1080/15563650903074507

Reece, A. S. (2010). Chronic immune stimulation as a contributing cause of chronic disease in opiate addiction including multi-system ageing. Med Hypotheses, 75(6), 613-619. doi:10.1016/j.mehy.2010.07.047

Reich, W., Bilkenroth, U., Schubert, J., Wickenhauser, C., & Eckert, A. W. (2015). Surgical treatment of bisphosphonate-associated osteonecrosis: Prognostic score and long-term results. J Craniomaxillofac Surg, 43(9), 1809-1822. doi:10.1016/j.jcms.2015.07.035

Reid, P., & Holen, I. (2009). Pathophysiological roles of osteoprotegerin (OPG). Eur J Cell Biol, 88(1), 1-17. doi:10.1016/j.ejcb.2008.06.004

Reigstad, L. J., Varhaug, J. E., & Lillehaug, J. R. (2005). Structural and functional specificities of PDGF-C and PDGF-D, the novel members of the platelet-derived growth factors family. FEBS J, 272(22), 5723-5741. doi:10.1111/j.1742-4658.2005.04989.x

Ren, Q., Yan, X., Zhou, Y., & Li, W. X. (2016). Periodontal therapy as adjunctive treatment for gastric Helicobacter pylori infection. Cochrane Database Syst Rev, 2(2), CD009477. doi:10.1002/14651858.CD009477.pub2

Rezazadeh, M., Gharesouran, J., Moradi, M., Noroozi, R., Omrani, M. D., Taheri, M., & Ghafouri-Fard, S. (2018). Association Study of ANRIL Genetic Variants and Multiple Sclerosis. J Mol Neurosci, 65(1), 54-59. doi:10.1007/s12031-018-1069-3

Rial, N. S., Choi, K., Nguyen, T., Snyder, B., & Slepian, M. J. (2012). Nuclear factor kappa B (NF-kappaB): a novel cause for diabetes, coronary artery disease and cancer initiation and promotion? Med Hypotheses, 78(1), 29-32. doi:10.1016/j.mehy.2011.09.034

Ribeiro, M. T., Rosa, M. A., Lima, R. M., Vargas, A. M., Haddad, J. P., & Ferreira, E. F. E. (2011). Edentulism and shortened dental arch in Brazilian elderly from the National Survey of Oral Health 2003. Rev Saude Publica, 45(5), 817-823. doi:10.1590/s0034-89102011005000057

Richards, C., Pantanowitz, L., & Dezube, B. J. (2011). Antimicrobial and non-antimicrobial tetracyclines in human cancer trials. Pharmacol Res, 63(2), 151-156. doi:10.1016/j.phrs.2010.10.008

Richters, J., Grulich, A., Ellard, J., Hendry, O., & Kippax, S. (2003). HIV transmission among gay men through oral sex and other uncommon routes: case series of HIV seroconverters, Sydney. Aids, 17(15), 2269-2271. doi:10.1097/00002030-200310170-00020

Riley, P., & Lamont, T. (2013). Triclosan/copolymer containing toothpastes for oral health. Cochrane Database Syst Rev(12), CD010514. doi:10.1002/14651858.CD010514.pub2

Risse, L., Negrier, P., Dang, P. M., Bedane, C., Bernard, P., Labrousse, F., & Bonnetblanc, J. M. (1995). Treatment of verrucous carcinoma with recombinant alfa-interferon. Dermatology, 190(2), 142-144. doi:10.1159/000246664

Ritchie, C. S. (2007). Obesity and periodontal disease. Periodontol 2000, 44, 154-163. doi:10.1111/j.1600-0757.2007.00207.x

Rittinger, O., Gottardi, E., & Wiesmayr, S. (2013). Abstract 24. Jahrestagung. Medizinische Genetik, 25(1), 62-190. doi:10.1007/s11825-013-0376-x

Rizvi, S. (2007). Multiple sclerosis: current and future treatment options. Endocr Metab Immune Disord Drug Targets, 7(4), 292-299. doi:10.2174/187153007782794380

Rizzardi, C., Schneider, M., Barresi, E., Brollo, A., & Melato, M. (2009). Metastasis of high grade renal cell carcinoma, clear cell type, in fibrous dysplasia with superimposed giant cell reparative granuloma. Pathologica, 101(6), 240-243.

Roa, H. S., & Mizrahi, S. J. (1972). [Choriocarcinoma of the testicle with metastasis to the gingiva (report of a case)]. Rev Guatem Estomatol, 2(3), 96-99.

Rodero, C. F., Fioramonti Calixto, G. M., Cristina Dos Santos, K., Sato, M. R., Aparecido Dos Santos Ramos, M., Miro, M. S., . . . Chorilli, M. (2018). Curcumin-Loaded Liquid Crystalline Systems for Controlled Drug Release and Improved Treatment of Vulvovaginal Candidiasis. Mol Pharm, 15(10), 4491-4504. doi:10.1021/acs.molpharmaceut.8b00507

Rogers, S. J., Williams, C. S., & Roman, G. C. (2004). Myelopathy in Sjogren's syndrome: role of nonsteroidal immunosuppressants. Drugs, 64(2), 123-132. doi:10.2165/00003495-200464020-00001

Rohart, C., Badelon, I., Fajnkuchen, F., Nghiem-Buffet, S., & Chaine, G. (2008). [Ophthalmologic disease in sarcoid-like granulomatosis and true sarcoidosis in immunodeficiency. Four case reports]. J Fr Ophtalmol, 31(7), 683-691. doi:10.1016/s0181-5512(08)74382-1

Rojas-Garcia, P., Alberu-Gomez, J., & Medina-Franco, H. (2010). Breast fibroadenomas associated with immunosuppressive drugs. Am Surg, 76(6), E48-49.

Rojas-Reyes, M. X., Granados Rugeles, C., & Charry-Anzola, L. P. (2014). Oxygen therapy for lower respiratory tract infections in children between 3 months and 15 years of age. Cochrane Database Syst Rev(12), CD005975. doi:10.1002/14651858.CD005975.pub3

Rojas, J. M., Oliva, J. L., & Santos, E. (2011). Mammalian son of sevenless Guanine nucleotide exchange factors: old concepts and new perspectives. Genes Cancer, 2(3), 298-305. doi:10.1177/1947601911408078

Rollason, V., Laverriere, A., MacDonald, L. C., Walsh, T., Tramer, M. R., & Vogt-Ferrier, N. B. (2016). Interventions for treating bisphosphonate-related osteonecrosis of the jaw (BRONJ). Cochrane Database Syst Rev, 2(2), CD008455. doi:10.1002/14651858.CD008455.pub2

Rosa, A. C., Mio, M., Andreadou, I., & Sumbayev, V. V. (2020). Editorial: The Challenge of New Therapeutic Approaches for Unmet Therapeutic Needs. Front Pharmacol, 11, 01341. doi:10.3389/fphar.2020.01341

Rose, R. (2005). "Na-no, na-no" bacteria? Dent Today, 24(10), 80, 82, 84.

Rosen, L. S., Gordon, M. S., Robert, F., & Matei, D. E. (2014). Endoglin for targeted cancer treatment. Curr Oncol Rep, 16(2), 365. doi:10.1007/s11912-013-0365-x

Rosen, L. S., Hurwitz, H. I., Wong, M. K., Goldman, J., Mendelson, D. S., Figg, W. D., . . . Gordon, M. S. (2012). A phase I first-in-human study of TRC105 (Anti-Endoglin Antibody) in patients with advanced cancer. Clin Cancer Res, 18(17), 4820-4829. doi:10.1158/1078-0432.CCR-12-0098

Ross, J. A., Miller, M. M., & Rojas Hernandez, C. M. (2017). Comparative effectiveness and safety of direct oral anticoagulants (DOACs) versus conventional anticoagulation for the treatment of cancer-related venous thromboembolism: A retrospective analysis. Thromb Res, 150, 86-89. doi:10.1016/j.thromres.2016.12.016

Roudebush, P., Allen, T. A., Dodd, C. E., & Novotny, B. J. (2004). Application of evidence-based medicine to veterinary clinical nutrition. J Am Vet Med Assoc, 224(11), 1765-1771. doi:10.2460/javma.2004.224.1766

Rozenblit, M., DeCarlo, K., Lin, D., & Nierodzik, M. L. (2017). Esophageal squamous cell carcinoma with metastases to the phalanx and gingiva. Journal of General Internal Medicine, 32(2), S493.

Ruffion, A., Manel, A., Valignat, C., Lopez, J. G., Perrin-Fayolle, O., & Perrin, P. (2000). Successful use of Samarium 153 for emergency treatment of disseminated intravascular coagulation due to metastatic hormone refractory prostate cancer. J Urol, 164(3 Pt 1), 782. doi:10.1097/00005392-200009010-00043

Ruggiero, F., Carbone, D., Mugavero, R., Cura, F., Baggi, L., Arcuri, C., . . . Carinci, F. (2018). Human papilloma virus in the tonsillar microbiota of an Afghan population group. J Biol Regul Homeost Agents, 32(2 Suppl. 1), 191-196.

Ruhoy, S. M., & Yates, A. (2016). Macrothrombocytopenia With Dohle Body-Like Granulocyte Inclusions: A Case Report of May-Hegglin Anomaly in a 33-Year-Old White Woman With an Update on the Molecular Findings of MYH9-Related Disease. Lab Med, 47(3), 246-250. doi:10.1093/labmed/lmw033

Ruospo, M., Palmer, S., Vecchio, M., Gargano, L., Petruzzi, M., De Benedictis, M., . . . Strippoli, G. (2012). Oral Disease in People with Chronic Kidney Disease: Meta-Analysis of Prevalence and Association with Clinical Outcomes. Nephrology Dialysis Transplantation, 27, 394-395.

Sabattini, S., Bassi, P., & Bettini, G. (2015). Histopathological findings and proliferative activity of canine sebaceous gland tumours with a predominant reserve cell population. J Comp Pathol, 152(2-3), 145-152. doi:10.1016/j.jcpa.2014.12.011

Sadarangani, S. P., Estes, L. L., & Steckelberg, J. M. (2015). Non-anti-infective effects of antimicrobials and their clinical applications: a review. Mayo Clin Proc, 90(1), 109-127. doi:10.1016/j.mayocp.2014.09.006

Sáenz, J. A. G., Tarruella, S. L., Paredes, B. G., Lajusticia, L. R., Villalobos, L., & Rubio, E. D. (2007). Osteonecrosis of the jaw as an adverse bisphosphonate event: Three cases of bone metastatic prostate cancer patients treated with zoledronic acid. Medicina Oral, Patologia Oral y Cirugia Bucal, 12(5), E351-E356.

Sahoo, A., Mandal, A. K., Dwivedi, K., & Kumar, V. (2020). A cross talk between the immunization and edible vaccine: Current challenges and future prospects. Life Sci, 261, 118343. doi:10.1016/j.lfs.2020.118343

Saimura, M., Mitsuyama, S., Anan, K., Koga, K., Ono, M., & Toyoshima, S. (2012). A rare case of rapidly progressing angiosarcoma of the breast with multiple metastases to the bone, liver, ovary, and gingiva. International Cancer Conference Journal, 1(3), 159-163. doi:10.1007/s13691-012-0032-3

Saito, N., Ariyoshi, W., Okinaga, T., Kamegawa, M., Matsukizono, M., Akebiyama, Y., . . . Nishihara, T. (2014). Inhibitory effects of ameloblastin on epithelial cell proliferation. Arch Oral Biol, 59(8), 835-840. doi:10.1016/j.archoralbio.2014.05.010

Sakagami, H., Amano, S., Kikuchi, H., Nakamura, Y., Kuroshita, R., Watanabe, S., . . . Oizumi, T. (2008). Antiviral, antibacterial and vitamin C-synergized radical-scavenging activity of Sasa senanensis Rehder extract. In Vivo, 22(4), 471-476.

Sakashita, H., Miyata, M., Miyamoto, H., & Kurumaya, H. (1996). A case of quadruple cancer, including triple cancers in the head and neck region. J Oral Maxillofac Surg, 54(4), 501-505. doi:10.1016/s0278-2391(96)90128-7

Sakashita, M., Sakashita, S., Sakata, A., Uesugi, N., Ishige, K., Hyodo, I., & Noguchi, M. (2017). An autopsy case of non-traumatic fat embolism syndrome. Pathol Int, 67(9), 477-482. doi:10.1111/pin.12556

Saltzstein, D., Sieber, P., Morris, T., & Gallo, J. (2005). Prevention and management of bicalutamide-induced gynecomastia and breast pain: randomized endocrinologic and clinical studies with tamoxifen and anastrozole. Prostate Cancer Prostatic Dis, 8(1), 75-83. doi:10.1038/sj.pcan.4500782

Samelis, G. F., Ekmektzoglou, K. A., Tsiakou, A., Giannakaki, S., Georgoulias, D., & Christophilopoulos, K. (2011). Survival benefit during zoledronic acid and docetaxel-based chemotherapy in metastatic hormone-refractory prostate cancer patients: an institutional report. J BUON, 16(4), 738-743.

Sandomenico, C., Costanzo, R., Carillio, G., Piccirillo, M. C., Montanino, A., Di Maio, M., . . . Morabito, A. (2012). Bevacizumab in non small cell lung cancer: development, current status and issues. Curr Med Chem, 19(7), 961-971. doi:10.2174/092986712799320673

Saremi, A., Nelson, R., Hanson, R., Tulloch-Reid, M., Shlossman, M., Genco, R., & Knowler, W. (2003). Effect of periodontal disease on mortality in type 2 diabetes. Diabetes, 52, A228-A228.

Sasaki, H., Ohara, N., Minamikawa, T., Umeda, M., Komori, T., Kojima, N., . . . Itoh, T. (2008). Gingival metastasis from ovarian mucinous cystadenocarcinoma as an initial manifestation (a rare case report). Kobe J Med Sci, 54(3), E174-182.

Satarug, S., Garrett, S. H., Sens, M. A., & Sens, D. A. (2010). Cadmium, environmental exposure, and health outcomes. Environ Health Perspect, 118(2), 182-190. doi:10.1289/ehp.0901234

Sato, K., Yoshimura, A., Kaneko, T., Ukai, T., Ozaki, Y., Nakamura, H., . . . Ogata, Y. (2012). A single nucleotide polymorphism in 3'-untranslated region contributes to the regulation of Toll-like receptor 4 translation. J Biol Chem, 287(30), 25163-25172. doi:10.1074/jbc.M111.338426

Schad, F., Axtner, J., Kroz, M., Matthes, H., & Steele, M. L. (2018). Safety of Combined Treatment With Monoclonal Antibodies and Viscum album L Preparations. Integr Cancer Ther, 17(1), 41-51. doi:10.1177/1534735416681641

Schaffer, M., Schaffer, P. M., & Bar-Sela, G. (2015). An update on Curcuma as a functional food in the control of cancer and inflammation. Curr Opin Clin Nutr Metab Care, 18(6), 605-611. doi:10.1097/MCO.0000000000000227

Schiodt, M., Vadhan-Raj, S., Chambers, M. S., Nicolatou-Galitis, O., Politis, C., Coropciuc, R., . . . Saunders, D. P. (2018). A multicenter case registry study on medication-related osteonecrosis of the jaw in patients with advanced cancer. Support Care Cancer, 26(6), 1905-1915. doi:10.1007/s00520-017-4003-2

Schloss, A. J. (2012). A systematic format for resolving ethical issues in clinical periodontics. J Am Coll Dent, 79(3), 42-47.

Schneider-Gold, C., Hartung, H. P., & Gold, R. (2006). Mycophenolate mofetil and tacrolimus: new therapeutic options in neuroimmunological diseases. Muscle Nerve, 34(3), 284-291. doi:10.1002/mus.20543

Scholer, N., Langer, C., & Kuchenbauer, F. (2011). Circulating microRNAs as biomarkers - True Blood? Genome Med, 3(11), 72. doi:10.1186/gm288

Scholl, I., Kopp, T., Bohle, B., & Jensen-Jarolim, E. (2006). Biodegradable PLGA particles for improved systemic and mucosal treatment of Type I allergy. Immunol Allergy Clin North Am, 26(2), 349-364, ix. doi:10.1016/j.iac.2006.02.007

Schoubben, A., Ricci, M., & Giovagnoli, S. (2019). Meeting the unmet: from traditional to cutting-edge techniques for poly lactide and poly lactide-co-glycolide microparticle manufacturing. Journal of Pharmaceutical Investigation, 49(4), 381-404. doi:10.1007/s40005-019-00446-y

Scolozzi, P., Marret, N., Bouzourene, H., Luthi, F., Bauer, J., Jaques, B., & Lombardi, T. (2006). Mixed testicular germ cell tumor presenting as metastatic pure choriocarcinoma involving the maxillary gingiva. J Oral Pathol Med, 35(9), 579-581. doi:10.1111/j.1600-0714.2006.00443.x

Scott, E. N., & Thomas, A. L. (2008). Pi-88. Drugs of the Future, 33(1), 21-26. doi:10.1358/dof.2008.033.01.1165464

Sebaratnam, D., & Murrell, D. F. (2014). Treatment of pemphigus vulgaris and pemphigus foliaceus. Expert Review of Dermatology, 4(5), 469-481. doi:10.1586/edm.09.45

Seftel, A. (2007). Testosterone replacement therapy for male hypogonadism: part III. Pharmacologic and clinical profiles, monitoring, safety issues, and potential future agents. Int J Impot Res, 19(1), 2-24. doi:10.1038/sj.ijir.3901366

Sellin, J. N., Gressot, L. V., Suki, D., St Clair, E. G., Chern, J., Rhines, L. D., . . . Tatsui, C. E. (2015). Prognostic Factors Influencing the Outcome of 64 Consecutive Patients Undergoing Surgery for Metastatic Melanoma of the Spine. Neurosurgery, 77(3), 386-393; discussion 393. doi:10.1227/NEU.0000000000000790

Selvi, F., Faquin, W. C., Michaelson, M. D., & August, M. (2016). Three Synchronous Atypical Metastases of Clear Cell Renal Carcinoma to the Maxillary Gingiva, Scalp and the Distal Phalanx of the Fifth Digit: A Case Report. J Oral Maxillofac Surg, 74(6), 1286 e1281-1289. doi:10.1016/j.joms.2016.01.054

Semeraro, F., Morescalchi, F., Parmeggiani, F., Arcidiacono, B., & Costagliola, C. (2011). Systemic adverse drug reactions secondary to anti-VEGF intravitreal injection in patients with neovascular age-related macular degeneration. Curr Vasc Pharmacol, 9(5), 629-646. doi:10.2174/157016111796642670

Seo, D. E., Kim, S., & Park, B. J. (2020). Signals of Adverse Drug Reactions of Paliperidone Compared to Other Atypical Antipsychotics Using the Korean Adverse Event Reporting System Database. Clin Drug Investig, 40(9), 873-881. doi:10.1007/s40261-020-00945-z

Seoane, J., Van der Waal, I., Van der Waal, R. I., Cameselle-Teijeiro, J., Anton, I., Tardio, A., . . . Diz, P. (2009). Metastatic tumours to the oral cavity: a survival study with a special focus on gingival metastases. J Clin Periodontol, 36(6), 488-492. doi:10.1111/j.1600-051X.2009.01407.x

Sessa, C., Tosi, D., Vigano, L., Albanell, J., Hess, D., Maur, M., . . . Gianni, L. (2010). Phase Ib study of weekly mammalian target of rapamycin inhibitor ridaforolimus (AP23573; MK-8669) with weekly paclitaxel. Ann Oncol, 21(6), 1315-1322. doi:10.1093/annonc/mdp504

Shafiee, M. N., Ismail, N. M., Shan, L. P., Kampan, N., Omar, M. H., & Dali, H. M. (2011). A case report: metastatic choriocarcinoma to the gum. Sex Reprod Healthc, 2(2), 91-92. doi:10.1016/j.srhc.2011.02.001

Shah, C. H., Pappot, H., Agerbaek, M., Holmsten, K., Jaderling, F., Yachnin, J., . . . Ullen, A. (2019). Safety and Activity of Sorafenib in Addition to Vinflunine in Post-Platinum Metastatic Urothelial Carcinoma (Vinsor): Phase I Trial. Oncologist, 24(6), 745-e213. doi:10.1634/theoncologist.2018-0795

Shah, H. N., Gharbia, S. E., & O'Toole, C. M. (1992). Assessment of the relative cytotoxicity of Porphyromonas gingivalis cells, products, and components on human epithelial cell lines. J Periodontol, 63(1), 44-51. doi:10.1902/jop.1992.63.1.44

Shahrokni, A., Rajebi, M. R., & Saif, M. W. (2009). Toxicity and efficacy of 5-fluorouracil and capecitabine in a patient with TYMS gene polymorphism: A challenge or a dilemma? Clin Colorectal Cancer, 8(4), 231-234. doi:10.3816/CCC.2009.n.039

Shan, B., Shen, W., & Wang, H. (2020). Anlotinib in patients with recurrent platinum-resistant or refractory ovarian carcinoma: A prospective, single-arm, single-center, phase II clinical study. Journal of Clinical Oncology, 38(15_suppl), 6061-6061. doi:10.1200/JCO.2020.38.15_suppl.6061

Shanmugam, T., & Banerjee, R. (2011). Nanostructured self assembled lipid materials for drug delivery and tissue engineering. Ther Deliv, 2(11), 1485-1516. doi:10.4155/tde.11.105

Shaqman, M., Ioannidou, E., Burleson, J., Hull, D., & Dongari-Bagtzoglou, A. (2010). Periodontitis and inflammatory markers in transplant recipients. J Periodontol, 81(5), 666-672. doi:10.1902/jop.2010.090570

Sharifi-Rad, J., Ezzat, S. M., El Bishbishy, M. H., Mnayer, D., Sharopov, F., Kilic, C. S., . . . Martins, N. (2020). Rosmarinus plants: Key farm concepts towards food applications. Phytother Res, 34(7), 1474-1518. doi:10.1002/ptr.6622

Sharma, M., Astekar, M., Soi, S., Manjunatha, B., & Shetty, D. (2015). Viral Carcinogenesis of Oral Region and Recent Trends in Treatment. Recent Patents on Biomarkers, 5(1), 25-34. doi:10.2174/2210309005666150505183429

Sharma, S., Sharma, A. K., Gill, S. S., Shrivastav, A., & Shrivastav, B. R. (2013). Oxidative stress, chronic diseases and antioxidant potential of some religious grasses of poaceae family: An overview. Pharmacophore, 4(5), 134-145.

Shen, L. J., & Wu, F. L. (2007). Nanomedicines in renal transplant rejection--focus on sirolimus. Int J Nanomedicine, 2(1), 25-32. doi:10.2147/nano.2007.2.1.25

Shibahara, T., Morikawa, T., Yago, K., Kishimoto, H., Imai, Y., & Kurita, K. (2018). National Survey on Bisphosphonate-Related Osteonecrosis of the Jaws in Japan. J Oral Maxillofac Surg, 76(10), 2105-2112. doi:10.1016/j.joms.2018.04.009

Shimazaki, Y., Akifusa, S., Takeshita, T., Shibata, Y., Doi, Y., Hata, J., . . . Yamashita, Y. (2011). Effectiveness of the salivary occult blood test as a screening method for periodontal status. J Periodontol, 82(4), 581-587. doi:10.1902/jop.2010.100304

Shimazaki, Y., Kushiyama, M., Murakami, M., & Yamashita, Y. (2013). Relationship between normal serum creatinine concentration and periodontal disease in Japanese middle-aged males. J Periodontol, 84(1), 94-99. doi:10.1902/jop.2012.110528

Shirataki, Y., Kawase, M., Sakagami, H., Nakashima, H., Tani, S., Tanaka, T., . . . Motohashi, N. (2005). Bioactivities of anastasia black (Russian sweet pepper). Anticancer Res, 25(3B), 1991-1999.

Shirley, D. K., Kaner, R. J., & Glesby, M. J. (2013). Effects of smoking on non-AIDS-related morbidity in HIV-infected patients. Clin Infect Dis, 57(2), 275-282. doi:10.1093/cid/cit207

Shuster, J. (2008). Fatty liver disease associated with leuprorelin acetate: Methotrexate used for psoriasis causes different dermatological problem - Mefloquine-induced eosinophilic pneumonia. Gingival bleeding due to venlafaxine: Depression with mycophenolate mofetil - Propofol-induced movement disorders. Erythema nodosum and erythema multiforme after local lidocaine spray. Hospital Pharmacy, 43(3), 172-175.

Siasos, G., Tsigkou, V., Kokkou, E., Oikonomou, E., Vavuranakis, M., Vlachopoulos, C., . . . Tousoulis, D. (2014). Smoking and atherosclerosis: mechanisms of disease and new therapeutic approaches. Curr Med Chem, 21(34), 3936-3948. doi:10.2174/092986732134141015161539

Sigusch, B. W. (2013). The role of vitamin C (ascorbic acid) in the prevention and therapy of oral diseases. Arch Oral Biol, 58(8), 905-906. doi:10.1016/j.archoralbio.2013.06.001

Sikka, S., Sikka, P., Kaur, G., & Shetty, D. C. (2013). A review of histopathological and immunohistochemical parameters in diagnosis of metastatic renal cell carcinoma with a case of gingival metastasis. J Cancer Res Ther, 9(1), 105-107. doi:10.4103/0973-1482.110395

Silva, A. P. d., Flores, M., Mazaro, R., Luz, F. d., Silva, M., & Fighera, R. A. (2019). Oral lesions and retroviruses in shelter cats. Pesquisa Veterinaria Brasileira, 39(7), 516-522. doi:10.1590/1678-5150-pvb-5892

Sim, I. W., Sanders, K. M., Seymour, J., & Ebeling, P. R. (2014). Declining incidence of antiresorptive drug-associated osteonecrosis of the jaw (ARONJ) in patients with cancer: The importance of oral hygiene. Endocrine Reviews, 35.

Simkova, D., Kharaishvili, G., Korinkova, G., Ozdian, T., Suchankova-Kleplova, T., Soukup, T., . . . Bouchal, J. (2016). The dual role of asporin in breast cancer progression. Oncotarget, 7(32), 52045-52060. doi:10.18632/oncotarget.10471

Simkova, D., Kharaishvili, G., Slabakova, E., Murray, P. G., & Bouchal, J. (2016). Glycoprotein asporin as a novel player in tumour microenvironment and cancer progression. Biomed Pap Med Fac Univ Palacky Olomouc Czech Repub, 160(4), 467-473. doi:10.5507/bp.2016.037

Singh, A., Kumar Sharma, P., & Malviya, R. (2012). Sustained Drug Delivery Using Mucoadhesive Microspheres: The Basic Concept, Preparation Methods and Recent Patents. Recent Patents on Nanomedicine, 2(1), 62-77. doi:10.2174/1877913111202010062

Singh, J. A., Hossain, A., Mudano, A. S., Tanjong Ghogomu, E., Suarez-Almazor, M. E., Buchbinder, R., . . . Wells, G. A. (2017). Biologics or tofacitinib for people with rheumatoid arthritis naive to methotrexate: a systematic review and network meta-analysis. Cochrane Database Syst Rev, 5(5), CD012657. doi:10.1002/14651858.CD012657

Singh, J. A., Hossain, A., Tanjong Ghogomu, E., Mudano, A. S., Maxwell, L. J., Buchbinder, R., . . . Wells, G. A. (2017). Biologics or tofacitinib for people with rheumatoid arthritis unsuccessfully treated with biologics: a systematic review and network meta-analysis. Cochrane Database Syst Rev, 3(3), CD012591. doi:10.1002/14651858.CD012591

Singh, S., & Watt, K. D. (2012). Long-term medical management of the liver transplant recipient: what the primary care physician needs to know. Mayo Clin Proc, 87(8), 779-790. doi:10.1016/j.mayocp.2012.02.021

Singh, T., & Newman, A. B. (2011). Inflammatory markers in population studies of aging. Ageing Res Rev, 10(3), 319-329. doi:10.1016/j.arr.2010.11.002

Singh, V., Gupta, P., Khatana, S., Bhagol, A., & Gupta, A. (2014). A nonhealing ulcer of mandibular alveolar ridge. Oral Surg Oral Med Oral Pathol Oral Radiol, 117(3), 272-276. doi:10.1016/j.oooo.2012.06.016

Singh, V. K., Means, M., Pham, D., & Kloecker, G. H. (2016). ITP or Not-ITP-a rare case of PTLD recurrence presenting as autoimmune thrombocytopenia. Blood, 128(22).

Singh, V. P., Sharma, J., Babu, S., Rizwanulla, & Singla, A. (2013). Role of probiotics in health and disease: a review. J Pak Med Assoc, 63(2), 253-257.

Sinha, V. R., & Trehan, A. (2005). Biodegradable microspheres for parenteral delivery. Crit Rev Ther Drug Carrier Syst, 22(6), 535-602. doi:10.1615/critrevtherdrugcarriersyst.v22.i6.20

Siozopoulou, V., & Vanhoenacker, F. M. (2020). World Health Organization Classification of Odontogenic Tumors and Imaging Approach of Jaw Lesions. Semin Musculoskelet Radiol, 24(5), 535-548. doi:10.1055/s-0040-1710357

Siracka, E., Durkovsky, J., Jancina, J., & Revesz, L. (1973). Acute reaction and late damage of the normal tissue in cancer patients given a dynamic dose-fractionation under breathing of oxygen. Neoplasma, 20(6), 643-653.

Sleiwah, A., Thomas, G., Crawford, I., & Stanek, A. (2017). Gastric volvulus: a potentially fatal cause of acute abdominal pain. BMJ Case Rep, 2017. doi:10.1136/bcr-2016-217708

Smith, A. L., Robin, T. P., & Ford, H. L. (2012). Molecular pathways: targeting the TGF-beta pathway for cancer therapy. Clin Cancer Res, 18(17), 4514-4521. doi:10.1158/1078-0432.CCR-11-3224

Soe, H. H. K., Abas, A. B., Than, N. N., Ni, H., Singh, J., Said, A., & Osunkwo, I. (2020). Vitamin D supplementation for sickle cell disease. Cochrane Database Syst Rev, 5(5), CD010858. doi:10.1002/14651858.CD010858.pub3

Sokolowska-Wojdylo, M., Florek, A., Baranska-Rybak, W., Sikorska, M., Starzynska, A., Drogoszewska, B., & Wlodarkiewicz, A. (2013). Natural killer/T-cell lymphoma, nasal type, masquerading as recalcitrant periodontitis in a patient with a diagnosis of Wegener's granulomatosis. Am J Med Sci, 345(2), 163-167. doi:10.1097/MAJ.0b013e318268bc65

Soldani, F. A., Lamont, T., Jones, K., Young, L., Walsh, T., Lala, R., & Clarkson, J. E. (2018). One-to-one oral hygiene advice provided in a dental setting for oral health. Cochrane Database Syst Rev, 10(10), CD007447. doi:10.1002/14651858.CD007447.pub2

Soliman, D. S., Al-Sabbagh, A., Ibrahim, F., El-Omri, H., Yassin, M. A., & Amer, A. (2019). Local Field Radiotherapy Induced Therapy Related Myeloid Neoplasms and Bone Marrow Suppression. Blood, 134(Supplement_1), 5113-5113. doi:10.1182/blood-2019-131683

Somma, F., Castagnola, R., Bollino, D., & Marigo, L. (2010). Oral inflammatory process and general health. Part 1: The focal infection and the oral inflammatory lesion. Eur Rev Med Pharmacol Sci, 14(12), 1085-1095.

Sonis, S., Treister, N., Chawla, S., Demetri, G., & Haluska, F. (2010). Preliminary characterization of oral lesions associated with inhibitors of mammalian target of rapamycin in cancer patients. Cancer, 116(1), 210-215. doi:10.1002/cncr.24696

Sonu, I., Blonski, W., Lin, M. V., & Lichtenstein, G. R. (2010). An approach to the management of refractory ulcerative colitis. Minerva Gastroenterol Dietol, 56(2), 213-231.

Soory, M. (2010). Oxidative stress induced mechanisms in the progression of periodontal diseases and cancer: a common approach to redox homeostasis? Cancers (Basel), 2(2), 670-692. doi:10.3390/cancers2020670

Spector, S. A. (2009). Vitamin D earns more than a passing grade. J Infect Dis, 200(7), 1015-1017. doi:10.1086/605723

Srikanth, S., & Chen, Z. (2016). Plant Protease Inhibitors in Therapeutics-Focus on Cancer Therapy. Front Pharmacol, 7(DEC), 470. doi:10.3389/fphar.2016.00470

Stein, S. H., Livada, R., & Tipton, D. A. (2014). Re-evaluating the role of vitamin D in the periodontium. J Periodontal Res, 49(5), 545-553. doi:10.1111/jre.12149

Sterenborg, H. J. C. M., & Robinson, D. J. (2010). Photodynamic therapy: Clinical applications. In (Vol. 16, pp. 56-58).

Stipp, D., Tully, S., Murphy, C., Kahn, J., Helyar, J., Sellers, P., . . . O'Keefe, B. (2002). The executive body. Middle are ain't what it used to be. Here's how to stay on top of your game. Fortune, 145(1), 44-55.

Stipp, D., Tully, S., Murphy, C., Kahn, J., Helyar, J., Sellers, P., . . . O'Keefe, B. (2002). The executive body. Middle are ain't what it used to be. Here's how to stay on top of your game. Fortune, 145(1), 44-55.

Stock, S. J., Oyston, C., & Norman, J. E. (2013). Management of a woman with a previous preterm birth. Obstetrics, Gynaecology & Reproductive Medicine, 23(2), 53-58. doi:10.1016/j.ogrm.2013.01.001

Stojanovic, M., Krasic, D., Trajkovic, M., & Petrovic, V. (2020). Rare renal cell carcinoma metastasis to mandibular gingiva: A case report and literature review. Niger J Clin Pract, 23(10), 1483-1486. doi:10.4103/njcp.njcp_55_19

Stone, R. L., Sood, A. K., & Coleman, R. L. (2010). Collateral damage: toxic effects of targeted antiangiogenic therapies in ovarian cancer. Lancet Oncol, 11(5), 465-475. doi:10.1016/S1470-2045(09)70362-6

Strohle, A., Zanker, K., & Hahn, A. (2010). Nutrition in oncology: the case of micronutrients (review). Oncol Rep, 24(4), 815-828. doi:10.3892/or.2010.815

Sugimoto, M., Wong, D. T., Hirayama, A., Soga, T., & Tomita, M. (2010). Capillary electrophoresis mass spectrometry-based saliva metabolomics identified oral, breast and pancreatic cancer-specific profiles. Metabolomics, 6(1), 78-95. doi:10.1007/s11306-009-0178-y

Sugiyama, T., Mizuno, M., Aoki, Y., Sakurai, M., Nishikawa, T., Ueda, E., . . . Takeshima, N. (2017). A single-arm study evaluating bevacizumab, cisplatin, and paclitaxel followed by single-agent bevacizumab in Japanese patients with advanced cervical cancer. Jpn J Clin Oncol, 47(1), 39-46. doi:10.1093/jjco/hyw143

Sukhumthammarat, W., Putthapiban, P., & Vutthikraivit, W. (2019). Paper Abstract. Journal of the American Geriatrics Society, 67(S1), S1-S384. doi:10.1111/jgs.15898

Sun, B., Wu, L., Wu, Y., Zhang, C., Qin, L., Hayashi, M., . . . Liu, T. (2020). Therapeutic Potential of Centella asiatica and Its Triterpenes: A Review. Front Pharmacol, 11, 568032. doi:10.3389/fphar.2020.568032

Sunakawa, Y., Furuse, J., Okusaka, T., Ikeda, M., Nagashima, F., Ueno, H., . . . Sasaki, Y. (2014). Regorafenib in Japanese patients with solid tumors: phase I study of safety, efficacy, and pharmacokinetics. Invest New Drugs, 32(1), 104-112. doi:10.1007/s10637-013-9953-8

Sunga, A. Y., Eberl, M. M., Oeffinger, K. C., Hudson, M. M., & Mahoney, M. C. (2005). Care of cancer survivors. Am Fam Physician, 71(4), 699-706.

Suryadevara, M., Schurman, S. J., Landas, S. K., Philip, A., Gerlach, C. B., Tavares, T., & Souid, A. K. (2008). Systemic calciphylaxis. Pediatr Blood Cancer, 51(4), 548-550. doi:10.1002/pbc.21631

Sutherland, A., Naessens, K., Plugge, E., Ware, L., Head, K., Burton, M. J., & Wee, B. (2018). Olanzapine for the prevention and treatment of cancer-related nausea and vomiting in adults. Cochrane Database Syst Rev, 9(9), CD012555. doi:10.1002/14651858.CD012555.pub2

Suzuki, K., Fujii, M., Ishii, Y., Mochizuki, F., Suzuki, K., Yamagata, M., . . . Sakabe, T. (1989). [Radio- and radio-chemosensitivity of human head and neck cancer cell line detected by human tumor clonogenic assay]. Nichidai Koko Kagaku, 15(2), 157-165.

Suzuki, Y., Hisada, K., Hiraki, T., & Ando, A. (1974). Clinical evaluation of tumor scanning with 57Co-bleomycin. Radiology, 113(1), 139-143. doi:10.1148/113.1.139

Swinkels, H., Pottie, K., Tugwell, P., Rashid, M., Narasiah, L., Canadian Collaboration for, I., & Refugee, H. (2011). Development of guidelines for recently arrived immigrants and refugees to Canada: Delphi consensus on selecting preventable and treatable conditions. CMAJ, 183(12), E928-932. doi:10.1503/cmaj.090290

Taguchi, Y. H., & Murakami, Y. (2013). Principal component analysis based feature extraction approach to identify circulating microRNA biomarkers. PLoS One, 8(6), e66714. doi:10.1371/journal.pone.0066714

Takasaki, S., Kawasaki, Y., Kikuchi, M., Tanaka, M., Suzuka, M., Noda, A., . . . Mano, N. (2018). Relationships between sunitinib plasma concentration and clinical outcomes in Japanese patients with metastatic renal cell carcinoma. Int J Clin Oncol, 23(5), 936-943. doi:10.1007/s10147-018-1302-7

Takayama, T., Nagata, M., Kai, F., Sugiyama, T., & Ozono, S. (2013). Axitinib controlled metastatic renal cell carcinoma for 5 years. Jpn J Clin Oncol, 43(7), 747-751. doi:10.1093/jjco/hyt067

Tamai, R., Sugiyama, A., & Kiyoura, Y. (2011). Alendronate regulates cytokine production induced by lipid A through nuclear factor-kappaB and Smad3 activation in human gingival fibroblasts. J Periodontal Res, 46(1), 13-20. doi:10.1111/j.1600-0765.2010.01302.x

Tan, A. R., Alexe, G., & Reiss, M. (2009). Transforming growth factor-beta signaling: emerging stem cell target in metastatic breast cancer? Breast Cancer Res Treat, 115(3), 453-495. doi:10.1007/s10549-008-0184-1

Tanaka, Y., Nagai, Y., Dohdoh, M., Oizumi, T., Ohki, A., Kuroishi, T., . . . Endo, Y. (2013). In vitro cytotoxicity of zoledronate (nitrogen-containing bisphosphonate: NBP) and/or etidronate (non-NBP) in tumour cells and periodontal cells. Arch Oral Biol, 58(6), 628-637. doi:10.1016/j.archoralbio.2012.11.010

Tanguturi, S. C., & Nagarakanti, S. (2018). Polycystic Ovary Syndrome and Periodontal disease: Underlying Links- A Review. Indian J Endocrinol Metab, 22(2), 267-273. doi:10.4103/ijem.IJEM_577_17

Tao, J. J., Cangemi, N. A., Makker, V., Cadoo, K. A., Liu, J. F., Rasco, D. W., . . . Hyman, D. M. (2019). First-in-Human Phase I Study of the Activin A Inhibitor, STM 434, in Patients with Granulosa Cell Ovarian Cancer and Other Advanced Solid Tumors. Clin Cancer Res, 25(18), 5458-5465. doi:10.1158/1078-0432.CCR-19-1065

Tao, J. J., Liu, J. F., Rasco, D. W., Navarro, W., Haqq, C. M., & Hyman, D. M. (2018). First in human study of activin-A inhibitor, STM434, in patients with granulosa cell ovarian cancer and other advanced solid tumors. Cancer Research, 78(13). doi:10.1158/1538-7445.AM2018-CT011

Techanukul, T., Sethuraman, G., Zlotogorski, A., Horev, L., Macarov, M., Trainer, A., . . . Lai-Cheong, J. E. (2011). Novel and recurrent FERMT1 gene mutations in Kindler syndrome. Acta Derm Venereol, 91(3), 267-270. doi:10.2340/00015555-1063

Temtem, M., & Santos, J. L. (2012). Sphere of influence. In (pp. 28-31).

Theodoraki, A., & Bouloux, P. M. (2009). Testosterone therapy in men. Menopause Int, 15(2), 87-92. doi:10.1258/mi.2009.009025

Theou-Anton, N., Faivre, S., Dreyer, C., & Raymond, E. (2009). Benefit-risk assessment of sunitinib in gastrointestinal stromal tumours and renal cancer. Drug Saf, 32(9), 717-734. doi:10.2165/00002018-200932090-00003

Thirumalai, A., Berkseth, K. E., & Amory, J. K. (2017). Treatment of Hypogonadism: Current and Future Therapies. F1000Res, 6, 68. doi:10.12688/f1000research.10102.1

Thiruppathy, M., Kishore Kumar, S., Amaladas, J., & Pavani, D. (2019). Tobacco: An overview. Drug Invention Today, 12(3), 532-536.

Thomas, L. V., Suzuki, K., & Zhao, J. (2015). Probiotics: a proactive approach to health. A symposium report. Br J Nutr, 114 Suppl 1, S1-15. doi:10.1017/S0007114515004043

Thorwarth, M., Rupprecht, S., Schlegel, A., Neureiter, D., & Kessler, P. (2004). [Central giant cell granuloma and osteitis fibrosa cystica of hyperparathyroidism. A challenge in differential diagnosis of patients with osteolytic jawbone lesions and a history of cancer]. Mund Kiefer Gesichtschir, 8(5), 316-321. doi:10.1007/s10006-004-0556-6

Threadgill, M. D. (2015). 5-Aminoisoquinolin-1-one (5-AIQ), a Water-Soluble Inhibitor of the Poly(ADP-Ribose)Polymerases (PARPs). Curr Med Chem, 22(33), 3807-3829. doi:10.2174/0929867322666151002110602

Thway, K., Fisher, C., & Sebire, N. J. (2012). Pediatric fibroblastic and myofibroblastic lesions. Adv Anat Pathol, 19(1), 54-65. doi:10.1097/PAP.0b013e31823d76ed

Tlaskalova-Hogenova, H., Stepankova, R., Hudcovic, T., Tuckova, L., Cukrowska, B., Lodinova-Zadnikova, R., . . . Kokesova, A. (2004). Commensal bacteria (normal microflora), mucosal immunity and chronic inflammatory and autoimmune diseases. Immunol Lett, 93(2-3), 97-108. doi:10.1016/j.imlet.2004.02.005

Tomeno, W., Imajo, K., Takayanagi, T., Ebisawa, Y., Seita, K., Takimoto, T., . . . Nakajima, A. (2020). Complications of Non-Alcoholic Fatty Liver Disease in Extrahepatic Organs. Diagnostics (Basel), 10(11). doi:10.3390/diagnostics10110912

Topaloglu, G., Koseoglu, O. T., Karaca, C., & Kosemehmetoglu, K. (2017). The effect of chronic dental inflammation on development of Stage 0 medication-related osteonecrosis of the jaw. J Craniomaxillofac Surg, 45(8), 1158-1164. doi:10.1016/j.jcms.2017.05.003

Torregrossa, V. R., Faria, K. M., Bicudo, M. M., Vargas, P. A., Almeida, O. P., Lopes, M. A., & Santos-Silva, A. R. (2016). Metastatic cervical carcinoma of the jaw presenting as periapical disease. Int Endod J, 49(2), 203-211. doi:10.1111/iej.12442

Toubi, E., Kessel, A., Bamberger, E., & Golan, T. D. (2004). Systemic Lupus Erythematosus Vasculitis: A Current Therapeutic Overview. Curr Treat Options Cardiovasc Med, 6(2), 87-97. doi:10.1007/s11936-004-0036-1

Toyokuni, S., & Akatsuka, S. (2007). Pathological investigation of oxidative stress in the post-genomic era. Pathol Int, 57(8), 461-473. doi:10.1111/j.1440-1827.2007.02127.x

Traber, M. G., & Stevens, J. F. (2011). Vitamins C and E: beneficial effects from a mechanistic perspective. Free Radic Biol Med, 51(5), 1000-1013. doi:10.1016/j.freeradbiomed.2011.05.017

Traish, A. M., Miner, M. M., Morgentaler, A., & Zitzmann, M. (2011). Testosterone deficiency. Am J Med, 124(7), 578-587. doi:10.1016/j.amjmed.2010.12.027

Translational Science 2012 Meeting Abstracts. (2012). Clinical and Translational Science, 5(2), 141-210. doi:10.1111/j.1752-8062.2012.00398.x

Trevillian, P. (2006). Immunosuppressants - Clinical applications. Australian Prescriber, 29(4), 102-108.

Tripathi, A., Kaymakcalan, M. D., LeBoeuf, N. R., & Harshman, L. C. (2016). Programmed cell death-1 pathway inhibitors in genitourinary malignancies: specific side-effects and their management. Curr Opin Urol, 26(6), 548-555. doi:10.1097/MOU.0000000000000332

Tripathy, D. (2008). Highlights from: The 44th Annual Meeting of the American Society of Clinical Oncology; Chicago, IL; May 30-June 3, 2008. Clinical breast cancer, 8(4), 313-321. doi:10.1016/s1526-8209(11)70524-9

Trost, L. W., Serefoglu, E., Gokce, A., Linder, B. J., Sartor, A. O., & Hellstrom, W. J. (2013). Androgen deprivation therapy impact on quality of life and cardiovascular health, monitoring therapeutic replacement. J Sex Med, 10 Suppl 1, 84-101. doi:10.1111/jsm.12036

True, E., Navratil, H., Grasset, D., Ciurana, A. J., Raux, A., & Navas, P. (1972). [Bourneville's tuberous sclerosis. Apropos of a case with predominantly renal localization]. J Urol Nephrol (Paris), 78(3), 270-275.

Tsao, C. W., Liu, C. Y., Cha, T. L., Wu, S. T., Chen, S. C., & Hsu, C. Y. (2015). Exploration of the association between chronic periodontal disease and erectile dysfunction from a population-based view point. Andrologia, 47(5), 513-518. doi:10.1111/and.12294

Tsianos, E. B., Karentzos, C., & Papadopoulos, N. E. (1987). Metastatic renal cell carcinoma in the gingiva of the maxilla and mandible: report of a case. J Oral Maxillofac Surg, 45(11), 975-977. doi:10.1016/0278-2391(87)90453-8

Tsuda, M., Davis, I. J., Argani, P., Shukla, N., McGill, G. G., Nagai, M., . . . Ladanyi, M. (2007). TFE3 fusions activate MET signaling by transcriptional up-regulation, defining another class of tumors as candidates for therapeutic MET inhibition. Cancer Res, 67(3), 919-929. doi:10.1158/0008-5472.CAN-06-2855

Tsung, J., & Burgess, D. J. (2012). Biodegradable polymers in drug delivery systems. In (Vol. 3, pp. 107-123).

Tunuguntla, H. S. G. R. (2005). Management of andropause: The male menopause. Clinical Geriatrics, 13(11), 27-34.

Turturro, A., Duffy, P., Hass, B., Kodell, R., & Hart, R. (2002). Survival characteristics and age-adjusted disease incidences in C57BL/6 mice fed a commonly used cereal-based diet modulated by dietary restriction. J Gerontol A Biol Sci Med Sci, 57(11), B379-389. doi:10.1093/gerona/57.11.b379

Tweeddale, D. N., Scott, R. C., Fields, M. J., Roddick, J. W., & Ball, M. J. (1968). Giant cells in cervico-vaginal smears. Acta Cytol, 12(4), 298-304.

Ugar-Cankal, D., & Ozmeric, N. (2006). A multifaceted molecule, nitric oxide in oral and periodontal diseases. Clin Chim Acta, 366(1-2), 90-100. doi:10.1016/j.cca.2005.10.018

Urasaki, T., Nakano, K., Tomomatsu, J., Komai, Y., Yuasa, T., Yamashita, K., . . . Takahashi, S. (2021). Adult genitourinary sarcoma: The era of optional chemotherapeutic agents for soft tissue sarcoma. Int J Urol, 28(1), 91-97. doi:10.1111/iju.14417

Uwitonze, A. M., Ojeh, N., Murererehe, J., Atfi, A., & Razzaque, M. S. (2020). Zinc Adequacy Is Essential for the Maintenance of Optimal Oral Health. Nutrients, 12(4). doi:10.3390/nu12040949

Vahtsevanos, K., Kyrgidis, A., Verrou, E., Katodritou, E., Triaridis, S., Andreadis, C. G., . . . Antoniades, K. (2009). Longitudinal cohort study of risk factors in cancer patients of bisphosphonate-related osteonecrosis of the jaw. J Clin Oncol, 27(32), 5356-5362. doi:10.1200/JCO.2009.21.9584

Valverde, P. (2008). Pharmacotherapies to manage bone loss-associated diseases: a quest for the perfect benefit-to-risk ratio. Curr Med Chem, 15(3), 284-304. doi:10.2174/092986708783497274

Van der Cruyssen, F., Grisar, K., Maes, H., & Politis, C. (2017). Case of a cerebral abscess caused by Porphyromonas gingivalis in a subject with periodontitis. BMJ Case Rep, 2017. doi:10.1136/bcr-2016-218845

van Dijk, E. H. C., Fauser, S., Breukink, M. B., Blanco-Garavito, R., Groenewoud, J. M. M., Keunen, J. E. E., . . . Boon, C. J. F. (2018). Half-Dose Photodynamic Therapy versus High-Density Subthreshold Micropulse Laser Treatment in Patients with Chronic Central Serous Chorioretinopathy: The PLACE Trial. Ophthalmology, 125(10), 1547-1555. doi:10.1016/j.ophtha.2018.04.021

van Veelen, W., Korsse, S. E., van de Laar, L., & Peppelenbosch, M. P. (2011). The long and winding road to rational treatment of cancer associated with LKB1/AMPK/TSC/mTORC1 signaling. Oncogene, 30(20), 2289-2303. doi:10.1038/onc.2010.630

van Westreenen, H. L., Westerterp, M., Jager, P. L., van Dullemen, H. M., Sloof, G. W., Comans, E. F., . . . Plukker, J. T. (2005). Synchronous primary neoplasms detected on 18F-FDG PET in staging of patients with esophageal cancer. J Nucl Med, 46(8), 1321-1325.

Vandenbroucke, R. E., & Libert, C. (2014). Is there new hope for therapeutic matrix metalloproteinase inhibition? Nat Rev Drug Discov, 13(12), 904-927. doi:10.1038/nrd4390

Vandooren, J., Van den Steen, P. E., & Opdenakker, G. (2013). Biochemistry and molecular biology of gelatinase B or matrix metalloproteinase-9 (MMP-9): the next decade. Crit Rev Biochem Mol Biol, 48(3), 222-272. doi:10.3109/10409238.2013.770819

Vardas, E., Nicolatou-Galitis, O., Papadopoulou, E., Kouri, M., Galiti, D., Torres, S., . . . Lalla, R. V. (2018). Abstracts of the MASCC/ISOO Annual Meeting 2018. Support Care Cancer, 26(2), 39-364. doi:10.1007/s00520-018-4193-2

Varshosaz, J. (2007). The promise of chitosan microspheres in drug delivery systems. Expert Opin Drug Deliv, 4(3), 263-273. doi:10.1517/17425247.4.3.263

Vasishta, P. A., Anjaneyalu, K., Sivaswamy, V., & Anjaneyalu, K. (2020). Lasers in Endodontics- A Review of Literature. Indian Journal of Forensic Medicine & Toxicology, 14(4), 4544-4550. doi:10.37506/ijfmt.v14i4.12357

Velangi, S. (2016). Invited Speaker Abstracts. Journal of Lower Genital Tract Disease, 20(4S), S1-S17. doi:10.1097/lgt.0000000000000257

Ventura, E., Dionisio, S., Ferreira, A., Saleiro, R., Marques, H., Magalhaes, M., & Monteiro, C. (2020). Maxillary mesenchymal chondrosarcoma leading to a diagnosis of Li-Fraumeni syndrome. J Surg Case Rep, 2020(1), rjz386. doi:10.1093/jscr/rjz386

Verhulst, M. J. L., Loos, B. G., Gerdes, V. E. A., & Teeuw, W. J. (2019). Evaluating All Potential Oral Complications of Diabetes Mellitus. Front Endocrinol (Lausanne), 10, 56. doi:10.3389/fendo.2019.00056

Vidal-Real, C., Perez-Sayans, M., Suarez-Penaranda, J. M., Gandara-Rey, J. M., & Garcia-Garcia, A. (2015). Osteonecrosis of the jaws in 194 patients who have undergone intravenous bisphosphonate therapy in Spain. Med Oral Patol Oral Cir Bucal, 20(3), e267-272. doi:10.4317/medoral.20092

Vieira, A. T., Castelo, P. M., Ribeiro, D. A., & Ferreira, C. M. (2017). Influence of Oral and Gut Microbiota in the Health of Menopausal Women. Front Microbiol, 8, 1884. doi:10.3389/fmicb.2017.01884

Vijayaraghavan, N., Karunanithi, G., Karthikeyan, A. K., & Basu, D. (2018). Therapy-Related Acute Myeloid Leukaemia in a Patient with Carcinoma Cervix Post-chemo-radiation Using Cisplatin. Indian Journal of Gynecologic Oncology, 16(1). doi:10.1007/s40944-018-0176-y

Vitetta, L., Briskey, D., Hayes, E., Shing, C., & Peake, J. (2012). A review of the pharmacobiotic regulation of gastrointestinal inflammation by probiotics, commensal bacteria and prebiotics. Inflammopharmacology, 20(5), 251-266. doi:10.1007/s10787-012-0126-8

Vogtmann, E., Hua, X., Yu, G., Hullings, A., Wan, Y., Dagnall, C. L., . . . et al. (2020). The human oral microbiota and risk of lung cancer: an analysis of three prospective cohort studies. Cancer Research, 80(8). doi:10.1158/1538-7445.MVC2020-A39

Vos, T., Abajobir, A. A., Abate, K. H., Abbafati, C., Abbas, K. M., Abd-Allah, F., . . . Murray, C. J. L. (2017). Global, regional, and national incidence, prevalence, and years lived with disability for 328 diseases and injuries for 195 countries, 1990–2016: a systematic analysis for the Global Burden of Disease Study 2016. The Lancet, 390(10100), 1211-1259. doi:10.1016/s0140-6736(17)32154-2

Vos, T., Barber, R. M., Bell, B., Bertozzi-Villa, A., Biryukov, S., Bolliger, I., . . . Murray, C. J. L. (2015). Global, regional, and national incidence, prevalence, and years lived with disability for 301 acute and chronic diseases and injuries in 188 countries, 1990–2013: a systematic analysis for the Global Burden of Disease Study 2013. The Lancet, 386(9995), 743-800. doi:10.1016/s0140-6736(15)60692-4

Voss, M. H., Bhatt, R. S., Plimack, E. R., Rini, B. I., Alter, R. S., Beck, J. T., . . . Atkins, M. B. (2017). The DART Study: Results from the Dose-Escalation and Expansion Cohorts Evaluating the Combination of Dalantercept plus Axitinib in Advanced Renal Cell Carcinoma. Clin Cancer Res, 23(14), 3557-3565. doi:10.1158/1078-0432.CCR-16-2395

Wada, N., Wang, B., Lin, N. H., Laslett, A. L., Gronthos, S., & Bartold, P. M. (2011). Induced pluripotent stem cell lines derived from human gingival fibroblasts and periodontal ligament fibroblasts. J Periodontal Res, 46(4), 438-447. doi:10.1111/j.1600-0765.2011.01358.x

Wakasugi, N., Uchida, H., & Uno, S. (2018). Safety and Effectiveness of Once-Daily, Prolonged-Release Tacrolimus in De Novo Kidney Transplant Recipients: 5-year, Multicenter Postmarketing Surveillance in Japan. Transplant Proc, 50(10), 3296-3305. doi:10.1016/j.transproceed.2018.08.049

Wald, N. J., & Hackshaw, A. K. (1996). Cigarette smoking: an epidemiological overview. Br Med Bull, 52(1), 3-11. doi:10.1093/oxfordjournals.bmb.a011530

Walker, R. H., & Reed, W. B. (1973). Genetic cutaneous disorders with gynecologic tumors. Am J Obstet Gynecol, 116(4), 485-492. doi:10.1016/0002-9378(73)90904-6

Walsh, T., Macey, R., Riley, P., Glenny, A. M., Schwendicke, F., Worthington, H. V., . . . Sengupta, A. (2021). Imaging modalities to inform the detection and diagnosis of early caries. Cochrane Database Syst Rev, 3(3), CD014545. doi:10.1002/14651858.CD014545

Walter, C., Grotz, K. A., Kunkel, M., & Al-Nawas, B. (2007). Prevalence of bisphosphonate associated osteonecrosis of the jaw within the field of osteonecrosis. Support Care Cancer, 15(2), 197-202. doi:10.1007/s00520-006-0120-z

Walter, C., Laux, C., & Sagheb, K. (2014). Radiologic bone loss in patients with bisphosphonate-associated osteonecrosis of the jaws: a case-control study. Clin Oral Investig, 18(2), 385-390. doi:10.1007/s00784-013-0974-7

Walters, G. D., Willis, N. S., Cooper, T. E., & Craig, J. C. (2020). Interventions for renal vasculitis in adults. Cochrane Database Syst Rev, 1(1), CD003232. doi:10.1002/14651858.CD003232.pub4

Wang, H., Sun, L., & Tan, W. (2015). Clinical features of children with pulmonary microscopic polyangiitis: report of 9 cases. PLoS One, 10(4), e0124352. doi:10.1371/journal.pone.0124352

Wang, Q., Cai, M., Shi, B. Y., Qian, Y. Y., Li, Z. L., Li, X. L., . . . Liang, T. (2009). Rapamycin combined immunosuppression for 12 renal transplant recipients. Journal of Clinical Rehabilitative Tissue Engineering Research, 13(44), 8749-8752. doi:10.3969/j.issn.1673-8225.2009.44.034

Wang, Q., Kessler, M. J., Kensler, T. B., & Dechow, P. C. (2016). The mandibles of castrated male rhesus macaques (Macaca mulatta): The effects of orchidectomy on bone and teeth. Am J Phys Anthropol, 159(1), 31-51. doi:10.1002/ajpa.22833

Wang, S., Zhang, J., Chen, M., & Wang, Y. (2013). Delivering flavonoids into solid tumors using nanotechnologies. Expert Opin Drug Deliv, 10(10), 1411-1428. doi:10.1517/17425247.2013.807795

Wang, X., Chen, W., & Yuan, Y. (2020). KSHV enhances mesenchymal stem cell homing and promotes KS-like pathogenesis. Virology, 549, 5-12. doi:10.1016/j.virol.2020.07.012

Watanabe, N., Sakamoto, K., Taniguchi, H., Kondoh, Y., Kimura, T., Kataoka, K., . . . Hasegawa, Y. (2014). Efficacy of combined therapy with cyclosporin and low-dose prednisolone in interstitial pneumonia associated with connective tissue disease. Respiration, 87(6), 469-477. doi:10.1159/000358098

Watson, C. J., & Dark, J. H. (2012). Organ transplantation: historical perspective and current practice. Br J Anaesth, 108 Suppl 1(SUPPL. 1), i29-42. doi:10.1093/bja/aer384

Watts, N. B., Chesnut, C. H., 3rd, Genant, H. K., Harris, S. T., Jackson, R. D., Licata, A. A., . . . Valent, D. (2020). History of etidronate. Bone, 134, 115222. doi:10.1016/j.bone.2020.115222

Waziry, R., Jawad, M., Ballout, R. A., Al Akel, M., & Akl, E. A. (2017). The effects of waterpipe tobacco smoking on health outcomes: an updated systematic review and meta-analysis. Int J Epidemiol, 46(1), 32-43. doi:10.1093/ije/dyw021

Weber, K. L., Doucet, M., Shaner, A., Hsu, N., Huang, D., Fogel, J., & Kominsky, S. L. (2012). MIP-1delta activates NFATc1 and enhances osteoclastogenesis: involvement of both PLCgamma2 and NFkappaB signaling. PLoS One, 7(7), e40799. doi:10.1371/journal.pone.0040799

Wei, M. Y., Kawachi, I., Okereke, O. I., & Mukamal, K. J. (2016). Diverse Cumulative Impact of Chronic Diseases on Physical Health-Related Quality of Life: Implications for a Measure of Multimorbidity. Am J Epidemiol, 184(5), 357-365. doi:10.1093/aje/kwv456

Weimer, R., Susal, C., Yildiz, S., Staak, A., Pelzl, S., Renner, F., . . . Opelz, G. (2006). Post-transplant sCD30 and neopterin as predictors of chronic allograft nephropathy: impact of different immunosuppressive regimens. Am J Transplant, 6(8), 1865-1874. doi:10.1111/j.1600-6143.2006.01407.x

Weng, H., Zeng, X. T., Wang, X. H., Liu, T. Z., & He, D. L. (2017). Genetic Association between Matrix Metalloproteinases Gene Polymorphisms and Risk of Prostate Cancer: A Meta-Analysis. Front Physiol, 8, 975. doi:10.3389/fphys.2017.00975

Weyer, G. W., Dunlap, B., & Shah, S. D. (2016). Hypertension in Women: Evaluation and Management. Obstet Gynecol Clin North Am, 43(2), 287-306. doi:10.1016/j.ogc.2016.01.002

White, E. S., & Lynch, J. P., 3rd. (2007). Current and emerging strategies for the management of sarcoidosis. Expert Opin Pharmacother, 8(9), 1293-1311. doi:10.1517/14656566.8.9.1293

Wibowo, E., Pollock, P. A., Hollis, N., & Wassersug, R. J. (2016). Tamoxifen in men: a review of adverse events. Andrology, 4(5), 776-788. doi:10.1111/andr.12197

Williams, F., Annetti, K., & Nagy, D. (2018). Cutaneous mast cell tumour and renal tubular adenocarcinoma in a Vietnamese potbellied pig. Veterinary Record Case Reports, 6(1). doi:10.1136/vetreccr-2017-000533

Willis, M., & Robertson, N. P. (2014). Drug safety evaluation of alemtuzumab for multiple sclerosis. Expert Opin Drug Saf, 13(8), 1115-1124. doi:10.1517/14740338.2014.928691

Wilson, B., & Plavsic, S. K. (2012). Role of Ultrasound in the Evaluation of Abnormal Vaginal Bleeding in Nonpregnant Patients in Reproductive Age. Donald School Journal of Ultrasound in Obstetrics and Gynecology, 6(1), 112-120. doi:10.5005/jp-journals-10009-1233

Wimalawansa, S. J. (2008). Insight into bisphosphonate-associated osteomyelitis of the jaw: pathophysiology, mechanisms and clinical management. Expert Opin Drug Saf, 7(4), 491-512. doi:10.1517/14740338.7.4.491

Wisanuyotin, S., & Jiravuttipong, A. (2009). Pediatric renal transplantation: a single-center experience in northeast Thailand. J Med Assoc Thai, 92(12), 1635-1639.

Wischke, C., & Schwendeman, S. P. (2012). Degradable polymeric carriers for parenteral controlled drug delivery. In (Vol. 3, pp. 171-228).

Wolach, B., Gavrieli, R., de Boer, M., Gottesman, G., Ben-Ari, J., Rottem, M., . . . Roos, D. (2008). Chronic granulomatous disease in Israel: clinical, functional and molecular studies of 38 patients. Clin Immunol, 129(1), 103-114. doi:10.1016/j.clim.2008.06.012

Wolf, B. J., Hill, E. G., & Slate, E. H. (2010). Logic Forest: an ensemble classifier for discovering logical combinations of binary markers. Bioinformatics, 26(17), 2183-2189. doi:10.1093/bioinformatics/btq354

Women and screening. Preventing, detecting common health problems. (2007). Mayo Clin Womens Healthsource, Suppl, 1-8.

Wong, C. J., & Pagalilauan, G. (2015). Primary Care of the Solid Organ Transplant Recipient. Med Clin North Am, 99(5), 1075-1103. doi:10.1016/j.mcna.2015.05.002

Woo, B. H., Kim, D. J., Choi, J. I., Kim, S. J., Park, B. S., Song, J. M., . . . Park, H. R. (2017). Oral cancer cells sustainedly infected with Porphyromonas gingivalis exhibit resistance to Taxol and have higher metastatic potential. Oncotarget, 8(29), 46981-46992. doi:10.18632/oncotarget.16550

Woodford, R., Jackson-Boeters, L., Darling, M., Shimizu, M., & Daley, T. (2013). Selected Human Kallikrein (KLK) Expression in Odontogenic Cysts and Tumors. Journal of Oral and Maxillofacial Surgery, 71(9), e36-e37. doi:10.1016/j.joms.2013.06.063

Woolacott, N., Bravo Vergel, Y., Hawkins, N., Kainth, A., Khadjesari, Z., Misso, K., . . . Riemsma, R. (2006). Etanercept and infliximab for the treatment of psoriatic arthritis: a systematic review and economic evaluation. Health Technol Assess, 10(31), iii-iv, xiii-xvi, 1-239. doi:10.3310/hta10310

Workalemahu, G., Abdela, O. A., & Yenit, M. K. (2020). Chemotherapy-Related Adverse Drug Reaction and Associated Factors Among Hospitalized Paediatric Cancer Patients at Hospitals in North-West Ethiopia. Drug Healthc Patient Saf, 12, 195-205. doi:10.2147/DHPS.S254644

Wu, C. S., Yang, T. H., Lin, H. C., Sheu, J. J., & Chu, D. (2013). Sudden sensorineural hearing loss associated with chronic periodontitis: a population-based study. Otol Neurotol, 34(8), 1380-1384. doi:10.1097/MAO.0b013e3182a1e925

Wu, J. S., Zheng, M., Zhang, M., Pang, X., Li, L., Wang, S. S., . . . Liang, X. H. (2018). Porphyromonas gingivalis Promotes 4-Nitroquinoline-1-Oxide-Induced Oral Carcinogenesis With an Alteration of Fatty Acid Metabolism. Front Microbiol, 9, 2081. doi:10.3389/fmicb.2018.02081

Wu, J. Y., Wu, X. N., Ding, L., Zhao, Y. B., Ai, B., Li, Y., . . . Cheng, G. (2010). Phase I safety and pharmacokinetic study of bevacizumab in Chinese patients with advanced cancer. Chin Med J (Engl), 123(7), 901-906. doi:10.3760/cma.j.issn.0366-6999.2010.07.025

Wu, L., Li, B. H., Wang, Y. Y., Wang, C. Y., Zi, H., Weng, H., . . . Zeng, X. T. (2019). Periodontal disease and risk of benign prostate hyperplasia: a cross-sectional study. Mil Med Res, 6(1), 34. doi:10.1186/s40779-019-0223-8

Wu, P. Y., Cheng, Y. M., Shen, M. R., Chen, Y. C., Huang, Y. F., & Chou, C. Y. (2020). Real-World Study of Adding Bevacizumab to Chemotherapy for Ovarian, Tubal, and Peritoneal Cancer as Front-Line or Relapse Therapy (ROBOT): 8-Year Experience. Front Oncol, 10, 1095. doi:10.3389/fonc.2020.01095

Wu, S., Guo, Y., Liu, C., Liu, Q., Deng, H., & Yuan, L. (2021). Identification of a de novo TSC2 variant in a Han-Chinese family with tuberous sclerosis complex. J Chin Med Assoc, 84(1), 46-50. doi:10.1097/JCMA.0000000000000455

Wu, W. K., Sung, J. J., Cheng, A. S., Chan, F. K., Ng, S. S., To, K. F., . . . Cho, C. H. (2014). The Janus face of cathelicidin in tumorigenesis. Curr Med Chem, 21(21), 2392-2400. doi:10.2174/0929867321666140205135351

Wu, Y. T. (1990). [Metastatic carcinoma to the oral tissues and jaws: a study of 25 cases]. Zhonghua Kou Qiang Yi Xue Za Zhi, 25(5), 258-261, 317.

Wuketich, S., Hienz, S. A., & Marosi, C. (2012). Prevalence of clinically relevant oral mucositis in outpatients receiving myelosuppressive chemotherapy for solid tumors. Support Care Cancer, 20(1), 175-183. doi:10.1007/s00520-011-1107-y

Xu, Q. L., Furuhashi, A., Zhang, Q. Z., Jiang, C. M., Chang, T. H., & Le, A. D. (2017). Induction of Salivary Gland-Like Cells from Dental Follicle Epithelial Cells. J Dent Res, 96(9), 1035-1043. doi:10.1177/0022034517711146

Yacoub, A. T., Krishnan, J., Acevedo, I. M., Halliday, J., & Greene, J. N. (2015). Nutritionally variant streptococci bacteremia in cancer patients: a retrospective study, 1999-2014. Mediterr J Hematol Infect Dis, 7(1), e2015030. doi:10.4084/MJHID.2015.030

Yahyaoui, Y., Zenzri, Y., Adouni, O., Letaief, F., Driss, M., & Mezlini, A. (2020). Prostate adenorcarcinoma revealed by gingival metastasis: An uncommon presentation. Urol Case Rep, 31, 101156. doi:10.1016/j.eucr.2020.101156

Yamamoto, T., Natio, M., Hara, S., Kudo, T., & Miwa, Y. (2016). The effect of aprepitant and dexamethasone combination on paclitaxel-induced hypersensitivity reaction. Eur J Gynaecol Oncol, 37(6), 833-836. doi:10.12892/ejgo3362.2016

Yang, J., Wise, L., & Fukuchi, K. I. (2020). TLR4 Cross-Talk With NLRP3 Inflammasome and Complement Signaling Pathways in Alzheimer's Disease. Front Immunol, 11, 724. doi:10.3389/fimmu.2020.00724

Yang, P. (1983). [Nursing of patients with stomatocace after chemotherapy for choriocarcinoma]. Zhonghua Hu Li Za Zhi, 18(2), 116-117.

Yang, R.-H., Ting, C.-H., & Chu, Y.-K. (2016). Cannonball lung metastases as a presenting feature of ectopic hCG expression. Journal of Oncological Sciences, 2(2-3), 58-62. doi:10.1016/j.jons.2016.07.003

Yang, Y., Tang, L. Q., & Wei, W. (2013). Prostanoids receptors signaling in different diseases/cancers progression. J Recept Signal Transduct Res, 33(1), 14-27. doi:10.3109/10799893.2012.752003

Yazdi, I., & Nowparast, B. (1974). Extraosseous adenomatoid odontogenic tumor with special reference to the probability of the basal-cell layer of oral epithelium as a potential source of origin. Oral Surgery, Oral Medicine, Oral Pathology, 37(2), 249-256. doi:10.1016/0030-4220(74)90420-4

Yoon, J., Seo, H., Oh, I. H., & Yoon, S. J. (2016). The Non-Communicable Disease Burden in Korea: Findings from the 2012 Korean Burden of Disease Study. J Korean Med Sci, 31 Suppl 2, S158-S167. doi:10.3346/jkms.2016.31.S2.S158

Yoon, M. S., Jankowski, V., Montag, S., Zidek, W., Henning, L., Schluter, H., . . . Jankowski, J. (2004). Characterisation of advanced glycation endproducts in saliva from patients with diabetes mellitus. Biochem Biophys Res Commun, 323(2), 377-381. doi:10.1016/j.bbrc.2004.08.118

Yoshikawa, K., Tsuchiya, A., Kido, T., Ota, T., Ikeda, K., Iwakura, M., . . . Maekawa, S. (2020). Long-Term Safety and Efficacy of Sitagliptin for Type 2 Diabetes Mellitus in Japan: Results of a Multicentre, Open-Label, Observational Post-Marketing Surveillance Study. Adv Ther, 37(5), 2442-2459. doi:10.1007/s12325-020-01293-2

Young-Choi, P., Ng, S. C., Lee, C., Roncolato, F., Badoux, X., Hugman, A. J., . . . Chong, B. (2013). Triple therapy for immune thrombocytopenia: A novel combination of conventional strategies to safely sustain platelet counts in ITP. Blood, 122(21).

Yu, C.-L., & Tsai, M.-H. (2001). Fetal fetuin selectively induces apoptosis in cancer cell lines and shows anti-cancer activity in tumor animal models. Cancer Letters, 166(2), 173-184. doi:10.1016/s0304-3835(01)00417-7

Zandieh, F., Ghazi, B. M., Izadi, A., Gharegozlu, M., Aghajani, M., & Sheikh, M. (2014). Papillon lefevre syndrome and footprints of mycobacterium tuberculosis. Iranian Journal of Allergy, Asthma and Immunology, 13(4), 286-289.

Zebic, L., & Patel, V. (2019). Preventing medication-related osteonecrosis of the jaw. Bmj, 365, l1733. doi:10.1136/bmj.l1733

Zhan, S., Li, J., & Ge, W. (2019). Multifaceted Roles of Asporin in Cancer: Current Understanding. Front Oncol, 9, 948. doi:10.3389/fonc.2019.00948

Zhang, H. L., Qin, X. J., Wang, H. K., Gu, W. J., Ma, C. G., Shi, G. H., . . . Ye, D. W. (2015). Clinicopathological and prognostic factors for long-term survival in Chinese patients with metastatic renal cell carcinoma treated with sorafenib: a single-center retrospective study. Oncotarget, 6(34), 36870-36883. doi:10.18632/oncotarget.4874

Zhang, J., Sun, X., Xiao, L., Xie, C., Xuan, D., & Luo, G. (2011). Gene polymorphisms and periodontitis. Periodontol 2000, 56(1), 102-124. doi:10.1111/j.1600-0757.2010.00371.x

Zhang, M., Zhu, B., & Davie, J. (2015). Alternative splicing of MEF2C pre-mRNA controls its activity in normal myogenesis and promotes tumorigenicity in rhabdomyosarcoma cells. J Biol Chem, 290(1), 310-324. doi:10.1074/jbc.M114.606277

Zhang, R., & Naughton, D. P. (2010). Vitamin D in health and disease: current perspectives. Nutr J, 9(1), 65. doi:10.1186/1475-2891-9-65

Zhang, S. M., Tian, F., Jiang, X. Q., Li, J., Xu, C., Guo, X. K., & Zhang, F. Q. (2009). Evidence for calcifying nanoparticles in gingival crevicular fluid and dental calculus in periodontitis. J Periodontol, 80(9), 1462-1470. doi:10.1902/jop.2009.080659

Zhang, W., Zhou, A. P., Qin, Q., Chang, C. X., Jiang, H. Y., Ma, J. H., & Wang, J. W. (2013). Famitinib in metastatic renal cell carcinoma: a single center study. Chin Med J (Engl), 126(22), 4277-4281. doi:10.3760/cma.j.issn.0366-6999.20131757

Zhang, Y., Chen, J., Wei, W., Zheng, L., Li, H., Feng, Y., . . . Huang, H. (2020). Anlotinib (AL3818) plus pemetrexed in patients with recurrent platinum-resistant advanced epithelial ovarian cancer: A single-arm, open-label, phase II study. Journal of Clinical Oncology, 38(15). doi:10.1200/JCO.2020.38.15-suppl.e18078

Zhang, Y., Knutsen, G. R., Brown, M. D., & Ruest, L. B. (2013). Control of endothelin-a receptor expression by progesterone is enhanced by synergy with Gata2. Mol Endocrinol, 27(6), 892-908. doi:10.1210/me.2012-1334

Zheng, C. J., Han, L. Y., Yap, C. W., Xie, B., & Chen, Y. Z. (2005). Trends in exploration of therapeutic targets. Drug News Perspect, 18(2), 109-127. doi:10.1358/dnp.2005.18.2.886480

Zhou, M., Hu, H., Han, Y., Li, J., Zhang, Y., Tang, S., . . . Zhang, X. (2021). Long non-coding RNA 01126 promotes periodontitis pathogenesis of human periodontal ligament cells via miR-518a-5p/HIF-1alpha/MAPK pathway. Cell Prolif, 54(1), e12957. doi:10.1111/cpr.12957

Zhou, S., Chan, E., Lim, L. Y., Boelsterli, U. A., Li, S. C., Wang, J., . . . Xu, A. (2004). Therapeutic drugs that behave as mechanism-based inhibitors of cytochrome P450 3A4. Curr Drug Metab, 5(5), 415-442. doi:10.2174/1389200043335450

Zhu, J., Zhang, S., Zhu, L., Li, X., Wang, Y., Duan, Y., & Huang, W. (2015). Primary testicular Ph-positive B lymphoblastic lymphoma: an unusual presentation and review. Cancer Biol Ther, 16(8), 1122-1127. doi:10.1080/15384047.2015.1056412

Zhu, S. M., Guo, S. H., Li, L. J., Luo, L. H., & Yao, Y. X. (2013). Successful management of an intra-operative pulmonary tumor embolism during resection of a retroperitoneal leiomyosarcoma. Chin Med J (Engl), 126(5), 980-981. doi:10.3760/cma.j.issn.0366-6999.20121080

Zhumagaliyeva, A., Nurgaliyeva, D., & Karazhanova, L. (2018). 23rd Congress of the European Hematology Association Stockholm, Sweden, June 14-17, 2018. HemaSphere, 2(S1), 1-1113. doi:10.1097/hs9.0000000000000060

Zielinski, S. L., & Travis, K. (2004). Surgeon general's report strengthens position on tobacco-related diseases. Journal of the National Cancer Institute, 96(12), 905-905.

Zinzani, P. L. (2006). Salvage chemotherapy in follicular non-Hodgkin's lymphoma: focus on tolerability. Clin Lymphoma Myeloma, 7(2), 115-124. doi:10.3816/CLM.2006.n.048

Zitvogel, L., & Kroemer, G. (2021). Cross-reactivity between cancer and microbial antigens. Oncoimmunology, 10(1), 1877416. doi:10.1080/2162402X.2021.1877416

Zou, H., Kang, X., Pang, L. J., Hu, W., Zhao, J., Qi, Y., . . . Li, F. (2014). Xp11 translocation renal cell carcinoma in adults: a clinicopathological and comparative genomic hybridization study. Int J Clin Exp Pathol, 7(1), 236-245.

Zou, X. L., Zeng, K., Xie, L. P., Wang, L., Chen, M., Liu, T., & Niu, T. (2013). Acute promyelocytic leukemia with Flt3-TKD and WT1 mutations relapsing in a testicle and followed by systemic relapse. Acta Haematol, 130(4), 223-229. doi:10.1159/000351054

Zupin, L., Polesello, V., Coelho, A. V., Boniotto, M., Arraes, L. C., Segat, L., & Crovella, S. (2015). Lactotransferrin gene functional polymorphisms do not influence susceptibility to human immunodeficiency virus-1 mother-to-child transmission in different ethnic groups. Mem Inst Oswaldo Cruz, 110(2), 222-229. doi:10.1590/0074-02760140447

Abstract of the 47th National Conference of Association of Clinical Biochemists of India. (2021). Indian Journal of Clinical Biochemistry, 36(SUPPL 1).

Akatsuka, J., Kimura, G., Obayashi, K., Tsutsumi, K., Yanagi, M., Endo, Y., . . . Kondo, Y. (2021). Outcomes of starting low-dose pazopanib in patients with metastatic renal cell carcinoma who do not meet eligibility criteria for clinical trials. Urological Science, 32(3), 104-110. doi:10.4103/UROS.UROS_145_20

Ala, M., & Ala, M. (2021). Metformin for Cardiovascular Protection, Inflammatory Bowel Disease, Osteoporosis, Periodontitis, Polycystic Ovarian Syndrome, Neurodegeneration, Cancer, Inflammation and Senescence: What Is Next? ACS PHARMACOLOGY & TRANSLATIONAL SCIENCE, 4(6), 1747-1770. doi:10.1021/acsptsci.1c00167

Almawash, S., Osman, S. K., Mustafa, G., & El Hamd, M. A. (2022). Current and Future Prospective of Injectable Hydrogels—Design Challenges and Limitations. Pharmaceuticals, 15(3). doi:10.3390/ph15030371

Aubeux, D., Peters, O. A., Hosseinpour, S., Tessier, S., Geoffroy, V., Perez, F., & Gaudin, A. (2021). Specialized pro-resolving lipid mediators in endodontics: a narrative review. BMC ORAL HEALTH, 21(1). doi:10.1186/s12903-021-01619-8

Avishai, G., Muchnik, D., Masri, D., Zlotogorski-Hurvitz, A., & Chaushu, L. (2022). Minimizing MRONJ after Tooth Extraction in Cancer Patients Receiving Bone-Modifying Agents. JOURNAL OF CLINICAL MEDICINE, 11(7). doi:10.3390/jcm11071807

Badmanaban, R., Saha, D., Sen, D. J., Biswas, A., Mandal, S., & Basak, S. (2021). Turmeric: A holistic solution for biochemical malfunction. Research Journal of Pharmacy and Technology, 14(10), 5540-5550. doi:10.52711/0974-360X.2021.00966

Baltanás, F. C., García-Navas, R., & Santos, E. (2021). Sos2 comes to the fore: Differential functionalities in physiology and pathology. INTERNATIONAL JOURNAL OF MOLECULAR SCIENCES, 22(12). doi:10.3390/ijms22126613

Barbosa, J. S., Almeida Paz, F. A., & Braga, S. S. (2021). Bisphosphonates, Old Friends of Bones and New Trends in Clinics. Journal of Medicinal Chemistry, 64(3), 1260-1282. doi:10.1021/acs.jmedchem.0c01292

Basilicata, M., Di Lauro, M., Campolattano, V., Marrone, G., Celotto, R., Mitterhofer, A. P., . . . Noce, A. (2022). Natural Bioactive Compounds in the Management of Oral Diseases in Nephropathic Patients. INTERNATIONAL JOURNAL OF ENVIRONMENTAL RESEARCH AND PUBLIC HEALTH, 19(3). doi:10.3390/ijerph19031665

Buchbender, M., Bauerschmitz, C., Pirkl, S., Kesting, M. R., & Schmitt, C. M. (2022). A Retrospective Data Analysis for the Risk Evaluation of the Development of Drug-Associated Jaw Necrosis through Dentoalveolar Interventions. INTERNATIONAL JOURNAL OF ENVIRONMENTAL RESEARCH AND PUBLIC HEALTH, 19(7). doi:10.3390/ijerph19074339

Caldas, R. J., Antunes, H. S., Pegoraro, C. D. R., Guedes, F. R., & Santos, P. S. D. (2021). Oral health condition in cancer patients under bisphosphonate therapy. SUPPORTIVE CARE IN CANCER, 29(12), 7687-7694. doi:10.1007/s00520-021-06362-y

Carneiro-Leão, D., Fernandes, S., Carvalho, M., Lopes, M., & Koch, C. (2021). Severe menorrhagia and glanzmann's thrombasthenia. the challenge of more invasive procedures. a case-report. Haemophilia, 27(SUPPL 2), 162. doi:10.1111/hae.14236

Carrieri, A. P., Haiminen, N., Maudsley-Barton, S., Gardiner, L. J., Murphy, B., Mayes, A. E., . . . Pyzer-Knapp, E. O. (2021). Explainable AI reveals changes in skin microbiome composition linked to phenotypic differences. SCIENTIFIC REPORTS, 11(1). doi:10.1038/s41598-021-83922-6

Chen, L., Chen, Q., Zhu, M., & Zhuang, Z. (2021). Dalteparin and rivaroxaban sequential use in cancer patients with venous thromboembolism. Journal of the College of Physicians and Surgeons Pakistan, 31(3), 294-297. doi:10.29271/JCPSP.2021.03.294

Chen, P., Chen, X., Chu, H., Xia, W., Zou, X., Wang, D., & Rong, M. (2021). Periodontitis regulates renal impairment in obese mice via TGF-β/Smad pathway. American Journal of Translational Research, 13(11), 12523-12535.

Choueiri, T. K., Zakharia, Y., Pal, S., Kocsis, J., Pachynski, R., Poprach, A., . . . Agarwal, N. (2021). Clinical Results and Biomarker Analyses of Axitinib and TRC105 versus Axitinib Alone in Patients with Advanced or Metastatic Renal Cell Carcinoma (TRAXAR). Oncologist, 26(7), 560-e1103. doi:10.1002/onco.13777

CONTENTS. (2021). Biochemical and Cellular Archives, 21(2), 1-2.

Das, N., Benko, C., Gill, S. E., & Dufour, A. (2021). The pharmacological TAILS of matrix metalloproteinases and their inhibitors. Pharmaceuticals, 14(1), 1-17. doi:10.3390/ph14010031

Dong, J. L., Li, Y., Xiao, H. W., Cui, M., & Fan, S. J. (2022). Commensal microbiota in the digestive tract: a review of its roles in carcinogenesis and radiotherapy. CANCER BIOLOGY & MEDICINE, 19(1), 43-55. doi:10.20892/j.issn.2095-3941.2020.0476

Duijster, J. W., Franz, E., Neefjes, J., & Mughini-Gras, L. (2021). Bacterial and Parasitic Pathogens as Risk Factors for Cancers in the Gastrointestinal Tract: A Review of Current Epidemiological Knowledge. Frontiers in Microbiology, 12. doi:10.3389/fmicb.2021.790256

Dziewas, R., Michou, E., Trapl-Grundschober, M., Lal, A., Arsava, E. M., Bath, P. M., . . . Verin, E. (2021). European Stroke Organisation and European Society for Swallowing Disorders guideline for the diagnosis and treatment of post-stroke dysphagia. European Stroke Journal, 6(3), LXXXIX-CXV. doi:10.1177/23969873211039721

Fang, C., Wu, L., Zhu, C., Xie, W. Z., Hu, H., & Zeng, X. T. (2021). A potential therapeutic strategy for prostatic disease by targeting the oral microbiome. Med Res Rev, 41(3), 1812-1834. doi:10.1002/med.21778

Fang, Y., Yang, Y., Li, N., Zhang, X. L., & Huang, H. F. (2021). Emerging role of long noncoding RNAs in recurrent hepatocellular carcinoma. World Journal of Clinical Cases, 9(32), 9699-9710. doi:10.12998/wjcc.v9.i32.9699

Fang, Z., Wu, Y., Li, Y. P., Zhang, X. H., Willett, W. C., Eliassen, A. H., . . . Giovannucci, E. L. (2021). Association of nut consumption with risk of total cancer and 5 specific cancers: evidence from 3 large prospective cohort studies. AMERICAN JOURNAL OF CLINICAL NUTRITION, 114(6), 1925-1935. doi:10.1093/ajcn/nqab295

Ferrillo, M., Migliario, M., Roccuzzo, A., Molinero-Mourelle, P., Falcicchio, G., Umano, G. R., . . . de Sire, A. (2021). Periodontal disease and vitamin D deficiency in pregnant women: Which correlation with preterm and low-weight birth? JOURNAL OF CLINICAL MEDICINE, 10(19). doi:10.3390/jcm10194578

Fischer, R. G., Gomes, I. S., da Cruz, S. S., Oliveira, V. B., Lira, R., Scannapieco, F. A., & Rego, R. O. (2021). What is the future of Periodontal Medicine? BRAZILIAN ORAL RESEARCH, 35. doi:10.1590/1807-3107bor-2021.vol35.0102

Fujiwara, K., Fujiwara, H., Yoshida, H., Satoh, T., Yonemori, K., Nagao, S., . . . Ray-Coquard, I. (2021). Olaparib plus bevacizumab as maintenance therapy in patients with newly diagnosed, advanced ovarian cancer: Japan subset from the paola-1/engot-ov25 trial. Journal of Gynecologic Oncology, 32(5). doi:10.3802/JGO.2021.32.E82

Gondhowiardjo, S., Christina, N., Ganapati, N. P. D., Hawariy, S., Radityamurti, F., Jayalie, V. F., . . . Priyambodho. (2021). Five-year cancer epidemiology at the nationa referral hospital: Hospital-based cancer registry data in Indonesia. JCO Global Oncology, 7, 190-203. doi:10.1200/GO.20.00155

Gonzalez-Moles, M. A., & Ramos-Garcia, P. (2021). State of Evidence on Oral Health Problems in Diabetic Patients: A Critical Review of the Literature. JOURNAL OF CLINICAL MEDICINE, 10(22). doi:10.3390/jcm10225383

Guti, S., Baidya, S. K., Banerjee, S., Adhikari, N., & Jha, T. (2021). A robust classification-dependent multi-molecular modelling study on some biphenyl sulphonamide based MMP-8 inhibitors. SAR AND QSAR IN ENVIRONMENTAL RESEARCH, 32(10), 835-861. doi:10.1080/1062936X.2021.1976831

Hasegawa, T., Ueda, N., Yamada, S., Kato, S., Iwata, E., Hayashida, S., . . . Japanese Study Grp Cooperative, D. (2021). Denosumab-related osteonecrosis of the jaw after tooth extraction and the effects of a short drug holiday in cancer patients: a multicenter retrospective study. OSTEOPOROSIS INTERNATIONAL, 32(11), 2323-2333. doi:10.1007/s00198-021-05995-3

Hsiao, Y. C., Lee, Y. H., Ho, C. M., Tseng, C. H., & Wang, J. H. (2021). Clinical Characteristics of Actinomyces viscosus Bacteremia. MEDICINA-LITHUANIA, 57(10). doi:10.3390/medicina57101064

Hunt, C., Montgomery, S., Berkenpas, J. W., Sigafoos, N., Oakley, J. C. T., Espinosa, J., . . . Cao, R. (2022). Recent Progress of Machine Learning in Gene Therapy. Current Gene Therapy, 22(2), 132-143. doi:10.2174/1566523221666210622164133

Iglesias-Lopez, C., Obach, M., Vallano, A., & Agustí, A. (2021). Comparison of regulatory pathways for the approval of advanced therapies in the European Union and the United States. Cytotherapy, 23(3), 261-274. doi:10.1016/j.jcyt.2020.11.008

Irimia, P., Santos-Lasaosa, S., García Bujalance, L., Ramos Pinazo, L., Rubio-Rodríguez, D., & Rubio-Terrés, C. (2021). Cost of fremanezumab, erenumab, galcanezumab and onabotulinumtoxinA associated adverse events, for migraine prophylaxis in Spain. Expert Review of Pharmacoeconomics and Outcomes Research, 21(2), 285-297. doi:10.1080/14737167.2020.1768850

Jazdarehee, A., Huget-Penner, S., & Pawlowska, M. (2022). Pseudo-pheochromocytoma due to obstructive sleep apnea: a case report. ENDOCRINOLOGY DIABETES AND METABOLISM CASE REPORTS, 2022. doi:10.1530/EDM-21-0100

Jha, R. K., & Chandi, D. H. (2021). A Case Study on Multiple Myeloma. JOURNAL OF PHARMACEUTICAL RESEARCH INTERNATIONAL, 33(37B). doi:10.9734/JPRI/2021/v33i37B32032

Jovicic, S. M. (2021). Global trend of clinical biomarkers of health and disease during the period (1913–2021): systematic review and bibliometric analysis. African Journal of Urology, 27(1). doi:10.1186/s12301-021-00239-6

Jung, E., Romero, R., Yeo, L., Gomez-Lopez, N., Chaemsaithong, P., Jaovisidha, A., . . . Erez, O. (2022). The etiology of preeclampsia. American Journal of Obstetrics and Gynecology, 226(2), S844-S866. doi:10.1016/j.ajog.2021.11.1356

Kadkhoda, S., & Ghafouri-Fard, S. (2022). Function of miRNA-145–5p in the pathogenesis of human disorders. Pathology Research and Practice, 231. doi:10.1016/j.prp.2022.153780

Kato, I., Zhang, J. L., & Sun, J. (2022). Bacterial-Viral Interactions in Human Orodigestive and Female Genital Tract Cancers: A Summary of Epidemiologic and Laboratory Evidence. CANCERS, 14(2). doi:10.3390/cancers14020425

Kehm, R., Baldensperger, T., Raupbach, J., & Höhn, A. (2021). Protein oxidation - Formation mechanisms, detection and relevance as biomarkers in human diseases. Redox Biology, 42. doi:10.1016/j.redox.2021.101901

Khader, A., Valiaveetil, B., Nalini, S. D., George, B., Sasidharanpillai, S., Nazeer, M. M. A., & Manikath, N. (2021). Effect of monthly cyclophosphamide pulses on skin sclerosis in systemic sclerosis. Indian Journal of Dermatology, Venereology and Leprology, 87(5), 728-731. doi:10.25259/IJDVL_796_19

Khaliq, N. U., Chobisa, D., Richard, C. A., Swinney, M. R., & Yeo, Y. (2021). Engineering microenvironment of biodegradable polyester systems for drug stability and release control. Therapeutic Delivery, 12(1), 37-54. doi:10.4155/tde-2020-0113

Kia, S. J., Basirat, M., Saedi, H. S., & Arab, S. A. (2021). Effects of nanomicelle curcumin capsules on prevention and treatment of oral mucosits in patients under chemotherapy with or without head and neck radiotherapy: a randomized clinical trial. BMC Complementary Medicine and Therapies, 21(1). doi:10.1186/s12906-021-03400-4

Kim, K., Kim, J. H., Kim, S. C., Kim, Y. B., Nam, B. H., No, J. H., . . . Kim, Y. H. (2021). Modulated electro-hyperthermia with weekly paclitaxel or cisplatin in patients with recurrent or persistent epithelial ovarian, fallopian tube or primary peritoneal carcinoma: The KGOG 3030 trial. Experimental and Therapeutic Medicine, 22(1). doi:10.3892/ETM.2021.10219

Kondo, S., Shimizu, T., Koyama, T., Sato, J., Iwasa, S., Yonemori, K., . . . Yamamoto, N. (2021). First-in-human study of the cancer peptide vaccine TAS0313 in patients with advanced solid tumors. Cancer Science, 112(4), 1514-1523. doi:10.1111/cas.14765

Kulmann-Leal, B., Ellwanger, J. H., & Chies, J. A. B. (2021). CCR5Δ32 in Brazil: Impacts of a European Genetic Variant on a Highly Admixed Population. Frontiers in Immunology, 12. doi:10.3389/fimmu.2021.758358

Kwon, M., Lee, C., Lee, S., Shin, H., Park, S., Joung, C., & Lee, S. (2021). Signal detection of adverse drug reactions of biologic DMARDs used in ankylosing spondylitis patients on real-world data in South Korea. International Journal of Rheumatic Diseases, 24(SUPPL 2), 271. doi:10.1111/1756-185X.14200

Ladak, A. A., Sandhu, S., & Itrat, A. (2021). Use of Intravenous Thrombolysis in Acute Ischemic Stroke Management in Patients with Active Malignancies: A Topical Review. Journal of Stroke and Cerebrovascular Diseases, 30(6). doi:10.1016/j.jstrokecerebrovasdis.2021.105728

Lan, Y. F., Liu, B. Y., & Guo, H. B. (2021). The role of M(6)A modification in the regulation of tumor-related lncRNAs. MOLECULAR THERAPY-NUCLEIC ACIDS, 24, 768-779. doi:10.1016/j.omtn.2021.04.002

Lee, K. S., Kim, H. I., Kim, H. Y., Cho, G. J., Hong, S. C., Oh, M. J., . . . Ahn, K. H. (2021). Association of preterm birth with depression and particulate matter: Machine learning analysis using national health insurance data. Diagnostics, 11(3). doi:10.3390/diagnostics11030555

Luo, Y. H., Yang, Y. W., Wu, C. F., Wang, C., Li, W. J., & Zhang, H. C. (2021). Fatigue prevalence in men treated for prostate cancer: A systematic review and meta-analysis. World Journal of Clinical Cases, 9(21), 5932-5942. doi:10.12998/wjcc.v9.i21.5932

Malta, D. C., Passos, V. M. A., Vasconcelos, A. M. N., Carneiro, M., Gomes, C. S., & Ribeiro, A. L. P. (2022). Disease burden in Brazil and its states. Estimates from the Global Burden of Disease Study 2019. Revista da Sociedade Brasileira de Medicina Tropical, 55. doi:10.1590/0037-8682-0622-2021

Mao, M., Zhu, H., Xie, Y., Ni, D., Zhu, F., & Chen, Q. (2022). Correlation between periodontitis and prostate-specific antigen levels in the elderly Chinese male population. BMC ORAL HEALTH, 22(1), 163. doi:10.1186/s12903-022-02171-9

Meurman, J. H., & Bascones-Martinez, A. (2021). Oral Infections and Systemic Health- More than Just Links to Cardiovascular Diseases. ORAL HEALTH & PREVENTIVE DENTISTRY, 19(1), 441-448. doi:10.3290/j.ohpd.b1993965

Moazen, M., Bayani, M., Ranjbar, M., Shaddel, M., Amini, M., & Zamani, A. (2022). Comparing Gingival Crevicular Fluid Visfatin Levels Between Patients with Endometrial Cancer and Chronic Periodontitis and Healthy Controls. Journal of Mazandaran University of Medical Sciences, 32(207), 48-58.

Moest, T., Lutz, R., Jahn, A. E., Heller, K., Schiffer, M., Adler, W., . . . Kesting, M. R. (2021). Oral health of patients suffering from end-stage solid organ insufficiency prior to solid organ re-transplantation: a retrospective case series study. BMC ORAL HEALTH, 21(1). doi:10.1186/s12903-021-01908-2

Mou, J., Wang, B., Liu, Y., Zhao, F., Wu, Y., Xu, W., . . . Yuan, C. (2022). FER1L4：A long non-coding RNA with multiple roles in the occurrence and development of tumors. Current pharmaceutical design. doi:10.2174/1381612828666220324141016

Nazia, T., Ullah, A., Tahir, A., Rashid, H. U., Rehman, T. U., Danish, S., . . . Akca, H. (2021). Strategies for reducing Cd concentration in paddy soil for rice safety. JOURNAL OF CLEANER PRODUCTION, 316. doi:10.1016/j.jclepro.2021.128116

Ngoude, J. X. E., Moor, V. J. A., Nadia-Flore, T. T., Agoons, B. B., Marcelle, G. G. C., MacBrain, E. E., . . . Nkeck, J. R. (2021). Relationship between periodontal diseases and newly-diagnosed metabolic syndrome components in a sub-Saharan population: a cross sectional study. BMC ORAL HEALTH, 21(1). doi:10.1186/s12903-021-01661-6

Ngu, S. F., Tse, K. Y., Chu, M. M. Y., Ngan, H. Y. S., & Chan, K. K. L. (2021). Olaparib dose re-escalation in ovarian cancer patients who experienced severe and/or uncommon adverse events: A case series. Asia-Pacific Journal of Clinical Oncology, 17(S3), 3-11. doi:10.1111/ajco.13584

Özkur, E., Sert, C., Kıvanç Altunay, İ., Yıldırırmak, Z. Y., Genç, D. B., Vural, S., & Erdem, Y. (2021). Cutaneous manifestations in pediatric oncology patients. Pediatric Dermatology, 38(1), 58-65. doi:10.1111/pde.14375

Parthasarathy, V., Menon, A. R., & Devaranavadagi, B. (2021). Target Fishing of Calactin, Calotropin and Calotoxin Using Reverse Phar-macophore Screening and Consensus Inverse Docking Approach. Current Drug Discovery Technologies, 18(6). doi:10.2174/1570163817666201207143958

Popa, G. V., Costache, A., Badea, O., Cojocaru, M. O., Mitroi, G., Lazar, A. C., . . . Mogoanta, L. (2021). Histopathological and immunohistochemical study of periodontal changes in chronic smokers. ROMANIAN JOURNAL OF MORPHOLOGY AND EMBRYOLOGY, 62(1), 209-217. doi:10.47162/RJME.62.1.20

Prasad, S., Black, S. M., Zhu, J. L., Sharma, S., & Jacobe, H. (2021). Morphea patients with mucocutaneous involvement: A cross-sectional study from the Morphea in Adults and Children (MAC) cohort. Journal of the American Academy of Dermatology, 85(1), 114-120. doi:10.1016/j.jaad.2020.10.093

Radaic, A., Ganther, S., Kamarajan, P., Grandis, J., Yom, S. S., & Kapila, Y. L. (2021). Paradigm shift in the pathogenesis and treatment of oral cancer and other cancers focused on the oralome and antimicrobial-based therapeutics. PERIODONTOLOGY 2000, 87(1), 76-93. doi:10.1111/prd.12388

Rahman, R. A., Lamarca, A., Hubner, R. A., Valle, J. W., & McNamara, M. G. (2021). The microbiome as a potential target for therapeutic manipulation in pancreatic cancer. CANCERS, 13(15). doi:10.3390/cancers13153779

Ray, M., Carney, H. C., Boynton, B., Quimby, J., Robertson, S., St Denis, K., . . . Wright, B. (2021). 2021 AAFP Feline Senior Care Guidelines. JOURNAL OF FELINE MEDICINE AND SURGERY, 23(7), 613-638. doi:10.1177/1098612X211021538

Rosa, R. B., Bianchi, M. V., Ribeiro, P. R., Argenta, F. F., Vielmo, A., de Sousa, F. A. B., . . . Sonne, L. (2021). Comparison of immunohistochemical profiles of ovarian germ cells in dysgerminomas of a captive maned wolf and domestic dogs. Journal of Veterinary Diagnostic Investigation, 33(4), 772-776. doi:10.1177/10406387211019959

Ruan, J., & Han, B. (2021). Effective treatment of aplastic anemia secondary to chemoradiotherapy using cyclosporine A. Chinese Medical Journal, 134(19), 2356-2358. doi:10.1097/CM9.0000000000001365

Russell, J. J., Grisanti, L. A., Brown, S. M., Bailey, C. A., Bender, S. B., & Chandrasekar, B. (2021). Reversion inducing cysteine rich protein with Kazal motifs and cardiovascular diseases: The RECKlessness of adverse remodeling. CELLULAR SIGNALLING, 83. doi:10.1016/j.cellsig.2021.109993

Schoenaker, M. H. D., Takada, S., van Deuren, M., Dommering, C. J., Henriët, S. S. V., Pico, I., . . . Kaanders, J. H. A. M. (2021). Considerations for radiotherapy in Bloom Syndrome: A case series. European Journal of Medical Genetics, 64(10). doi:10.1016/j.ejmg.2021.104293

Sharma, M., Chopra, C., Mehta, M., Sharma, V., Mallubhotla, S., Sistla, S., . . . Bhushan, I. (2021). An Insight into Vaginal Microbiome Techniques. LIFE-BASEL, 11(11). doi:10.3390/life11111229

Shiga, T., Nakata, M., Miwa, Y., Chambers, J. K., Uchida, K., Sasaki, N., . . . Nakayama, H. (2021). A retrospective study (2006-2020) of cytology and biopsy findings in pet rabbits (Oryctolagus cuniculus), ferrets (Mustela putorius furo) and four-toed hedgehogs (Atelerix albiventris) seen at an exotic animal clinic in Tokyo, Japan. JOURNAL OF EXOTIC PET MEDICINE, 38, 11-17. doi:10.1053/j.jepm.2021.03.008

Shimizu, T., Kuboki, Y., Lin, C. C., Yonemori, K., Yanai, T., Faller, D. V., . . . Kim, K. P. (2022). A Phase 1 Study of Sapanisertib (TAK-228) in East Asian Patients with Advanced Nonhematological Malignancies. TARGETED ONCOLOGY, 17(1), 15-24. doi:10.1007/s11523-021-00855-w

Sobocki, B. K., Basset, C. A., Bruhn-Olszewska, B., Olszewski, P., Szot, O., Kazmierczak-Siedlecka, K., . . . Leone, A. (2022). Molecular Mechanisms Leading from Periodontal Disease to Cancer. INTERNATIONAL JOURNAL OF MOLECULAR SCIENCES, 23(2). doi:10.3390/ijms23020970

Sowińska-Przepiera, E., Starzyński, D., Syrenicz, A., Dziuba, I., Wiszniewska, B., & Rzeszotek, S. (2021). Neuroendocrine tumors: Clinical, histological and immunohistochemical perspectives and case report— mature teratoma in a 16-year-old girl. Pathophysiology, 28(3), 373-386. doi:10.3390/PATHOPHYSIOLOGY28030025

Spodzieja, K., & Olczak-Kowalczyk, D. (2022). Premature Loss of Deciduous Teeth as a Symptom of Systemic Disease: A Narrative Literature Review. INTERNATIONAL JOURNAL OF ENVIRONMENTAL RESEARCH AND PUBLIC HEALTH, 19(6). doi:10.3390/ijerph19063386

Strait, R. H., Barnes, S., & Smith, D. K. (2021). Associations between oral health and general health: a survey-wide association study of the NHANES. COMMUNITY DENTAL HEALTH, 38(2), 83-88. doi:10.1922/CDH_00121Strait06

Takehara, K., Matsumoto, T., Hamanishi, J., Hasegawa, K., Matsuura, M., Miura, K., . . . Takeshima, N. (2021). Phase 2 single-arm study on the safety of maintenance niraparib in japanese patients with platinum-sensitive relapsed ovarian cancer. Journal of Gynecologic Oncology, 32(2), 1-11. doi:10.3802/jgo.2021.32.e21

Tawde, V., Chaurasia, S., Gupta, S., Rastogi, R., Dantuluri, A. K., Liu, W., & McMahon, S. (2021). The Trend of Commercialized Products with Smart Bioresorbable Polymers in Pharmaceuticals and Medical Devices. Pharma Times, 53(12), 23-30.

Toyohiro, K., Yuki, N., Shoko, Y., Shiraishi, T., Mamoru, S., Daisuke, I., . . . Kenji, Y. (2021). Development of flash cards to teach about lesions in the jaws and maxillary sinuses. Oral Radiology, 37(2), 231-235. doi:10.1007/s11282-020-00435-0

Ueda, N., Aoki, K., Shimotsuji, H., Nakashima, C., Kawakami, M., Imai, Y., & Kirita, T. (2021). Oral risk factors associated with medication-related osteonecrosis of the jaw in patients with cancer. Journal of Bone and Mineral Metabolism, 39(4), 623-630. doi:10.1007/s00774-020-01195-x

Varsha, K. K., Maheshwari, A. P., & Nampoothiri, K. M. (2021). Accomplishment of probiotics in human health pertaining to immunoregulation and disease control. CLINICAL NUTRITION ESPEN, 44, 26-37. doi:10.1016/j.clnesp.2021.06.020

Vernerova, A., Krcmova, L. K., Heneberk, O., Radochova, V., Strouhal, O., Kasparovsky, A., . . . Svec, F. (2021). Chromatographic method for the determination of inflammatory biomarkers and uric acid in human saliva. TALANTA, 233. doi:10.1016/j.talanta.2021.122598

Wang, H., Shan, B., & Shen, W. (2021). Anlotinib in patients with recurrent platinum-resistant or -refractory ovarian carcinoma: A prospective, single-arm, single-center, phase II clinical study. Annals of Oncology, 32, S733. doi:10.1016/j.annonc.2021.08.1173

Wang, H., Shen, W., & Shan, B. (2021). Anlotinib in patients with recurrent platinum-resistant or refractory ovarian carcinoma: A prospective, single-arm, singlecenter, phase II clinical study. Journal of Clinical Oncology, 39(15 SUPPL). doi:10.1200/JCO.2021.39.15_suppl.e17524

Wang, W., Yan, Y. X., Guo, Z., Hou, H. F., Garcia, M., Tan, X. R., . . . European Assoc Predictive, P. (2021). All around suboptimal health - a joint position paper of the Suboptimal Health Study Consortium and European Association for Predictive, Preventive and Personalised Medicine. EPMA JOURNAL, 12(4), 403-433. doi:10.1007/s13167-021-00253-2

Watanabe, H., Ariji, Y., Fukuda, M., Kuwada, C., Kise, Y., Nozawa, M., . . . Ariji, E. (2021). Deep learning object detection of maxillary cyst-like lesions on panoramic radiographs: preliminary study. Oral Radiology, 37(3), 487-493. doi:10.1007/s11282-020-00485-4

Wei, L. Y., Kok, S. H., Lee, Y. C., Chiu, W. Y., Wang, J. J., Cheng, S. J., . . . Lee, J. J. (2021). Prognosis of medication-related osteonecrosis of the jaws in cancer patients using antiresorptive agent zoledronic acid. Journal of the Formosan Medical Association, 120(8), 1572-1580. doi:10.1016/j.jfma.2020.11.017

Wen, K., Fang, X., Yang, J., Yao, Y., Nandakumar, K. S., Salem, M. L., & Cheng, K. (2021). Recent research on flavonoids and their biomedical applications. Current Medicinal Chemistry, 28(5), 1042-1066. doi:10.2174/0929867327666200713184138

Wu, B., Sodji, Q. H., & Oyelere, A. K. (2022). Inflammation, Fibrosis and Cancer: Mechanisms, Therapeutic Options and Challenges. CANCERS, 14(3). doi:10.3390/cancers14030552

Xu, G., & Lu, Y. (2021). Progression of Cabozantinib-Related Osteonecrosis Mimicking Metastases on Bone Scan. Clinical Nuclear Medicine, 46(7), 592-594. doi:10.1097/RLU.0000000000003522

Yamamoto, H., Wada, Y., Ito, S., Kawase, T., & Tamura, M. (2022). Iron deficiency anemia improved by dental implantation: A case report. Saudi Journal of Medicine and Medical Sciences, 10(1), 67-71. doi:10.4103/sjmms.sjmms_353_21

Younan, R. G., Raad, R. A., Sawan, B. Y., & Said, R. (2021). Aplastic anemia secondary to dual cancer immunotherapies a physician nightmare: case report and literature review. Allergy, Asthma and Clinical Immunology, 17(1). doi:10.1186/s13223-021-00616-4

Yuan, S., Fang, C., Leng, W. D., Wu, L., Li, B. H., Wang, X. H., . . . Zeng, X. T. (2021). Oral microbiota in the oral-genitourinary axis: identifying periodontitis as a potential risk of genitourinary cancers. MILITARY MEDICAL RESEARCH, 8(1). doi:10.1186/s40779-021-00344-1

Zhang, X., Zhao, M., & Zheng, C. (2021). Drug fever induced by carboplatin-based regimens: Higher incidence in a women's hospital. Taiwanese Journal of Obstetrics and Gynecology, 60(5), 882-887. doi:10.1016/j.tjog.2021.07.018

Zheng, S., Lin, R., Chen, S., Zheng, J., Lin, Z., Zhang, Y., . . . Miao, W. (2021). Characterization of the benign lesions with increased 68Ga-FAPI-04 uptake in PET/CT. Annals of Nuclear Medicine, 35(12), 1312-1320. doi:10.1007/s12149-021-01673-w

Zhou, L. Y., Lin, S. N., Rieder, F., Chen, M. H., Zhang, S. H., & Mao, R. (2021). Noncoding RNAs as Promising Diagnostic Biomarkers and Therapeutic Targets in Intestinal Fibrosis of Crohn's Disease: The Path from Bench to Bedside. Inflammatory Bowel Diseases, 27(7), 971-982. doi:10.1093/ibd/izaa321

Zhuang, Z. H., Gao, M., Lv, J., Yu, C. Q., Guo, Y., Bian, Z., . . . China Kadoorie Biobank, C. K. B. C. (2021). Associations of toothbrushing behaviour with risks of vascular and nonvascular diseases in Chinese adults. EUROPEAN JOURNAL OF CLINICAL INVESTIGATION, 51(12). doi:10.1111/eci.13634
